# Effects of gastroprotectant drugs for the prevention and treatment of peptic ulcer disease and its complications: a meta-analysis of randomised trials

**DOI:** 10.1016/S2468-1253(18)30037-2

**Published:** 2018-02-21

**Authors:** Benjamin Scally, Jonathan R Emberson, Enti Spata, Christina Reith, Kelly Davies, Heather Halls, Lisa Holland, Kate Wilson, Neeraj Bhala, Christopher Hawkey, Marc Hochberg, Richard Hunt, Loren Laine, Angel Lanas, Carlo Patrono, Colin Baigent

**Affiliations:** a Emergency Department, Glasgow Royal Infirmary, Glasgow, UK; b Medical Research Council Population Health Research Unit, Nuffield Department of Population Health, Oxford, UK; c Clinical Trial Service Unit and Epidemiological Studies Unit, Nuffield Department of Population Health, Oxford, UK; d Gastroenterology and Liver Unit, Queen Elizabeth Hospital, Mindelsohn Way, Birmingham, UK; e Institute of Applied Health Research, University of Birmingham, Edgbaston, UK; f Nottingham Digestive Diseases Centre, Queen's Medical Centre, Nottingham, UK; g Department of Medicine and Department of Epidemiology and Public Health, University of Maryland School of Medicine, Baltimore, MD, USA; h Division of Gastroenterology and Farncombe Family Digestive Health Research Institute, McMaster University Health Science Centre, Hamilton, ON, Canada; i Section of Digestive Diseases, Yale School of Medicine, New Haven, CT, USA; j VA Connecticut Healthcare System, West Haven, CT, USA; k Service of Digestive Diseases, University Clinic Hospital, University of Zaragoza, IIS Aragón, CIBERehd, Zaragoza, Spain; l Department of Pharmacology, Catholic University School of Medicine, Rome, Italy

## Abstract

**Background:**

Gastroprotectant drugs are used for the prevention and treatment of peptic ulcer disease and might reduce its associated complications, but reliable estimates of the effects of gastroprotectants in different clinical settings are scarce. We aimed to examine the effects of proton-pump inhibitors (PPIs), prostaglandin analogues, and histamine-2 receptor antagonists (H2RAs) in different clinical circumstances by doing meta-analyses of tabular data from all relevant unconfounded randomised trials of gastroprotectant drugs.

**Methods:**

We searched MEDLINE and Embase from Jan 1, 1950, to Dec 31, 2015, to identify unconfounded, randomised trials of a gastroprotectant drug (defined as a PPI, prostaglandin analogue, or H2RA) versus control, or versus another gastroprotectant. Two independent researchers reviewed the search results and extracted the prespecified outcomes and key characteristics for each trial. We did meta-analyses of the effects of gastroprotectant drugs on ulcer development, bleeding, and mortality overall, according to the class of gastroprotectant, and according to the individual drug within a gastroprotectant class.

**Findings:**

We identified comparisons of gastroprotectant versus control in 849 trials (142 485 participants): 580 prevention trials (110 626 participants), 233 healing trials (24 033 participants), and 36 trials for the treatment of acute upper gastrointestinal bleeding (7826 participants). Comparisons of one gastroprotectant drug versus another were available in 345 trials (64 905 participants), comprising 160 prevention trials (32 959 participants), 167 healing trials (28 306 participants), and 18 trials for treatment of acute upper gastrointestinal bleeding (3640 participants). The median number of patients in each trial was 78 (IQR 44·0–210·5) and the median duration was 1·4 months (0·9–2·8). In prevention trials, gastroprotectant drugs reduced development of endoscopic ulcers (odds ratio [OR] 0·27, 95% CI 0·25–0·29; p<0·0001), symptomatic ulcers (0·25, 0·22–0·29; p<0·0001), and upper gastrointestinal bleeding (0·40, 0·32–0·50; p<0·0001), but did not significantly reduce mortality (0·85, 0·69–1·04; p=0·11). Larger proportional reductions in upper gastrointestinal bleeding were observed for PPIs than for other gastroprotectant drugs (PPIs 0·21, 99% CI 0·12–0·36; prostaglandin analogues 0·63, 0·35–1·12; H2RAs 0·49, 0·30–0·80; p_het_=0·0005). Gastroprotectant drugs were effective in preventing bleeding irrespective of the use of non-steroidal anti-inflammatory drugs (p_het_=0·56). In healing trials, gastroprotectants increased endoscopic ulcer healing (3·49, 95% CI 3·28–3·72; p<0·0001), with PPIs more effective (5·22, 99% CI 4·00–6·80) than prostaglandin analogues (2·27, 1·91–2·70) and H2RAs (3·80, 3·44–4·20; p_het_<0·0001). In trials among patients with acute bleeding, gastroprotectants reduced further bleeding (OR 0·68, 95% CI 0·60–0·78; p<0·0001), blood transfusion (0·75, 0·65–0·88; p=0·0003), further endoscopic intervention (0·56, 0·45–0·70; p<0·0001), and surgery (0·72, 0·61–0·84; p<0·0001), but did not significantly reduce mortality (OR 0·90, 0·72–1·11; p=0·31). PPIs had larger protective effects than did H2RAs for further bleeding (p_het_=0·0107) and blood transfusion (p_het_=0·0130).

**Interpretation:**

Gastroprotectants, in particular PPIs, reduce the risk of peptic ulcer disease and its complications and promote healing of peptic ulcers in a wide range of clinical circumstances. However, this meta-analysis might have overestimated the benefits owing to small study bias.

**Funding:**

UK Medical Research Council and the British Heart Foundation.

## Introduction

Worldwide, peptic ulcer disease is responsible for substantial premature mortality, with much of the burden in low-income and middle-income countries.^1^, ^2^ Peptic ulcer disease comprises both gastric and duodenal ulcers—defects that penetrate, respectively, beyond the muscularis mucosae of the gastric or duodenal mucosa—and its complications can include upper gastrointestinal bleeding, perforation and, rarely, gastric outlet obstruction.^3^, ^4^ Gastroprotectant drugs, defined here as proton-pump inhibitors (PPIs), prostaglandin analogues, and histamine-2 receptor antagonists (H2RAs), have been developed for the protection of the mucosa, healing of mucosal damage, and stabilisation of gastrointestinal bleeding, and are prescribed for the prevention of peptic ulcer disease, to promote healing, and as treatment for bleeding complications.

**Research in context**

**Evidence before the study**

We searched MEDLINE and Embase from Jan 1, 1950, to Dec 31, 2015, for randomised controlled trials of gastroprotectant drugs (including proton-pump inhibitors [PPIs], histamine-2 receptor antagonists, and prostaglandin analogues), with no language restrictions. These searches revealed a very large number of studies that have assessed the use of such therapy for the prevention or treatment of peptic ulcer disease. Previous systematic reviews and meta-analyses have reported varying efficacy for specific drugs, or drug classes, on certain peptic ulcer disease outcomes in particular clinical settings, often in patients treated with non-steroidal anti-inflammatory drugs (NSAIDs). However, a comprehensive summary of the relative and absolute effects of different gastroprotectant drugs on different types of upper gastrointestinal outcomes, with or without NSAID use, and in the context both of prevention and treatment, has not been reported.

**Added value of the study**

This meta-analysis of more than 1200 trials included around 200 000 participants and quantified the relative treatment effects of available gastroprotectants in the settings of ulcer prevention, ulcer healing, and treatment of acute upper gastrointestinal bleeding. The findings provide evidence for benefits of gastroprotectant therapy in all three clinical contexts, with PPIs showing consistent superiority to other agents. The relative benefits of gastroprotectants were of broadly similar magnitude irrespective of whether patients were taking NSAIDs. In the absence of large-scale randomised trials, however, some uncertainty remains about whether the effect size estimates in this meta-analysis were inflated by small study bias, and insufficient reliable information is available on the safety of such treatments.

**Implications of all the available evidence**

This study indicates that, in the context of peptic ulcer disease, gastroprotectants—and in particular PPIs—are effective in ulcer prevention, ulcer healing, and in reducing rebleeding. The relative benefits appear similar irrespective of concomitant NSAID use. Reliable information is still needed about the long-term safety of PPIs; in particular, there is concern that PPIs might have adverse cardiovascular effects. The large ongoing COMPASS trial of pantoprazole versus placebo in 17 000 patients with stable cardiovascular disease might provide useful safety information, and could also help to determine the true size of any beneficial effects.

The most frequent causes of peptic ulcer disease are *Helicobacter pylori* infection and the use of non-steroidal anti-inflammatory drugs (NSAIDs), including aspirin.^3^ NSAIDs are among the most widely used drugs in the world and are known to substantially increase the risk of upper gastrointestinal complications^5^ (probably as a consequence of inhibited mucosal prostaglandin production). Optimal use of gastroprotectant drugs might therefore help to reduce the global burden of peptic ulcer disease and its complications. Clinical guidelines currently recommend that PPIs are used as first-choice gastroprotectant drugs, supported by systematic reviews and meta-analyses in particular clinical settings,^6^, ^7^, ^8^ but to date no comprehensive effort has been made to bring together all of the evidence from randomised trials of gastroprotectant drugs in different clinical contexts. In particular, reliable estimates of the effects of different gastroprotectant drugs in specific clinical circumstances (eg, in the context of prevention, healing, or acute bleeding), at different anatomical locations (gastric or duodenal), and according to concomitant NSAID use are not available.

We aimed to examine the effects of PPIs, prostaglandin analogues, and H2RAs in different clinical circumstances by doing meta-analyses of tabular data from all relevant unconfounded randomised trials of gastroprotectant drugs. Here we describe the main findings of these analyses.

## Methods

### Search strategy and selection criteria

Full details of the search strategy, including search terms used, are provided in the appendix (pp 34–36). We searched MEDLINE and Embase using Ovid SP and the Cochrane strategy^9^ from Jan 1, 1950, to Dec 31, 2015, inclusive, with no language restrictions. Studies were eligible if they were prospective clinical trials with adequate randomisation (ie, randomised sequence generation with robust allocation concealment) and were unconfounded (ie, protocol-mandated use of non-gastroprotectant drugs did not differ between treatment groups). We excluded trials if they were conducted exclusively among participants with non-peptic upper gastrointestinal bleeding (eg, from varices), were of less than 2 weeks' treatment duration (except in acute trials of upper gastrointestinal bleeding, in which there were no duration constraints), were done in a critical care setting, or if treatment was given less frequently than once daily. The review of search results was done independently by two authors (two of HH, KD, KW, and LH).

We included trials if they randomly assigned participants to at least one gastroprotectant drug—defined as a PPI, prostaglandin analogue, or H2RA—and if they included a comparison of a gastroprotectant versus placebo or open control (no gastroprotectant; henceforth referred to as control), or a gastroprotectant of a given class versus a gastroprotectant of another class.

Review of the data indicated that the published trials had assessed gastroprotectant drugs in three main clinical scenarios: people with no ulcer at baseline (so-called prevention trials); people with a non-bleeding ulcer at baseline (so-called healing trials); and people presenting with upper gastrointestinal bleeding at baseline, with or without a confirmed diagnosis of peptic ulcer disease (so-called acute upper gastrointestinal bleeding treatment trials).

### Data analyses

Two authors (two of HH, KD, KW, and LH) independently extracted key trial characteristics (including trial design, number of patients randomised, and indication) and prespecificed outcomes, with extracted information entered into a customised database. Where possible, a different pair of reviewers to those who extracted the data assessed the trial for inclusion. We used the trialists' own definitions of outcomes as far as possible (appendix p 2). When definitions were inconsistent, events were adjudicated by at least two reviewers, including one clinician (NB or BS), until resolved. We recorded the number of patients who experienced each outcome; patients with multiple experiences of the same outcome were only counted once. However, where possible, outcome data were extracted for separate mucosal sites (gastric or duodenal). The mortality outcome included deaths recorded up to the end of each trial.

We did intention-to-treat analyses of the effects of gastroprotectant drugs overall, according to the class of gastroprotectant, and according to the individual drug within a gastroprotectant class. Only trials with at least one relevant event were included in analyses, and all statistical tests were two-sided. The main meta-analyses employed inverse-variance weighted methods for combining 2 × 2 contingency tables, as previously described.^10^ To allow for multiple subdivisions of the data, only summary odds ratios (ORs) are presented with 95% CIs; all other ORs are presented with 99% CIs. We calculated absolute effects from the crude differences between treatment groups.

We did exploratory analyses according to drug dose; NSAID use at randomisation; reported method of allocation concealment (sealed envelopes, closed list of random numbers, or unspecified method *vs* secure method) and method of blinding (double blind *vs* other). Additionally, we used a network meta-analysis approach to combine the direct and indirect randomised evidence (using the “netmeta” function in R). We created funnel plots to help to visually assess how the results from the larger trials (nearer the tip of each funnel) compared with the results from the smaller trials (nearer the base), with Egger's statistics used to test for bias through funnel plot asymmetry.

We did all statistical analyses using SAS version 9.3 and R for Windows version 3.2.4.

### Role of the funding source

The funders had no role in study design, data collection, data analysis, data interpretation, or writing of the report. The corresponding author had full access to the data and had final responsibility for the decision to submit for publication.

## Results

**Figure 1:**
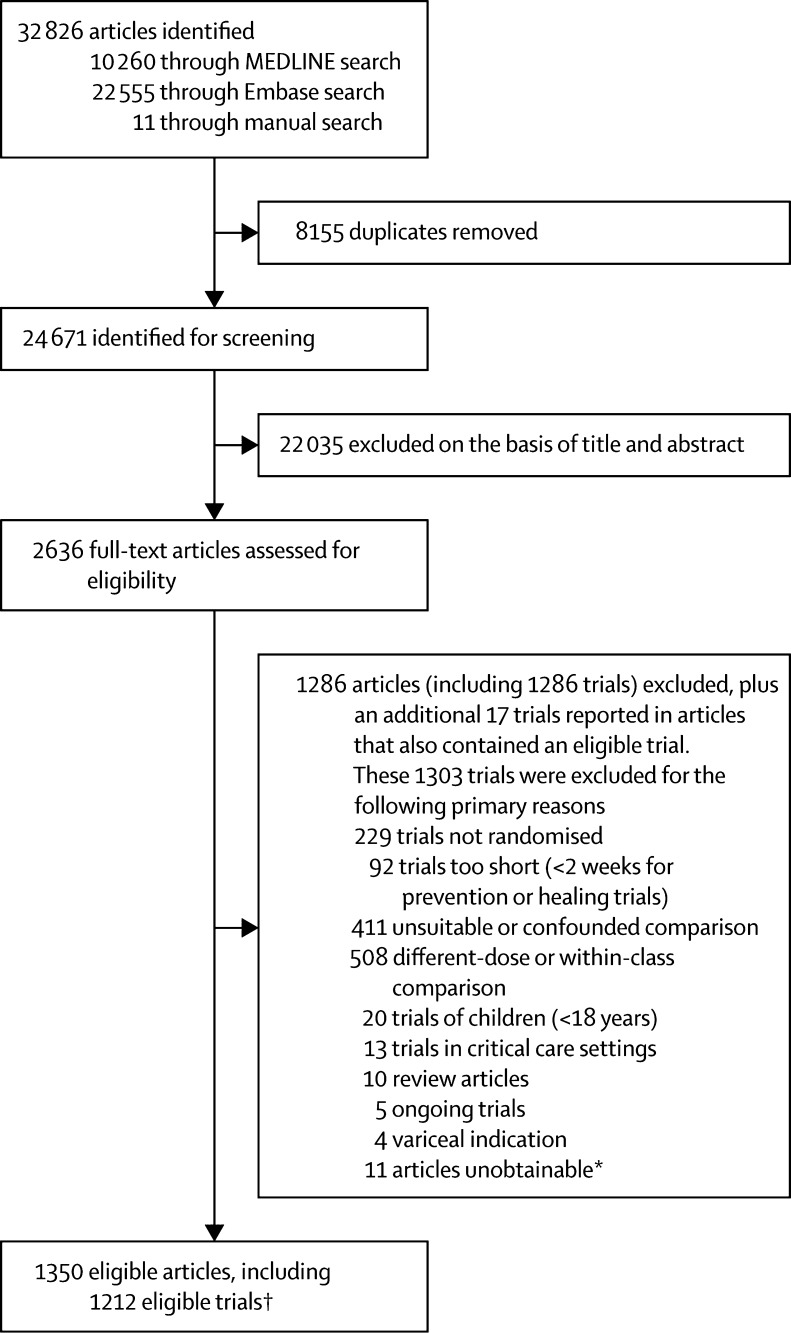
PRISMA diagram *Full-text copies of these articles were unobtainable from all available sources including the British Library. †Some trials were published in more than one article (and, conversely, a few articles reported results from more than one trial).

We found 24 671 titles and abstracts from which we identified 1212 unconfounded randomised trials for analysis (see PRISMA^11^ diagram [figure 1]; details of included trials and their main design features are in the appendix, pp 37–91). Data from comparisons of a gastroprotectant versus control were available in 849 trials with a total of 142 485 participants (table). Endpoints of a relevant type were reported for 55% of prevention trials, 92% of healing trials, and 94% of acute upper gastrointestinal bleeding treatment trials (table). Data from comparisons of one gastroprotectant versus a gastroprotectant of a different class were available in 345 trials (table). Endpoints of a relevant type were reported for 54% of prevention trials, 92% of healing trials, and 94% of acute bleeding trials (table). 11 trials had a comparison both of a gastroprotectant versus control and of one gastroprotectant versus another gastroprotectant. 29 trials did not report the number of participants randomised, and therefore were not included in analyses; this exclusion occurred after initial assessments of eligibility. The median number of participants in each trial was 78 (IQR 44·0–210·5): 97 (IQR 50–278) in the prevention trials, 59 (36–121) in the healing trials, and 147 (70–289) in the acute upper gastrointestinal bleeding trials. The median duration of the trials was 1·4 months (IQR 0·9–2·8): 180 days (84–360) in the prevention trials, 30 days (28–42) in the healing trials and 3 days (2–4) in the acute upper gastrointestinal bleeding trials.

**Table:** Availability of data for measuring the effects of gastroprotectants

	**Prevention trials**		**Healing trials**		**Acute bleeding trials**	
	Trials	Participants	Trials	Participants	Trials	Participants
**Gastroprotectant *vs* control**						
Endoscopic ulcer*	162 (28%)	31580 (29%)	207 (89%)	22086 (92%)	··	··
Symptomatic ulcer*	73 (13%)	21505 (19%)	··	··	··	··
All-cause mortality	185 (32%)	57472 (52%)	43 (18%)	6243 (26%)	31 (86%)	7596 (97%)
Further bleeding, endoscopy, surgery, or transfusion	··	··	··	··	33 (92%)	7662 (98%)
Bleeds, perforations, or obstructions	115 (20%)	41764 (38%)	··	··	··	··
No data available for any of the above outcomes	259 (45%)	33122 (30%)	18 (8%)	1250 (5%)	2 (6%)	106 (1%)
Total	580	110626	233	24 033	36	7826
**Gastroprotectant *vs* a different class of gastroprotectant**						
Endoscopic ulcer*	22 (14%)	5781 (18%)	150 (90%)	25494 (90%)	··	··
Symptomatic ulcer*	15 (9%)	3199 (10%)	··	··	··	··
All-cause mortality	61 (38%)	10716 (33%)	68 (41%)	11739 (41%)	16 (89%)	3478 (96%)
Further bleeding, endoscopy, surgery, or transfusion	··	··	··	··	17 (94%)	3518 (97%)
Bleeds, perforations, or obstructions	41 (26%)	8065 (24%)	··	··	··	··
No data available for any of the above outcomes	74 (46%)	16813 (51%)	14 (8%)	2661 (9%)	1 (6%)	122 (3%)
Total	160	32959	167	28306	18	3640

Trials in which an event is reported as not occurring (ie, 0 *vs* 0) are considered to have data available. In addition to the trials included in this table, there were 29 eligible trials for which the number of patients randomised was not available. No events were reported in any of these 29 trials.

* Duodenal, gastric, or any reported ulcer.

**Figure 2:**
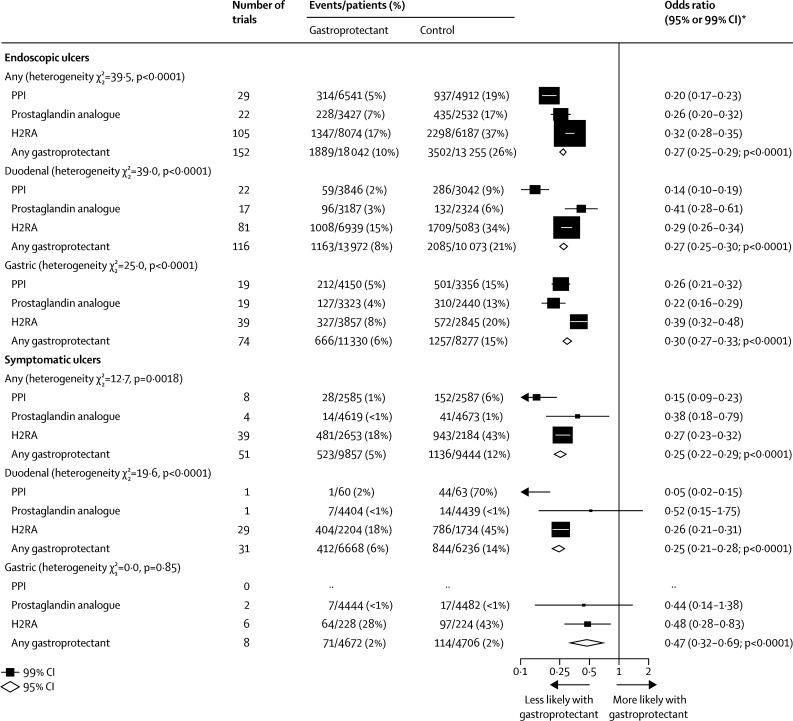
Prevention trials: effects of gastroprotectants on endoscopic and symptomatic ulcers For 97 trials for the endoscopic outcome and 36 trials for the symptomatic outcome, in which the total number of ulcers was not reported but the breakdown was, we assumed that the total number of ulcers was equal to gastric plus duodenal: 1256 of 7414 events *vs* 2277 of 5766 events for endoscopic ulcers and 469 of 2492 events *vs* 927 of 2021 events for symptomatic ulcers. PPI=proton-pump inhibitor. H2RA=histamine-2 receptor antagonist. *Summary odds ratios, indicated by diamonds, are presented with 95% CIs; all other odds ratios are presented with 99% CIs.

In prevention trials, we found that overall, compared with control, allocation to a gastroprotectant drug resulted in a substantial reduction in the odds of an endoscopic ulcer (OR 0·27 [95% CI 0·25–0·29]; absolute benefit 160 events [151–168] avoided per 1000 patients allocated treatment; p<0·0001). PPIs appeared to be more effective (OR 0·20 [99% CI 0·17–0·23]) than prostaglandin analogues (0·26 [0·20–0·32]) or H2RAs (0·32 [0·28–0·35]; p_het_<0·0001; figure 2). The relative effectiveness of particular gastroprotectant classes on duodenal ulcers and gastric ulcers also differed significantly: for trials of a gastroprotectant versus control in duodenal ulcer prevention, PPIs were the most effective gastroprotectant class, followed by H2RAs and finally prostaglandin analogues (figure 2), which was consistent with the ordering implied by the results of trials comparing one gastroprotectant class with another gastroprotectant class (appendix p 3). For prevention of gastric ulcers, however, the analogous ordering of effectiveness was prostaglandin analogues, followed by PPIs and finally H2RAs, and this was again consistent with the ordering implied by the results of trials directly comparing different gastroprotectant classes. PPIs were more effective at reducing the risk of duodenal ulcers than of gastric ulcers (p<0·0001; figure 2).

Few data were available with which to assess the relative efficacy of drugs within each gastroprotectant class, but the available trials provided no evidence that any individual PPI or prostaglandin analogue was more effective for the prevention of endoscopic ulcer than any other in the same class and we found little evidence of heterogeneity in treatment effects among H2RAs (p_het_=0·0286; appendix p 4).

Overall, compared with control, allocation to a gastroprotectant drug resulted in a substantial reduction in the odds of a symptomatic ulcer (OR 0·25 [95% CI 0·22–0·29]; absolute benefit 67 [59–75] events avoided per 1000 allocated treatment; p<0·0001; figure 2). However, because there were only about a third as many symptomatic ulcers as endoscopic ulcers available for analysis, the relative effectiveness of individual gastroprotectant classes on symptomatic ulcers was less clear. Overall, PPIs were more effective than were H2RAs and prostaglandin analogues (between the three classes p_het_=0·0018), but data were insufficient to provide separate estimates of effectiveness for duodenal and gastric ulcers (figure 2). We found no clear evidence that any individual PPI or prostaglandin analogue was more effective for the prevention of symptomatic ulcer than any other of the same class (appendix p 5), and only weak evidence of heterogeneity in treatment effects among H2RAs (p_het_=0·0447; appendix p 5).

**Figure 3:**
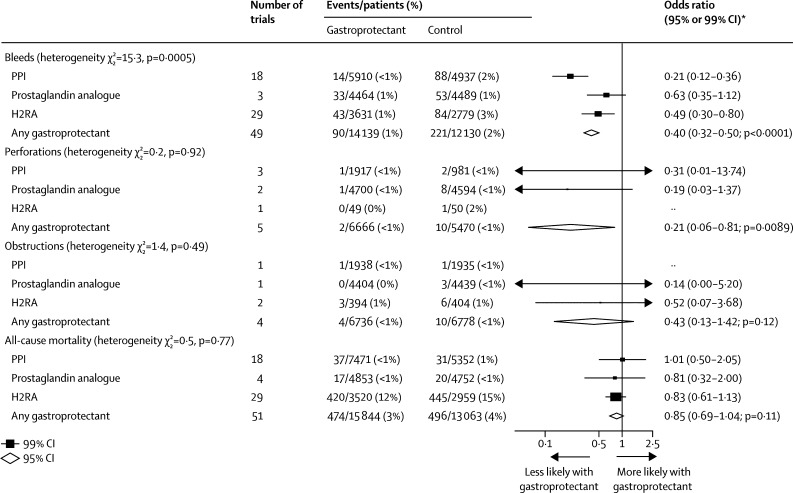
Prevention trials: effects of gastroprotectants on ulcer complications and all-cause mortality PPI=proton-pump inhibitor. H2RA=histamine-2 receptor antagonist. *Summary odds ratios, indicated by diamonds, are presented with 95% CIs; all other odds ratios are presented with 99% CIs.

Overall, compared with control, allocation to a gastroprotectant resulted in a substantial reduction in the odds of upper gastrointestinal bleeding (OR 0·40 [95% CI 0·32–0·50], absolute benefit 12 [9–15] avoided per 1000 allocated treatment; p<0·0001; figure 3). The effects of PPIs on ulcer bleeding were similar after exclusion of data from the COGENT trial^12^ (all trials OR 0·21 [95% CI 0·14–0·32]; after excluding COGENT 0·17 [0·10–0·28]). Larger proportional reductions in ulcer bleeding were observed for PPIs than for prostaglandin analogues or H2RAs (figure 3). We found no evidence that individual PPIs differed in their effects on bleeding (p_het_=0·48; appendix p 6). Ranitidine appeared more effective than other H2RAs for the prevention of ulcer bleeding (p_het_=0·0012; appendix p 6). Few data were available on perforations and obstructions, but overall, compared with control, allocation to a gastroprotectant resulted in a substantial reduction in the odds of gastrointestinal perforation (OR 0·21, 0·06–0·81; p=0·0089) and a non-significant reduction in the odds of gastrointestinal obstruction (0·43, 0·13–1·42; p=0·12; figure 3). Allocation to a gastroprotectant had no significant effect on all-cause mortality, either overall or for any individual gastroprotectant class (figure 3).

**Figure 4:**
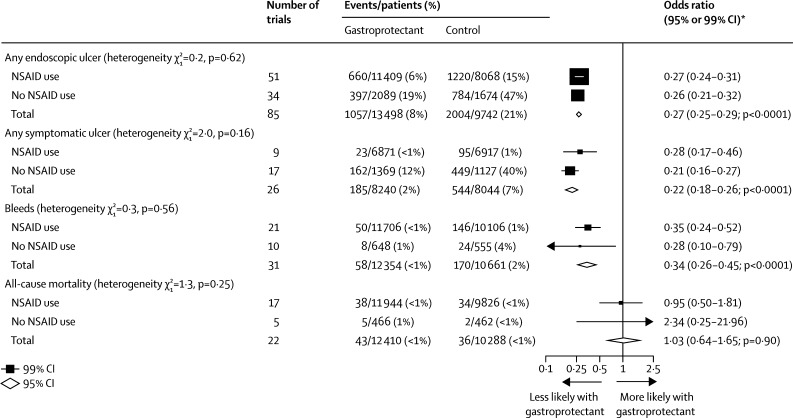
Prevention trials: effects of gastroprotectants on endoscopic and symptomatic ulcers, bleeds, and all-cause mortality, subdivided by use of NSAIDs We categorised trials by NSAID use, defining NSAID use as at least 80% of patients took a traditional NSAID, coxib, or aspirin, and no NSAID use as fewer than 80% of patients took a traditional NSAID, coxib, or aspirin. NSAID=non-steroidal anti-inflammatory drugs. *Summary odds ratios, indicated by diamonds, are presented with 95% CIs; all other odds ratios are presented with 99% CIs.

Overall, compared with control, the proportional effects of gastroprotectants on endoscopic ulcers, symptomatic ulcers, bleeds, and mortality were similar irrespective of NSAID use at the randomisation visit (all heterogeneity p values non-significant; figure 4). The results for trials in which information about NSAID use at baseline was unavailable were similar to those with data available: any endoscopic ulcer: OR 0·28 (99% CI 0·24–0·32) unavailable versus 0·27 (0·25–0·29) available; any symptomatic ulcer: 0·29 (0·23–0·36) versus 0·22 (0·18–0·26); bleeds: 0·62 (0·33–1·14) versus 0·34 (0·26–0·45); and all-cause mortality: 0·81 (0·60–1·10) versus 1·03 (0·64–1·65). PPIs were effective in prevention of endoscopic and symptomatic ulcers irrespective of the use of NSAIDs, although for endoscopic ulcers the proportional odds reduction appeared to be greater in those not taking NSAIDs at trial entry (appendix p 7).

For cimetidine, the proportional reduction in endoscopic ulcers in the prevention trials increased with dose studied (p_trend_<0·0001). For the other three drugs, we found no evidence that their benefits increased with increasing dose (appendix p 8).

**Figure 5:**
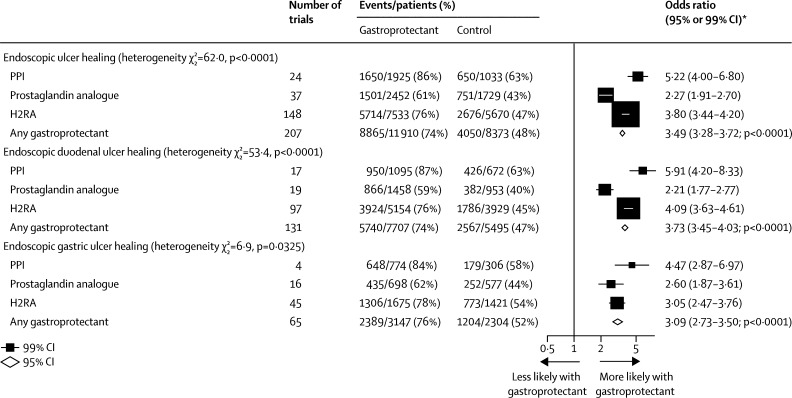
Healing trials: effects of gastroprotectants on ulcer healing We counted the number of patients who became ulcer free on endoscopy post treatment. For 204 trials for the endoscopic outcome, the total number of ulcers was not reported but the breakdown was; we assumed that the total number of ulcers was equal to gastric plus duodenal: 8861 of 11 844 events *vs* 4040 of 8312 events. PPI=proton-pump inhibitor. H2RA=histamine-2 receptor antagonist. *Summary odds ratios, indicated by diamonds, are presented with 95% CIs; all other odds ratios are presented with 99% CIs.

In the healing trials, we found that allocation to a gastroprotectant drug more than trebled the odds of endoscopic ulcer healing overall, compared with control (207 trials with median duration 0·9 months [IQR 0·9–1·4]: OR 3·49 [95% CI 3·28–3·72]; absolute benefit 261 [247–274] avoided per 1000 allocated treatment; p<0·0001; figure 5). Based on trials comparing a gastroprotectant with control, the order of effectiveness of gastroprotectant classes for duodenal and gastric ulcer healing was: PPIs as the most effective, followed by H2RAs and finally prostaglandin analogues (p_het_<0·0001 and p_het_=0·0325 respectively; figure 5). This order was consistent with the ordering implied by the results of direct comparisons of one gastroprotectant class versus another gastroprotectant class (appendix p 9).

Individual PPIs were all effective for endoscopic ulcer healing, but differed in their degree of effect (p_het_=0·0010; appendix p 10), with lansoprazole and pantoprazole seemingly exhibiting the largest effects and esomeprazole the smallest. We found no significant differences between individual H2RAs or prostaglandin analogues (appendix p 10).

As was observed in the prevention trials (appendix p 8), we found some evidence that the proportional increase in endoscopic ulcer healing for cimetidine was greater at higher doses (p_trend_=0·0478; appendix p 11).

**Figure 6:**
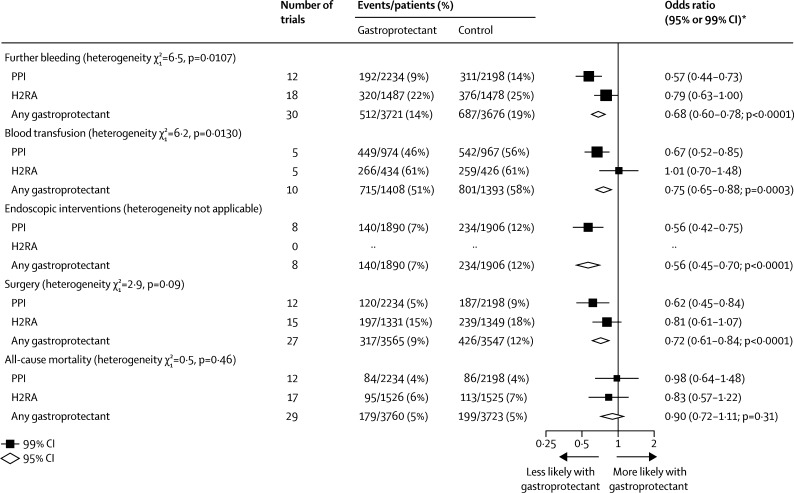
Acute upper gastrointestinal bleeding treatment trials: effects of gastroprotectants on further bleeding, need for blood transfusion, endoscopic interventions, surgery, and all-cause mortality PPI=proton-pump inhibitor. H2RA=histamine-2 receptor antagonist. *Summary odds ratios, indicated by diamonds, are presented with 95% CIs; all other odds ratios are presented with 99% CIs.

In the trials for treatment of acute upper gastrointestinal bleeding, we found that, overall, allocation to a gastroprotectant reduced the odds of further bleeding (30 trials with median duration of 3 days [IQR 2·00–4·00]: OR 0·68 [95% CI 0·60–0·78]; absolute benefit 49 [33–66] per 1000 allocated treatment; p<0·0001); blood transfusion (ten trials with median duration 2·5 days (2·00–3·75): OR 0·75 [0·65–0·88]; absolute benefit 67 [30–104] per 1000 allocated treatment; p<0·0001); further endoscopic intervention (eight trials with median duration 3 days [2·50–3·00]: OR 0·56 [0·45–0·70], absolute benefit 49 [30–68] per 1000 allocated treatment; p<0·0001); and the need for surgery (27 trials with median duration 3 days [2·00–3·50]: OR 0·72 [0·61–0·84]; absolute benefit 31 [17–45] per 1000 allocated treatment; p<0·0001), but had no significant effect on all-cause mortality (figure 6).

PPIs were more effective than were H2RAs in prevention of further bleeding (p_het_=0·0107) and the need for blood transfusion (p_het_=0·0130; figure 6). The limited direct information available from head-to-head comparisons supported this suggestion of superiority of PPIs over H2RAs for further bleeds and also endoscopic interventions (appendix p 12), but not the need for transfusion (although only one small head-to-head trial assessed this outcome). The benefits of PPIs (but not other gastroprotectant classes) were larger in trials in which the cause of bleeding was definitely attributed to peptic ulcer (appendix p 13) than in trials in which the cause was uncertain (appendix p 14). We found no consistent evidence that any single PPI or H2RA was more effective for the treatment of acute upper gastrointestinal bleeding than any other of the same class (appendix pp 15–19).

When assessing the potential for bias, we found that the proportional reduction in symptomatic ulcers and bleeds was smaller in the trials known to have used a secure method of allocation concealment (p_het_<0·0001 and p_het_=0·0002, respectively; appendix p 20); this was also the case, for bleeds only, in the trials that were double blind (p_het_=0·0006; appendix p 21). Among the analyses comparing a particular class of gastroprotectant versus control for various outcomes, no single trial dominated, and we found no consistent evidence of funnel plot asymmetry for any outcome in any of the three settings (prevention, healing, or acute upper gastrointestinal bleeding; appendix pp 22–24). The main results (arising from the direct head-to-head comparisons) were broadly similar to those derived from alternative network meta-analyses (appendix pp 25–33).

## Discussion

This meta-analysis of data from more than 1200 randomised trials of the main gastroprotectant drugs currently in use, which included more than 200 000 participants in total, showed that gastroprotectant therapies are effective for the prevention and treatment of peptic ulcer disease and its main complication, upper gastrointestinal bleeding, in a wide range of clinical circumstances. However, the absence of individual trials that recorded large numbers of ulcer complications, and the paucity of reliable information on drug safety, are limitations that constrain wider use of such treatments.

The results of trials of a gastroprotectant versus control, and of one gastroprotectant versus another gastroprotectant of a different class, were consistent, with PPIs being more effective than other classes of gastroprotectant drugs; this superiority was observed uniformly in the separate clinical circumstances in which these agents are used in peptic ulcer disease, namely prevention, healing, and treatment of acute upper gastrointestinal bleeding. In the context of prevention, most information was available from trials assessing the effects of gastroprotectant drugs on endoscopic ulcers, but the evidence of benefit was corroborated by similar findings in the smaller number of trials assessing their effects on symptomatic ulcers. The relative benefits of gastroprotectants for the prevention of endoscopic ulcers, symptomatic ulcers, and bleeding were large and of broadly similar magnitude irrespective of whether patients were taking an NSAID (including aspirin). This result is especially relevant in view of the recent evidence of large excess risks of bleeding in association with aspirin use in the elderly,^13^ and hence the potential for wider use of gastroprotectant therapy (eg, PPIs) in this demographic group. Although the absolute risk of developing endoscopic or symptomatic ulcers was actually higher in trials with no concomitant NSAID use than in trials with NSAID use (figure 4; appendix p 7), this is probably due to the fact that many patients classed as not using NSAIDs in these trials had a history of a recently healed ulcer (and hence would be likely to be at higher risk of recurrence of further such events). We found some evidence to suggest that PPIs reduced the risk of duodenal ulcers more than gastric ulcers; however, the relevance of this result is uncertain. There was also a suggestion that prostaglandin analogues are at least as effective as PPIs for the prevention of gastric ulcers; however, the difference between drug classes was not as marked as for duodenal ulcers, and prostaglandin analogues are less well tolerated than PPIs because they cause nausea, diarrhoea, and abdominal pain, as previously highlighted by a Cochrane review on misoprostol.^6^

This meta-analysis confirmed that gastroprotectant therapies prevent the clinical symptoms of ulcers and reduce the risk of upper gastrointestinal bleeding. The COGENT trial^12^ previously found that the addition of omeprazole to dual antiplatelet therapy (clopidogrel plus aspirin) reduced the risk of upper gastrointestinal bleeding.^14^, ^15^ Our meta-analysis suggests that this is a class-wide effect of PPIs and not restricted to omeprazole. A sensitivity analysis excluding the COGENT trial^12^ data found a similar, slightly greater, reduction in bleeding risk.

This meta-analysis does not provide much information about the effects of gastroprotectant regimens on peptic ulcer disease complications other than bleeding; although our analyses suggested that gastroprotectant regimens might reduce the risk of perforation associated with peptic ulcer disease, perforations were reported in only five of 115 prevention trials that reported at least one complication. Although this small number of reports might be partly due to the brevity of follow-up and the rarity of this complication, it is possible that reporting of the results was biased in favour of trials reporting favourable findings. A similar limitation applies to gastric outlet obstruction, which was reported in just four prevention trials. Therefore, further randomised trial evidence is needed to determine the effects of gastroprotectant regimens on such complications. This meta-analysis showed that gastroprotectants effectively reduced the risk of further bleeding, the need for blood transfusion, and the need for further endoscopic intervention or surgery in upper gastrointestinal bleeding secondary to peptic ulcer disease. An exploratory analysis showed that gastroprotectants were less effective in acute bleeding trials that recruited patients with upper gastrointestinal bleeding of any cause and initiated treatment prior to endoscopic diagnosis. This result is qualitatively consistent with a previous Cochrane review^16^ that found no evidence that PPI treatment initiated before endoscopy for upper gastrointestinal bleeding significantly reduced mortality, rebleeding, or the need for surgery.

The major strengths of this meta-analysis are the large number of events overall, which allowed precise quantitative estimates of treatment effects, together with the diversity of inclusion criteria among the large number of trials, which allowed study of a wide range of patients at risk of peptic ulcer disease. However, the study has a number of limitations. By far the most important of these is the absence of large placebo-controlled randomised trials that individually recorded substantial numbers of upper gastrointestinal complications: almost all of the available trials were short term and included small numbers of bleeds, and furthermore many did not publish all relevant outcomes. Additionally, the decision to report particular findings in any given trial might have been determined post hoc by their statistical significance alone, which could inflate estimates of efficacy. This possibility is reinforced by the implausibly large effect sizes observed for some of the treatments and outcomes in our meta-analysis. Even though analysis of funnel plots in this meta-analysis did not provide consistent evidence of publication bias, such plots are known to be insensitive, and it is possible that small study bias^17^, ^18^ might have led to overestimation of the magnitude of the relative and absolute benefits.

Second, although the results of trials comparing a gastroprotectant versus control and of trials comparing different classes of gastroprotectant yielded broadly consistent results on the relative effectiveness of PPIs, H2RAs, and prostaglandin analogues, and our exploratory network meta-analytic findings were generally (although not always) compatible with our primary effect estimates from trials of a gastroprotectant versus control, in the absence of individual participant data we were unable to check the assumptions underlying network meta-analysis (hence the decision to reserve the latter method for an exploratory analysis). Therefore, our estimates of the relative efficacy of different gastroprotectants might be unreliable. Additionally, the absence of individual participant data also meant that we were unable to explore potential modifiers of treatment effects or to investigate whether treatment effects varied over time.

Third, we were unable to explore the role of *H pylori* in this meta-analysis because of the inconsistency of recording of participant *H pylori* status; notably, many of the included trials pre-date the discovery of this pathogen. Similarly, although we assessed the effect of concomitant NSAID therapy on the effect of gastroprotectants in peptic ulcer disease, we could not assess whether other antithrombotic drugs (eg, vitamin K antagonists or novel oral anticoagulants) influenced the efficacy of gastroprotectant therapy owing to inconsistent information on such drug use.

Fourth, we excluded studies in the critical care setting because this clinical scenario is often not considered to represent typical peptic ulcer disease, so the results of this meta-analysis cannot be generalised to prevention of bleeding from stress-related mucosal disease in this context. However, a systematic review and meta-analysis of critically ill patients has also reported PPIs as being superior to H2RAs in this setting.^19^ Interventional radiology is also now commonly used after failed endoscopy, but this approach was introduced after the completion of most of the trials in our meta-analysis.

Finally, our literature search, although exhaustive, was completed several years ago and is now out of date. However, an updated search to Nov 24, 2017, did not yield any large trials that, had they been included, would have materially altered our conclusions.

Although this meta-analysis provides strong evidence of efficacy for the prevention of upper gastrointestinal complications, the use of gastroprotectant therapy to reduce bleeding risk among patients at high risk of cardiovascular disease is limited by concerns about drug safety, especially if gastroprotectant drugs need to be taken long term. In particular, in observational studies, PPIs have been shown to be associated with an increased risk of myocardial infarction,^20^, ^21^ bone fractures,^22^, ^23^ hypomagnesaemia,^24^, ^25^ food poisoning and bacterial gut infections,^26^ dementia,^27^ and chronic kidney disease.^28^, ^29^ Despite the fact that the COGENT trial^12^ reported no apparent cardiovascular hazard of omeprazole, concerns about the cardiovascular safety of PPIs have persisted. In 2018, results are expected from a sub-study of the COMPASS trial (NCT01776424) comparing around 3 years of pantoprazole versus placebo in 17 000 patients with stable cardiovascular disease who were treated with aspirin, rivaroxaban, or both drugs.^30^ Not only will this trial provide much needed information about long-term drug safety, but it will also help to determine the magnitude of any beneficial effects of a PPI more reliably than was possible in this meta-analysis.

In conclusion, this meta-analysis of randomised trials summarises the effects of gastroprotectant treatments on a range of outcomes in different treatment settings. PPIs generally appeared to be the most effective class of gastroprotectant for the management of peptic ulcer disease. Gastroprotectant drugs, many of which are available cheaply in generic form, are a potential strategy for reducing the global burden of peptic ulcer disease and, indirectly, might also have a beneficial effect on cardiovascular disease by allowing wider use of more potent antithrombotic regimens in high-risk patients.
