# Supplementary material for: Effects of gastroprotectant drugs for the prevention and treatment of peptic ulcer disease and its complications: a meta-analysis of randomised trials
Source: Lancet Gastroenterol Hepatol. 2018 Feb 21;3(4):231–41. doi: 10.1016/S2468-1253(18)30037-2 (PMC5842491; doi:10.1016/S2468-1253(18)30037-2)
Supplement: Supplementary appendix [file mmc1.pdf]

THE LANCET
Gastroenterology & Hepatology

Supplementary appendix

This appendix formed part of the original submission and has been peer reviewed. We post it as supplied by the authors.

Supplement to: Scally B, Emberson JR, Spata E, et al. Effects of gastroprotectant drugs for the prevention and treatment of peptic ulcer disease and its complications: a meta-analysis of randomised trials. *Lancet Gastroenterol Hepatol* 2018; published online Feb 20. http://dx.doi.org/10.1016/S2468-1253(18)30037-2.

Effects of gastroprotectants for the prevention and treatment of peptic ulcer disease and its complications: meta-analysis of randomised trials

Supplementary appendix

Pg

Box: Definition of study outcomes used in the meta-analysis **2**

Webfigures 1-6: Prevention Trials

Comparison of gastroprotectants on any endoscopic ulcer 3
Effects of gastroprotectants on any endoscopic ulcer 4
Effects of individual gastroprotectant drugs on any symptomatic ulcer 5
Effects of gastroprotectants on bleeds 6
Effects of proton pump inhibitors on endoscopic and symptomatic ulcers, bleeds and all-cause mortality, subdivided by use of NSAIDs 7
Effects of esomeprazole, omeprazole, cimetidine and ranitidine on any endoscopic ulcer, by dose studied 8

Webfigures 7-9: Healing trials

Comparison of gastroprotectants on endoscopic ulcer healing 9
Effects of individual gastroprotectant drugs on endoscopic ulcer healing 10
Effects of esomeprazole, omeprazole, cimetidine and ranitidine on endoscopic ulcers, by dose studied 11

Webfigures 10-12, 13a-e: Acute UGI bleeding trials

Comparison of gastroprotectants on further bleeding, need for blood transfusion, endoscopic intervention, surgery and all-cause mortality 12

Effects of gastroprotectants on further bleeding, need for blood transfusion, endoscopic intervention, surgery and all-cause mortality (acute UGI bleeding treatment trials of peptic ulcer) 13

Effects of gastroprotectants on further bleeding, need for blood transfusion, endoscopic intervention, surgery and all-cause mortality (acute UGI bleeding treatment trials of uncertain aetiology) 14

Effects of individual gastroprotectant drugs on: 15-19

a) further bleeding
b) blood transfusion
c) endoscopic interventions
d) surgery
e) all-cause mortality

Webfigures 14a-b: Prevention trials – effects on main outcomes by method of allocation concealment and method of blinding of investigators and patients **20-21**

Webfigures 15a-c: 'Funnel' plots for the main outcomes for each drug class in the prevention (15a), healing (15b) and acute UGI bleeding (15c) trials **22-24**

Webfigures 16a-i: Direct comparisons versus 'network' meta-analytic approach **25-33**

a) Prevention trials: effects on any endoscopic ulcer
b) Prevention trials: effects on any symptomatic ulcer
c) Prevention trials: effects on bleeds
d) Prevention trials: effects on all-cause mortality
e) Healing trials: effects on endoscopic ulcer healing
f) Acute UGI bleeding trials: effects on further bleeding
g) Acute UGI bleeding trials: effects on blood transfusion
h) Acute UGI bleeding trials: effects on surgery
i) Acute UGI bleeding trials: effects on all-cause mortality

Electronic Search Strategy **34-36**

Webtables 1-7: Full list of eligible trials, with references **37-91**

Box. Definitions of study outcomes used in the meta-analysis

Trial population	Outcome	Summary of trialists' definitions
**Prevention**	Endoscopic ulcers	Lesions ≥5mm (or ≥3mm in older studies).<br>Perception of 'depth' within lesion at endoscopy.
	Symptomatic ulcers	Any subjective clinical symptoms that a clinician may associate with peptic ulcer disease. Commonly described symptoms were dyspepsia or epigastric pain.
	Bleeding	Based on a range of clinical, biochemical and endoscopic parameters.
	Perforation	Based on clinical assessment, usually including imaging with erect chest x-ray.
	Obstruction	Based on clinical assessment.
	All-cause mortality	Death from any cause
**Healing**	Endoscopic ulcer healing	Endoscopic evidence of complete healing. Changes of scarring and mucosal regeneration were accepted as evidence of healing.
	All-cause mortality	Death from any cause
**Acute UGI bleeding treatment**	Further bleeding	Failure to establish initial haemostasis or any episode of upper gastrointestinal bleeding that occurred after initial haemostasis was established
	Blood transfusion	Any requirement for patients to receive red blood cell transfusion
	Endoscopic interventions	Any further endoscopic interventions that were performed after initial haemostasis was established
	Surgery	Any surgical interventions that were performed after initial haemostasis was established
	All-cause mortality	Death from any cause

Webfigure 1: Prevention trials − comparison of gastroprotectants on any endoscopic ulcer

	No.	Events/Patients (%) Allocated GPT 1	Allocated GPT 2	Odds Ratio (95 or 99% CI)
**PPI vs H2RA**				
Duodenal	8	162/1469 (11.0%)	186/1029 (18.1%)	0.40 (0.29 − 0.56)
Gastric	5	15/645 (2.3%)	46/497 (9.3%)	0.32 (0.15 − 0.66)
Any	10	179/1746 (10.3%)	233/1170 (19.9%)	0.39 (0.29 − 0.52)
**H2RA vs PA**				
Duodenal	6	49/562 (8.7%)	87/572 (15.2%)	0.45 (0.26 − 0.78)
Gastric	4	22/390 (5.6%)	8/408 (2.0%)	3.17 (1.11 − 9.07)
Any	8	70/659 (10.6%)	95/665 (14.3%)	0.68 (0.42 − 1.10)
**PPI vs PA**				
Duodenal	2	7/543 (1.3%)	35/430 (8.1%)	0.23 (0.10 − 0.56)
Gastric	2	86/543 (15.8%)	42/430 (9.8%)	1.63 (0.97 − 2.75)
Any	3	94/800 (11.8%)	80/688 (11.6%)	0.94 (0.60 − 1.47)

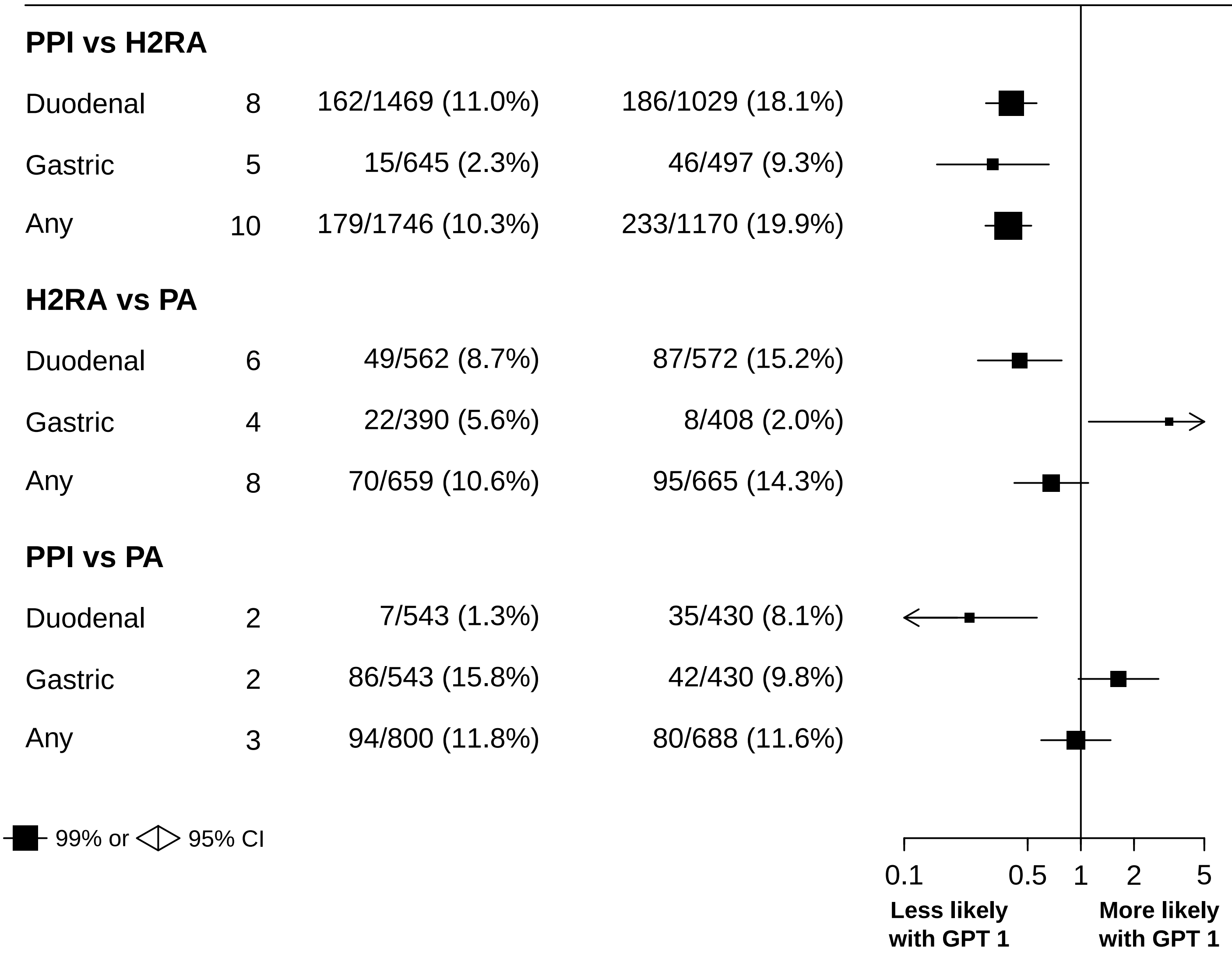

PPI=Proton pump inhibitors, PA=Prostaglandin analogues, H2RA=Histamine−2 receptor antagonists

There was one trial comparing PA + H2RA versus H2RA (misoprostol + ranitidine vs ranitidine) with 3/25 vs 14/28 endoscopic ulcers.

Webfigure 2: Prevention trials − effects of gastroprotectants on any endoscopic ulcer

	No.	Events/Patients (%) Allocated GPT	Allocated control	Odds Ratio (95 or 99% CI)
**Proton Pump Inhibitor** (Heterogeneity χ^2_3 =2.7, p=0.44)				
Esomeprazole	11	111/4210 (2.6%)	408/2908 (14.0%)	0.19 (0.15 − 0.25)
Rabeprazole	0	−	−	
Omeprazole	11	109/1781 (6.1%)	347/1665 (20.8%)	0.21 (0.16 − 0.28)
Lansoprazole	6	87/480 (18.1%)	175/305 (57.4%)	0.17 (0.11 − 0.25)
Pantoprazole	1	7/70 (10.0%)	7/34 (20.6%)	0.41 (0.07 − 2.35)
Dexlansoprazole	0	−	−	
Any PPI	29	314/6541 (4.8%)	937/4912 (19.1%)	**0.20 (0.17 − 0.22)** p<0.0001
**Prostaglandin Analogues** (Heterogeneity χ^2_1 =0.6, p=0.43)				
Misoprostol	21	228/3358 (6.8%)	434/2498 (17.4%)	0.26 (0.20 − 0.32)
Enprostil	1	0/69 (0.0%)	1/34 (2.9%)	
Rebamipide	0	−	−	
Rioprostil	0	−	−	
Arbaprostil	0	−	−	
Other PAs	0	−	−	
Any PA	22	228/3427 (6.7%)	435/2532 (17.2%)	**0.26 (0.21 − 0.31)** p<0.0001
**Histamine−2 Receptor Antagonists** (Heterogeneity χ^2_5 =12.5, p=0.0286)				
Cimetidine	52	530/2311 (22.9%)	819/1681 (48.7%)	0.30 (0.24 − 0.36)
Famotidine	11	274/2374 (11.5%)	408/1502 (27.2%)	0.36 (0.28 − 0.46)
Ranitidine	35	272/1824 (14.9%)	618/1800 (34.3%)	0.30 (0.24 − 0.38)
Nizatidine	4	156/826 (18.9%)	259/717 (36.1%)	0.41 (0.29 − 0.56)
Roxatidine	2	91/364 (25.0%)	166/361 (46.0%)	0.40 (0.27 − 0.60)
Oxmetidine	0	−	−	
Other H2RA	2	24/375 (6.4%)	67/229 (29.3%)	0.19 (0.09 − 0.41)
Any H2RA	105	1347/8074 (16.7%)	2298/6187 (37.1%)	**0.32 (0.29 − 0.34)** p<0.0001
**Any gastroprotectant drug**				
Any GPT	152	1889/18042 (10.5%)	3502/13255 (26.4%)	**0.27 (0.25 − 0.29)** p<0.0001

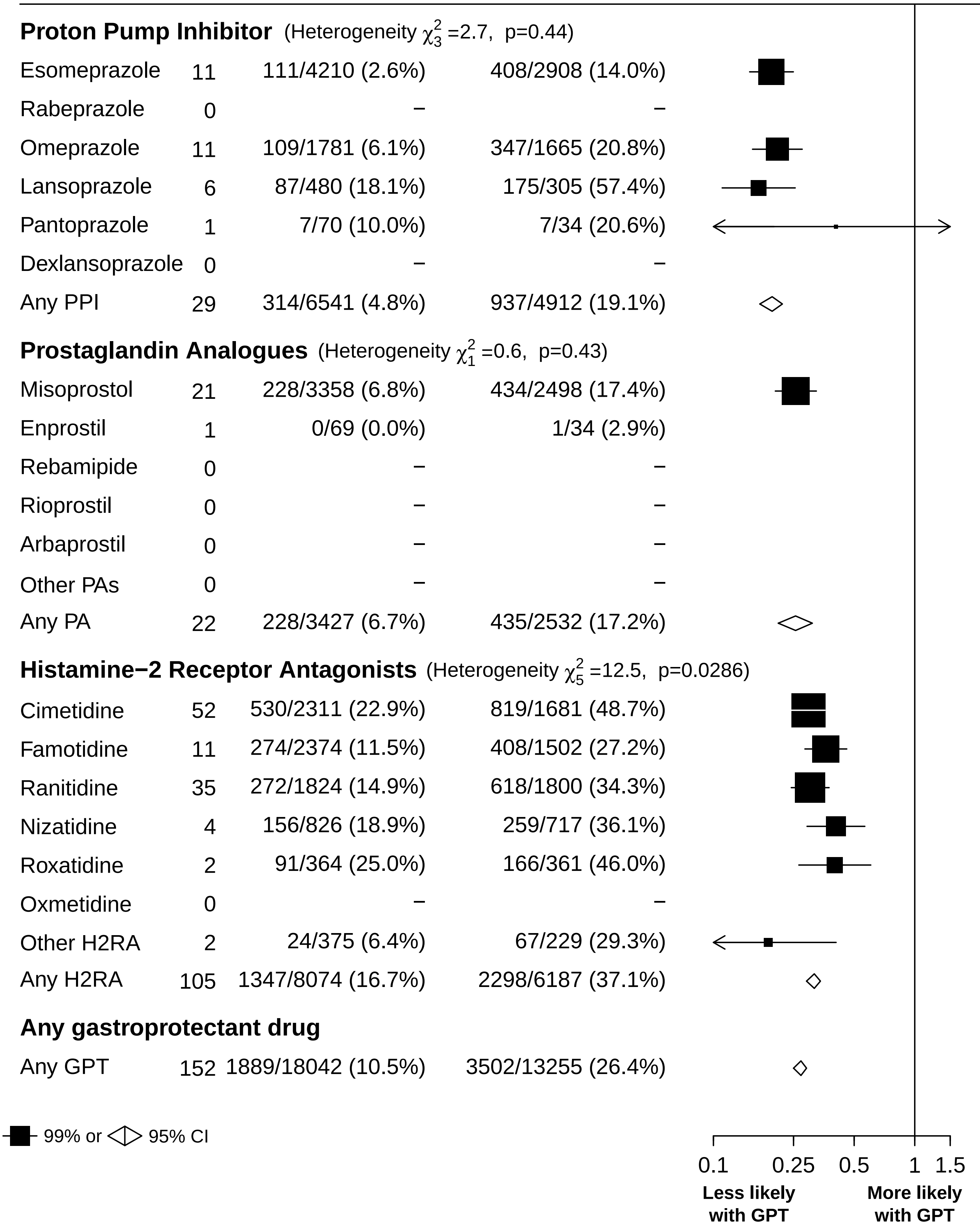

PPI=Proton pump inhibitors, PA=Prostaglandin analogues, H2RA=Histamine−2 receptor antagonists, GPT=Gastroprotectant drug

Webfigure 3: Prevention trials − effects of individual gastroprotectant drugs on any symptomatic ulcer

	No.	Events/Patients (%) Allocated GPT	Events/Patients (%) Allocated control	Odds Ratio (95 or 99% CI)
**Proton Pump Inhibitor** (Heterogeneity χ^2_2 =1.1, p=0.56)				
Esomeprazole	3	5/415 (1.2%)	39/411 (9.5%)	0.18 (0.08 − 0.43)
Rabeprazole	0	−	−	
Omeprazole	4	22/2148 (1.0%)	108/2155 (5.0%)	0.13 (0.07 − 0.23)
Lansoprazole	1	1/22 (4.5%)	5/21 (23.8%)	0.21 (0.02 − 2.87)
Pantoprazole	0	−	−	
Dexlansoprazole	0	−	−	
Any PPI	8	28/2585 (1.1%)	152/2587 (5.9%)	**0.15 (0.10 − 0.21)** p<0.0001
**Prostaglandin Analogues** (Heterogeneity χ^2_1 =1.9, p=0.17)				
Misoprostol	3	14/4588 (0.3%)	35/4639 (0.8%)	0.43 (0.20 − 0.94)
Enprostil	0	−	−	
Rebamipide	1	0/31 (0.0%)	6/34 (17.6%)	0.13 (0.01 − 1.62)
Rioprostil	0	−	−	
Arbaprostil	0	−	−	
Other PAs	0	−	−	
Any PA	4	14/4619 (0.3%)	41/4673 (0.9%)	**0.38 (0.21 − 0.67)** p=0.0004
**Histamine−2 Receptor Antagonists** (Heterogeneity χ^2_3 =8.1, p=0.0447)				
Cimetidine	28	401/1879 (21.3%)	724/1483 (48.8%)	0.29 (0.24 − 0.35)
Famotidine	1	7/115 (6.1%)	11/123 (8.9%)	0.67 (0.17 − 2.65)
Ranitidine	10	50/373 (13.4%)	163/386 (42.2%)	0.21 (0.13 − 0.32)
Nizatidine	1	23/286 (8.0%)	99/295 (33.6%)	0.22 (0.12 − 0.37)
Roxatidine	0	−	−	
Oxmetidine	0	−	−	
Other H2RA	0	−	−	
Any H2RA	39	481/2653 (18.1%)	943/2184 (43.2%)	**0.27 (0.23 − 0.31)** p<0.0001
**Any gastroprotectant drug**				
Any GPT	51	523/9857 (5.3%)	1136/9444 (12.0%)	**0.25 (0.22 − 0.29)** p<0.0001

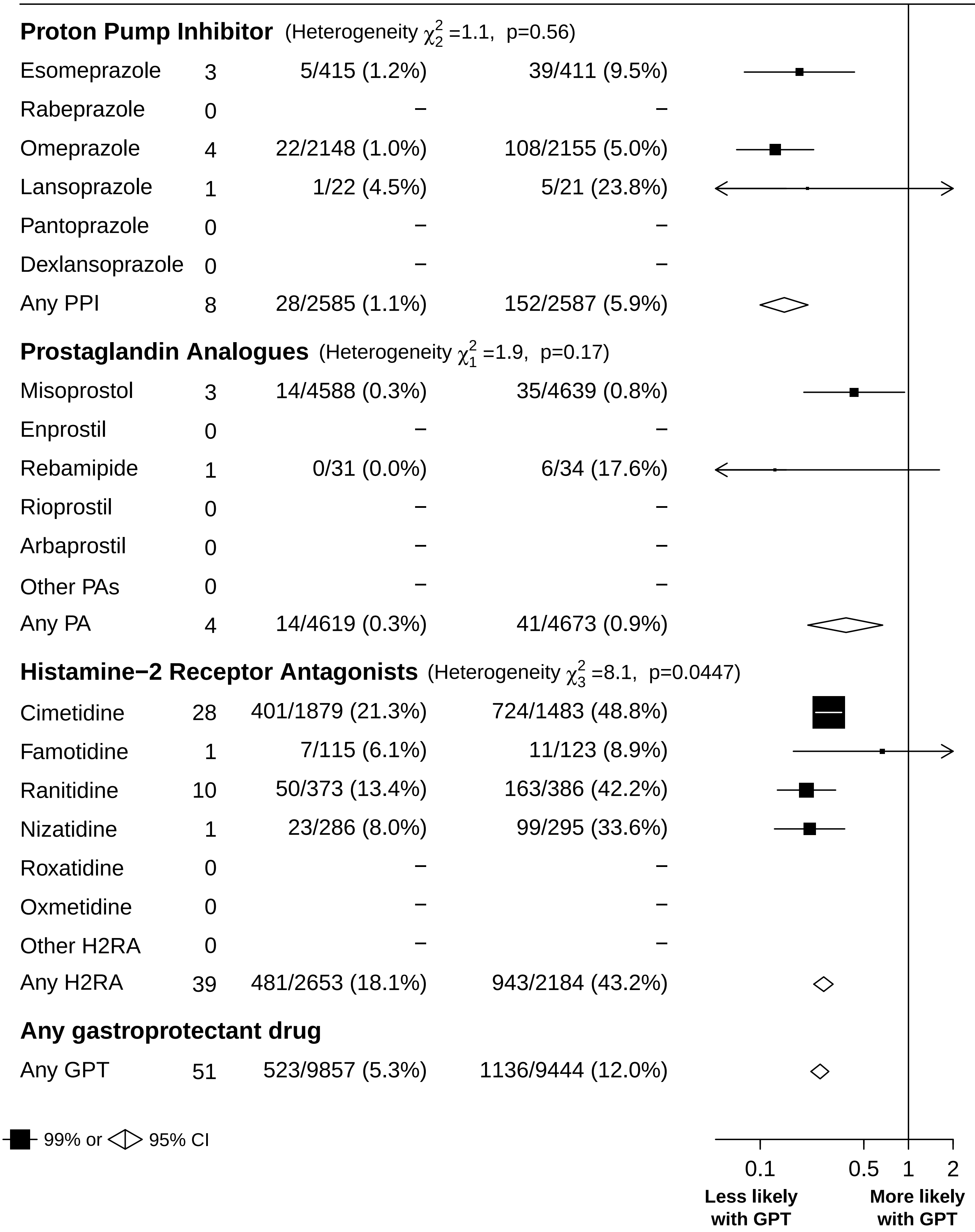

PPI=Proton pump inhibitors, PA=Prostaglandin analogues, H2RA=Histamine−2 receptor antagonists, GPT=Gastroprotectant drug

Webfigure 4: Prevention trials − effects of gastroprotectants on bleeds

	No.	Events/Patients (%) Allocated GPT	Events/Patients (%) Allocated control	Odds Ratio (95 or 99% CI)
**Proton Pump Inhibitor** (Heterogeneity χ^2_3 =2.5, p=0.48)				
Esomeprazole	7	3/2885 (0.1%)	25/2070 (1.2%)	0.18 (0.06 − 0.51)
Rabeprazole	0	−	−	
Omeprazole	7	9/2502 (0.4%)	37/2501 (1.5%)	0.29 (0.13 − 0.65)
Lansoprazole	3	2/353 (0.6%)	12/216 (5.6%)	0.20 (0.04 − 0.97)
Pantoprazole	2	0/170 (0.0%)	16/170 (9.4%)	0.12 (0.03 − 0.53)
Dexlansoprazole	0	−	−	
Any PPI	18	14/5910 (0.2%)	88/4937 (1.8%)	**0.21 (0.14 − 0.32)** p<0.0001
**Prostaglandin Analogues** (Heterogeneity χ^2_1 =1.5, p=0.22)				
Misoprostol	2	33/4435 (0.7%)	52/4475 (1.2%)	0.64 (0.36 − 1.15)
Enprostil	0	−	−	
Rebamipide	1	0/29 (0.0%)	1/14 (7.1%)	
Rioprostil	0	−	−	
Arbaprostil	0	−	−	
Other PAs	0	−	−	
Any PA	3	33/4464 (0.7%)	53/4489 (1.2%)	**0.63 (0.40 − 0.98)** p=0.0306
**Histamine−2 Receptor Antagonists** (Heterogeneity χ^2_4 =18.1, p=0.0012)				
Cimetidine	10	25/481 (5.2%)	21/442 (4.8%)	1.20 (0.51 − 2.79)
Famotidine	5	7/1661 (0.4%)	7/985 (0.7%)	0.79 (0.16 − 3.83)
Ranitidine	11	11/866 (1.3%)	48/875 (5.5%)	0.27 (0.13 − 0.55)
Nizatidine	1	0/248 (0.0%)	1/248 (0.4%)	
Roxatidine	0	−	−	
Oxmetidine	0	−	−	
Other H2RA	2	0/375 (0.0%)	7/229 (3.1%)	0.11 (0.01 − 1.15)
Any H2RA	29	43/3631 (1.2%)	84/2779 (3.0%)	**0.49 (0.33 − 0.71)** p=0.0001
**Any gastroprotectant drug**				
Any GPT	49	90/14139 (0.6%)	221/12130 (1.8%)	**0.40 (0.32 − 0.50)** p<0.0001

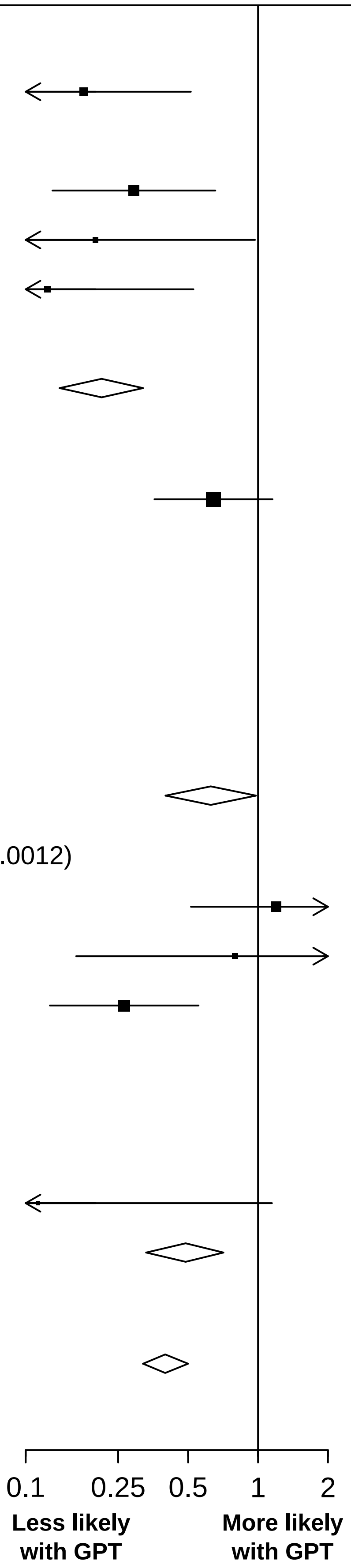

99% or 95% CI

PPI=Proton pump inhibitors, PA=Prostaglandin analogues, H2RA=Histamine−2 receptor antagonists, GPT=Gastroprotectant drug

Webfigure 5: Prevention trials − effects of proton pump inhibitors on endoscopic and symptomatic ulcers, bleeds and all−cause mortality, subdivided by use of NSAIDs

tNSAID/ COXIB/ aspirin use	No.	Events/Patients (%) Allocated PPI	Allocated control	Odds Ratio (95 or 99% CI)
**Any endoscopic ulcer** (Heterogeneity χ^2_1 =12.8, p=0.0003)				
NSAID use	18	249/5797 (4.3%)	653/4215 (15.5%)	0.23 (0.19 − 0.27)
No NSAID use	8	55/474 (11.6%)	225/446 (50.4%)	0.12 (0.08 − 0.18)
Total	26	304/6271 (4.8%)	878/4661 (18.8%)	**0.20 (0.18 − 0.23)** p<0.0001
**Any symptomatic ulcer** (Heterogeneity χ^2_1 =2.1, p=0.14)				
NSAID use	4	7/2221 (0.3%)	51/2215 (2.3%)	0.19 (0.09 − 0.40)
No NSAID use	3	20/207 (9.7%)	94/211 (44.5%)	0.11 (0.06 − 0.22)
Total	7	27/2428 (1.1%)	145/2426 (6.0%)	**0.14 (0.10 − 0.21)** p<0.0001
**Bleeds** (Heterogeneity χ^2_1 =0.4, p=0.54)				
NSAID use	14	14/5534 (0.3%)	80/4556 (1.8%)	0.22 (0.13 − 0.39)
No NSAID use	3	0/219 (0.0%)	6/220 (2.7%)	0.13 (0.01 − 1.53)
Total	17	14/5753 (0.2%)	86/4776 (1.8%)	**0.21 (0.14 − 0.33)** p<0.0001
**All−cause mortality** (Heterogeneity χ^2_1 =0.2, p=0.62)				
NSAID use	11	20/5872 (0.3%)	14/4364 (0.3%)	1.06 (0.40 − 2.80)
No NSAID use	2	2/135 (1.5%)	1/135 (0.7%)	1.94 (0.05 − 74.68)
Total	13	22/6007 (0.4%)	15/4499 (0.3%)	**1.12 (0.55 − 2.29)** p=0.74

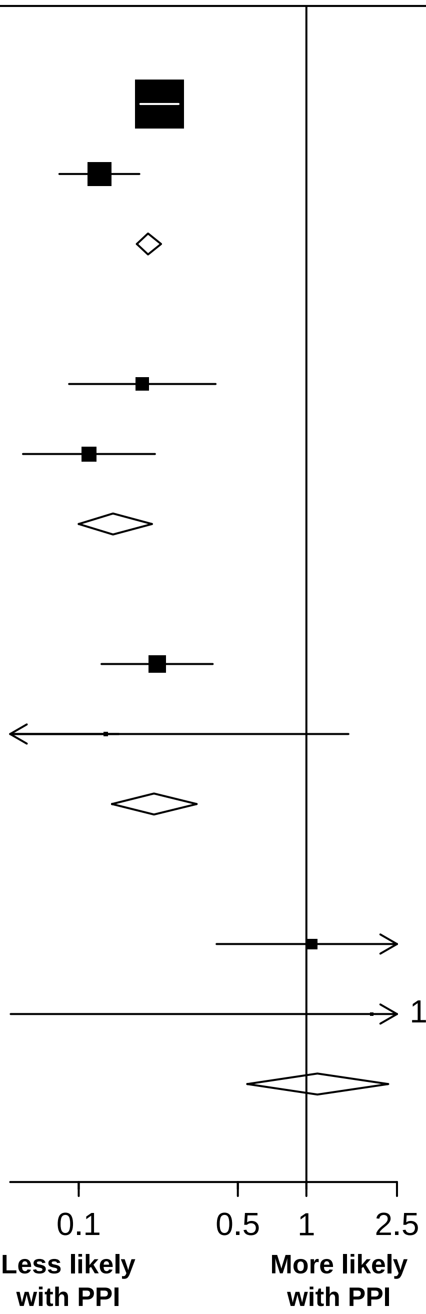

■ 99% or ◇ 95% CI

PPI: Proton pump inhibitor

Trial categorization:
NSAID use: greater than or equal to 80% of patients took a tNSAID, COXIB or aspirin.
No NSAID use: less than 80% of patients took a tNSAID, COXIB or aspirin.

The results for trials in which information about NSAID use at baseline was unavailable were similar to those with data available: any endoscopic ulcer RR=0.12 (0.06−0.25) unavailable vs 0.20 (0.18−0.23) available; any symptomatic ulcer RR=0.14 (0.03−1.80) unavailable vs 0.14 (0.10−0.21) available; bleeds no data for unavailable; all−cause mortality RR=0.87 (0.28−2.75) unavailable vs 1.12 (0.55−2.29) available.

Webfigure 6: Prevention trials − effects of esomeprazole, omeprazole, cimetidine and ranitidine on any endoscopic ulcer, by dose studied

	No.	Events/Patients (%) Allocated GPT	Events/Patients (%) Allocated control	Odds Ratio (95 or 99% CI)
**Esomeprazole*** (Trend test χ^2_1 =0.4, p=0.55)				
20 mg	9	53/2483 (2.1%)	284/2477 (11.5%)	0.22 (0.16 − 0.29)
40 mg	5	58/1727 (3.4%)	250/1709 (14.6%)	0.24 (0.17 − 0.33)
Total	11	111/4210 (2.6%)	408/2908 (14.0%)	**0.19 (0.16 − 0.24)** p<0.0001
**Omeprazole** (Trend test χ^2_1 =8.0, p=0.0047)				
10 mg	1	17/64 (26.6%)	44/66 (66.7%)	0.20 (0.08 − 0.53)
20 mg	7	57/672 (8.5%)	197/552 (35.7%)	0.15 (0.10 − 0.22)
40 mg	3	35/1045 (3.3%)	106/1047 (10.1%)	0.34 (0.21 − 0.54)
Total	11	109/1781 (6.1%)	347/1665 (20.8%)	**0.21 (0.17 − 0.26)** p<0.0001
**Cimetidine** (Trend test χ^2_1 =55.7, p<0.0001)				
200 mg	1	48/98 (49.0%)	39/103 (37.9%)	1.57 (0.72 − 3.40)
400 mg	30	339/1346 (25.2%)	516/1142 (45.2%)	0.39 (0.31 − 0.49)
600 mg	7	81/394 (20.6%)	115/223 (51.6%)	0.25 (0.16 − 0.41)
800 mg	14	54/382 (14.1%)	231/362 (63.8%)	0.11 (0.08 − 0.17)
1000 mg	2	3/37 (8.1%)	16/34 (47.1%)	0.11 (0.02 − 0.60)
1200 mg	1	5/54 (9.3%)	2/50 (4.0%)	2.29 (0.23 − 23.14)
Total	52	530/2311 (22.9%)	819/1681 (48.7%)	**0.30 (0.26 − 0.34)** p<0.0001
**Ranitidine** (Trend test χ^2_1 =0.4, p=0.52)				
150 mg	23	234/1164 (20.1%)	514/1153 (44.6%)	0.30 (0.23 − 0.38)
200** mg	2	4/64 (6.2%)	23/63 (36.5%)	0.15 (0.04 − 0.53)
300 mg	9	31/581 (5.3%)	73/569 (12.8%)	0.38 (0.22 − 0.67)
600 mg	1	3/15 (20.0%)	8/15 (53.3%)	0.25 (0.03 − 2.25)
Total	35	272/1824 (14.9%)	618/1800 (34.3%)	**0.30 (0.26 − 0.36)** p<0.0001

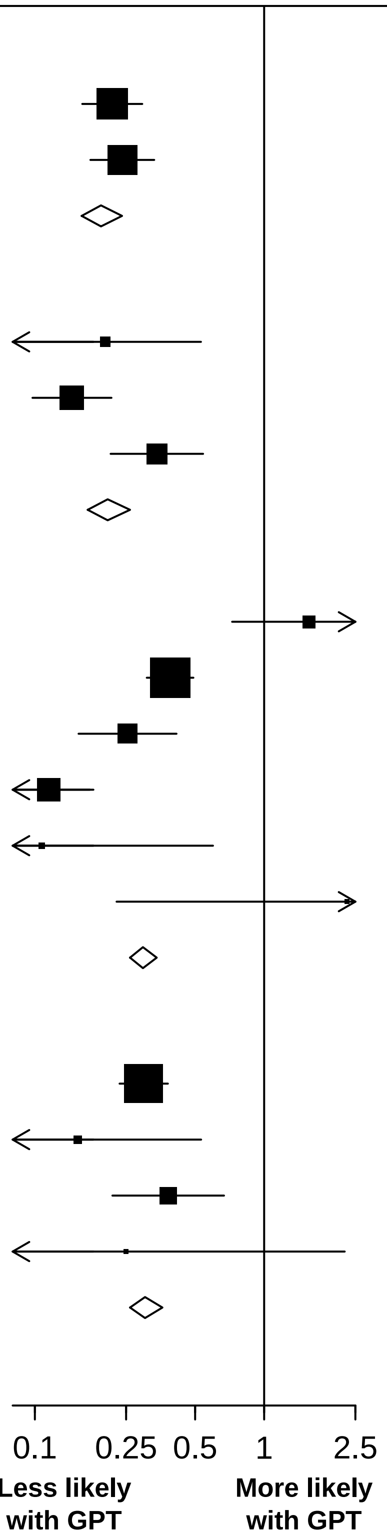

99% or 95% CI

*Some trials involve 20mg vs 40mg vs control, which happened to lead both dose−specific estimates being less extreme than the overall estimate.

**Penston (1993) trial was included in this dose group which reported a ranitidine dose of 225 mg.

Webfigure 7: Healing trials − comparison of gastroprotectants on endoscopic ulcer healing

	No.	Events/Patients (%) Allocated GPT 1	Events/Patients (%) Allocated GPT 2	Odds Ratio (95 or 99% CI)
**PPI vs H2RA**				
Duodenal	72	5573/6059 (92.0%)	4168/5078 (82.1%)	2.62 (2.24 − 3.06)
Gastric	33	2559/2972 (86.1%)	1534/2070 (74.1%)	2.54 (2.06 − 3.13)
Any	106	8214/9117 (90.1%)	5786/7245 (79.9%)	2.58 (2.28 − 2.92)
**H2RA vs PA**				
Duodenal	27	2018/2470 (81.7%)	2041/2909 (70.2%)	1.67 (1.39 − 1.99)
Gastric	10	327/402 (81.3%)	279/387 (72.1%)	1.81 (1.09 − 3.02)
Any	37	2345/2872 (81.7%)	2320/3296 (70.4%)	1.68 (1.42 − 1.99)
**PPI vs PA**				
Duodenal	1	133/146 (91.1%)	60/76 (78.9%)	2.90 (0.90 − 9.31)
Gastric	3	297/361 (82.3%)	181/233 (77.7%)	1.41 (0.79 − 2.50)
Any	4	457/538 (84.9%)	267/338 (79.0%)	1.57 (0.96 − 2.57)

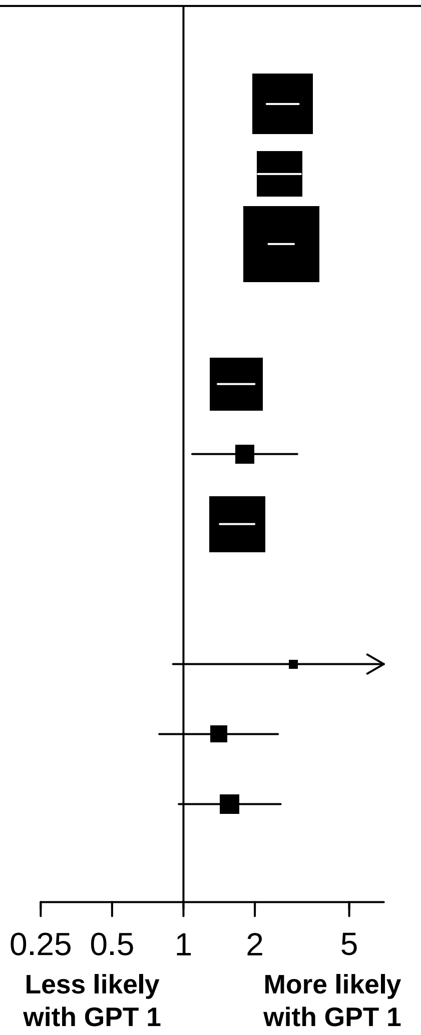

99% or 95% CI

PPI=Proton pump inhibitors, PA=Prostaglandin analogues, H2RA=Histamine−2 receptor antagonists

There was one trial comparing PA + H2RA versus H2RA with 76/82 vs 59/84 endoscopic ulcers.
Two additional trials compared PA + PPI versus PPI with a total of 36/130 vs 19/127 endoscopic ulcers.

Webfigure 8: Healing trials − effects of individual gastroprotectant drugs on endoscopic ulcer healing

	No.	Events/Patients (%) Allocated GPT	Events/Patients (%) Allocated control	Odds Ratio (95 or 99% CI)
**Proton Pump Inhibitor** (Heterogeneity χ^2_4 =18.4, p=0.0010)				
Esomeprazole	1	90/110 (81.8%)	71/111 (64.0%)	2.46 (1.08 − 5.59)
Rabeprazole	3	150/170 (88.2%)	58/102 (56.9%)	7.54 (3.27 − 17.35)
Omeprazole	14	757/919 (82.4%)	334/522 (64.0%)	4.18 (2.86 − 6.10)
Lansoprazole	5	609/682 (89.3%)	153/248 (61.7%)	8.97 (5.22 − 15.42)
Pantoprazole	1	44/44 (100.0%)	34/50 (68.0%)	9.41 (1.98 − 44.72)
Dexlansoprazole	0	−	−	
Any PPI	24	1650/1925 (85.7%)	650/1033 (62.9%)	**5.22 (4.26 − 6.39)** p<0.0001
**Prostaglandin Analogues** (Heterogeneity χ^2_5 =5.0, p=0.42)				
Misoprostol	10	584/954 (61.2%)	291/647 (45.0%)	1.99 (1.51 − 2.63)
Enprostil	5	186/266 (69.9%)	74/179 (41.3%)	3.10 (1.80 − 5.32)
Rebamipide	5	232/293 (79.2%)	188/285 (66.0%)	2.10 (1.24 − 3.57)
Rioprostil	3	69/124 (55.6%)	27/83 (32.5%)	2.90 (1.27 − 6.61)
Arbaprostil	14	423/796 (53.1%)	171/535 (32.0%)	2.37 (1.74 − 3.23)
Other PAs	1	7/19 (36.8%)	4/16 (25.0%)	1.71 (0.21 − 14.14)
Any PAs	37	1501/2452 (61.2%)	751/1729 (43.4%)	**2.27 (1.99 − 2.59)** p<0.0001
**Histamine−2 Receptor Antagonists** (Heterogeneity χ^2_5 =5.9, p=0.31)				
Cimetidine	85	2450/3324 (73.7%)	1088/2385 (45.6%)	3.58 (3.08 − 4.17)
Famotidine	6	585/736 (79.5%)	300/560 (53.6%)	3.38 (2.43 − 4.69)
Ranitidine	49	1651/2077 (79.5%)	919/1869 (49.2%)	4.25 (3.54 − 5.11)
Nizatidine	9	863/1190 (72.5%)	306/726 (42.1%)	3.75 (2.85 − 4.93)
Roxatidine	1	131/166 (78.9%)	73/163 (44.8%)	4.24 (2.30 − 7.80)
Oxmetidine	1	34/40 (85.0%)	15/36 (41.7%)	6.47 (1.69 − 24.76)
Other H2RA	0	−	−	
Any H2RA	148	5714/7533 (75.9%)	2676/5670 (47.2%)	**3.80 (3.52 − 4.10)** p<0.0001
**Any gastroprotectant drug**				
Any GPT	207	8865/11910 (74.4%)	4050/8373 (48.4%)	**3.49 (3.28 − 3.72)** p<0.0001

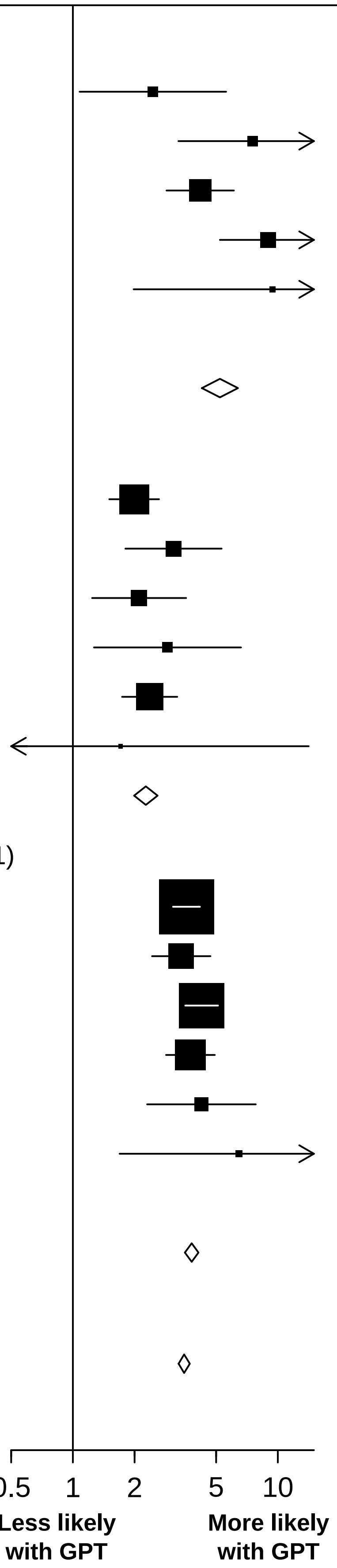

PPI=Proton pump inhibitors, PA=Prostaglandin analogues, H2RA=Histamine−2 receptor antagonists, GPT=Gastroprotectant drug

Webfigure 9: Healing trials − effects of esomeprazole, omeprazole, cimetidine and ranitidine on endoscopic ulcers, by dose studied

	No.	Events/Patients (%) Allocated GPT	Events/Patients (%) Allocated control	Odds Ratio (95 or 99% CI)
**Esomeprazole**				
20 mg	1	90/110 (81.8%)	71/111 (64.0%)	2.46 (1.08 − 5.59)
Total	1	90/110 (81.8%)	71/111 (64.0%)	**2.46 (1.30 − 4.64)** p=0.0029
**Omeprazole** (Trend test χ_1^2 =0.0, p=0.99)				
20 mg	11	472/574 (82.2%)	274/428 (64.0%)	3.89 (2.55 − 5.94)
40 mg	3	239/290 (82.4%)	95/177 (53.7%)	4.69 (2.59 − 8.47)
80 mg	1	46/55 (83.6%)	15/21 (71.4%)	2.14 (0.33 − 13.66)
Total	14	757/919 (82.4%)	334/522 (64.0%)	**4.18 (3.13 − 5.59)** p<0.0001
**Cimetidine** (Trend test χ_1^2 =3.9, p=0.0478)				
400 mg	3	235/366 (64.2%)	146/339 (43.1%)	2.34 (1.57 − 3.49)
800 mg	9	454/590 (76.9%)	279/579 (48.2%)	3.50 (2.53 − 4.84)
1000 mg	52	930/1243 (74.8%)	490/1161 (42.2%)	4.20 (3.36 − 5.25)
1200 mg	22	453/642 (70.6%)	315/648 (48.6%)	2.59 (1.91 − 3.53)
1600 mg	5	316/401 (78.8%)	159/365 (43.6%)	4.43 (2.99 − 6.58)
2000 mg	1	45/64 (70.3%)	13/46 (28.3%)	5.32 (1.83 − 15.47)
Unknown	1	17/18 (94.4%)	10/17 (58.8%)	7.12 (0.67 − 75.37)
Total	85	2450/3324 (73.7%)	1088/2385 (45.6%)	**3.58 (3.19 − 4.02)** p<0.0001
**Ranitidine** (Trend test χ_1^2 =6.8, p=0.0094)				
200 mg	5	67/77 (87.0%)	29/74 (39.2%)	7.99 (3.07 − 20.81)
300 mg	40	1425/1827 (78.0%)	745/1626 (45.8%)	4.22 (3.49 − 5.11)
400 mg	1	17/25 (68.0%)	8/23 (34.8%)	3.68 (0.72 − 18.94)
600 mg	3	142/148 (95.9%)	137/146 (93.8%)	1.45 (0.31 − 6.79)
Total	49	1651/2077 (79.5%)	919/1869 (49.2%)	**4.25 (3.69 − 4.89)** p<0.0001

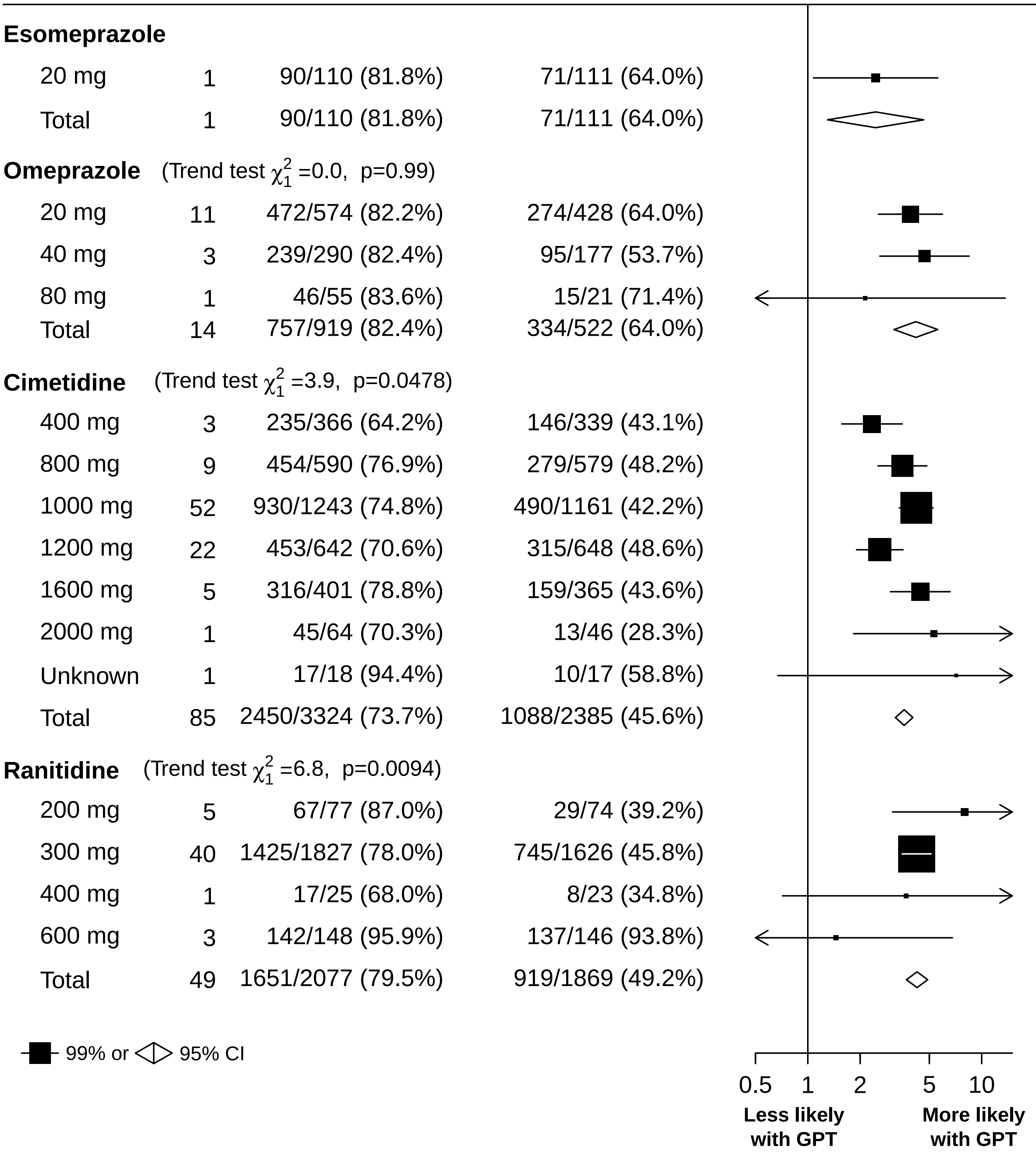

Webfigure 10: Acute UGI bleeding treatment trials − comparison of gastroprotectants on further bleeding, need for blood transfusion, endoscopic intervention, surgery and all−cause mortality

	No.	Events/Patients (%) Allocated GPT 1	Events/Patients (%) Allocated GPT 2	Odds Ratio (95 or 99% CI)
**PPI vs H2RA**				
Further bleeding	16	101/1737 (5.8%)	162/1658 (9.8%)	0.51 (0.36 − 0.72)
Transfusion	1	115/267 (43.1%)	112/252 (44.4%)	0.95 (0.59 − 1.52)
Endoscopic	9	68/1157 (5.9%)	94/1093 (8.6%)	0.60 (0.38 − 0.93)
Surgery	10	37/1395 (2.7%)	52/1319 (3.9%)	0.67 (0.37 − 1.20)
Mortality	11	19/1520 (1.2%)	23/1446 (1.6%)	0.74 (0.31 − 1.76)

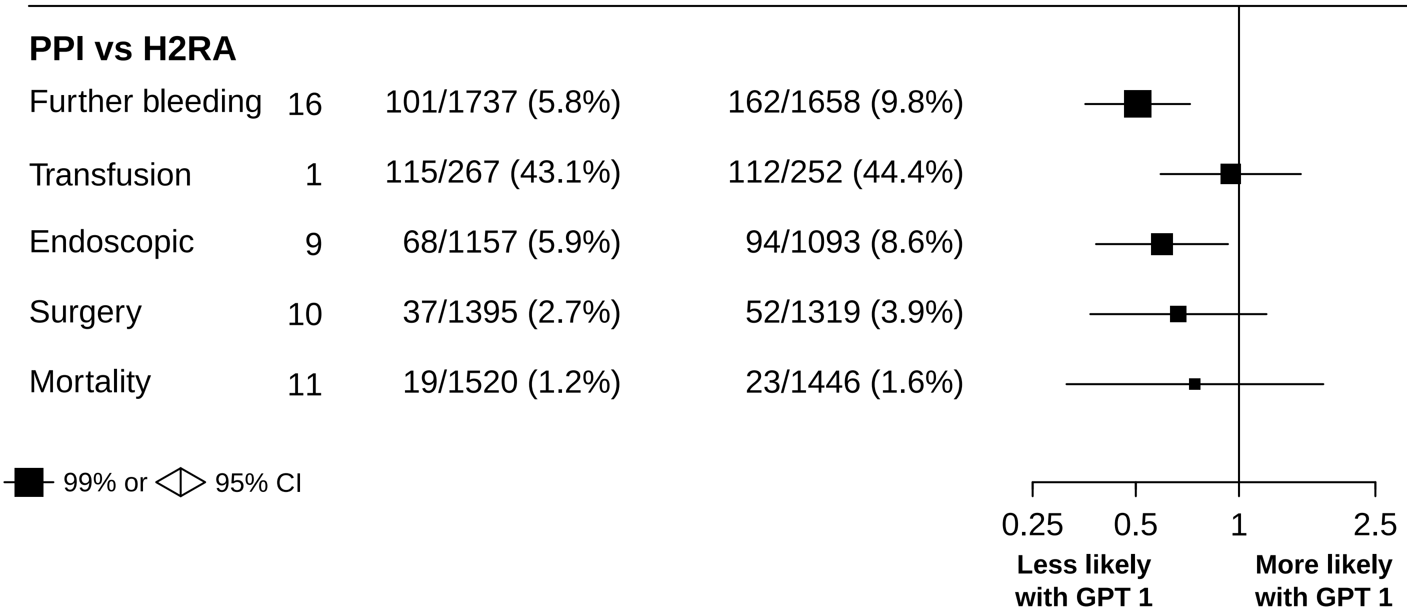

PPI=Proton pump inhibitors, H2RA=Histamine−2 receptor antagonists

One additional trial compared PA + PPI versus PPI, in which there were 7/56 vs 8/67 rebleeds, 1/56 vs 2/67 surgical procedures and 2/56 vs 1/67 deaths.

Webfigure 11: Effects of gastroprotectants on further bleeding, need for blood transfusion, endoscopic intervention, surgery and all−cause mortality (acute UGI bleeding treatment trials of peptic ulcer)

	No.	Events/Patients (%) Allocated GPT	Events/Patients (%) Allocated control	Odds Ratio (95 or 99% CI)
**Further bleeding** (Heterogeneity χ^2_1 =12.0, p=0.0005)				
PPI	9	89/1235 (7.2%)	194/1207 (16.1%)	0.42 (0.30 − 0.59)
H2RA	7	148/681 (21.7%)	180/692 (26.0%)	0.78 (0.56 − 1.09)
Any GPT	16	237/1916 (12.4%)	374/1899 (19.7%)	**0.57 (0.48 − 0.69)** p<0.0001
**Blood transfusion** (Heterogeneity χ^2_1 =4.8, p=0.0293)				
PPI	3	84/294 (28.6%)	180/295 (61.0%)	0.26 (0.17 − 0.40)
H2RA	3	77/117 (65.8%)	87/111 (78.4%)	0.54 (0.24 − 1.21)
Any GPT	6	161/411 (39.2%)	267/406 (65.8%)	**0.31 (0.23 − 0.42)** p<0.0001
**Endoscopic interventions**				
PPI	5	55/891 (6.2%)	117/915 (12.8%)	0.46 (0.30 − 0.71)
H2RA	0	−	−	
Any GPT	5	55/891 (6.2%)	117/915 (12.8%)	**0.46 (0.33 − 0.64)** p<0.0001
**Surgery** (Heterogeneity χ^2_1 =7.6, p=0.0059)				
PPI	9	52/1235 (4.2%)	114/1207 (9.4%)	0.44 (0.29 − 0.68)
H2RA	8	116/728 (15.9%)	145/749 (19.4%)	0.80 (0.55 − 1.14)
Any GPT	17	168/1963 (8.6%)	259/1956 (13.2%)	**0.62 (0.50 − 0.77)** p<0.0001
**Total mortality** (Heterogeneity χ^2_1 =0.9, p=0.35)				
PPI	9	34/1235 (2.8%)	44/1207 (3.6%)	0.78 (0.42 − 1.45)
H2RA	6	38/682 (5.6%)	38/704 (5.4%)	1.06 (0.56 − 2.00)
Any GPT	15	72/1917 (3.8%)	82/1911 (4.3%)	**0.90 (0.64 − 1.26)** p=0.53

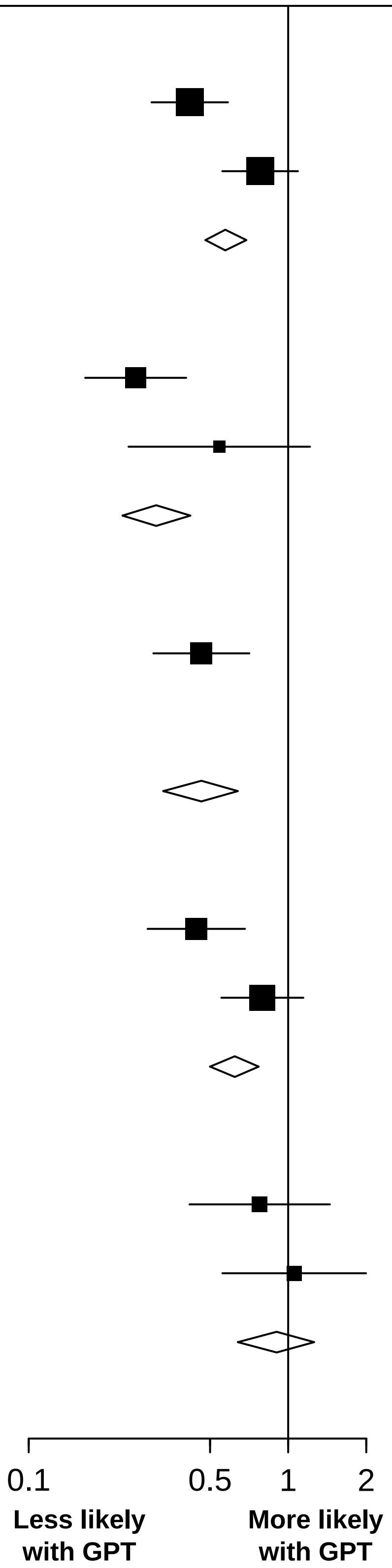

■ 99% or ◇ 95% CI

PPI=Proton pump inhibitors, H2RA=Histamine−2 receptor antagonists, GPT=Gastroprotectant drug

Webfigure 12: Effects of gastroprotectants on further bleeding, need for blood transfusion, endoscopic intervention, surgery and all−cause mortality (acute UGI bleeding treatment trials of uncertain aetiology)

	No.	Events/Patients (%) Allocated GPT	Events/Patients (%) Allocated control	Odds Ratio (95 or 99% CI)
**Further bleeding** (Heterogeneity χ^2_1 =0.1, p=0.79)				
PPI	3	103/999 (10.3%)	117/991 (11.8%)	0.85 (0.58 − 1.25)
H2RA	11	172/806 (21.3%)	196/786 (24.9%)	0.81 (0.59 − 1.11)
Any GPT	14	275/1805 (15.2%)	313/1777 (17.6%)	**0.82 (0.68 − 0.99)** p=0.0378
**Blood transfusion** (Heterogeneity χ^2_1 =1.1, p=0.29)				
PPI	2	365/680 (53.7%)	362/672 (53.9%)	0.99 (0.75 − 1.32)
H2RA	2	189/317 (59.6%)	172/315 (54.6%)	1.22 (0.80 − 1.88)
Any GPT	4	554/997 (55.6%)	534/987 (54.1%)	**1.06 (0.88 − 1.27)** p=0.52
**Endoscopic interventions**				
PPI	3	85/999 (8.5%)	117/991 (11.8%)	0.68 (0.45 − 1.02)
H2RA	0	−	−	
Any GPT	3	85/999 (8.5%)	117/991 (11.8%)	**0.68 (0.49 − 0.93)** p=0.0121
**Surgery** (Heterogeneity χ^2_1 =0.2, p=0.69)				
PPI	3	68/999 (6.8%)	73/991 (7.4%)	0.91 (0.57 − 1.46)
H2RA	7	81/603 (13.4%)	94/600 (15.7%)	0.83 (0.53 − 1.29)
Any GPT	10	149/1602 (9.3%)	167/1591 (10.5%)	**0.87 (0.68 − 1.11)** p=0.24
**Total mortality** (Heterogeneity χ^2_1 =3.1, p=0.08)				
PPI	3	50/999 (5.0%)	42/991 (4.2%)	1.19 (0.67 − 2.11)
H2RA	11	57/844 (6.8%)	75/821 (9.1%)	0.72 (0.44 − 1.18)
Any GPT	14	107/1843 (5.8%)	117/1812 (6.5%)	**0.89 (0.67 − 1.18)** p=0.42

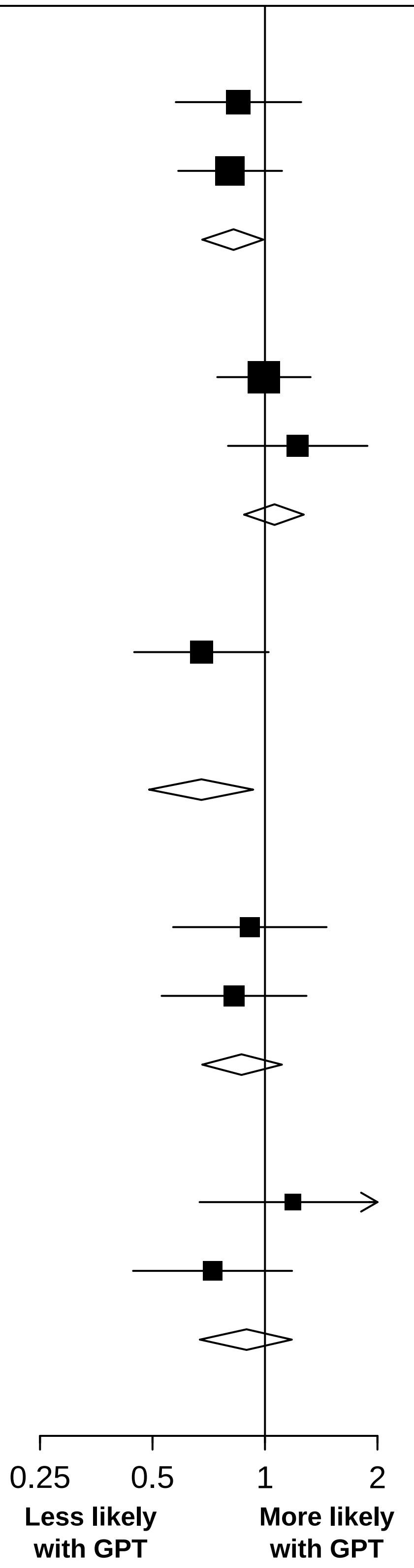

■ 99% or ◇ 95% CI

PPI=Proton pump inhibitors, H2RA=Histamine−2 receptor antagonists, GPT=Gastroprotectant drug

Webfigure 13a: Acute UGI bleeding treatment trials − effects of individual gastroprotectant drugs on further bleeding

	No.	Events/Patients (%) Allocated GPT	Events/Patients (%) Allocated control	Odds Ratio (95 or 99% CI)
**Proton Pump Inhibitor** (Heterogeneity χ^2_3 =5.1, p=0.17)				
Esomeprazole	1	29/376 (7.7%)	53/391 (13.6%)	0.54 (0.29 − 1.02)
Omeprazole	8	141/1542 (9.1%)	220/1549 (14.2%)	0.60 (0.44 − 0.81)
Lansoprazole	1	10/102 (9.8%)	10/103 (9.7%)	1.01 (0.27 − 3.78)
Pantoprazole	2	12/214 (5.6%)	28/155 (18.1%)	0.30 (0.12 − 0.78)
Any PPI	12	192/2234 (8.6%)	311/2198 (14.1%)	**0.57 (0.47 − 0.69)** p<0.0001
**Histamine−2 Receptor Antagonists** (Heterogeneity χ^2_2 =1.9, p=0.39)				
Cimetidine	12	160/801 (20.0%)	185/780 (23.7%)	0.78 (0.57 − 1.09)
Famotidine	2	119/507 (23.5%)	133/518 (25.7%)	0.89 (0.60 − 1.30)
Ranitidine	4	41/179 (22.9%)	58/180 (32.2%)	0.60 (0.31 − 1.17)
Any H2RA	18	320/1487 (21.5%)	376/1478 (25.4%)	**0.79 (0.67 − 0.95)** p=0.0084
**Any gastroprotectant drug**				
Any GPT	30	512/3721 (13.8%)	687/3676 (18.7%)	**0.68 (0.60 − 0.78)** p<0.0001

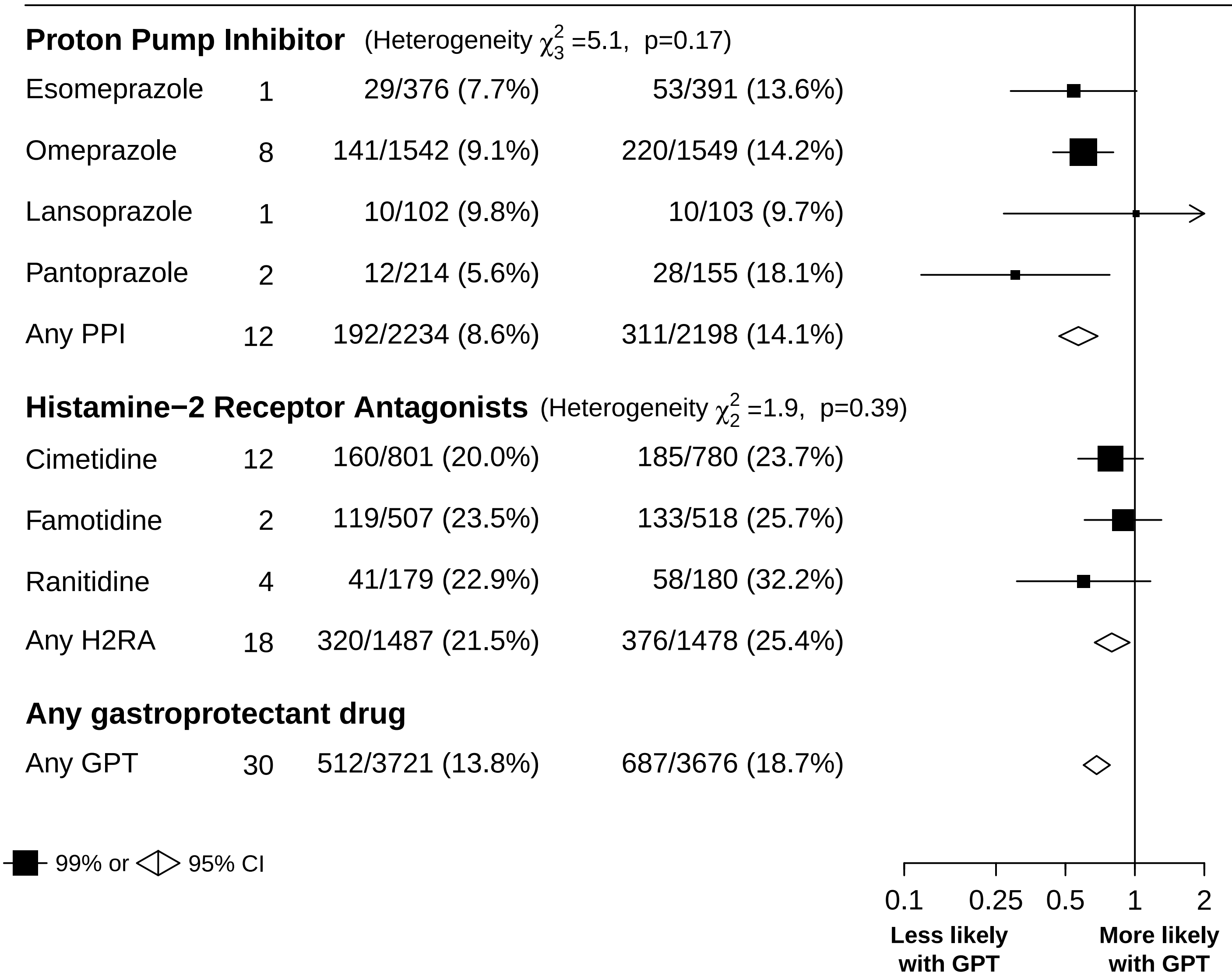

PPI=Proton pump inhibitors, H2RA=Histamine−2 receptor antagonists, GPT=Gastroprotectant drug

Webfigure 13b: Acute UGI bleeding treatment trials − effects of individual gastroprotectant drugs on blood transfusion

	No.	Events/Patients (%) Allocated GPT	Events/Patients (%) Allocated control	Odds Ratio (95 or 99% CI)
**Proton Pump Inhibitor** (Heterogeneity χ^2_2 =8.4, p=0.0146)				
Esomeprazole	0	−	−	
Omeprazole	3	359/770 (46.6%)	441/763 (57.8%)	0.64 (0.49 − 0.84)
Lansoprazole	1	67/102 (65.7%)	60/103 (58.3%)	1.37 (0.63 − 2.99)
Pantoprazole	1	23/102 (22.5%)	41/101 (40.6%)	0.44 (0.19 − 0.99)
Any PPI	5	449/974 (46.1%)	542/967 (56.0%)	**0.67 (0.55 − 0.80)** p<0.0001
**Histamine−2 Receptor Antagonists** (Heterogeneity χ^2_1 =0.4, p=0.52)				
Cimetidine	4	229/384 (59.6%)	218/374 (58.3%)	1.04 (0.70 − 1.55)
Famotidine	0	−	−	
Ranitidine	1	37/50 (74.0%)	41/52 (78.8%)	0.77 (0.21 − 2.82)
Any H2RA	5	266/434 (61.3%)	259/426 (60.8%)	**1.01 (0.76 − 1.35)** p=0.92
**Any gastroprotectant drug**				
Any GPT	10	715/1408 (50.8%)	801/1393 (57.5%)	**0.75 (0.65 − 0.88)** p=0.0003

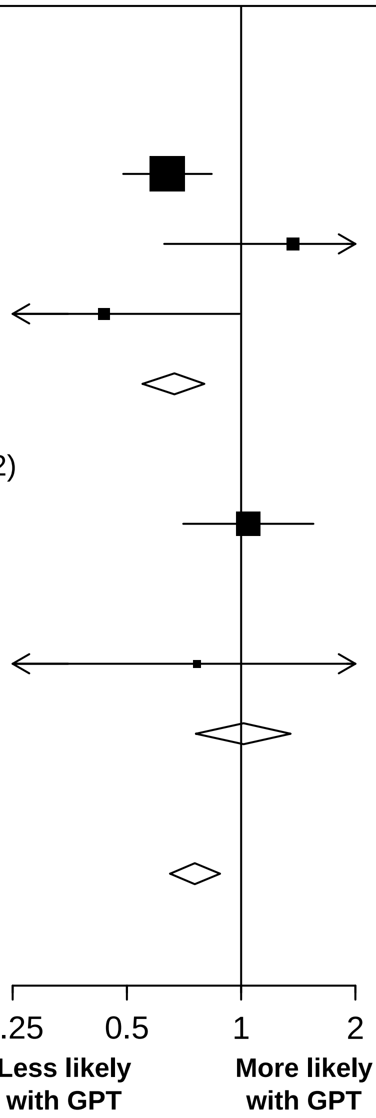

■ 99% or ◇ 95% CI

PPI=Proton pump inhibitors, H2RA=Histamine−2 receptor antagonists, GPT=Gastroprotectant drug

Webfigure 13c: Acute UGI bleeding treatment trials − effects of individual gastroprotectant drugs on endoscopic interventions

	No.	Events/Patients (%) Allocated GPT	Allocated control	Odds Ratio (95 or 99% CI)
**Proton Pump Inhibitor** (Heterogeneity χ^2_3 =2.6, p=0.45)				
Esomeprazole	1	24/376 (6.4%)	45/391 (11.5%)	0.54 (0.27 − 1.06)
Omeprazole	5	99/1310 (7.6%)	161/1311 (12.3%)	0.57 (0.40 − 0.81)
Lansoprazole	1	10/102 (9.8%)	10/103 (9.7%)	1.01 (0.27 − 3.78)
Pantoprazole	1	7/102 (6.9%)	18/101 (17.8%)	0.36 (0.11 − 1.20)
Any PPI	8	140/1890 (7.4%)	234/1906 (12.3%)	**0.56 (0.45 − 0.70)** p<0.0001
**Histamine−2 Receptor Antagonists**				
Cimetidine	0	−	−	
Famotidine	0	−	−	
Ranitidine	0	−	−	
Any H2RA	0	−	−	
**Any gastroprotectant drug**				
Any GPT	8	140/1890 (7.4%)	234/1906 (12.3%)	**0.56 (0.45 − 0.70)** p<0.0001

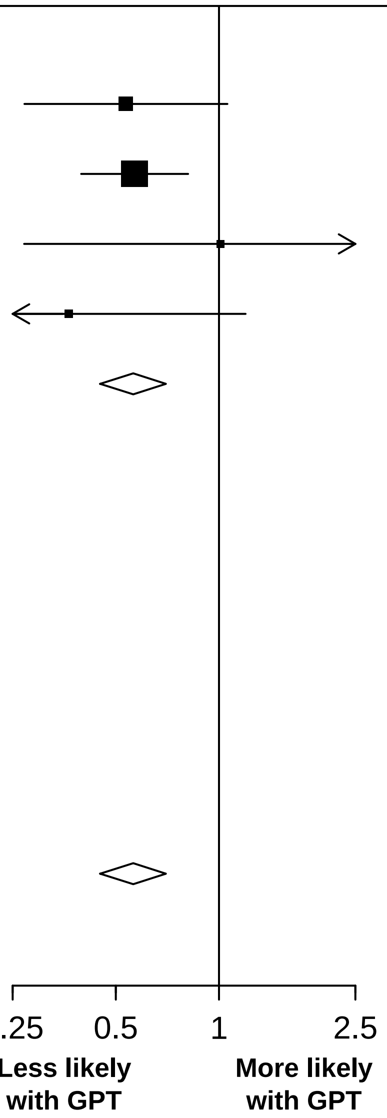

99% or 95% CI

PPI=Proton pump inhibitors, H2RA=Histamine−2 receptor antagonists, GPT=Gastroprotectant drug

Webfigure 13d: Acute UGI bleeding treatment trials − effects of individual gastroprotectant drugs on surgery

	No.	Events/Patients (%) Allocated GPT	Events/Patients (%) Allocated control	Odds Ratio (95 or 99% CI)
**Proton Pump Inhibitor** (Heterogeneity χ^2_3 =3.5, p=0.32)				
Esomeprazole	1	10/376 (2.7%)	21/391 (5.4%)	0.50 (0.18 − 1.37)
Omeprazole	8	103/1542 (6.7%)	148/1549 (9.6%)	0.67 (0.47 − 0.96)
Lansoprazole	1	3/102 (2.9%)	6/103 (5.8%)	0.50 (0.07 − 3.67)
Pantoprazole	2	4/214 (1.9%)	12/155 (7.7%)	0.26 (0.06 − 1.16)
Any PPI	12	120/2234 (5.4%)	187/2198 (8.5%)	**0.62 (0.48 − 0.78)** p<0.0001
**Histamine−2 Receptor Antagonists** (Heterogeneity χ^2_2 =0.5, p=0.76)				
Cimetidine	8	81/614 (13.2%)	102/618 (16.5%)	0.77 (0.50 − 1.20)
Famotidine	2	77/507 (15.2%)	87/518 (16.8%)	0.89 (0.56 − 1.40)
Ranitidine	5	39/210 (18.6%)	50/213 (23.5%)	0.73 (0.38 − 1.42)
Any H2RA	15	197/1331 (14.8%)	239/1349 (17.7%)	**0.81 (0.65 − 1.00)** p=0.0445
**Any gastroprotectant drug**				
Any GPT	27	317/3565 (8.9%)	426/3547 (12.0%)	**0.72 (0.61 − 0.84)** p<0.0001

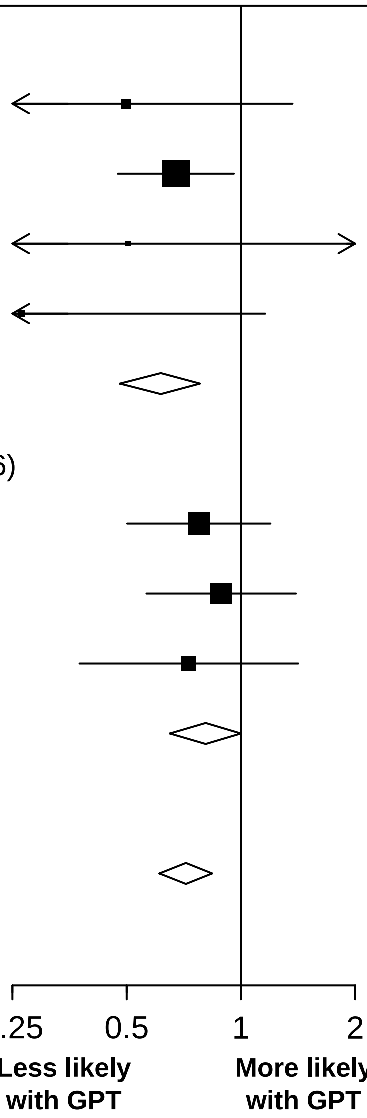

■ 99% or ◇ 95% CI

PPI=Proton pump inhibitors, H2RA=Histamine−2 receptor antagonists, GPT=Gastroprotectant drug

Webfigure 13e: Acute UGI bleeding treatment trials − effects of individual gastroprotectant drugs on all−cause mortality

	No.	Events/Patients (%) Allocated GPT	Events/Patients (%) Allocated control	Odds Ratio (95 or 99% CI)
**Proton Pump Inhibitor** (Heterogeneity χ^2_3 =5.7, p=0.13)				
Esomeprazole	1	3/376 (0.8%)	8/391 (2.0%)	0.41 (0.07 − 2.38)
Omeprazole	8	77/1542 (5.0%)	68/1549 (4.4%)	1.14 (0.73 − 1.80)
Lansoprazole	1	2/102 (2.0%)	5/103 (4.9%)	0.42 (0.04 − 4.05)
Pantoprazole	2	2/214 (0.9%)	5/155 (3.2%)	0.37 (0.04 − 3.59)
Any PPI	12	84/2234 (3.8%)	86/2198 (3.9%)	**0.98 (0.71 − 1.34)** p=0.88
**Histamine−2 Receptor Antagonists** (Heterogeneity χ^2_2 =3.7, p=0.16)				
Cimetidine	13	55/870 (6.3%)	78/857 (9.1%)	0.68 (0.42 − 1.11)
Famotidine	1	31/497 (6.2%)	25/508 (4.9%)	1.28 (0.61 − 2.71)
Ranitidine	3	9/159 (5.7%)	10/160 (6.2%)	0.89 (0.24 − 3.39)
Any H2RA	17	95/1526 (6.2%)	113/1525 (7.4%)	**0.83 (0.62 − 1.12)** p=0.21
**Any gastroprotectant drug**				
Any GPT	29	179/3760 (4.8%)	199/3723 (5.3%)	**0.90 (0.72 − 1.11)** p=0.31

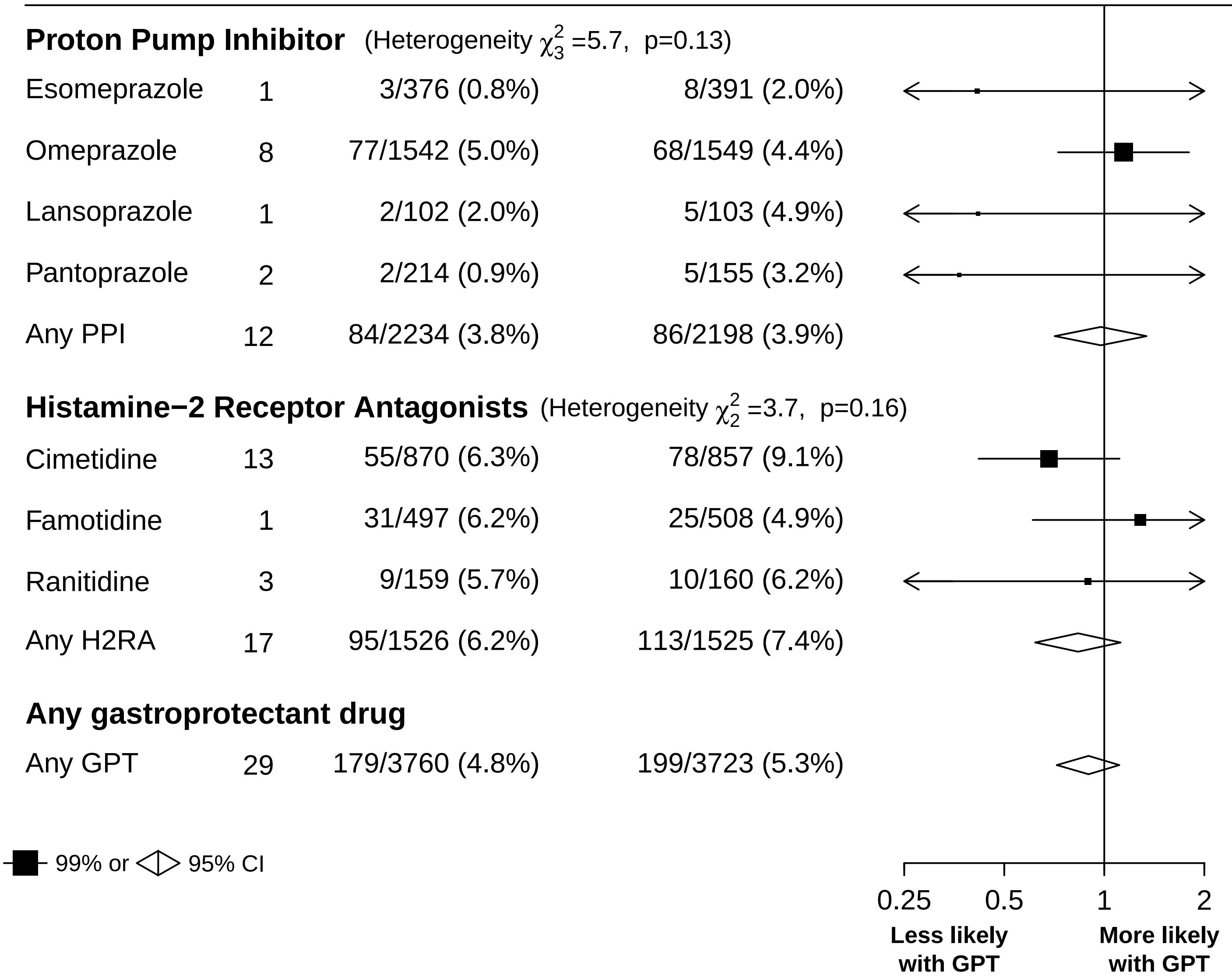

PPI=Proton pump inhibitors, H2RA=Histamine−2 receptor antagonists, GPT=Gastroprotectant drug

Webfigure 14a: Prevention trials − effects of gastroprotectants on endoscopic and symptomatic ulcers, bleeds and all−cause mortality, subdivided by method of allocation concealment (secure vs other method)

Allocation concealment	No.	Events/Patients (%) Allocated GPT	Events/Patients (%) Allocated control	Odds Ratio (95 or 99% CI)
**Any endoscopic ulcer** (Heterogeneity χ^2_1 =0.2, p=0.62)				
Secure method	13	265/3594 (7.4%)	495/2494 (19.8%)	0.26 (0.21 − 0.33)
Other method	139	1624/14448 (11.2%)	3007/10761 (27.9%)	0.27 (0.25 − 0.30)
Total	152	1889/18042 (10.5%)	3502/13255 (26.4%)	**0.27 (0.25 − 0.29)** p<0.0001
**Any symptomatic ulcer** (Heterogeneity χ^2_1 =16.4, p<0.0001)				
Secure method	7	130/6987 (1.9%)	254/7021 (3.6%)	0.39 (0.28 − 0.54)
Other method	44	393/2870 (13.7%)	882/2423 (36.4%)	0.22 (0.18 − 0.26)
Total	51	523/9857 (5.3%)	1136/9444 (12.0%)	**0.25 (0.22 − 0.29)** p<0.0001
**Bleeds** (Heterogeneity χ^2_1 =14.0, p=0.0002)				
Secure method	10	62/8948 (0.7%)	102/8197 (1.2%)	0.59 (0.39 − 0.90)
Other method	39	28/5191 (0.5%)	119/3933 (3.0%)	0.25 (0.16 − 0.39)
Total	49	90/14139 (0.6%)	221/12130 (1.8%)	**0.40 (0.32 − 0.50)** p<0.0001
**All−cause mortality** (Heterogeneity χ^2_1 =0.0, p=0.84)				
Secure method	14	202/10556 (1.9%)	220/9084 (2.4%)	0.83 (0.54 − 1.27)
Other method	37	272/5288 (5.1%)	276/3979 (6.9%)	0.87 (0.61 − 1.23)
Total	51	474/15844 (3.0%)	496/13063 (3.8%)	**0.85 (0.69 − 1.04)** p=0.11

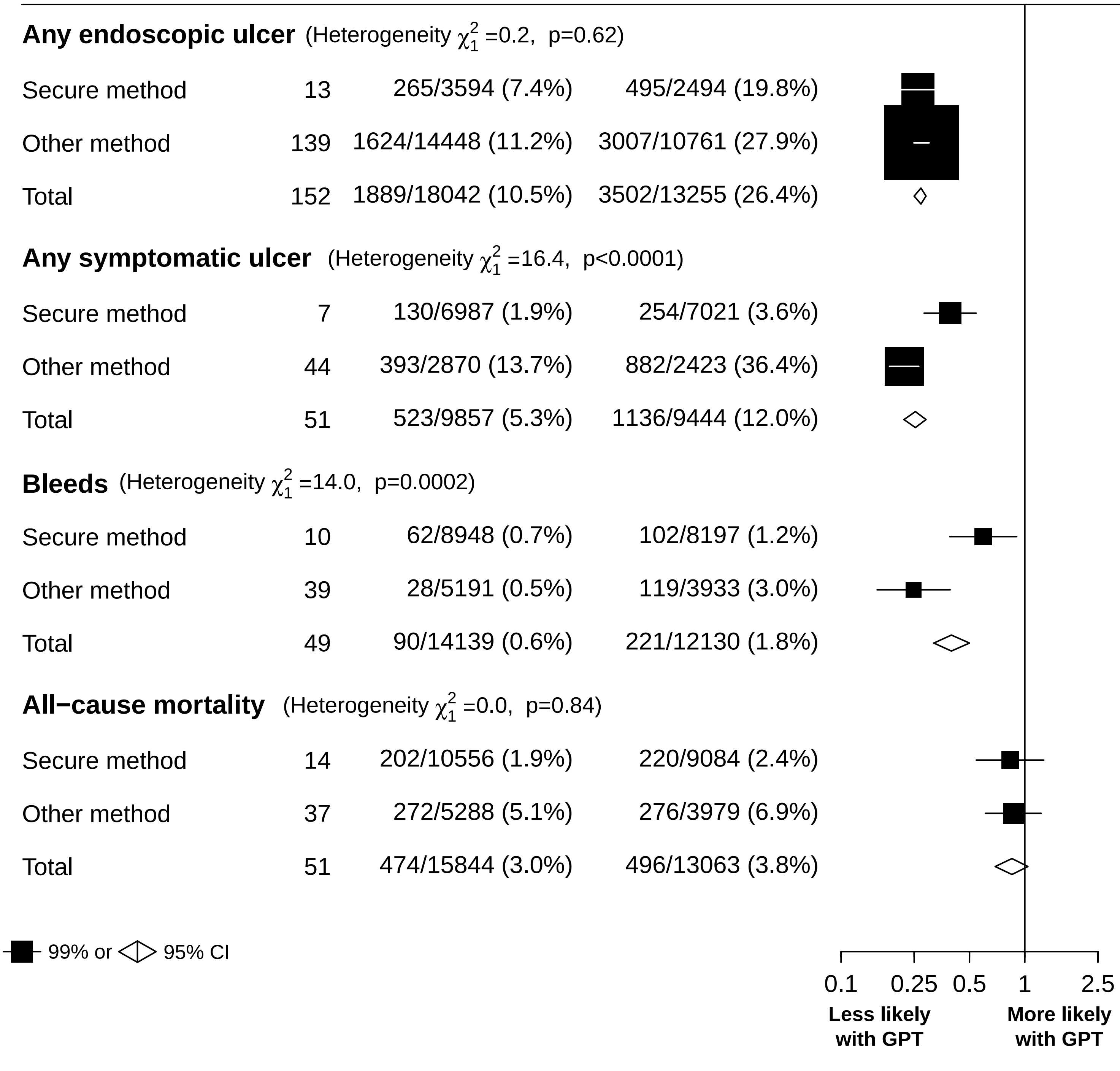

Secure methods of allocation concealment were considered to be: IVRS (Interactive Voice Response System); telephone central office; pharmacy coded containers administered sequentially; and onsite computer accessible only after entering patients details. Other methods included sealed envelopes, closed lists of random numbers, or unspecified method.

Webfigure 14b: Prevention trials − effects of gastroprotectants on endoscopic and symptomatic ulcers, bleeds and all−cause mortality, subdivided by methods of blinding of investigators and patients

Blinding method	No.	Events/Patients (%) Allocated GPT	Events/Patients (%) Allocated control	Odds Ratio (95 or 99% CI)
**Any endoscopic ulcer** (Heterogeneity χ^2_1 =8.2, p=0.0041)				
Double	124	1556/15916 (9.8%)	3021/11631 (26.0%)	0.26 (0.24 − 0.29)
Other	28	333/2126 (15.7%)	481/1624 (29.6%)	0.35 (0.27 − 0.44)
Total	152	1889/18042 (10.5%)	3502/13255 (26.4%)	**0.27 (0.25 − 0.29)** p<0.0001
**Any symptomatic ulcer** (Heterogeneity χ^2_1 =1.1, p=0.28)				
Double	41	368/8798 (4.2%)	883/8565 (10.3%)	0.24 (0.20 − 0.29)
Other	10	155/1059 (14.6%)	253/879 (28.8%)	0.28 (0.21 − 0.39)
Total	51	523/9857 (5.3%)	1136/9444 (12.0%)	**0.25 (0.22 − 0.29)** p<0.0001
**Bleeds** (Heterogeneity χ^2_1 =11.9, p=0.0006)				
Double	37	86/12846 (0.7%)	177/11084 (1.6%)	0.47 (0.34 − 0.66)
Other	12	4/1293 (0.3%)	44/1046 (4.2%)	0.16 (0.07 − 0.35)
Total	49	90/14139 (0.6%)	221/12130 (1.8%)	**0.40 (0.32 − 0.50)** p<0.0001
**All−cause mortality** (Heterogeneity χ^2_1 =1.1, p=0.30)				
Double	42	370/15328 (2.4%)	381/12580 (3.0%)	0.90 (0.67 − 1.21)
Other	9	104/516 (20.2%)	115/483 (23.8%)	0.69 (0.37 − 1.28)
Total	51	474/15844 (3.0%)	496/13063 (3.8%)	**0.85 (0.69 − 1.04)** p=0.11

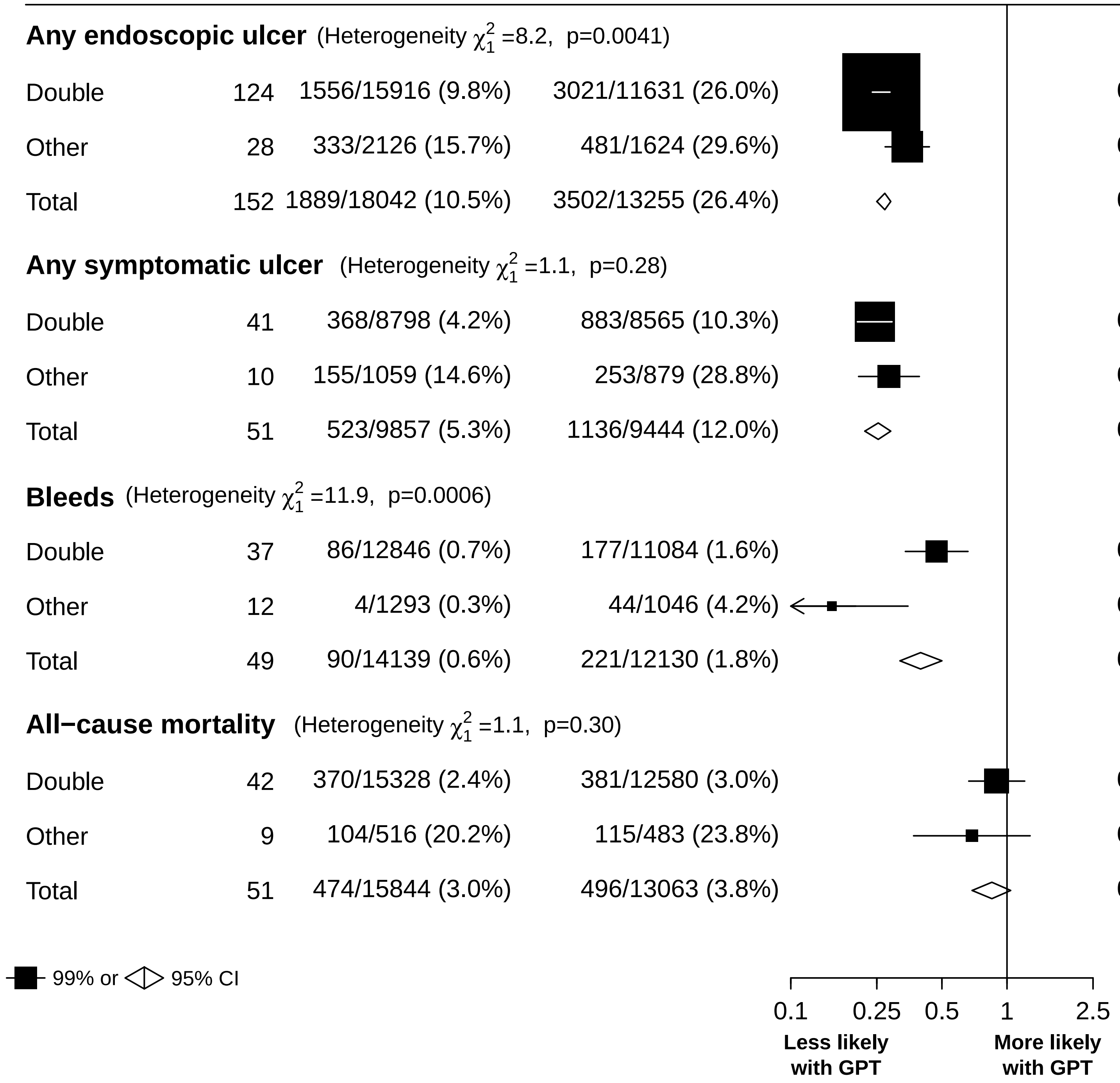

Double=double−blind (ie, both patients and investigators were unaware of the treatment allocation)

Webfigure 15a: Prevention trials − funnel plots of each gastroprotectant class vs control for particular outcomes

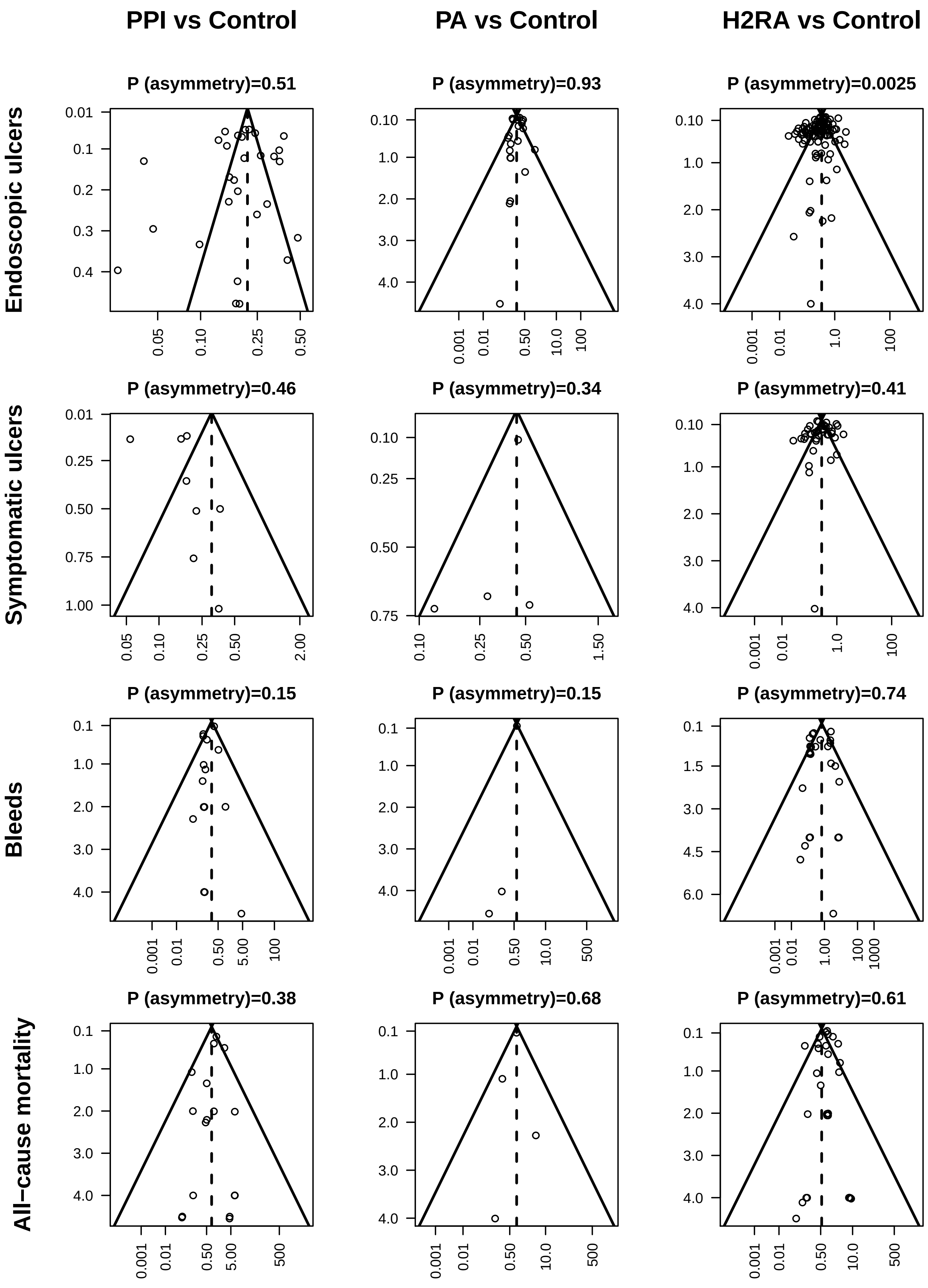

Each point represents the result from a single trial. In each panel, the odds ratio is shown on the x−axis (on a doubling scale) and the standard error of the log−odds ratio is shown on the y−axis. The vertical dashed line in each panel represent the information−weighted average odds ratio across the trials.

P (asymmetry) is the corresponding p−value of Egger's test for asymmetry of the funnel plot.

Webfigure 15b: Healing trials − funnel plots of each gastroprotectant class vs control for endoscopic ulcer healing

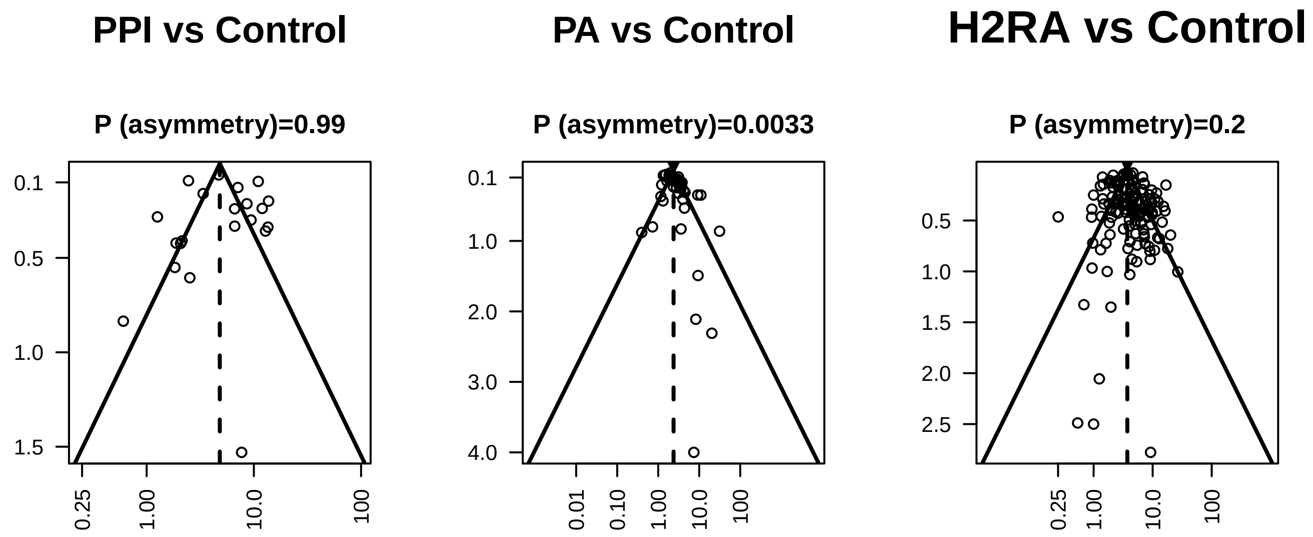

Each point represents the result from a single trial. In each panel, the odds ratio is shown on the x−axis (on a doubling scale) and the standard error of the log−odds ratio is shown on the y−axis. The vertical dashed line in each panel represent the information−weighted average odds ratio across the trials.

P (asymmetry) is the corresponding p−value of Egger's test for asymmetry of the funnel plot.

Webfigure 15c: Acute bleeding trials − funnel plots of each gastroprotectant class vs control for particular outcomes

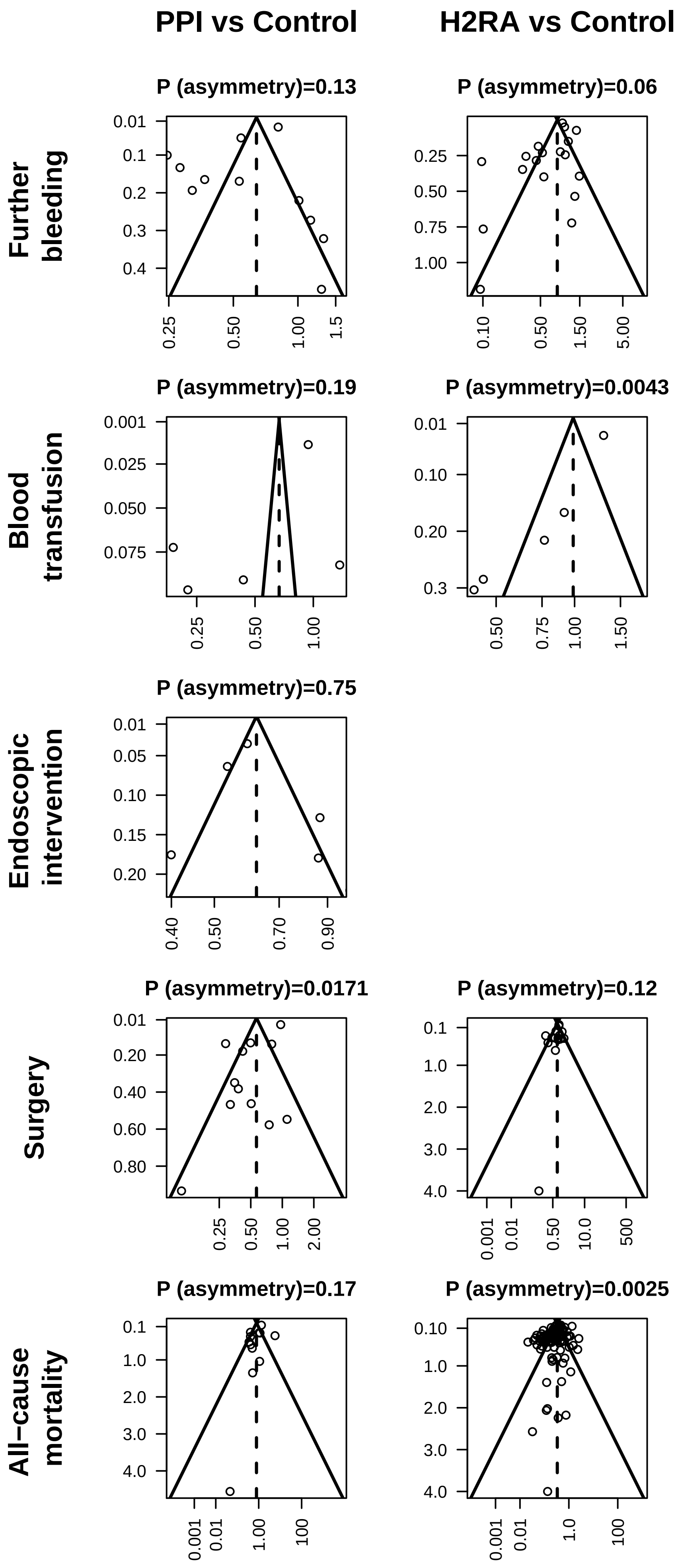

Each point represents the result from a single trial. In each panel, the odds ratio is shown on the x−axis (on a doubling scale) and the standard error of the log−odds ratio is shown on the y−axis. The vertical dashed line in each panel represent the information−weighted average odds ratio across the trials.

P (asymmetry) is the corresponding p−value of Egger's test for asymmetry of the funnel plot.

Webfigure 16a: Prevention trials − effects of gastroprotectants on any endoscopic ulcers (direct vs network meta−analytic effect estimates)

	No.	Events/Patients (%) Allocated Active	Events/Patients (%) Allocated Comparator	Odds Ratio (95 or 99% CI)
**Gastroprotectant vs control** (Heterogeneity χ^2_2 =39.5, p<0.0001)				
PPI	29	314/6541 (4.8%)	937/4912 (19.1%)	0.20 (0.17 − 0.23)
				0.18 (0.16 − 0.21)
PA	22	228/3427 (6.7%)	435/2532 (17.2%)	0.26 (0.20 − 0.32)
				0.27 (0.22 − 0.32)
H2RA	105	1347/8074 (16.7%)	2298/6187 (37.1%)	0.32 (0.28 − 0.35)
				0.32 (0.29 − 0.36)
GPT	152	1889/18042 (10.5%)	3502/13255 (26.4%)	**0.27 (0.25 − 0.29)** p<0.0001
**Gastroprotectant vs gastroprotectant**				
PPI−H2RA	10	179/1746 (10.3%)	233/1170 (19.9%)	0.39 (0.29 − 0.52)
				0.56 (0.48 − 0.66)
PPI−PA	3	94/800 (11.8%)	80/688 (11.6%)	0.94 (0.60 − 1.47)
				0.68 (0.54 − 0.84)
H2RA−PA	8	70/659 (10.6%)	95/665 (14.3%)	0.68 (0.42 − 1.10)
				1.20 (0.98 − 1.48)

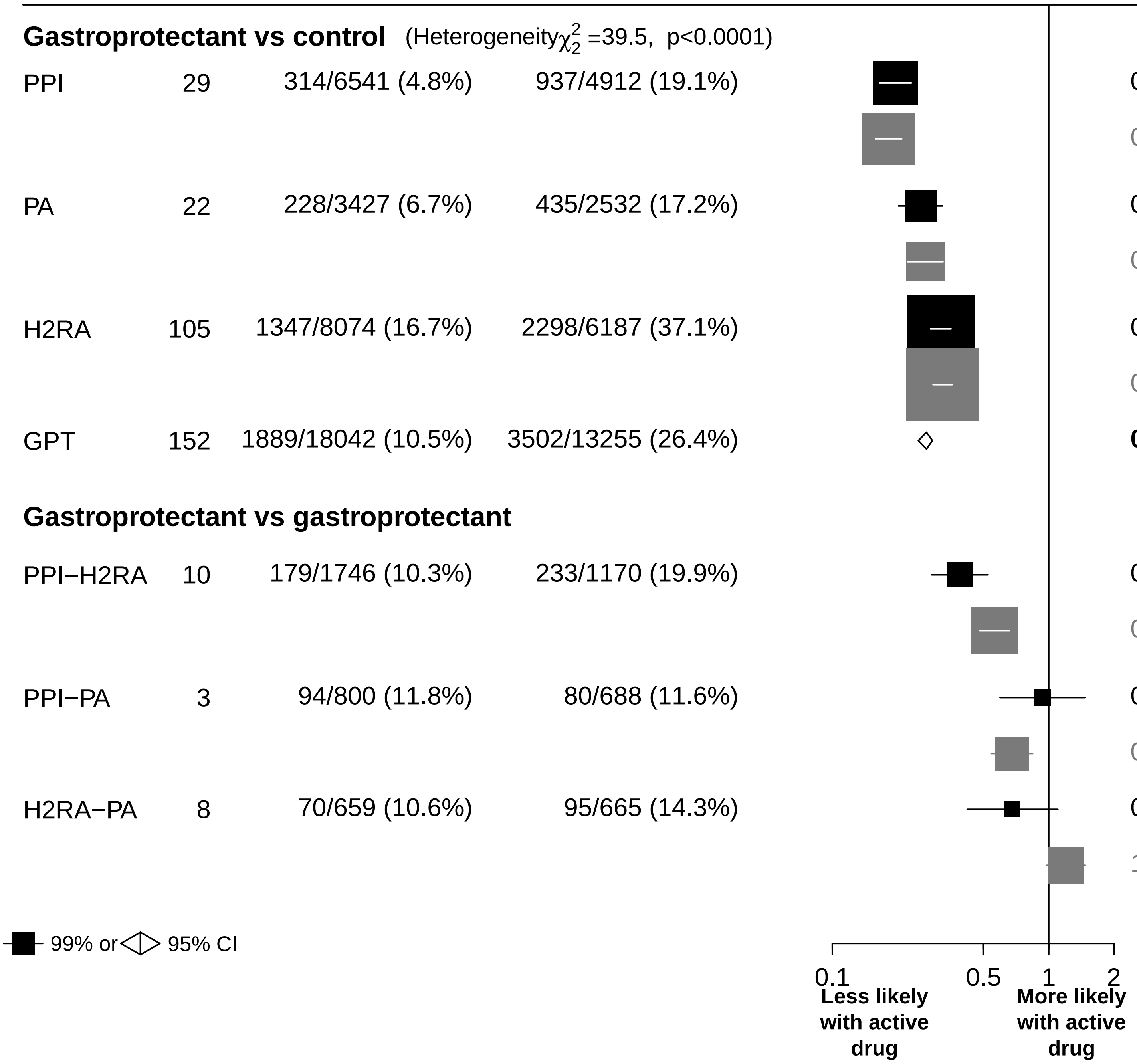

PPI=Proton pump inhibitors, PA=Prostaglandin analogues, H2RA=Histamine−2 receptor antagonists, GPT=Any gastroprotectant drug

Black squares and lines correspond to the direct treatment comparisons and grey squares and lines to the network meta−analysis estimates.

Webfigure 16b: Prevention trials − effects of gastroprotectants on any symptomatic ulcers (direct vs network meta−analytic effect estimates)

	No.	Events/Patients (%) Allocated Active	Events/Patients (%) Allocated Comparator	Odds Ratio (95 or 99% CI)
**Gastroprotectant vs control** (Heterogeneity χ^2_2 =12.7, p=0.0018)				
PPI	8	28/2585 (1.1%)	152/2587 (5.9%)	0.15 (0.09 − 0.23)
				0.15 (0.11 − 0.20)
PA	4	14/4619 (0.3%)	41/4673 (0.9%)	0.38 (0.18 − 0.79)
				0.45 (0.25 − 0.83)
H2RA	39	481/2653 (18.1%)	943/2184 (43.2%)	0.27 (0.23 − 0.32)
				0.26 (0.22 − 0.31)
GPT	51	523/9857 (5.3%)	1136/9444 (12.0%)	**0.25 (0.22 − 0.29)** p<0.0001
**Gastroprotectant vs gastroprotectant**				
PPI−H2RA	3	106/1074 (9.9%)	95/647 (14.7%)	0.56 (0.37 − 0.86)
				0.56 (0.41 − 0.76)
PPI−PA	0	−	−	
				0.33 (0.17 − 0.64)
H2RA−PA	1	7/64 (10.9%)	17/64 (26.6%)	0.36 (0.10 − 1.28)
				0.59 (0.32 − 1.08)

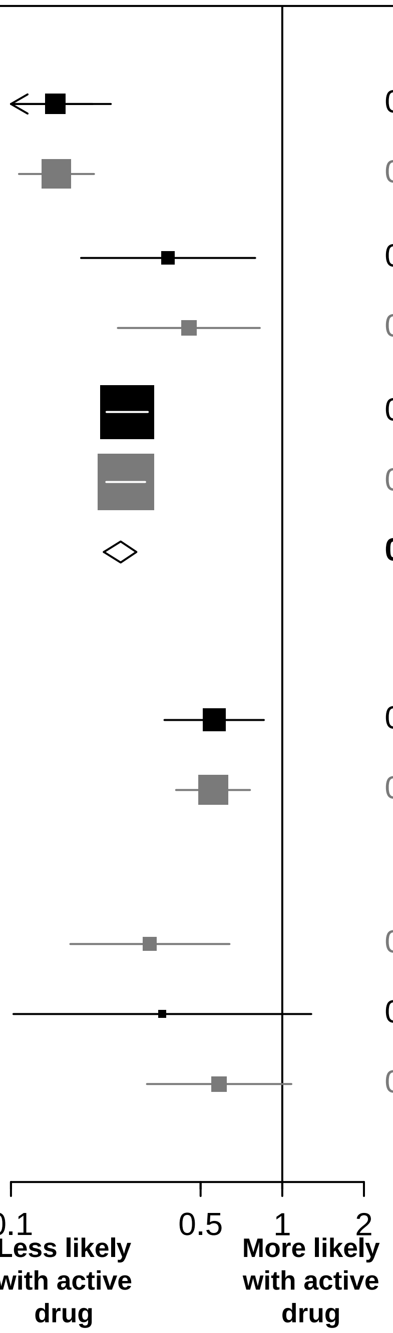

99% or 95% CI

PPI=Proton pump inhibitors, PA=Prostaglandin analogues, H2RA=Histamine−2 receptor antagonists, GPT=Any gastroprotectant drug

Black squares and lines correspond to the direct treatment comparisons and grey squares and lines to the network meta−analysis estimates.

Webfigure 16c: Prevention trials − effects of gastroprotectants on bleeds (direct vs network meta−analytic effect estimates)

	No.	Events/Patients (%) Allocated Active	Events/Patients (%) Allocated Comparator	Odds Ratio (95 or 99% CI)
**Gastroprotectant vs control** (Heterogeneity χ^2_2 =15.3, p=0.0005)				
PPI	18	14/5910 (0.2%)	88/4937 (1.8%)	0.21 (0.12 − 0.36)
				0.21 (0.13 − 0.33)
PA	3	33/4464 (0.7%)	53/4489 (1.2%)	0.63 (0.35 − 1.12)
				0.61 (0.35 − 1.06)
H2RA	29	43/3631 (1.2%)	84/2779 (3.0%)	0.49 (0.30 − 0.80)
				0.51 (0.33 − 0.78)
GPT	49	90/14139 (0.6%)	221/12130 (1.8%)	**0.40 (0.32 − 0.50)** p<0.0001
**Gastroprotectant vs gastroprotectant**				
PPI−H2RA	12	9/1299 (0.7%)	24/1180 (2.0%)	0.36 (0.13 − 0.94)
				0.41 (0.23 − 0.71)
PPI−PA	1	1/269 (0.4%)	0/134 (0.0%)	
				0.34 (0.17 − 0.70)
H2RA−PA	0	−	−	
				0.83 (0.41 − 1.68)

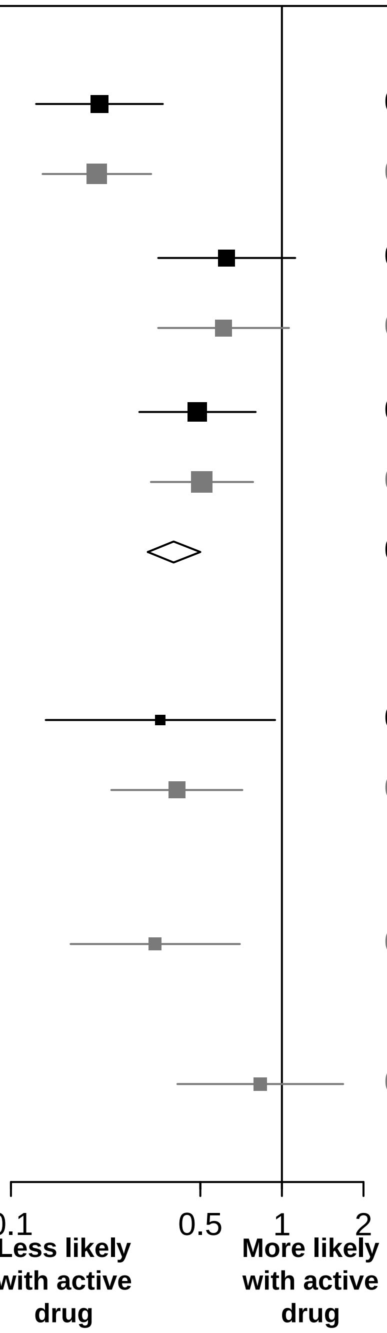

99% or 95% CI

PPI=Proton pump inhibitors, PA=Prostaglandin analogues, H2RA=Histamine−2 receptor antagonists, GPT=Any gastroprotectant drug

Black squares and lines correspond to the direct treatment comparisons and grey squares and lines to the network meta−analysis estimates.

Webfigure 16d: Prevention trials − effects of gastroprotectants on all−cause mortality (direct vs network meta−analytic effect estimates)

	No.	Events/Patients (%) Allocated Active	Events/Patients (%) Allocated Comparator	Odds Ratio (95 or 99% CI)
**Gastroprotectant vs control** (Heterogeneity χ^2_2 =0.5, p=0.77)				
PPI	18	37/7471 (0.5%)	31/5352 (0.6%)	1.01 (0.50 − 2.05)
				1.09 (0.61 − 1.95)
PA	4	17/4853 (0.4%)	20/4752 (0.4%)	0.81 (0.32 − 2.00)
				0.74 (0.32 − 1.69)
H2RA	29	420/3520 (11.9%)	445/2959 (15.0%)	0.83 (0.61 − 1.13)
				0.83 (0.61 − 1.11)
GPT	51	474/15844 (3.0%)	496/13063 (3.8%)	**0.85 (0.69 − 1.04)** p=0.11
**Gastroprotectant vs gastroprotectant**				
PPI−H2RA	7	13/1151 (1.1%)	7/831 (0.8%)	1.39 (0.38 − 5.05)
				1.32 (0.71 − 2.45)
PPI−PA	1	2/257 (0.8%)	0/258 (0.0%)	
				1.48 (0.54 − 4.01)
H2RA−PA	0	−	−	
				1.12 (0.46 − 2.70)

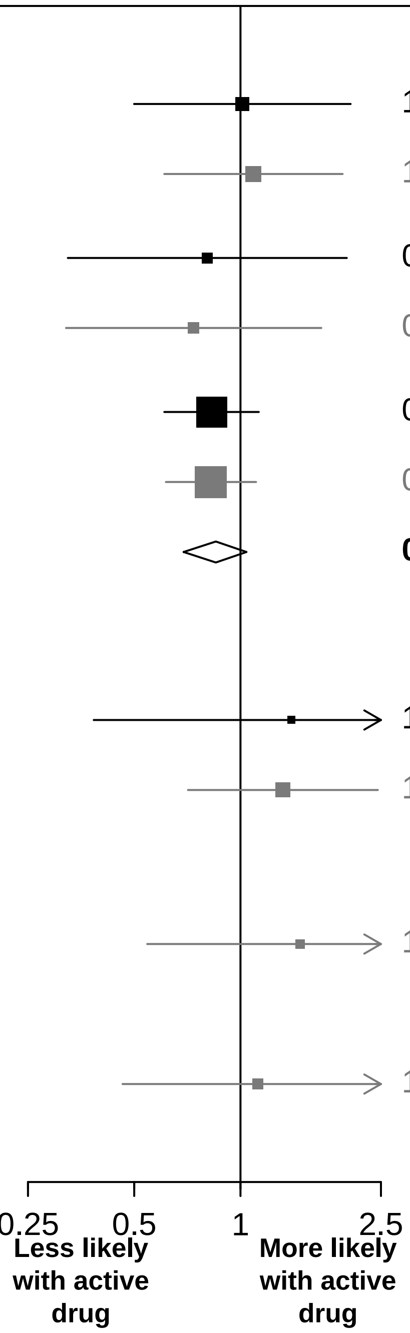

99% or 95% CI

PPI=Proton pump inhibitors, PA=Prostaglandin analogues, H2RA=Histamine−2 receptor antagonists, GPT=Any gastroprotectant drug

Black squares and lines correspond to the direct treatment comparisons and grey squares and lines to the network meta−analysis estimates.

Webfigure 16e: Healing trials − effects of gastroprotectants on endoscopic ulcer healing (direct vs network meta−analytic effect estimates)

	No.	Events/Patients (%) Allocated Active	Allocated Comparator	Odds Ratio (95 or 99% CI)
**Gastroprotectant vs control** (Heterogeneity χ^2_2 =62.0, p<0.0001)				
PPI	24	1650/1925 (85.7%)	650/1033 (62.9%)	5.22 (4.00 − 6.80)
				7.95 (6.99 − 9.04)
PA	37	1501/2452 (61.2%)	751/1729 (43.4%)	2.27 (1.91 − 2.70)
				2.30 (2.03 − 2.60)
H2RA	148	5714/7533 (75.9%)	2676/5670 (47.2%)	3.80 (3.44 − 4.20)
				3.56 (3.26 − 3.88)
GPT	207	8865/11910 (74.4%)	4050/8373 (48.4%)	**3.49 (3.28 − 3.72)** p<0.0001
**Gastroprotectant vs gastroprotectant**				
PPI−H2RA	106	8214/9117 (90.1%)	5786/7245 (79.9%)	2.58 (2.28 − 2.92)
				2.23 (2.00 − 2.49)
PPI−PA	4	457/538 (84.9%)	267/338 (79.0%)	1.57 (0.96 − 2.57)
				3.46 (2.96 − 4.04)
H2RA−PA	37	2345/2872 (81.7%)	2320/3296 (70.4%)	1.68 (1.42 − 1.99)
				1.55 (1.37 − 1.75)

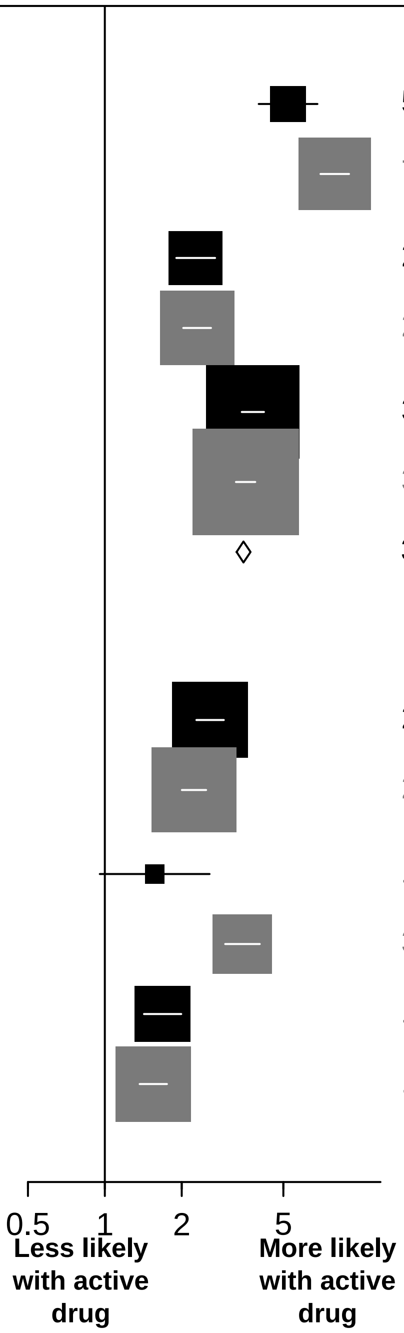

99% or 95% CI

PPI=Proton pump inhibitors, PA=Prostaglandin analogues, H2RA=Histamine−2 receptor antagonists, GPT=Any gastroprotectant drug

Black squares and lines correspond to the direct treatment comparisons and grey squares and lines to the network meta−analysis estimates.

Webfigure 16f: Acute UGI bleeding treatment trials − effects of gastroprotectants on further bleeding (direct vs network meta−analytic effect estimates)

	No.	Events/Patients (%) Allocated Active	Events/Patients (%) Allocated Comparator	Odds Ratio (95 or 99% CI)
**Gastroprotectant vs control** (Heterogeneity χ^2_1 =6.5, p=0.0107)				
PPI	12	192/2234 (8.6%)	311/2198 (14.1%)	0.57 (0.44 − 0.73)
				0.57 (0.44 − 0.73)
H2RA	18	320/1487 (21.5%)	376/1478 (25.4%)	0.79 (0.63 − 1.00)
				0.79 (0.63 − 0.99)
GPT	30	512/3721 (13.8%)	687/3676 (18.7%)	**0.68 (0.60 − 0.78)** p<0.0001
**Gastroprotectant vs gastroprotectant**				
PPI−H2RA	16	101/1737 (5.8%)	162/1658 (9.8%)	0.51 (0.36 − 0.72)
				0.72 (0.51 − 1.00)

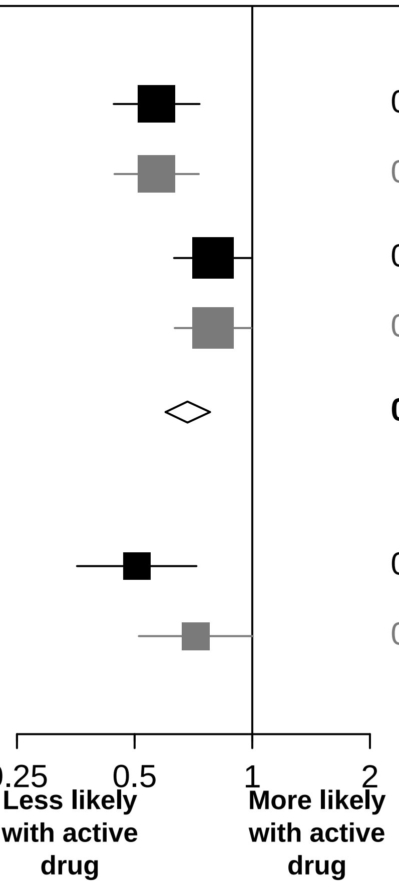

99% or 95% CI

PPI=Proton pump inhibitors, H2RA=Histamine−2 receptor antagonists, GPT=Any gastroprotectant drug

Black squares and lines correspond to the direct treatment comparisons and grey squares and lines to the network meta−analysis estimates.

Webfigure 16g: Acute UGI bleeding treatment trials − effects of gastroprotectants on blood transfusion (direct vs network meta−analytic effect estimates)

	No.	Events/Patients (%) Allocated Active	Allocated Comparator	Odds Ratio (95 or 99% CI)
**Gastroprotectant vs control** (Heterogeneity χ^2_1 =6.2, p=0.0130)				
PPI	5	449/974 (46.1%)	542/967 (56.0%)	0.67 (0.52 − 0.85)
				0.67 (0.53 − 0.84)
H2RA	5	266/434 (61.3%)	259/426 (60.8%)	1.01 (0.70 − 1.48)
				1.01 (0.70 − 1.46)
GPT	10	715/1408 (50.8%)	801/1393 (57.5%)	**0.75 (0.65 − 0.88)** p=0.0003
**Gastroprotectant vs gastroprotectant**				
PPI−H2RA	1	115/267 (43.1%)	112/252 (44.4%)	0.95 (0.59 − 1.52)
				0.66 (0.43 − 1.02)

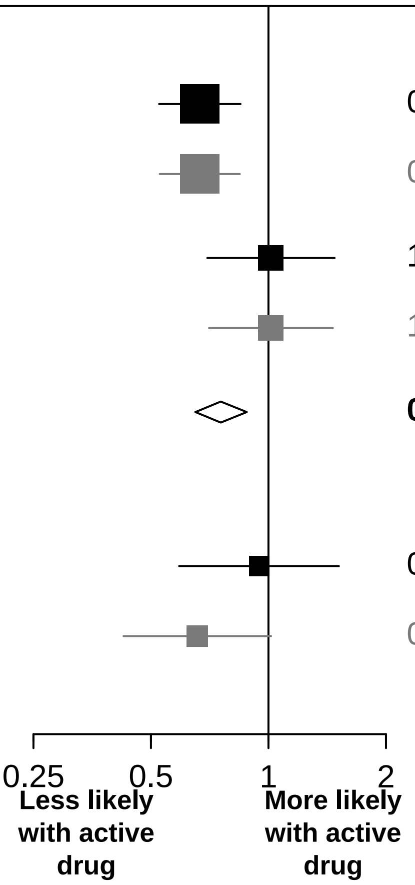

99% or 95% CI

PPI=Proton pump inhibitors, H2RA=Histamine−2 receptor antagonists, GPT=Any gastroprotectant drug

Black squares and lines correspond to the direct treatment comparisons and grey squares and lines to the network meta−analysis estimates.

Webfigure 16h: Acute UGI bleeding treatment trials − effects of gastroprotectants on surgery (direct vs network meta−analytic effect estimates)

	No.	Events/Patients (%) Allocated Active	Events/Patients (%) Allocated Comparator	Odds Ratio (95 or 99% CI)
**Gastroprotectant vs control** (Heterogeneity χ^2_1 =2.9, p=0.09)				
PPI	12	120/2234 (5.4%)	187/2198 (8.5%)	0.62 (0.45 − 0.84)
				0.62 (0.45 − 0.84)
H2RA	15	197/1331 (14.8%)	239/1349 (17.7%)	0.81 (0.61 − 1.07)
				0.81 (0.61 − 1.06)
GPT	27	317/3565 (8.9%)	426/3547 (12.0%)	**0.72 (0.61 − 0.84)** p<0.0001
**Gastroprotectant vs gastroprotectant**				
PPI−H2RA	10	37/1395 (2.7%)	52/1319 (3.9%)	0.67 (0.37 − 1.20)
				0.76 (0.50 − 1.15)

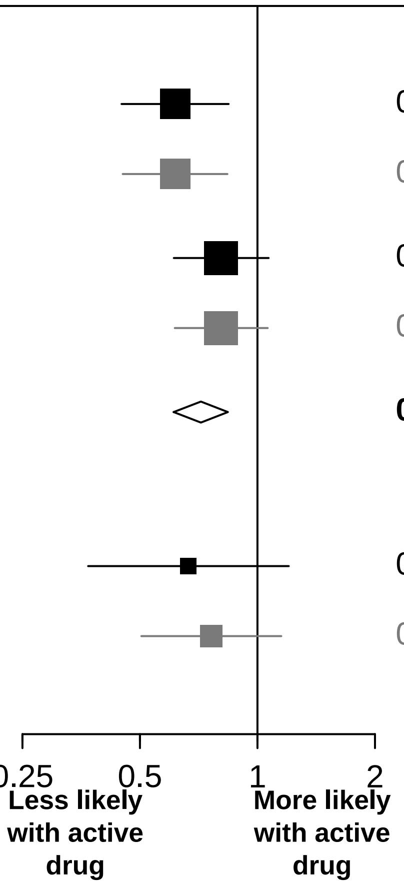

99% or 95% CI

PPI=Proton pump inhibitors, H2RA=Histamine−2 receptor antagonists, GPT=Any gastroprotectant drug

Black squares and lines correspond to the direct treatment comparisons and grey squares and lines to the network meta−analysis estimates.

Webfigure 16i: Acute UGI bleeding treatment trials − effects of gastroprotectants on all−cause mortality (direct vs network meta−analytic effect estimates)

	No.	Events/Patients (%) Allocated Active	Events/Patients (%) Allocated Comparator	Odds Ratio (95 or 99% CI)
**Gastroprotectant vs control** (Heterogeneity χ^2_1 =0.5, p=0.46)				
PPI	12	84/2234 (3.8%)	86/2198 (3.9%)	0.98 (0.64 − 1.48)
				0.98 (0.65 − 1.46)
H2RA	17	95/1526 (6.2%)	113/1525 (7.4%)	0.83 (0.57 − 1.22)
				0.83 (0.57 − 1.21)
GPT	29	179/3760 (4.8%)	199/3723 (5.3%)	**0.90 (0.72 − 1.11)** p=0.31
**Gastroprotectant vs gastroprotectant**				
PPI−H2RA	11	19/1520 (1.2%)	23/1446 (1.6%)	0.74 (0.31 − 1.76)
				1.17 (0.67 − 2.03)

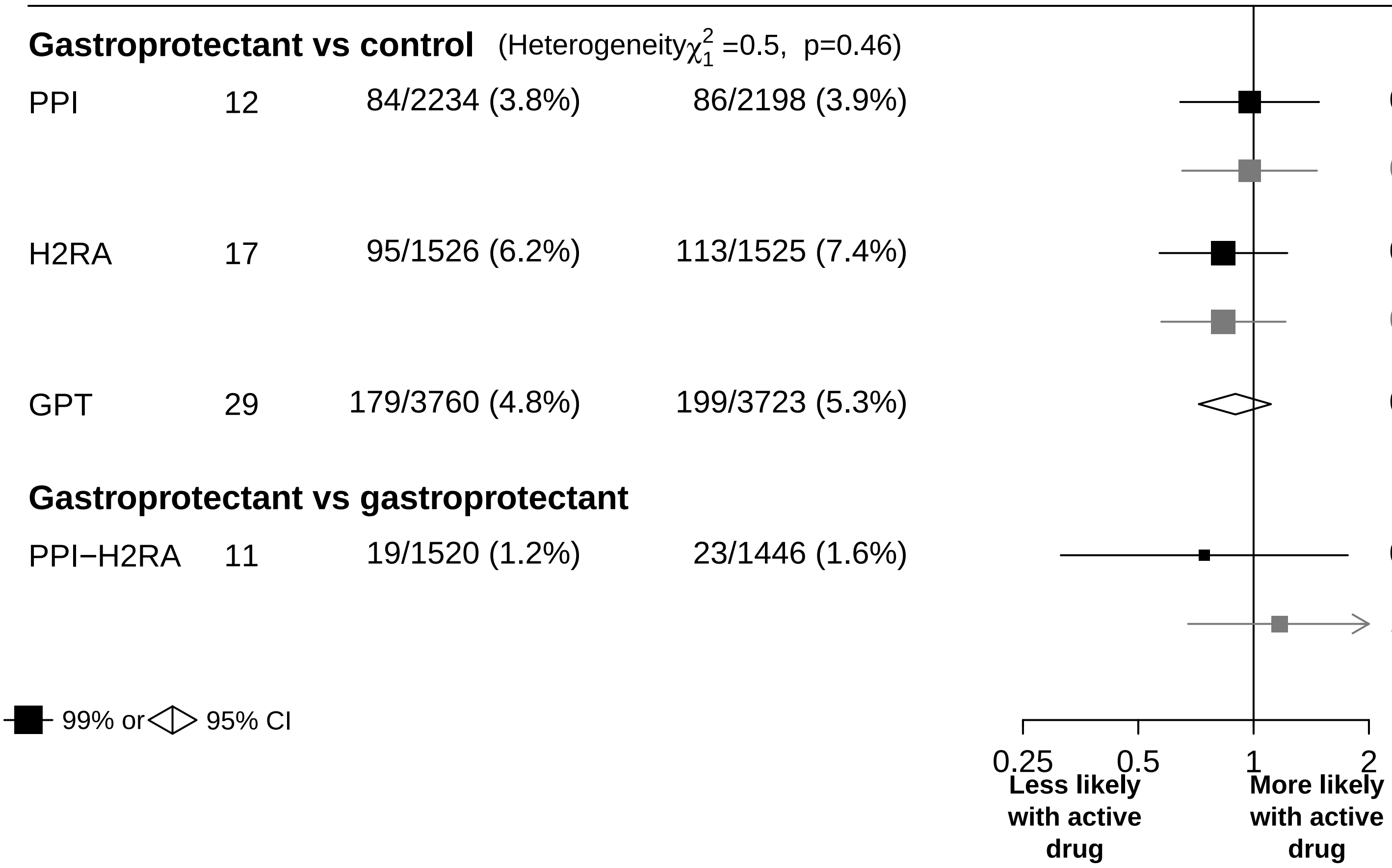

PPI=Proton pump inhibitors, H2RA=Histamine−2 receptor antagonists, GPT=Any gastroprotectant drug

Black squares and lines correspond to the direct treatment comparisons and grey squares and lines to the network meta−analysis estimates.

Electronic Search Strategy

Medline and EMBASE were searched using OVID SP for all trial publications (including protocols, results papers, abstracts, conferences proceedings and reviews) by adapting the first Cochrane Search Strategy Design (Higgins & Green 2008[1]). No language restriction was applied to the search. The search was completed by LH and results were downloaded into EndNote X7 where duplicates were removed. The remaining references were initially screened on the basis of title with abstracts and full papers obtained where further information was required. Reasons for exclusion were recorded in custom fields in EndNote. The review of search results and extraction from eligible studies of pre-specified outcomes and key trial characteristics was done independently by two authors (always two of KD, HH, LH and KW). The extracted information was then entered directly into a customised database.

All eligibility criteria were decided *a priori*. Acceptable methods of concealment of allocation at the point of randomisation were: IVRS (Interactive Voice Response System); telephone central office; pharmacy coded containers administered sequentially; onsite computer accessible only after entering patient details; sealed envelopes sequentially numbered or opaque; sealed envelopes (no further information); closed list of random numbers and 'randomised', but method not specified. Acceptable methods of blinding were: double blind (subject and investigators unaware of allocation; single blind (subject only unaware of allocation); open (subjects and investigators aware of allocation) and not described (unknown).

Medline Search (Ovid MEDLINE(R) In-Process & Other Non-Indexed Citations and Ovid MEDLINE(R) 1950 to January Week 3 2016

1. proton pump inhibitor.mp.
2. (proton AND pump AND inhibitor).mp.
3. (proton AND pump AND inhibitors).mp.
4. Proton pump inhibitors/ OR proton pump inhibitors.mp.
5. PPI.mp.
6. PPIs.mp.
7. dexlansoprazole.mp.
8. esomeprazole.mp.
9. tenatoprazole.mp.
10. benatoprazole.mp.
11. rabeprazole.mp.
12. omeprazole.mp or omeprazole/
13. lansoprazole.mp.
14. pantoprazole.mp.
15. ilaprazole.mp.
16. AZD0865.mp.
17. histamine H2 receptor antagonist.mp.
18. histamine H2 receptor antagonists.mp.
19. (histamine AND H2 receptor AND antagonist).mp.
20. (histamine AND H2 receptor AND antagonist).mp.
21. histamine h2 antagonists/ OR histamine H2 antagonists.mp.
22. (histamine AND H2 AND antagonist).mp.
23. (histamine AND H2 AND antagonists).mp.
24. H2RA.mp.
25. H2RAs.mp.
26. cimetidine/ OR cimetidine.mp.
27. famotidine/ OR famotidine.mp.
28. ranitidine/ OR ranitidine.mp.
29. nizatidine/ OR nizatidine.mp.
30. lafutidine.mp.
31. roxatidine.mp.
32. prostaglandin E2 analogues.mp. OR Prostaglandin E2 analogue.mp.
33. Prostaglandins E, Synthetic/
34. (prostaglandin AND analogue AND E2).mp.
35. (prostaglandin AND analogues AND E2).mp.
36. PGE2 analogue.mp.
37. prostaglandin E2 derivative.mp.
38. (prostaglandin AND E2 AND derivative).mp.

39. (prostaglandin AND E2 AND derivatives).mp.
40. misoprostol/ OR misoprostol.mp.
41. rioprostil.mp. OR rioprostil/
42. arbaprostil/ OR arbaprostil.mp.
43. enprostil/ OR enprostil.mp.
44. rebamipide.mp.
45. /or 1-44
46. randomized controlled trial.pt.
47. controlled clinical trial.pt.
48. randomized.ab.
49. placebo.ab.
50. clinical trials as topic.sh.
51. randomly.ab.
52. trial.ti.
53. /or 46-52
54. exp animals/ not humans.sh.
55. 53 not 54
56. 45 and 55

EMBASE via OVID SP 1980 to 2016 Week 05

1. Proton pump inhibitor.mp. OR proton pump inhibitor/
2. Proton pump inhibitors.mp.
3. (proton AND pump AND inhibitor).mp.
4. (proton AND pump AND inhibitors).mp
5. PPI.mp.
6. PPIs.mp.
7. dexlansoprazole/ OR dexlansoprazole.mp.
8. esomeprazole.mp. OR esomeprazole/
9. tenatoprazole/ OR tenatoprazole.mp.
10. benatoprazole/ OR benatoprazole.mp.
11. rabeprazole/ OR rabeprazole.mp.
12. omeprazole/ OR omeprazole.mp.
13. lansoprazole/ OR lansoprazole.mp.
14. pantoprazole/ OR pantoprazole.mp.
15. ilaprazole.mp.
16. AZD0865.mp.
17. histamine H2 receptor antagonist.mp. OR histamine H2 receptor antagonist/
18. histamine H2 receptor antagonists.mp.
19. histamine H2 antagonists.mp. OR Histamine H2 Antagonists/
20. histamine H2 antagonist.mp. OR Histamine H2 Antagonist/
21. (histamine AND H2 receptor AND antagonist).mp.
22. (histamine AND H2 receptor AND antagonists).mp.
23. (histamine AND H2 AND antagonist).mp.
24. (histamine AND H2 AND antagonists).mp.
25. H2RA.mp.
26. H2RAs.mp.
27. cimetidine/ OR cimetidine.mp.
28. famotidine/ OR famotidine.mp.
29. ranitidine/ OR ranitidine.mp.
30. nizatidine/ OR nizatidine.mp.
31. lafutidine.mp.
32. roxatidine.mp.
33. prostaglandin E2 analogues.mp.
34. prostaglandin E2 analogue.mp.
35. (prostaglandin AND E2 AND analogue).mp.
36. (prostaglandin AND E2 AND analogues).mp.
37. PGE2 analogue.mp.
38. prostaglandin E2 derivative.mp. or prostaglandin E2 derivative/
39. (prostaglandin AND E2 AND derivative).mp.
40. (prostaglandin AND E2 AND derivatives).mp.

41. misoprostol/ OR misoprostol.mp.
42. rioprostil.mp. OR rioprostil/
43. arbaprostil/ OR arbaprostil.mp.
44. enprostil/ OR enprostil.mp.
45. rebamipide.mp.
46. /or 1-45
47. random$.mp.
48. factorial$.mp.
49. crossover$.mp.
50. cross over$.mp.
51. cross-over$.mp.
52. placebo$.mp.
53. (doubl$ adj blind$).mp.
54. (singl$ adj blind$).mp.
55. assign$.mp
56. allocat$.mp.
57. volunteer$.mp. OR volunteer/
58. crossover procedure/
59. double-blind procedure/
60. randomized controlled trial/
61. single blind procedure/
62. /or 47-61
63. 62 and 46

Further suitable drugs were identified during publication processing. These were oxmetidine and ramixotidine and eligible trials using these drugs were included.

[1] Higgins JPT & Green S (editors). Cochrane Handbook for Systematic Reviews of Interventions Version 5.0.1 [updated September 2008]. The Cochrane Collaboration, 2008. Available from www.cochrane-handbook.org.

Webtable 1: Details of trials involving a randomised comparison of a proton pump inhibitor (X) versus control (Y)

Trial name	Regimen	Ref.	Indication for treatment	Design	Allocation Concealment	Blinding of investigators and patients	Availability of data	No. of patients NX	NY
**Prevention Trials**									
Dexlansoprazole vs Control									
Metz 2009	30 mg	w1	Gastro-oesophageal reflux	Parallel	Telephone	Double	Yes	140	147
Fass 2011	30 mg	w2	Gastro-oesophageal reflux	Parallel	IVRS	Double	Yes	152	153
Howden 2009	60 mg	w3	Gastro-oesophageal reflux	Parallel	IVRS	Double	Yes	159	140
Metz 2009	60 mg	w1	Gastro-oesophageal reflux	Parallel	Telephone	Double	Yes	158	147
Howden 2009	90 mg	w3	Gastro-oesophageal reflux	Parallel	IVRS	Double	Yes	152	140
Esomeprazole vs Control									
Johnson 2001	10 mg	w4	Gastro-oesophageal reflux	Parallel	Sealed envelopes	Double	Yes	77	77
Vakil 2001	10 mg	w5	Gastro-oesophageal reflux	Parallel	Sealed envelopes	Double	No	91	94
PLUTO	20 mg	w6	Arthritis, rheumatic or musculoskeletal disorders	Parallel	Closed list	Double	Yes	195	192
ASTERIX	20 mg	w7	Antiplatelet therapy	Parallel	Onsite computer	Double	Yes	494	498
VENUS	20 mg	w6	Arthritis, rheumatic or musculoskeletal disorders	Parallel	Other method	Double	Yes	281	281
NASA1	20 mg	w8	Antiplatelet therapy	Parallel	Unspecified method	Double	No	195	207
SPACE1	20 mg	w8	Antiplatelet therapy	Parallel	Unspecified method	Double	Yes	187	189
Johnson 2005	20 mg	w9	Gastro-oesophageal reflux	Parallel	Closed list	Double	No	226	229
Johnson 2001	20 mg	w4	Gastro-oesophageal reflux	Parallel	Sealed envelopes	Double	Yes	82	77
NASA2 and SPACE2	20 mg	w10	Antiplatelet therapy	Parallel	Closed list	Double	Yes	202	212
Katz 2003 A	20 mg	w11	Gastro-oesophageal reflux	Parallel	Sealed envelopes	Double	No	121	124
Vakil 2001	20 mg	w5	Gastro-oesophageal reflux	Parallel	Sealed envelopes	Double	No	98	94
Katz 2003 B	20 mg	w11	Gastro-oesophageal reflux	Parallel	Sealed envelopes	Double	No	113	118
Cheung 2013	20 mg	w12	Gastro-oesophageal reflux	Parallel	Unspecified method	Double	No	-	-
Hsu 2010	20 mg	w13	Antiplatelet therapy	Parallel	Unspecified method	Unknown	Yes	79	75
Yu 2013 A	20 mg	w14	Cardiovascular disease	Parallel	Unspecified method	Unknown	No	122	148
Tan 2011	20 mg	w15	Gastro-oesophageal reflux	Parallel	Sealed envelopes	Double	No	85	90
Esomeprazole NSAID Preventive Study	20 mg	w16	Recently healed ulcers	Parallel	Onsite computer	Double	Yes	175	168
Hsu 2012	20 mg	w17	Recently healed ulcers	Parallel	Unspecified method	Unknown	Yes	157	161
LAVENDER	20 mg	w18	Recently healed ulcers	Parallel	Onsite computer	Double	Yes	215	215
Peura 2014 A	20 mg	w19	Gastro-oesophageal reflux	Parallel	Unspecified method	Double	Yes	171	169
Peura 2014 B	20 mg	w19	Gastro-oesophageal reflux	Parallel	Unspecified method	Double	Yes	170	171
Pilichiewicz 2013	20 mg	w20	Gastro-oesophageal reflux	Factorial	Unspecified method	Double	No	-	-
Hsu 2011	20 mg	w21	Recently healed ulcers	Parallel	Sealed envelopes	Open	Yes	83	82
Faruqi 2011	20 mg	w22	Chronic cough	Parallel	Unspecified method	Double	Yes	26	25
OBERON	20 mg	w23	Antiplatelet therapy	Parallel	Sealed envelopes	Double	Yes	804	805
Johnson 2010	20 mg	w24	Gastro-oesophageal reflux	Parallel	Unspecified method	Double	Yes	143	133
Reimer 2009	40 mg	w25	Healthy volunteers	Parallel	Sealed envelopes	Double	No	60	60
Wilder-Smith 2009	40 mg	w26	Dental	Parallel	Closed list	Double	Yes	15	15
PLUTO	40 mg	w6	Arthritis, rheumatic or musculoskeletal disorders	Parallel	Closed list	Double	Yes	198	192
VENUS	40 mg	w6	Arthritis, rheumatic or musculoskeletal disorders	Parallel	Other method	Double	Yes	282	281
Chan 2007	40 mg	w27	Arthritis, rheumatic or musculoskeletal disorders	Parallel	Pharmacy	Double	Yes	137	136
NASA1	40 mg	w8	Antiplatelet therapy	Parallel	Unspecified method	Double	No	206	207
SPACE1	40 mg	w8	Antiplatelet therapy	Parallel	Unspecified method	Double	Yes	180	189
Johnson 2005	40 mg	w9	Gastro-oesophageal reflux	Parallel	Closed list	Double	No	220	229
Johnson 2001	40 mg	w4	Gastro-oesophageal reflux	Parallel	Sealed envelopes	Double	Yes	82	77
Fass 2010	40 mg	w28	Gastro-oesophageal reflux	Parallel	Unspecified method	Double	No	24	17
Johnsson 2003	40 mg	w29	Gastro-oesophageal reflux	Parallel	Unspecified method	Double	No	176	92
Johnsson 2003	40 mg	w29	Gastro-oesophageal reflux	Parallel	Unspecified method	Double	No	172	92
NASA2 and SPACE2	40 mg	w10	Antiplatelet therapy	Parallel	Closed list	Double	Yes	196	212
Katz 2003 A	40 mg	w11	Gastro-oesophageal reflux	Parallel	Sealed envelopes	Double	No	123	124
Van Zanten 2006	40 mg	w30	Gastro-oesophageal reflux	Parallel	Unspecified method	Double	Yes	109	115
Vakil 2001	40 mg	w5	Gastro-oesophageal reflux	Parallel	Sealed envelopes	Double	No	92	94

Webtable 1 (cont.): Details of trials involving a randomized comparison of a proton pump inhibitor (X) versus control (Y)

Trial name	Regimen	Ref.	Indication for treatment	Design	Allocation Concealment	Blinding of investigators and patients	Availability of data	No. of patients NX	NY
Reichel 2008	40 mg	w31	Gastro-oesophageal reflux	Parallel	Unspecified method	Double	Yes	32	30
Talley 2007	40 mg	w32	Gastro-oesophageal reflux	Parallel	Other method	Double	Yes	1065	524
Yi 2008	40 mg	w33	Gastro-oesophageal reflux	Parallel	Unspecified method	Unknown	No	15	13
Kiljander 2010	40 mg	w34	Asthma	Parallel	Unspecified method	Double	Yes	313	328
Katz 2003 B	40 mg	w11	Gastro-oesophageal reflux	Parallel	Sealed envelopes	Double	No	118	118
Hove 2013	40 mg	w35	Diabetes	Factorial	Sealed envelopes	Double	Yes	20	21
Fernando 2011	40 mg	w36	Acute coronary syndrome	Crossover	Pharmacy	Double	No	16	16
Liuzzo 2011	40 mg	w37	Cardiovascular disease	Parallel	Pharmacy	Double	No	78	84
Meineche-Schmidt 2011	40 mg	w38	Gastro-oesophageal reflux	Parallel	Sealed envelopes	Double	No	410	397
OBERON	40 mg	w23	Antiplatelet therapy	Parallel	Sealed envelopes	Double	Yes	817	805
PN400-301	40 mg	w39	Antiplatelet therapy	Parallel	IVRS	Double	Yes	218	220
Study PN400-302	40 mg	w40	Antiplatelet therapy	Parallel	IVRS	Double	Yes	212	211
Andersson 2014	40 mg	w41	Healthy volunteers	Crossover	Onsite computer	Unknown	Yes	27	27
Jiang 2013	40 mg	w42	Gastro-oesophageal reflux	Parallel	Unspecified method	Unknown	Yes	60	60
America Lung Association Asthma Clinical Research Centers	80 mg	w43	Asthma	Parallel	Unspecified method	Double	Yes	208	204
Vaezi 2006	80 mg	w44	Gastro-oesophageal reflux	Parallel	Unspecified method	Double	No	95	51
Kiljander 2006	80 mg	w45	Asthma	Parallel	Unspecified method	Unknown	Yes	387	383
Kiljander 2010	80 mg	w34	Asthma	Parallel	Unspecified method	Double	Yes	320	328
DiMango 2014	80 mg	w46	Gastro-oesophageal reflux	Parallel	Unspecified method	Double	No	9	8
Shaheen 2011	80 mg	w47	Chronic cough	Parallel	Unspecified method	Double	Yes	22	18
STARS II	160 mg	w48	Gastro-oesophageal reflux	Parallel	Closed list	Double	Yes	822	428
Byun 2015	Unknown	w49	Chronic cough	Parallel	Unspecified method	Double	No	-	-
Byun 2015	Unknown	w49	Chronic cough	Parallel	Unspecified method	Double	No	-	-
Lansoprazole vs Control									
Peura 2009 A	15 mg	w50	Gastro-oesophageal reflux	Parallel	Other method	Double	Yes	291	284
Graham 2002	15 mg	w51	Recently healed ulcers	Parallel	Other method	Double	Yes	136	134
Wong 2002	15 mg	w52	Gastro-oesophageal reflux	Parallel	Sealed envelopes	Double	No	152	152
Hongo 2008 B	15 mg	w53	Gastro-oesophageal reflux	Parallel	Unspecified method	Double	No	70	72
Kushner 2009	15 mg	w54	Gastro-oesophageal reflux	Parallel	Unspecified method	Double	No	570	564
Peura 2004	15 mg	w55	Gastro-oesophageal reflux	Parallel	Onsite computer	Double	No	305	308
Sasaki 2009	15 mg	w56	COPD	Parallel	Closed list	Single	No	51	52
Kovacs 1999	15 mg	w57	Recently healed ulcers	Parallel	Unspecified method	Double	Yes	15	19
Müller 1997	15 mg	w58	Healthy volunteers	Parallel	Unspecified method	Double	No	10	10
Earnest 1998	15 mg	w59	Gastro-oesophageal reflux	Parallel	Unspecified method	Double	No	72	66
Castell 1996	15 mg	w60	Gastro-oesophageal reflux	Parallel	Unspecified method	Double	Yes	218	213
Lanza 1997	15 mg	w61	Recently healed ulcers	Parallel	Unspecified method	Double	Yes	92	88
Kovacs 1998	15 mg	w62	Recently healed ulcers	Parallel	Unspecified method	Double	Yes	19	15
Sontag 1996	15 mg	w63	Gastro-oesophageal reflux	Parallel	Unspecified method	Double	No	53	56
Robinson 1996	15 mg	w64	Gastro-oesophageal reflux	Parallel	Unspecified method	Double	No	60	57
Richter 1999	15 mg	w65	Gastro-oesophageal reflux	Parallel	Unspecified method	Double	No	82	44
Russo 1997 X3	15 mg	w66	Recently healed ulcers	Extension	Closed list	Double	Yes	30	28
Richter 2000 C	15 mg	w67	Gastro-oesophageal reflux	Parallel	Unspecified method	Double	No	284	70
ELF Study	15 mg	w68	Gastro-oesophageal reflux	Parallel	Onsite computer	Double	No	24	30
Peura 2009 A	30 mg	w50	Gastro-oesophageal reflux	Parallel	Closed list	Double	Yes	277	284
Graham 2002	30 mg	w51	Recently healed ulcers	Parallel	Other method	Double	Yes	133	134
Wong 2002	30 mg	w52	Gastro-oesophageal reflux	Parallel	Sealed envelopes	Double	No	149	152
Hongo 2008 B	30 mg	w53	Gastro-oesophageal reflux	Parallel	Unspecified method	Double	No	78	72
Petite 1991 A	30 mg	w69	Healthy volunteers	Crossover	Unspecified method	Double	Yes	5	5
Peura 2004	30 mg	w55	Gastro-oesophageal reflux	Parallel	Onsite computer	Double	No	308	308
Kovacs 1999	30 mg	w57	Recently healed ulcers	Parallel	Unspecified method	Double	Yes	18	19
Earnest 1998	30 mg	w59	Gastro-oesophageal reflux	Parallel	Unspecified method	Double	No	71	66
Bell 2000	30 mg	w70	Healthy volunteers	Parallel	Unspecified method	Double	No	7	8

Webtable 1 (cont.): Details of trials involving a randomized comparison of a proton pump inhibitor (X) versus control (Y)

Trial name	Regimen	Ref.	Indication for treatment	Design	Allocation Concealment	Blinding of investigators and patients	Availability of data	No. of patients NX	NY
Castell 1996	30 mg	w60	Gastro-oesophageal reflux	Parallel	Unspecified method	Double	Yes	422	213
Brummer 1997	30 mg	w71	Healthy volunteers	Crossover	Unspecified method	Double	No	5	5
Dumper J 2008	30 mg	w72	Globus	Parallel	Other method	Double	No	19	21
Gaetani 1995	30 mg	w73	Healthy volunteers	Crossover	Unspecified method	Double	No	6	6
Jang 2003	30 mg	w74	Pancreaticoduodenectomy (Whipple's procedure)	Parallel	Unspecified method	Unknown	No	18	19
Lai 2003	30 mg	w75	Recently healed ulcers	Parallel	Closed list	Open	Yes	22	21
Pakozdi 2009	30 mg	w76	Multiple sclerosis	Parallel	Unspecified method	Double	Yes	11	10
Kovacs 1998	30 mg	w62	Recently healed ulcers	Parallel	Unspecified method	Double	Yes	15	15
Sontag 1996	30 mg	w63	Gastro-oesophageal reflux	Parallel	Unspecified method	Double	No	54	56
Robinson 1996	30 mg	w64	Gastro-oesophageal reflux	Parallel	Unspecified method	Double	No	56	57
Richter 1999	30 mg	w65	Gastro-oesophageal reflux	Parallel	Unspecified method	Double	No	88	44
Richter 2000 C	30 mg	w67	Gastro-oesophageal reflux	Parallel	Unspecified method	Double	No	288	70
Xia 2003	30 mg	w77	Gastro-oesophageal reflux	Parallel	Unspecified method	Single	No	36	34
Leung 2007	30 mg	w78	Gastro-oesophageal reflux	Parallel	Sealed envelopes	Double	No	78	79
Lai 2002	30 mg	w79	Antiplatelet therapy	Parallel	Closed list	Double	Yes	62	61
DOSAPI	30 mg	w80	Cardiovascular disease	Parallel	Unspecified method	Double	No	40	42
Talwar 2010	30 mg	w81	Cardiovascular disease	Crossover	Unspecified method	Double	No	57	51
Zhang 2015	30 mg	w82	Antiplatelet therapy	Parallel	Unspecified method	Open	Yes	27	26
Feng 2014	30 mg	w83	Gastro-oesophageal reflux	Parallel	Unspecified method	Unknown	Yes	59	59
Fletcher 2011	30 mg	w84	Gastro-oesophageal reflux	Parallel	Unspecified method	Unknown	No	70	35
Jones 2010	30 mg	w85	Gastro-oesophageal reflux	Parallel	Unspecified method	Double	No	69	33
Adamopoulos 2009	40 mg	w86	Antiplatelet therapy	Crossover	Unspecified method	Open	No	16	16
Littner 2005	60 mg	w87	Asthma	Parallel	Unspecified method	Double	No	99	108
Havas 1999	60 mg	w88	Sore throat	Parallel	Unspecified method	Double	Yes	8	7
Earnest 1998	60 mg	w59	Gastro-oesophageal reflux	Parallel	Unspecified method	Double	No	79	66
El-Serag 2001	60 mg	w89	Sore throat	Parallel	Onsite computer	Double	No	12	10
Borjesson 2003	60 mg	w90	Nutcracker oesophagus	Crossover	Unspecified method	Double	No	7	12
Fuchs 1994	60 mg	w91	Healthy volunteers	Crossover	Unspecified method	Single	No	12	12
Meikle 1994	60 mg	w92	Healthy volunteers	Parallel	Unspecified method	Double	No	18	16
Vaezi 2010	60 mg	w93	Post nasal drip	Parallel	Unspecified method	Double	Yes	36	39
Andersson 2014	60 mg	w41	Healthy volunteers	Crossover	Onsite computer	Unknown	Yes	27	27
Takahiko 2011	Unknown	w94	COPD	Parallel	Unspecified method	Open	No	50	50
Takebayashi 2014	Unknown	w95	Diabetes	Parallel	Onsite computer	Open	No	50	50
Inukai 2015	Unknown	w96	Diabetes	Parallel	Unspecified method	Open	No	46	43
Omeprazole vs Control									
Farup 1999	10 mg	w97	Gastro-oesophageal reflux	Parallel	Unspecified method	Double	No	6	10
Bardhan 1998	10 mg	w98	Gastro-oesophageal reflux	Extension	Closed list	Double	No	130	133
Talley 1998 A	10 mg	w99	Gastro-oesophageal reflux	Parallel	Closed list	Double	No	409	426
Lauritsen 1991	10 mg	w100	Recently healed ulcers	Parallel	Closed list	Double	Yes	64	66
Carlsson 1998 (no oesophagitis)	10 mg	w101	Gastro-oesophageal reflux	Parallel	Unspecified method	Double	No	86	88
Bate 1995	10 mg	w102	Gastro-oesophageal reflux	Parallel	Unspecified method	Double	Yes	61	63
Allgood 2005	10 mg	w103	Gastro-oesophageal reflux	Parallel	Unspecified method	Double	Yes	1038	1053
Blum 2000	10 mg	w104	Gastro-oesophageal reflux	Parallel	Unspecified method	Double	Yes	202	203
Richter 2000 B	10 mg	w105	Gastro-oesophageal reflux	Parallel	Unspecified method	Double	Yes	118	123
Venables 1997 B	10 mg	w106	Gastro-oesophageal reflux	Parallel	Unspecified method	Double	Yes	242	253
Laursen 1995 X2	10 mg	w107	Gastro-oesophageal reflux	Extension	Onsite computer	Double	Yes	64	29
Lind 1997	10 mg	w108	Gastro-oesophageal reflux	Parallel	Other method	Double	Yes	199	105
Uemura 2008	10 mg	w109	Gastro-oesophageal reflux	Parallel	Unspecified method	Double	Yes	96	95
OPPULENT	20 mg	w110	Antiplatelet therapy	Parallel	Unspecified method	Double	Yes	83	86
Ekström 1996	20 mg	w111	Arthritis, rheumatic or musculoskeletal disorders	Parallel	Unspecified method	Double	Yes	86	91
Bianchi Porro 1998	20 mg	w112	Osteoarthritis	Parallel	Unspecified method	Double	Yes	57	57

Webtable 1 (cont.): Details of trials involving a randomized comparison of a proton pump inhibitor (X) versus control (Y)

Trial name	Regimen	Ref.	Indication for treatment	Design	Allocation Concealment	Blinding of investigators and patients	Availability of data	No. of patients NX	NY
OMNIUM 1998 X2	20 mg	w113	Recently healed ulcers	Extension	Unspecified method	Double	Yes	274	155
Brun 2000	20 mg	w114	Gastro-oesophageal reflux	Parallel	Unspecified method	Double	No	-	-
Sgouros 2005	20 mg	w115	Schatzki's rings	Parallel	Sealed envelopes	Unknown	No	15	15
Meineche-Schmidt 2004	20 mg	w116	Gastro-oesophageal reflux	Parallel	Closed list	Double	No	273	272
Veldhuyzen van Zanten 1998	20 mg	w117	Gastritis or duodenitis	Parallel	Unspecified method	Double	No	45	47
Farup 1999	20 mg	w97	Gastro-oesophageal reflux	Parallel	Unspecified method	Double	No	8	10
COGENT	20 mg	w118	Dual antiplatelet therapy	Parallel	Other method	Double	Yes	1938	1935
Talley 1998 A	20 mg	w99	Gastro-oesophageal reflux	Parallel	Closed list	Double	No	427	426
Ishizaki 1995	20 mg	w119	Healthy volunteers	Crossover	Unspecified method	Single	Yes	5	5
Marzio 1995 X2	20 mg	w120	Recently healed ulcers	Extension	Unspecified method	Unknown	Yes	36	25
Castell 1996	20 mg	w60	Gastro-oesophageal reflux	Parallel	Unspecified method	Double	Yes	431	213
Brummer 1997	20 mg	w71	Healthy volunteers	Crossover	Unspecified method	Double	No	5	5
Carlsson 1998 (no oesophagitis)	20 mg	w101	Gastro-oesophageal reflux	Parallel	Unspecified method	Double	No	87	88
Hetzel 1988 B	20 mg	w121	Gastro-oesophageal reflux	Parallel	Unspecified method	Double	No	82	32
Goh 1995	20 mg	w122	Recently healed ulcers	Parallel	Closed list	Double	Yes	60	63
Bate 1995	20 mg	w102	Gastro-oesophageal reflux	Parallel	Unspecified method	Double	Yes	69	63
Ford 1994	20 mg	w123	Asthma	Crossover	Unspecified method	Double	No	6	6
Allgood 2005	20 mg	w103	Gastro-oesophageal reflux	Parallel	Unspecified method	Double	Yes	1047	1053
Blohme 1993	20 mg	w124	Renal transplant	Crossover	Unspecified method	Double	No	5	5
Blum 2000	20 mg	w104	Gastro-oesophageal reflux	Parallel	Unspecified method	Double	Yes	193	203
Guslandi 1992	20 mg	w125	Gastro-oesophageal reflux	Parallel	Unspecified method	Double	No	12	12
Garg 1995	20 mg	w126	Varices	Parallel	Unspecified method	Double	No	23	24
Gerson 2005	20 mg	w127	Gastro-oesophageal reflux	Parallel	Pharmacy	Double	Yes	21	19
Hatlebakk 1999	20 mg	w128	Gastro-oesophageal reflux	Parallel	Pharmacy	Double	No	161	159
Sutfin 1989	20 mg	w129	Healthy volunteers	Crossover	Unspecified method	Double	Yes	10	10
Unge 1992	20 mg	w130	Anticoagulation	Crossover	Unspecified method	Double	Yes	13	15
Sartori 2000	20 mg	w131	Cancer or palliative care (non gastrointestinal)	Parallel	Unspecified method	Unknown	Yes	76	75
Sontag 1992	20 mg	w132	Gastro-oesophageal reflux	Parallel	Unspecified method	Double	No	93	46
Richter 2000 B	20 mg	w105	Gastro-oesophageal reflux	Parallel	Unspecified method	Double	Yes	118	123
Simon 1995 A	20 mg	w133	Healthy volunteers	Parallel	Unspecified method	Double	No	12	12
Sontag 1997 A	20 mg	w134	Gastro-oesophageal reflux	Parallel	Unspecified method	Double	No	138	131
Howard 1994	20 mg	w135	Gastro-oesophageal reflux	Parallel	Unspecified method	Double	No	18	17
Rokkas 2001	20 mg	w136	Gastro-oesophageal reflux	Parallel	Onsite computer	Open	Yes	40	40
Laursen 1995 X2	20 mg	w107	Gastro-oesophageal reflux	Extension	Onsite computer	Double	Yes	65	29
Bate 1996	20 mg	w137	Gastro-oesophageal reflux	Parallel	Unspecified method	Double	Yes	98	111
Heijerman 1993	20 mg	w138	Cystic fibrosis	Crossover	Unspecified method	Double	Yes	6	6
Lind 1997	20 mg	w108	Gastro-oesophageal reflux	Parallel	Other method	Double	Yes	205	105
Levin 1998	20 mg	w139	Asthma	Crossover	Other method	Double	No	5	4
Uemura 2008	20 mg	w109	Gastro-oesophageal reflux	Parallel	Unspecified method	Double	Yes	93	95
Meineche-Schmidt 1997 (healthy)	20 mg	w140	Healthy volunteers	Parallel	Closed list	Double	No	278	270
Houben 1995	20 mg	w141	Healthy volunteers	Crossover	Unspecified method	Double	No	3	3
Ostovaneh 2014	20 mg	w142	Gastro-oesophageal reflux	Parallel	Closed list	Double	No	48	48
Aimi 2014	20 mg	w143	Insomnia	Parallel	Pharmacy	Double	No	89	86
Stephenson 2015	20 mg	w144	Laryngeal cancer	Parallel	Onsite computer	Double	No	21	19
Ren 2011	20 mg	w145	Percutaneous coronary intervention	Parallel	Unspecified method	Unknown	Yes	86	86
Dammann 1985 C	30 mg	w146	Healthy volunteers	Parallel	Unspecified method	Double	Yes	10	5
Hetzel 1988 A	30 mg	w121	Gastro-oesophageal reflux	Parallel	Unspecified method	Double	Yes	32	32
Maev 2002	40 mg	w147	Asthma	Parallel	Unspecified method	Unknown	No	64	53
Bolling-Sternevald 2002	40 mg	w148	Gastro-oesophageal reflux	Parallel	Closed list	Double	Yes	100	97
Laine 1998 C	40 mg	w149	Recently healed ulcers	Parallel	Unspecified method	Double	No	48	50
Meineche-Schmidt 2004	40 mg	w116	Gastro-oesophageal reflux	Parallel	Closed list	Double	No	283	272

Webtable 1 (cont.): Details of trials involving a randomized comparison of a proton pump inhibitor (X) versus control (Y)

Trial name	Regimen	Ref.	Indication for treatment	Design	Allocation Concealment	Blinding of investigators and patients	Availability of data	No. of patients NX	NY
Rabeneck 2002	40 mg	w150	Gastro-oesophageal reflux	Parallel	Onsite computer	Double	Yes	71	69
Kiljander 1999	40 mg	w151	Asthma	Crossover	Unspecified method	Double	No	27	25
Scheiman 1994	40 mg	w152	Healthy volunteers	Crossover	Unspecified method	Double	No	11	9
Achem 1997	40 mg	w153	Gastro-oesophageal reflux	Parallel	Onsite computer	Double	No	17	19
Caraco 1995	40 mg	w154	Healthy volunteers	Crossover	Unspecified method	Double	No	8	8
Hetzel 1988 B	40 mg	w121	Gastro-oesophageal reflux	Parallel	Unspecified method	Double	No	82	32
Bortolotti 2006	40 mg	w155	Gastro-oesophageal reflux	Parallel	Unspecified method	Double	No	10	10
Dorta 2000	40 mg	w156	Healthy volunteers	Crossover	Sealed envelopes	Double	Yes	6	6
Kiljander 2000	40 mg	w157	Gastro-oesophageal reflux	Crossover	Unspecified method	Double	No	14	14
Fallahi 2008	40 mg	w158	Gastro-oesophageal reflux	Parallel	Unspecified method	Double	No	18	18
Meier 1994	40 mg	w159	Asthma	Crossover	Unspecified method	Double	Yes	10	5
Sharma 1987	40 mg	w160	Healthy volunteers	Parallel	Unspecified method	Unknown	Yes	8	8
Teichtahl 1996	40 mg	w161	Gastro-oesophageal reflux	Crossover	Unspecified method	Double	No	12	12
Sontag 1992	40 mg	w132	Gastro-oesophageal reflux	Parallel	Unspecified method	Double	No	91	46
Rabeneck 2001	40 mg	w162	Gastro-oesophageal reflux	Parallel	Unspecified method	Double	No	-	-
Simon 1995 A	40 mg	w133	Healthy volunteers	Parallel	Unspecified method	Double	No	12	12
Unge 1989 B	40 mg	w163	Gastritis or duodenitis	Parallel	Unspecified method	Double	No	10	7
Watson 1997	40 mg	w164	Oesophageal disorders	Crossover	Unspecified method	Double	Yes	10	10
Serfaty-Lacrosniere 1995	40 mg	w165	Healthy volunteers	Parallel	Unspecified method	Unknown	No	8	5
Budzynski 2008	40 mg	w166	Cardiovascular disease	Crossover	Pharmacy	Double	No	24	24
Houben 1995	40 mg	w141	Healthy volunteers	Crossover	Unspecified method	Double	No	3	3
Chen 2013	40 mg	w167	Gastro-oesophageal reflux	Parallel	Unspecified method	Unknown	Yes	30	30
Emami 2014	40 mg	w168	Chronic cough	Crossover	Unspecified method	Double	No	23	22
Poh 2011	40 mg	w169	Gastro-oesophageal reflux	Parallel	Unspecified method	Unknown	No	31	16
Study 301	40 mg	w170	Cardiovascular disease	Parallel	Unspecified method	Double	Yes	265	265
Study 302	40 mg	w170	Cardiovascular disease	Parallel	Unspecified method	Double	Yes	259	260
Goldstein 2013	40 mg	w171	Antiplatelet therapy	Parallel	Unspecified method	Double	Yes	521	522
Cai 2010	40 mg	w172	Percutaneous coronary intervention	Parallel	Unspecified method	Unknown	Yes	20	20
Boeree 1998	80 mg	w173	Gastro-oesophageal reflux	Parallel	Unspecified method	Double	No	18	18
Noordzij 2001	80 mg	w174	Gastro-oesophageal reflux	Parallel	Unspecified method	Double	Yes	15	15
Pandak 2002	80 mg	w175	Gastro-oesophageal reflux	Crossover	Unspecified method	Double	Yes	22	20
Ours 1999	80 mg	w176	Chronic cough	Parallel	Unspecified method	Double	Yes	8	9
Freedberg 2015	80 mg	w177	Healthy volunteers	Crossover	Unspecified method	Unknown	No	6	6
Andersson 2014	80 mg	w41	Healthy volunteers	Crossover	Onsite computer	Unknown	Yes	27	27
Dabirmoghadam 2012	Unknown	w178	Gastro-oesophageal reflux	Parallel	Unspecified method	Unknown	No	30	30
Pantoprazole vs Control									
Richter 2000 A	10 mg	w179	Gastro-oesophageal reflux	Parallel	Unspecified method	Double	No	174	82
Pilotto 2003	20 mg	w180	Gastro-oesophageal reflux	Parallel	Unspecified method	Double	No	49	56
Richter 2000 A	20 mg	w179	Gastro-oesophageal reflux	Parallel	Unspecified method	Double	No	174	82
van Rensburg 2008	20 mg	w181	Gastro-oesophageal reflux	Parallel	Unspecified method	Double	Yes	207	212
Holtmann 2011	20 mg	w182	Antiplatelet therapy	Parallel	Unspecified method	Double	Yes	404	396
van der Velden 2010	20 mg	w183	Gastro-oesophageal reflux	Parallel	Closed list	Double	No	62	141
Bianchi Porro 2000	40 mg	w184	Arthritis, rheumatic or musculoskeletal disorders	Parallel	Other method	Double	Yes	70	34
Richter 2000 A	40 mg	w179	Gastro-oesophageal reflux	Parallel	Unspecified method	Double	No	173	82
Dammann 1994	40 mg	w185	Healthy volunteers	Crossover	Unspecified method	Double	No	6	8
Santos 2006	40 mg	w186	Asthma	Parallel	Unspecified method	Double	No	22	22
Dabos 2010	40 mg	w187	Gastritis or duodenitis	Parallel	Unspecified method	Unknown	No	13	13
Wo 2006	40 mg	w188	Gastro-oesophageal reflux	Parallel	Closed list	Double	Yes	20	19
Schwartz 2001	40 mg	w189	Gastro-oesophageal reflux	Parallel	Unspecified method	Double	No	10	10
Müller 1998	40 mg	w190	Healthy volunteers	Parallel	Unspecified method	Double	Yes	16	8
Suurna 2008	40 mg	w191	Gastro-oesophageal reflux	Crossover	Sealed envelopes	Double	No	30	30

Webtable 1 (cont.): Details of trials involving a randomized comparison of a proton pump inhibitor (X) versus control (Y)

Trial name	Regimen	Ref.	Indication for treatment	Design	Allocation Concealment	Blinding of investigators and patients	Availability of data	No. of patients NX	NY
Cervelli 2012	40 mg	w192	Haemodialysis	Crossover	Unspecified method	Double	No	13	13
Niklasson 2010	40 mg	w193	Healthy volunteers	Parallel	Closed list	Double	No	25	25
Baysal 2015	40 mg	w194	Gastro-oesophageal reflux	Parallel	Onsite computer	Double	Yes	132	132
Yu 2013 A	40 mg	w14	Cardiovascular disease	Parallel	Unspecified method	Unknown	No	100	148
Gonzalez-Ortiz 2015	40 mg	w195	Diabetes	Parallel	Unspecified method	Double	No	7	7
Lai 2012	40 mg	w196	Haemodialysis	Crossover	Unspecified method	Double	Yes	5	5
Yu 2013 B	40 mg	w197	Dual antiplatelet therapy	Parallel	Unspecified method	Unknown	No	35	35
Yang 2014	40 mg	w198	Gastro-oesophageal reflux	Parallel	Unspecified method	Unknown	Yes	75	74
Cai 2010	40 mg	w172	Percutaneous coronary intervention	Parallel	Unspecified method	Unknown	Yes	20	20
Zhao 2015	75 mg	w199	Percutaneous coronary intervention	Parallel	Unspecified method	Unknown	Yes	150	150
Eherer 2003	80 mg	w200	Gastro-oesophageal reflux	Crossover	Unspecified method	Double	Yes	10	10
Singh 2012	80 mg	w201	Diabetes	Parallel	Closed list	Double	Yes	16	15
Rabeprazole vs Control									
Japan Rabeprazole Study Group for NERD	5 mg	w202	Gastro-oesophageal reflux	Parallel	Unspecified method	Double	Yes	93	93
Caos 2005	10 mg	w203	Gastro-oesophageal reflux	Parallel	Onsite computer	Double	No	165	169
Caos 2000	10 mg	w204	Gastro-oesophageal reflux	Parallel	Unspecified method	Double	Yes	70	70
Cloud 1998 C	10 mg	w205	Gastro-oesophageal reflux	Parallel	Unspecified method	Double	No	27	25
Birbara 2000	10 mg	w206	Gastro-oesophageal reflux	Parallel	Unspecified method	Double	No	95	99
Miner 2006 B	10 mg	w207	Gastro-oesophageal reflux	Parallel	Unspecified method	Double	Yes	65	70
CAESAR	10 mg	w208	Gastro-oesophageal reflux	Parallel	Unspecified method	Double	No	49	49
Tanaka 2014	10 mg	w209	Osteoporosis	Parallel	Unspecified method	Unknown	No	49	47
Munesue 2012	10 mg	w210	Gastro-oesophageal reflux	Parallel	Unspecified method	Double	No	-	-
Hidaka 2012	10 mg	w211	Varices	Parallel	Onsite computer	Open	Yes	21	22
Itoh 2013	10 mg	w212	Osteoporosis	Parallel	Unspecified method	Unknown	Yes	94	86
SAMURAI	10 mg	w213	Gastro-oesophageal reflux	Parallel	Unspecified method	Double	Yes	84	85
Japan Rabeprazole Study Group for NERD	10 mg	w202	Gastro-oesophageal reflux	Parallel	Unspecified method	Double	Yes	102	93
Chen H 2010	20 mg	w214	Cholecystectomy	Parallel	Sealed envelopes	Unknown	No	61	59
Caos 2005	20 mg	w203	Gastro-oesophageal reflux	Parallel	Onsite computer	Double	No	163	169
Caos 2000	20 mg	w204	Gastro-oesophageal reflux	Parallel	Unspecified method	Double	Yes	69	70
Dammann 1999	20 mg	w215	Healthy volunteers	Crossover	Unspecified method	Double	Yes	6	6
Cloud 1998 C	20 mg	w205	Gastro-oesophageal reflux	Parallel	Unspecified method	Double	No	25	25
Birbara 2000	20 mg	w206	Gastro-oesophageal reflux	Parallel	Unspecified method	Double	No	94	99
Laheij 2003	20 mg	w216	Antiplatelet therapy	Parallel	Closed list	Double	Yes	76	74
Miner 2006 B	20 mg	w207	Gastro-oesophageal reflux	Parallel	Unspecified method	Double	Yes	68	70
van Rossum 2004	20 mg	w217	Antiplatelet therapy	Parallel	Closed list	Double	No	73	69
Santarelli 2003	20 mg	w218	Gastro-oesophageal reflux	Parallel	Closed list	Open	No	20	20
Johanson 2001	20 mg	w219	Gastro-oesophageal reflux	Parallel	Unspecified method	Double	No	59	58
Chan 2014 A	20 mg	w220	Arthritis, rheumatic or musculoskeletal disorders	Parallel	Closed list	Double	No	58	55
Munesue 2012	20 mg	w210	Gastro-oesophageal reflux	Parallel	Unspecified method	Double	No	-	-
SAMURAI	20 mg	w213	Gastro-oesophageal reflux	Parallel	Unspecified method	Double	Yes	85	85
Katare 2013	20 mg	w221	Gastritis or duodenitis	Parallel	Unspecified method	Unknown	No	30	30
Sun 2014	20 mg	w222	Gastro-oesophageal reflux	Parallel	Unspecified method	Unknown	No	30	30
Peterson 2008	30 mg	w223	Asthma	Parallel	Onsite computer	Double	No	23	8
Pawar 2007	40 mg	w224	Post nasal drip	Parallel	Unspecified method	Double	No	21	26
Cloud 1998 C	40 mg	w205	Gastro-oesophageal reflux	Parallel	Unspecified method	Double	No	26	25
Steward 2004	40 mg	w225	Gastro-oesophageal reflux	Parallel	Sealed envelopes	Double	Yes	21	21
Lam 2010	40 mg	w226	Gastro-oesophageal reflux	Parallel	Closed list	Double	No	43	43
Leelayuthachai 2012	40 mg	w227	Gastro-oesophageal reflux	Parallel	Unspecified method	Unknown	No	-	-
Munesue 2012	40 mg	w210	Gastro-oesophageal reflux	Parallel	Unspecified method	Double	No	-	-
SAMURAI	40 mg	w213	Gastro-oesophageal reflux	Parallel	Unspecified method	Double	Yes	84	85

Webtable 1 (cont.): Details of trials involving a randomized comparison of a proton pump inhibitor (X) versus control (Y)

Trial name	Regimen	Ref.	Indication for treatment	Design	Allocation Concealment	Blinding of investigators and patients	Availability of data	No. of patients NX	NY
Other PPI vs Control									
Johansson 2009	40-60 mg	w228	Oesophagectomy	Parallel	Sealed envelopes	Open	Yes	40	40
Holtmann 2013 (without herbal preparation)	Unknown	w229	Gastro-oesophageal reflux	Parallel	Unspecified method	Double	No	30	25
Holtmann 2013 (with herbal preparation)	Unknown	w229	Gastro-oesophageal reflux	Parallel	Unspecified method	Double	No	28	27

Healing Trials

Trial name	Regimen	Ref.	Indication for treatment	Design	Allocation Concealment	Blinding of investigators and patients	Availability of data	NX	NY
Esomeprazole vs Control									
Tulassay 2008	20 mg	w230	Peptic ulcers	Parallel	Closed list	Double	Yes	164	152
Lansoprazole vs Control									
Avner 1995 B	15 mg	w231	Peptic ulcers	Parallel	Unspecified method	Double	Yes	73	75
Avner 1995 A	15 mg	w232	Peptic ulcers	Parallel	Unspecified method	Double	Yes	64	60
Jhala 1995	15 mg	w233	Peptic ulcers	Parallel	Unspecified method	Double	Yes	34	19
Lanza 1994	15 mg	w234	Peptic ulcers	Parallel	Unspecified method	Double	Yes	87	44
Avner 1995 B	30 mg	w231	Peptic ulcers	Parallel	Unspecified method	Double	Yes	75	75
Avner 1995 A	30 mg	w232	Peptic ulcers	Parallel	Unspecified method	Double	Yes	62	60
Jhala 1995	30 mg	w233	Peptic ulcers	Parallel	Unspecified method	Double	Yes	33	19
Lanza 1994	30 mg	w234	Peptic ulcers	Parallel	Unspecified method	Double	Yes	84	44
Fu 2012	30 mg	w235	Peptic ulcers	Parallel	Unspecified method	Unknown	Yes	56	56
Avner 1995 B	60 mg	w231	Peptic ulcers	Parallel	Unspecified method	Double	Yes	73	75
Avner 1995 A	60 mg	w232	Peptic ulcers	Parallel	Unspecified method	Double	Yes	59	60
Harford 1996	60 mg	w236	Peptic ulcers	Parallel	Unspecified method	Double	Yes	68	67
Harford 1996	90 mg	w236	Peptic ulcers	Parallel	Unspecified method	Double	Yes	72	67
Omeprazole vs Control									
Hsu 2003	20 mg	w237	Peptic ulcers	Parallel	Unspecified method	Open	Yes	40	39
D'Imperio 1994	20 mg	w238	Post-endoscopic ulcers	Parallel	Unspecified method	Open	No	73	73
Jung 2008	20 mg	w239	Peptic ulcers	Parallel	Unspecified method	Open	Yes	14	12
Dotto 1993	20 mg	w240	Peptic ulcers	Parallel	Unspecified method	Unknown	Yes	12	10
Valenzuela 1996	20 mg	w241	Peptic ulcers	Parallel	Unspecified method	Double	Yes	202	104
Sezer 1995 (with ciprofloxacin)	20 mg	w242	Peptic ulcers	Parallel	Unspecified method	Double	Yes	24	21
Sezer 1995 (no ciprofloxacin)	20 mg	w242	Peptic ulcers	Parallel	Unspecified method	Double	Yes	23	24
Graham 1990	20 mg	w243	Peptic ulcers	Parallel	Unspecified method	Double	Yes	102	51
Hepylog Investigator Study Group	20 mg	w244	Peptic ulcers	Parallel	Sealed envelopes	Double	Yes	76	74
Marzio 2003	20 mg	w245	Peptic ulcers	Parallel	Unspecified method	Double	Yes	50	53
Tepeš 2001	20 mg	w246	Peptic ulcers	Parallel	Unspecified method	Double	Yes	38	39
Labenz 1997 C	20 mg	w247	Peptic ulcers	Parallel	Unspecified method	Double	Yes	21	20
Laine 1998 B	40 mg	w149	Peptic ulcers	Parallel	Unspecified method	Double	Yes	62	59
Valenzuela 1996	40 mg	w241	Peptic ulcers	Parallel	Unspecified method	Double	Yes	214	104
Uygurer 1998	40 mg	w248	Peptic ulcers	Parallel	Unspecified method	Unknown	Yes	25	25
Zafar 2012	40 mg	w249	Peptic ulcers	Parallel	Unspecified method	Unknown	No	-	-
Laine 1998 A	80 mg	w149	Peptic ulcers	Parallel	Unspecified method	Double	Yes	72	37
Neil 1998 A	80 mg	w250	Peptic ulcers	Parallel	Unspecified method	Double	No	36	13
Pantoprazole vs Control									
Bago 1998	80 mg	w251	Peptic ulcers	Parallel	Unspecified method	Unknown	Yes	44	50
Rabeprazole vs Control									
Takeuchi 2012	10 mg	w252	Peptic ulcers	Parallel	Closed list	Open	Yes	40	38
Cloud 1998 A	20 mg	w253	Peptic ulcers	Parallel	Unspecified method	Double	Yes	32	31
Cloud 1998 B	20 mg	w253	Peptic ulcers	Parallel	Unspecified method	Double	Yes	34	33
Cloud 1998 A	40 mg	w253	Peptic ulcers	Parallel	Unspecified method	Double	Yes	31	31
Cloud 1998 B	40 mg	w253	Peptic ulcers	Parallel	Unspecified method	Double	Yes	33	33

Webtable 1 (cont.): Details of trials involving a randomized comparison of a proton pump inhibitor (X) versus control (Y)

Trial name	Regimen	Ref.	Indication for treatment	Design	Allocation Concealment	Blinding of investigators and patients	Availability of data	No. of patients NX	NY
**Acute Bleeding Trials**									
Esomeprazole vs Control									
Peptic Ulcer Bleed Study	192 mg	w254	Bleeding Peptic Ulcer	Parallel	Onsite computer	Double	Yes	376	391
Lansoprazole vs Control									
Hawkey 2001	120 mg	w255	UGI bleeding of any cause	Factorial	Unspecified method	Double	Yes	102	103
Omeprazole vs Control									
Khuroo 1997	80 mg	w256	Bleeding Peptic Ulcer	Parallel	Sealed envelopes	Double	Yes	110	110
Javid 2001	80 mg	w257	Bleeding Peptic Ulcer	Parallel	Sealed envelopes	Double	Yes	82	84
Sofia 2000	120 mg	w258	Bleeding Peptic Ulcer	Parallel	Other method	Open	Yes	40	44
Lau 2000	192 mg	w259	Bleeding Peptic Ulcer	Parallel	Pharmacy	Single	Yes	120	120
Hasselgren 1997	192 mg	w260	Bleeding Peptic Ulcer	Parallel	Unspecified method	Double	Yes	159	163
Lau 2007	192 mg	w261	UGI bleeding of any cause	Parallel	Pharmacy	Double	Yes	319	319
Schaffalitzky de Muckadell 1997	192 mg	w262	Bleeding Peptic Ulcer	Parallel	Onsite computer	Double	Yes	134	140
Daneshmend 1992	200 mg	w263	UGI bleeding of any cause	Parallel	Unspecified method	Double	Yes	578	569
Pantoprazole vs Control									
Zargar 2006	192 mg	w264	Bleeding Peptic Ulcer	Parallel	Sealed envelopes	Double	Yes	102	101
Hung 2007	192 mg	w265	Bleeding Peptic Ulcer	Parallel	Closed list	Open	Yes	56	54
Hung 2007	197 mg	w265	Bleeding Peptic Ulcer	Parallel	Closed list	Open	Yes	56	54

Design: Parallel=parallel trial. Factorial=Factorial trial. ‘Extension’ studies are studies in which patients are randomised more than once during the trial (only the parts of the trial that involve a valid randomised comparison for this meta-analysis, labelled X1, X2 etc., are shown in this table). Data shown for crossover trials reflect only events during the first phase of the trial. The suffixes A, B, C, D are used to distinguish different trials published by the same author in the same year.

Allocation concealment: IVRS (Interactive Voice Response System); Telephone (telephone central office); Pharmacy (pharmacy coded containers administered sequentially); Onsite computer (onsite computer accessible only after entering patient details); Sealed envelopes (sequentially numbered, opaque or without further specification); Closed list (closed list of random numbers); Other (other specified method); Unspecified (stated as being ‘randomised’ but with no other details).

Blinding of investigators and patients: Single=single-blind; Double=double-blind; Open=open label.

Availability of data: Data available for at least one endpoint of interest in the meta-analyses.

In cases where the number randomised was not reported, the number evaluated was used instead. In addition, for crossover trials, where neither the number randomised nor the number evaluated was reported, the total number of participants divided by number of arms was used.

Webtable 2: Details of trials involving a randomised comparison of a prostaglandin analogue (X) versus control (Y)

Trial name	Regimen	Ref.	Indication for treatment	Design	Allocation Concealment	Blinding of investigators and patients	Availability of data	No. of patients NX	NY
**Prevention Trials**									
Arbaprostil vs Control									
Reele 1982	400 ug	w266	Healthy volunteers	Parallel	Unspecified method	Double	No	6	6
Enprostil vs Control									
Lanza 1990	70 ug	w267	Healthy volunteers	Parallel	Closed list	Double	Yes	33	34
Davis 1989	105 ug	w268	Diabetes	Crossover	Unspecified method	Double	No	6	6
Lanza 1990	140 ug	w267	Healthy volunteers	Parallel	Closed list	Double	Yes	36	34
Misoprostol vs Control									
Santillan 1999	50 ug	w269	Antiplatelet therapy	Parallel	Unspecified method	Double	Yes	19	19
Donnelly 2000	100 ug	w270	Antiplatelet therapy	Parallel	Unspecified method	Double	Yes	16	16
Delmas 1994	400 ug	w271	Arthritis, rheumatic or musculoskeletal disorders	Parallel	Unspecified method	Double	Yes	73	103
Graham 1988	400 ug	w272	Osteoarthritis	Parallel	Unspecified method	Double	Yes	143	138
Spaggiari 1993	400 ug	w273	Post-operative	Parallel	Unspecified method	Open	No	50	45
OMNIUM 1998 X2	400 ug	w113	Recently healed ulcers	Extension	Unspecified method	Double	Yes	296	155
Bocanegra 1998	400 ug	w274	Osteoarthritis	Parallel	Unspecified method	Double	Yes	175	154
Van Beusekom 1995	400 ug	w275	Rheumatoid arthritis	Parallel	Unspecified method	Double	No	38	38
Munger 2008	400 ug	w276	Hypertension	Crossover	Unspecified method	Double	Yes	10	10
Ahmed 1991 B	400 ug	w277	Antiplatelet therapy	Parallel	Unspecified method	Double	No	18	19
Goldin 1991	400 ug	w278	Recently healed ulcers	Parallel	Closed list	Double	Yes	25	12
Bach 1991	400 ug	w279	Arthritis, rheumatic or musculoskeletal disorders	Parallel	Unspecified method	Double	Yes	68	70
Curtis 1993	400 ug	w280	Renal transplant	Parallel	Unspecified method	Double	No	58	100
Raskin 1995	400 ug	w281	Arthritis, rheumatic or musculoskeletal disorders	Parallel	Sealed envelopes	Double	Yes	344	308
Pazzi 1994	400 ug	w282	Gastritis or duodenitis	Parallel	Unspecified method	Double	Yes	22	26
De Melo Gomes 1992 A	455 ug	w283	Osteoarthritis	Parallel	Unspecified method	Double	Yes	178	183
Doherty 1992	460 ug	w284	Osteoarthritis	Parallel	Unspecified method	Double	No	228	227
Agrawal 1995	500 ug	w285	Arthritis, rheumatic or musculoskeletal disorders	Parallel	Unspecified method	Unknown	Yes	193	191
De Melo Gomes 1992 B	500 ug	w283	Rheumatoid arthritis	Parallel	Unspecified method	Double	Yes	164	175
Hannequin 1992	500 mg	w286	Rheumatoid arthritis	Parallel	Unspecified method	Double	No	177	169
Akkuzu 2004	540 ug	w287	Tinnitis	Parallel	Unspecified method	Double	No	28	14
Zuinen 1993	541 ug	w288	Arthritis, rheumatic or musculoskeletal disorders	Parallel	Unspecified method	Double	No	185	187
Chandrasekaran 1991	600 ug	w289	Arthritis, rheumatic or musculoskeletal disorders	Parallel	Unspecified method	Double	Yes	45	45
Henriksson 1993	600 ug	w290	Rheumatoid arthritis	Parallel	Unspecified method	Double	Yes	20	20
Spaziani 1994	600 mg	w291	Gastritis or duodenitis	Parallel	Unspecified method	Unknown	No	16	14
Bardhan 1993	600 ug	w292	Arthritis, rheumatic or musculoskeletal disorders	Parallel	Unspecified method	Double	Yes	144	157
Boers 1991	600 ug	w293	Rheumatoid arthritis	Crossover	Unspecified method	Double	No	12	12
Briner 1993	600 ug	w294	Tinnitis	Parallel	Unspecified method	Unknown	Yes	12	12
Fujimori 2009	600 ug	w295	Antiplatelet therapy	Parallel	Unspecified method	Single	Yes	16	16
Kelly 1994	600 ug	w296	Antiplatelet therapy	Parallel	Unspecified method	Double	Yes	5	10
Evers 1998	600 ug	w297	Chronic cluster headache	Crossover	Unspecified method	Double	Yes	4	4
Raskin 1995	600 ug	w281	Arthritis, rheumatic or musculoskeletal disorders	Parallel	Sealed envelopes	Double	Yes	332	308
Levi 1992	600 ug	w298	Arthritis, rheumatic or musculoskeletal disorders	Parallel	Unspecified method	Unknown	Yes	10	10
Elliott 1994	625 ug	w299	Arthritis, rheumatic or musculoskeletal disorders	Parallel	Unspecified method	Double	Yes	40	43
Duenas-Gonzalez 1995	750 ug	w300	Cancer or palliative care (non gastrointestinal)	Parallel	Unspecified method	Double	No	9	7
Roth 1993	800 ug	w301	Osteoarthritis	Parallel	Unspecified method	Unknown	Yes	60	53
Delmas 1994	800 ug	w271	Arthritis, rheumatic or musculoskeletal disorders	Parallel	Unspecified method	Double	Yes	80	103
Graham 1993	800 ug	w302	Arthritis, rheumatic or musculoskeletal disorders	Parallel	Unspecified method	Double	Yes	320	323
Graham 2002	800 ug	w51	Recently healed ulcers	Parallel	Other method	Double	Yes	134	134

Webtable 2 (cont.): Details of trials involving a randomised comparison of a prostaglandin analogue (X) versus control (Y)

Trial name	Regimen	Ref.	Indication for treatment	Design	Allocation Concealment	Blinding of investigators and patients	Availability of data	No. of patients NX	NY
Graham 1988	800 ug	w272	Osteoarthritis	Parallel	Unspecified method	Double	Yes	140	138
MUCOSA	800 ug	w303	Rheumatoid arthritis	Parallel	Pharmacy	Double	Yes	4404	4439
Shiokawa 1991	800 ug	w304	Rheumatoid arthritis	Parallel	Unspecified method	Double	Yes	133	130
Saggioro 1991	800 ug	w305	Arthritis, rheumatic or musculoskeletal disorders	Parallel	Unspecified method	Double	Yes	82	84
Fullerton 1993	800 ug	w306	Osteoarthritis	Crossover	Closed list	Double	No	6	6
Bolten 1989	800 ug	w307	Rheumatoid arthritis	Parallel	Unspecified method	Double	Yes	31	36
Bennett 1991	800 ug	w308	Healthy volunteers	Crossover	Unspecified method	Double	No	6	6
Hilbrands 1996	800 ug	w309	Renal transplant	Parallel	Unspecified method	Double	Yes	22	18
Goodwin 1987	800 ug	w310	Healthy volunteers	Parallel	Unspecified method	Double	No	25	25
Curtis 1993	800 ug	w280	Renal transplant	Parallel	Unspecified method	Double	No	99	100
Moran 1990	800 ug	w311	Renal transplant	Parallel	Other method	Double	No	38	39
Weinblatt 1994	800 ug	w312	Rheumatoid arthritis	Parallel	Unspecified method	Double	No	23	27
Raskin 1995	800 ug	w281	Arthritis, rheumatic or musculoskeletal disorders	Parallel	Sealed envelopes	Double	Yes	148	308
Mavligit 1987	800 ug	w313	Colorectal cancer	Parallel	Unspecified method	Unknown	Yes	11	10
Kim 1996	800 ug	w314	Renal transplant	Parallel	Unspecified method	Unknown	No	10	10
Hausken 1990	800 ug	w315	Gastro-oesophageal reflux	Parallel	Unspecified method	Double	No	68	69
Yilmaz 2004	800 ug	w316	Tinnitis	Parallel	Unspecified method	Double	No	28	12
Wasiak 1999	1200 ug	w317	Asthma	Crossover	Unspecified method	Double	No	8	8
Rebamipide vs Control									
Nishida 2011	100 mg	w318	Healthy volunteers	Crossover	Closed list	Double	Yes	5	5
Han 2015	300 mg	w319	Gastritis or duodenitis	Parallel	Unspecified method	Unknown	No	89	88
Mizukami 2012	300 mg	w320	Healthy volunteers	Crossover	Unspecified method	Double	No	6	6
Tozawa 2014 (ASA)	300 mg	w321	Antiplatelet therapy	Parallel	Unspecified method	Double	Yes	8	8
Tozawa 2014 (ASA+clopidogrel)	300 mg	w321	Dual antiplatelet therapy	Parallel	Unspecified method	Double	Yes	8	8
Fujimori 2011	300 mg	w322	Healthy volunteers	Parallel	Unspecified method	Double	No	34	38
Kato 2011 A	300 mg	w323	Healthy volunteers	Crossover	Unspecified method	Double	Yes	5	5
Kamada 2014	300 mg	w324	Gastritis or duodenitis	Parallel	Unspecified method	Open	No	82	87
Kurokawa 2014	300 mg	w325	Antiplatelet therapy	Parallel	Closed list	Double	Yes	31	31
Horiki 2012	300 mg	w326	Arthritis, rheumatic or musculoskeletal disorders	Parallel	Unspecified method	Open	Yes	31	34
Adachi 2012	300 mg	w327	Gastro-oesophageal reflux	Parallel	Onsite computer	Double	No	31	29
Abou-Raya 2013	300 mg	w328	Osteoarthritis	Parallel	Unspecified method	Unknown	No	178	89
Watanabe 2015	900 mg	w329	Antiplatelet therapy	Parallel	Unspecified method	Double	Yes	29	14
Rioprostil vs Control									
Paladini 1989	400 ug	w330	Antiplatelet therapy	Parallel	Unspecified method	Double	Yes	11	9
Paladini 1989	450 ug	w330	Antiplatelet therapy	Parallel	Unspecified method	Double	Yes	10	9
Purushotham 1991	600 ug	w331	Gastro-oesophageal reflux	Parallel	Unspecified method	Double	Yes	14	12

Healing Trials

Trial name	Regimen	Ref.	Indication for treatment	Design	Allocation Concealment	Blinding of investigators and patients	Availability of data	NX	NY
Arbaprostil vs Control									
Karim 1976 B	4 mg	w332	Peptic ulcers	Parallel	Unspecified method	Double	Yes	10	10
Gibinski 1977 (gastric ulcer)	4.5 ug/kg	w333	Peptic ulcers	Parallel	Unspecified method	Double	Yes	12	7
Gibinski 1977 (duodenal ulcer)	4.5 ug/kg	w333	Peptic ulcers	Parallel	Unspecified method	Double	Yes	39	19
Jaszewski 1988	40 ug	w334	Peptic ulcers	Parallel	Unspecified method	Double	Yes	10	15
Euler 1989 B	40 ug	w335	Peptic ulcers	Parallel	Unspecified method	Double	Yes	64	60
Euler 1987	40 ug	w336	Peptic ulcers	Parallel	Sealed envelopes	Double	Yes	40	44
Euler 1990 A	40 ug	w337	Peptic ulcers	Parallel	Unspecified method	Double	Yes	8	24
Euler 1990 C	40 ug	w337	Peptic ulcers	Parallel	Unspecified method	Double	Yes	32	34
Jaszewski 1987	40 ug	w338	Peptic ulcers	Parallel	Unspecified method	Double	No	15	16
Euler 1989 A	50 ug	w339	Peptic ulcers	Parallel	Unspecified method	Double	Yes	23	20
Euler 1990 D	100 ug	w340	Peptic ulcers	Parallel	Unspecified method	Double	Yes	211	207

Webtable 2 (cont.): Details of trials involving a randomised comparison of a prostaglandin analogue (X) versus control (Y)

Trial name	Regimen	Ref.	Indication for treatment	Design	Allocation Concealment	Blinding of investigators and patients	Availability of data	No. of patients NX	NY
Euler 1989 A	100 ug	w339	Peptic ulcers	Parallel	Unspecified method	Double	Yes	21	20
Euler 1990 B	100 ug	w337	Peptic ulcers	Parallel	Unspecified method	Double	Yes	31	30
Euler 1990 C	100 ug	w337	Peptic ulcers	Parallel	Unspecified method	Double	Yes	28	34
Euler 1990 D	200 ug	w340	Peptic ulcers	Parallel	Unspecified method	Double	Yes	212	207
Euler 1990 C	200 ug	w337	Peptic ulcers	Parallel	Unspecified method	Double	Yes	23	34
Chen 1980 B	300 ug	w341	Peptic ulcers	Parallel	Unspecified method	Double	Yes	17	16
Vantrappen 1982	400 ug	w342	Peptic ulcers	Parallel	Unspecified method	Double	Yes	89	92
Karim 1976 A	600 ug	w332	Peptic ulcers	Parallel	Unspecified method	Double	Yes	10	10
Enprostil vs Control									
Dal Monte 1988	70 ug	w343	Peptic ulcers	Parallel	Unspecified method	Double	Yes	21	21
Schubert 1989	70 ug	w344	Peptic ulcers	Parallel	Other method	Double	Yes	63	64
Bright-Asare 1986 A	70 ug	w345	Peptic ulcers	Parallel	Unspecified method	Double	Yes	43	44
Navert 1986	70 ug	w346	Peptic ulcers	Parallel	Unspecified method	Double	Yes	44	42
Thomson 1986	70 ug	w347	Peptic ulcers	Parallel	Unspecified method	Double	Yes	40	36
Sontag 1994	70 ug	w348	Peptic ulcers	Parallel	Closed list	Double	No	37	43
Sontag 1994	105 ug	w348	Peptic ulcers	Parallel	Closed list	Double	No	39	43
Navert 1986	140 ug	w346	Peptic ulcers	Parallel	Unspecified method	Double	Yes	43	42
Thomson 1986	140 ug	w347	Peptic ulcers	Parallel	Unspecified method	Double	Yes	36	36
Misoprostol vs Control									
Agrawal 1985	100 ug	w349	Peptic ulcers	Parallel	Unspecified method	Double	Yes	107	103
Lam 1986	200 ug	w350	Peptic ulcers	Parallel	Unspecified method	Double	No	77	76
Brand 1985	200 ug	w351	Peptic ulcers	Parallel	Unspecified method	Double	Yes	101	100
Lam 1986	300 ug	w350	Peptic ulcers	Parallel	Unspecified method	Double	No	76	76
Agrawal 1985	400 ug	w349	Peptic ulcers	Parallel	Unspecified method	Double	Yes	94	103
Bright-Asare 1986 B	400 ug	w352	Peptic ulcers	Parallel	Unspecified method	Double	Yes	118	111
Sontag 1985	400 ug	w353	Peptic ulcers	Parallel	Unspecified method	Double	Yes	142	144
Mazure 1985	400 ug	w354	Peptic ulcers	Parallel	Pharmacy	Double	Yes	16	19
Brand 1985	800 ug	w351	Peptic ulcers	Parallel	Unspecified method	Double	Yes	107	100
Ahmed 1991 A	800 ug	w355	Peptic ulcers	Parallel	Unspecified method	Double	Yes	25	25
Bright-Asare 1986 B	800 ug	w352	Peptic ulcers	Parallel	Unspecified method	Double	Yes	101	111
Jaszewski 1992	800 ug	w356	Peptic ulcers	Parallel	Unspecified method	Double	Yes	77	85
Newman 1987	800 ug	w357	Peptic ulcers	Parallel	Unspecified method	Double	Yes	117	108
Roth 1989	800 ug	w358	Peptic ulcers	Parallel	Unspecified method	Double	Yes	123	116
Barakat 1984 B	Unknown	w359	Peptic ulcers	Parallel	Unspecified method	Double	Yes	35	16
Rebamipide vs Control									
Terano 2007	300 mg	w360	Peptic ulcers	Parallel	Unspecified method	Double	Yes	157	152
Hahm 1998	300 mg	w361	Peptic ulcers	Parallel	Unspecified method	Unknown	Yes	36	21
Fujioka 2003	300 mg	w362	Peptic ulcers	Parallel	Unspecified method	Double	Yes	104	102
Sawaguchi 2011	300 mg	w363	Endoscopic submucosal dissection	Parallel	Unspecified method	Unknown	No	30	30
Kato 2010 A	300 mg	w364	Endoscopic submucosal dissection	Parallel	Other method	Unknown	Yes	31	31
Fujiwara 2011	300 mg	w365	Endoscopic submucosal dissection	Parallel	Closed list	Open	Yes	30	34
Nebiki 1998	900 mg	w366	Peptic ulcers	Parallel	Sealed envelopes	Unknown	Yes	60	60
Higuchi 1998	900 mg	w367	Peptic ulcers	Parallel	Unspecified method	Unknown	Yes	20	20
Rioprostil vs Control									
Caldwell 1989	400 ug	w368	Peptic ulcers	Parallel	Unspecified method	Double	Yes	30	26
Boucekkine 1989	600 ug	w369	Peptic ulcers	Parallel	Sealed envelopes	Double	Yes	57	58
Benvestito 1988	600 ug	w370	Peptic ulcers	Parallel	Unspecified method	Double	Yes	16	16
Caldwell 1989	1200 ug	w368	Peptic ulcers	Parallel	Unspecified method	Double	Yes	26	26

Webtable 2 (cont.): Details of trials involving a randomised comparison of a prostaglandin analogue (X) versus control (Y)

Trial name	Regimen	Ref.	Indication for treatment	Design	Allocation Concealment	Blinding of investigators and patients	Availability of data	No. of patients NX	NY
Other PA vs Control									
Chen 1980 B	1.5 mg	w341	Peptic ulcers	Parallel	Unspecified method	Double	Yes	19	16

Design: Parallel=parallel trial. Factorial=Factorial trial. ‘Extension’ studies are studies in which patients are randomised more than once during the trial (only the parts of the trial that involve a valid randomised comparison for this meta-analysis, labelled X1, X2 etc., are shown in this table). Data shown for crossover trials reflect only events during the first phase of the trial. The suffixes A, B, C, D are used to distinguish different trials published by the same author in the same year.

Allocation concealment: IVRS (Interactive Voice Response System); Telephone (telephone central office); Pharmacy (pharmacy coded containers administered sequentially); Onsite computer (onsite computer accessible only after entering patient details); Sealed envelopes (sequentially numbered, opaque or without further specification); Closed list (closed list of random numbers); Other (other specified method); Unspecified (stated as being ‘randomised’ but with no other details).

Blinding of investigators and patients: Single=single-blind; Double=double-blind; Open=open label.

Availability of data: Data available for at least one endpoint of interest in the meta-analyses.

In cases where the number randomised was not reported, the number evaluated was used instead. In addition, for crossover trials, where neither the number randomised nor the number evaluated was reported, the total number of participants divided by number of arms was used.

Webtable 3: Details of trials involving a randomised comparison of a histamine-2 receptor antagonists (X) versus control (Y)

Trial name	Regimen	Ref.	Indication for treatment	Design	Allocation Concealment	Blinding of investigators and patients	Availability of data	No. of patients NX	No. of patients NY
**Prevention Trials**									
Cimetidine vs Control									
Yilmaz 1996	32.5 mg/kg	w371	Warts	Parallel	Unspecified method	Double	No	35	35
Bergstrand 1985	100 mg	w372	Asthma	Parallel	Unspecified method	Double	No	13	7
Hui 1992	200 mg	w373	Recently healed ulcers	Parallel	Unspecified method	Single	Yes	98	103
Furman 1982	300 mg	w374	Gastro-oesophageal reflux	Parallel	Unspecified method	Double	No	-	-
Kibblewhite 1990	300 mg	w375	Post nasal drip	Parallel	Unspecified method	Double	No	9	11
Welch 1978	300 mg	w376	Antiplatelet therapy	Crossover	Unspecified method	Double	No	13	13
Roth 1987 X2	400 mg	w377	Arthritis, rheumatic or musculoskeletal disorders	Extension	Unspecified method	Double	Yes	12	14
Wu 2008	400 mg	w378	Urticaria	Parallel	Unspecified method	Open	No	33	34
Sonnenberg 1979 X2	400 mg	w379	Recently healed ulcers	Extension	Unspecified method	Double	Yes	24	23
Kokron 1993	400 mg	w380	Renal cell cancer	Parallel	Unspecified method	Unknown	Yes	25	19
Lööf 1987	400 mg	w381	Recently healed ulcers	Parallel	Unspecified method	Double	Yes	49	50
Becker 1984	400 mg	w382	Recently healed ulcers	Parallel	Unspecified method	Double	Yes	30	26
Sontag 1984	400 mg	w383	Recently healed ulcers	Parallel	Unspecified method	Double	Yes	76	81
Sontag 1984	400 mg	w383	Recently healed ulcers	Parallel	Unspecified method	Double	Yes	91	81
Kinloch 1984	400 mg	w384	Recently healed ulcers	Parallel	Unspecified method	Double	Yes	24	27
Visconti 1983	400 mg	w385	Recently healed ulcers	Parallel	Unspecified method	Unknown	Yes	20	20
Moshal 1982	400 mg	w386	Recently healed ulcers	Parallel	Unspecified method	Double	Yes	20	20
Bardhan 1988 A	400 mg	w387	Recently healed ulcers	Parallel	Unspecified method	Double	Yes	64	62
Eichenberger 1982 X2	400 mg	w388	Recently healed ulcers	Extension	Unspecified method	Double	Yes	13	12
Ubilluz 1982	400 mg	w389	Recently healed ulcers	Parallel	Unspecified method	Double	Yes	15	15
Sontag 1981	400 mg	w390	Recently healed ulcers	Parallel	Unspecified method	Double	Yes	46	49
Sontag 1981	400 mg	w390	Recently healed ulcers	Parallel	Unspecified method	Double	Yes	68	49
Da Silva 1981	400 mg	w391	Recently healed ulcers	Parallel	Unspecified method	Double	Yes	9	7
Salera 1979	400 mg	w392	Recently healed ulcers	Parallel	Unspecified method	Double	Yes	67	66
Cocco 1981	400 mg	w393	Recently healed ulcers	Parallel	Unspecified method	Unknown	No	-	-
Bianchi Porro 1979	400 mg	w394	Recently healed ulcers	Parallel	Unspecified method	Double	Yes	25	25
Massarrat 1982	400 mg	w395	Recently healed ulcers	Parallel	Unspecified method	Unknown	Yes	29	14
Massarrat 1982	400 mg	w395	Recently healed ulcers	Parallel	Unspecified method	Unknown	Yes	23	14
Dew 1984	400 mg	w396	Recently healed ulcers	Parallel	Onsite computer	Double	Yes	22	22
Fitzpatrick 1982	400 mg	w397	Recently healed ulcers	Parallel	Unspecified method	Double	Yes	19	17
Berstad 1979 B	400 mg	w398	Recently healed ulcers	Parallel	Unspecified method	Double	Yes	20	24
Broor 1983	400 mg	w399	Recently healed ulcers	Parallel	Unspecified method	Double	Yes	15	15
Hentschel 1983	400 mg	w400	Recently healed ulcers	Parallel	Unspecified method	Double	Yes	54	54
Hui 1992	400 mg	w373	Recently healed ulcers	Parallel	Unspecified method	Single	Yes	94	103
Piper 1986	400 mg	w401	Recently healed ulcers	Parallel	Unspecified method	Double	Yes	88	82
Pym 1990 (gastric ulcer)	400 mg	w402	Recently healed ulcers	Parallel	Other method	Double	Yes	97	87
Pym 1990 (duodenal ulcer)	400 mg	w402	Recently healed ulcers	Parallel	Other method	Double	Yes	60	67
Sonnenberg 1981 X2	400 mg	w403	Recently healed ulcers	Extension	Closed list	Double	Yes	34	32
Marks 1989	400 mg	w404	Recently healed ulcers	Parallel	Unspecified method	Single	Yes	39	39
Murray 1991	400 mg	w405	Gastro-oesophageal reflux	Parallel	Unspecified method	Double	No	20	17
Kaul 1986	400 mg	w406	Gastro-oesophageal reflux	Parallel	Unspecified method	Double	Yes	14	10
Kumar 1990	400 mg	w407	Recently healed ulcers	Parallel	Unspecified method	Double	Yes	50	50
Salim 1993 A	400 mg	w408	Recently healed ulcers	Parallel	Unspecified method	Double	Yes	75	76
Salim 1993 B	400 mg	w409	Recently healed ulcers	Parallel	Sealed envelopes	Unknown	Yes	37	35
Young 1989 B	400 mg	w410	Recently healed ulcers	Parallel	Unspecified method	Unknown	Yes	45	43
Takemoto 1987 (healed gastric ulcer)	400 mg	w411	Recently healed ulcers	Parallel	Unspecified method	Double	Yes	48	39
Takemoto 1987 (healed duodenal ulcer)	400 mg	w411	Recently healed ulcers	Parallel	Unspecified method	Double	Yes	36	35
Rohner 1985	400 mg	w412	Recently healed ulcers	Parallel	Unspecified method	Double	No	-	-
Burland 1980	400 mg	w413	Recently healed ulcers	Parallel	Unspecified method	Double	Yes	179	333
Gray 1978 (completely healed ulcers)	400 mg	w414	Recently healed ulcers	Parallel	Unspecified method	Double	Yes	27	27

Webtable 3 (cont.): Details of trials involving a randomised comparison of a histamine-2 receptor antagonists (X) versus control (Y)

Trial name	Regimen	Ref.	Indication for treatment	Design	Allocation Concealment	Blinding of investigators and patients	Availability of data	No. of patients NX	NY
Salim 1990	400 mg	w415	Recently healed ulcers	Parallel	Sealed envelopes	Unknown	Yes	77	74
Jean 1985 (healed duodenal ulcer)	600 mg	w416	Recently healed ulcers	Parallel	Unspecified method	Unknown	Yes	25	24
Jean 1985 (healed gastric ulcer)	600 mg	w416	Recently healed ulcers	Parallel	Unspecified method	Double	Yes	11	9
Sontag 1984	600 mg	w383	Recently healed ulcers	Parallel	Unspecified method	Double	Yes	172	81
Leão 1980	600 mg	w417	Recently healed ulcers	Parallel	Unspecified method	Double	Yes	6	3
Sontag 1981	600 mg	w390	Recently healed ulcers	Parallel	Unspecified method	Double	Yes	115	49
Aubia 1980	600 mg	w418	Pruritis	Parallel	Unspecified method	Unknown	No	6	7
Lai 1982	600 mg	w419	End stage renal disease	Parallel	Unspecified method	Open	No	9	6
Paoluzi 1985	600 mg	w420	Recently healed ulcers	Parallel	Unspecified method	Double	Yes	30	26
Libeskind 1983	600 mg	w421	Recently healed ulcers	Parallel	Unspecified method	Single	Yes	35	31
Stoa-Birketvedt 1993	600 mg	w422	Weight loss	Parallel	Unspecified method	Double	No	30	30
Rasmussen 1993	600 mg	w423	Weight loss	Parallel	Unspecified method	Double	No	30	30
Langman 1999	600 mg	w424	Gastric cancer	Parallel	IVRS	Double	Yes	218	224
Huang 2007	800 mg	w425	Rectal cancer	Parallel	Unspecified method	Open	No	30	30
Macphee 1984	800 mg	w426	Healthy volunteers	Crossover	Unspecified method	Unknown	No	4	4
Thilagarajah 2001	800 mg	w427	Painful bladder disease	Parallel	Unspecified method	Double	No	17	17
La Brooy 1980	800 mg	w428	Recently healed ulcers	Parallel	Unspecified method	Double	Yes	15	14
Farup 1990	800 mg	w429	Gastro-oesophageal reflux	Parallel	Unspecified method	Double	Yes	29	29
Danielsson 1981 X2	800 mg	w430	Recently healed ulcers	Extension	Unspecified method	Double	Yes	23	25
Frank 1989 C	800 mg	w431	Healthy volunteers	Parallel	Unspecified method	Double	Yes	18	21
Frank 1989 C	800 mg	w431	Healthy volunteers	Parallel	Unspecified method	Double	Yes	18	21
Barr 1983	800 mg	w432	Recently healed ulcers	Parallel	Other method	Double	Yes	27	30
Korman 1980	800 mg	w433	Recently healed ulcers	Parallel	Unspecified method	Double	Yes	15	26
Jensen 1980	800 mg	w434	Recently healed ulcers	Parallel	Closed list	Double	Yes	35	35
Kang 1980	800 mg	w435	Recently healed ulcers	Parallel	Unspecified method	Double	No	16	15
Nyren 1986	800 mg	w436	Gastro-oesophageal reflux	Parallel	Unspecified method	Double	No	51	54
Bardhan 1979 (healed duodenal ulcer)	800 mg	w437	Recently healed ulcers	Parallel	Unspecified method	Double	Yes	32	33
Dronfield 1979	800 mg	w438	Recently healed ulcers	Parallel	Pharmacy	Double	Yes	22	22
Bijlsma 1988	800 mg	w439	Antiplatelet therapy	Parallel	Unspecified method	Double	No	64	63
Anderson 1985	800 mg	w440	Bronchiestasis	Crossover	Unspecified method	Double	No	6	6
Blackwood 1978	800 mg	w441	Recently healed ulcers	Parallel	Unspecified method	Double	Yes	28	27
Bodemar 1979 A	800 mg	w442	Recently healed ulcers	Parallel	Unspecified method	Double	Yes	32	36
Hetzel 1978 B	800 mg	w443	Recently healed ulcers	Parallel	Unspecified method	Double	Yes	36	31
Gudmand-Høyer 1978	800 mg	w444	Recently healed ulcers	Parallel	Unspecified method	Double	Yes	29	28
Frost 1979 X2	800 mg	w445	Recently healed ulcers	Extension	Unspecified method	Double	Yes	11	12
Colle 1988	800 mg	w446	Healthy volunteers	Parallel	Closed list	Double	No	8	8
Matsumoto 1996	800 mg	w447	Colorectal cancer	Parallel	Unspecified method	Unknown	Yes	34	30
Johnsson 1993	800 mg	w448	Gastro-oesophageal reflux	Parallel	Unspecified method	Double	Yes	124	127
Hetzel 1979	800 mg	w449	Recently healed ulcers	Parallel	Unspecified method	Double	Yes	28	28
Hansky 1979	800 mg	w450	Recently healed ulcers	Parallel	Unspecified method	Double	Yes	20	20
Mekel 1978	800 mg	w451	Recently healed ulcers	Parallel	Unspecified method	Double	Yes	33	14
Nesland 1985	800 mg	w452	Gastro-oesophageal reflux	Parallel	Unspecified method	Double	No	50	50
Svendsen 1995	800 mg	w453	Colorectal cancer	Parallel	Unspecified method	Double	No	98	91
Talley 1986	800 mg	w454	Gastro-oesophageal reflux	Crossover	Unspecified method	Double	No	29	29
Singal 1989	800 mg	w455	Gastro-oesophageal reflux	Parallel	Unspecified method	Double	No	33	34
Riemann 1991	800 mg	w456	Gastro-oesophageal reflux	Parallel	Unspecified method	Double	Yes	80	82
Burland 1980	800 mg	w413	Recently healed ulcers	Parallel	Unspecified method	Double	Yes	184	333
Rugstad 1994	800 mg	w457	Antiplatelet therapy	Parallel	Unspecified method	Double	No	285	285
Gotthard 1988	800 mg	w458	Gastro-oesophageal reflux	Parallel	Unspecified method	Double	Yes	73	75
Lee 2001	800 mg	w459	Planar warts	Parallel	Unspecified method	Unknown	No	9	8
Tonnesen 1988	800 mg	w460	Gastric cancer	Parallel	Telephone	Double	Yes	68	77

Webtable 3 (cont.): Details of trials involving a randomised comparison of a histamine-2 receptor antagonists (X) versus control (Y)

Trial name	Regimen	Ref.	Indication for treatment	Design	Allocation Concealment	Blinding of investigators and patients	Availability of data	No. of patients NX	NY
Carpenter 1983	900 mg	w461	Allergic rhinitis	Crossover	Unspecified method	Double	No	12	12
Mackinnon 1982	1000 mg	w462	Gastritis or duodenitis	Parallel	Unspecified method	Double	No	14	12
Niv 1990	1000 mg	w463	Gastritis or duodenitis	Parallel	Unspecified method	Double	Yes	10	7
Rune 1980	1000 mg	w464	Recently healed ulcers	Parallel	Unspecified method	Double	Yes	25	20
Bennett 1980	1000 mg	w465	Gastro-oesophageal reflux	Parallel	Unspecified method	Unknown	No	-	-
Willing 1980	1000 mg	w466	Gastritis or duodenitis	Parallel	Unspecified method	Double	No	11	10
Camarri 1980	1000 mg	w467	Rheumatoid arthritis	Crossover	Unspecified method	Double	No	4	4
Breen 1983	1000 mg	w468	Gastro-oesophageal reflux	Parallel	Sealed envelopes	Double	No	14	13
Hamid 1983	1000 mg	w469	Crohn's disease	Parallel	Unspecified method	Double	No	18	18
Kelbaek 1985	1000 mg	w470	Gastro-oesophageal reflux	Parallel	Unspecified method	Double	No	24	26
Machell 1979	1000 mg	w471	Recently healed ulcers	Parallel	Unspecified method	Double	Yes	12	14
Brown 1979	1000 mg	w472	Gastro-oesophageal reflux	Parallel	Unspecified method	Double	No	11	11
Bardhan 1982	1000 mg	w473	Recently healed ulcers	Parallel	Unspecified method	Double	Yes	66	65
Popoola 1978 (antroduodenitis)	1000 mg	w474	Gastritis or duodenitis	Parallel	Unspecified method	Double	Yes	5	4
Sillero 1981	1053 mg	w475	Acute pancreatiits	Parallel	Sealed envelopes	Double	Yes	30	30
Perez De Oteyza 1980	1067 mg	w476	Acute pancreatiits	Parallel	Unspecified method	Double	Yes	20	20
Roth 1987 X1	1200 mg	w377	Arthritis, rheumatic or musculoskeletal disorders	Extension	Unspecified method	Double	Yes	54	50
Støa-Birketvedt 1998	1200 mg	w477	Diabetes	Parallel	Unspecified method	Double	No	31	31
Bendayan 1993	1200 mg	w478	Healthy volunteers	Crossover	Unspecified method	Double	No	23	23
Bright-Asare 1980	1200 mg	w479	Gastro-oesophageal reflux	Parallel	Unspecified method	Double	No	20	10
Cocco 1981	1200 mg	w393	Recently healed ulcers	Parallel	Unspecified method	Unknown	No	-	-
Layne 1981	1200 mg	w480	Neurological diseases	Parallel	Unspecified method	Double	Yes	16	20
Cohen 1996	1200 mg	w481	HIV	Parallel	Unspecified method	Double	Yes	94	88
Ferguson 1979	1200 mg	w482	Gastro-oesophageal reflux	Crossover	Unspecified method	Double	No	10	10
Monroe 1981	1200 mg	w483	Urticaria	Crossover	Unspecified method	Double	Yes	9	9
Behar 1978	1200 mg	w484	Gastro-oesophageal reflux	Parallel	Unspecified method	Double	No	49	45
Greenblatt 1984	1200 mg	w485	Insomnia	Crossover	Unspecified method	Double	No	5	5
Hoogwerf 1984	1200 mg	w486	Healthy volunteers	Crossover	Unspecified method	Double	Yes	12	12
Yan 1999	1200 mg	w487	Gastrointestinal cancer	Parallel	Unspecified method	Open	No	25	24
Augusto Larrain 1991	1200 mg	w488	Gastro-oesophageal reflux	Parallel	Unspecified method	Unknown	No	30	30
Diller 1983	1200 mg	w489	Urticaria	Crossover	Unspecified method	Double	No	8	8
Shanehsaz 2015	1200 mg	w490	Cutaneous leishmaniasis	Parallel	Unspecified method	Double	Yes	30	30
Lissak 1989	1500 mg	w491	Healthy volunteers	Parallel	Unspecified method	Unknown	No	12	8
Lepsien 1979	1600 mg	w492	Gastro-oesophageal reflux	Parallel	Unspecified method	Double	Yes	16	20
Wallach 1982	1600 mg	w493	Psoriasis	Parallel	Unspecified method	Double	No	18	18
Fiasse 1980	1600 mg	w494	Gastro-oesophageal reflux	Parallel	Unspecified method	Double	Yes	23	21
Festen 1980	1600 mg	w495	Gastro-oesophageal reflux	Parallel	Unspecified method	Double	No	13	7
Bleehen 1987	1600 mg	w496	Urticaria	Parallel	Unspecified method	Double	No	21	19
Commens 1978	1600 mg	w497	Urticaria	Crossover	Unspecified method	Double	No	-	-
Cook 1979	1600 mg	w498	Urticaria	Parallel	Unspecified method	Double	No	-	-
Palmer 1993	1600 mg	w499	Gastro-oesophageal reflux	Parallel	Unspecified method	Double	No	85	82
Palmer 1993	1600 mg	w499	Gastro-oesophageal reflux	Parallel	Unspecified method	Double	No	83	82
Sitsen 2000	1600 mg	w500	Healthy volunteers	Crossover	Unspecified method	Double	Yes	6	6
Powell-Jackson 1978	1600 mg	w501	Gastro-oesophageal reflux	Crossover	Unspecified method	Double	No	14	14
Wesdorp 1978	1600 mg	w502	Gastro-oesophageal reflux	Parallel	Unspecified method	Double	Yes	13	13
Palmer 1990	1600 mg	w503	Gastro-oesophageal reflux	Parallel	Unspecified method	Double	Yes	93	86
Fitzpatrick 1986	1600 mg	w504	Ileal resection	Crossover	Unspecified method	Unknown	No	5	5
Matthews 1979	1600 mg	w505	Demographism	Crossover	Unspecified method	Double	No	8	8
Jameson 2014	1600 mg	w506	Colorectal cancer	Parallel	Unspecified method	Double	No	65	63
Bennett 1980	2000 mg	w465	Gastro-oesophageal reflux	Parallel	Unspecified method	Unknown	No	-	-
Famotidine vs Control									
Baglioni 1985	20 mg	w507	Recently healed ulcers	Parallel	Unspecified method	Double	Yes	167	177

Webtable 3 (cont.): Details of trials involving a randomised comparison of a histamine-2 receptor antagonists (X) versus control (Y)

Trial name	Regimen	Ref.	Indication for treatment	Design	Allocation Concealment	Blinding of investigators and patients	Availability of data	No. of patients NX	NY
Berlin 1991	20 mg	w508	Recently healed ulcers	Parallel	Unspecified method	Double	Yes	102	100
Texter 1986	20 mg	w509	Recently healed ulcers	Parallel	Unspecified method	Double	Yes	97	99
Zaterka 1988 X2	20 mg	w510	Recently healed ulcers	Extension	Unspecified method	Single	Yes	20	19
Hongo 2008 A	20 mg	w511	Gastro-oesophageal reflux	Parallel	Closed list	Double	No	177	178
FAMOUS	40 mg	w512	Antiplatelet therapy	Parallel	Sealed envelopes	Double	Yes	204	200
Taha 1996	40 mg	w513	Arthritis, rheumatic or musculoskeletal disorders	Parallel	Closed list	Double	Yes	95	93
Kato 2005	40 mg	w514	Gastro-oesophageal reflux	Crossover	Unspecified method	Double	No	9	10
Poyurovsky 2003	40 mg	w515	Acute psychosis	Parallel	Unspecified method	Double	Yes	7	7
Schwartz 1995	40 mg	w516	Healthy volunteers	Parallel	Unspecified method	Double	Yes	64	64
Texter 1986	40 mg	w509	Recently healed ulcers	Parallel	Unspecified method	Double	Yes	107	99
Robinson 1991 B	40 mg	w517	Gastro-oesophageal reflux	Parallel	Unspecified method	Double	No	158	76
Robinson 1991 B	40 mg	w517	Gastro-oesophageal reflux	Parallel	Unspecified method	Double	No	155	76
Simon 1994 B	40 mg	w518	Gastro-oesophageal reflux	Parallel	Unspecified method	Double	No	125	66
Simon 1995 B	40 mg	w519	Gastro-oesophageal reflux	Parallel	Unspecified method	Double	No	69	31
Simon 1996	40 mg	w520	Gastro-oesophageal reflux	Parallel	Unspecified method	Double	Yes	110	49
Hongo 2008 A	40 mg	w511	Gastro-oesophageal reflux	Parallel	Closed list	Double	No	174	178
Liu 2003	40 mg	w521	Recently healed ulcers	Parallel	Closed list	Unknown	Yes	18	26
Sabesin 1991	40 mg	w522	Gastro-oesophageal reflux	Parallel	Unspecified method	Double	Yes	137	66
Sabesin 1991	40 mg	w522	Gastro-oesophageal reflux	Parallel	Unspecified method	Double	Yes	135	66
Ohara 2010 A	40 mg	w523	Gastro-oesophageal reflux	Parallel	Unspecified method	Unknown	No	136	65
Ohara 2010 B	40 mg	w524	Gastro-oesophageal reflux	Parallel	Closed list	Double	No	163	169
Hsu 2015	40 mg	w525	Antiplatelet therapy	Parallel	Unspecified method	Double	Yes	115	123
Hsu 2013	40 mg	w526	Antiplatelet therapy	Parallel	Unspecified method	Unknown	Yes	91	90
REDUCE-1 and REDUCE-2	79.8 mg	w527	Antiplatelet therapy	Parallel	IVRS	Double	Yes	1022	513
Hudson 1997	80 mg	w528	Arthritis, rheumatic or musculoskeletal disorders	Parallel	Unspecified method	Double	Yes	39	39
Taha 1996	80 mg	w513	Arthritis, rheumatic or musculoskeletal disorders	Parallel	Closed list	Double	Yes	97	93
Marrero 1994	80 mg	w529	Gastro-oesophageal reflux	Parallel	Unspecified method	Double	No	14	13
Schwartz 1995	80 mg	w516	Healthy volunteers	Parallel	Unspecified method	Double	Yes	64	64
Simon 1994 B	80 mg	w518	Gastro-oesophageal reflux	Parallel	Unspecified method	Double	No	127	66
Simon 1995 B	80 mg	w519	Gastro-oesophageal reflux	Parallel	Unspecified method	Double	No	72	31
Simon 1996	80 mg	w520	Gastro-oesophageal reflux	Parallel	Unspecified method	Double	Yes	108	49
Mestre 2014	80 mg	w530	Parkinson's disease	Crossover	Unspecified method	Double	No	2	2
Mestre 2014	120 mg	w530	Parkinson's disease	Crossover	Unspecified method	Double	No	2	2
Mestre 2014	160 mg	w530	Parkinson's disease	Crossover	Unspecified method	Double	No	2	2
Meskanen 2013	200 mg	w531	Schizophrenia	Parallel	Closed list	Double	Yes	16	14
Lafutidine vs Control									
Namikawa 2014	10 mg	w532	Gastric cancer	Parallel	Sealed envelopes	Unknown	No	10	12
Ohara 2010 A	20 mg	w523	Gastro-oesophageal reflux	Parallel	Unspecified method	Unknown	No	135	65
Ohara 2010 B	20 mg	w524	Gastro-oesophageal reflux	Parallel	Closed list	Double	No	169	169
Kato 2010 B	20 mg	w533	Antiplatelet therapy	Crossover	Unspecified method	Double	Yes	8	8
Nizatidine vs Control									
Carlson 2002	150 mg	w534	Alzheimer's Disease	Parallel	Unspecified method	Double	No	25	26
Cerulli 1987	150 mg	w535	Recently healed ulcers	Parallel	Unspecified method	Double	Yes	257	256
Rampal 1994	150 mg	w536	Recently healed ulcers	Parallel	Other method	Open	Yes	286	295
GISU 1993	150 mg	w537	Recently healed ulcers	Parallel	Unspecified method	Double	Yes	123	118
Levine 1993	300 mg	w538	Osteoarthritis	Parallel	Unspecified method	Double	Yes	248	248
Cavazzoni 2003	300 mg	w539	Schizophrenia	Parallel	Unspecified method	Double	No	57	60
Dammann 1990	300 mg	w540	Healthy volunteers	Crossover	Unspecified method	Single	Yes	4	4
Hansen 1998	300 mg	w541	Gastro-oesophageal reflux	Parallel	Onsite computer	Double	No	111	110
Cloud 1991	300 mg	w542	Gastro-oesophageal reflux	Parallel	Onsite computer	Double	Yes	151	160
Cloud 1991	300 mg	w542	Gastro-oesophageal reflux	Parallel	Onsite computer	Double	Yes	155	160

Webtable 3 (cont.): Details of trials involving a randomised comparison of a histamine-2 receptor antagonists (X) versus control (Y)

Trial name	Regimen	Ref.	Indication for treatment	Design	Allocation Concealment	Blinding of investigators and patients	Availability of data	No. of patients NX	NY
Atmaca 2003 A	300 mg	w543	Schizophrenia	Parallel	Unspecified method	Double	No	18	17
Atmaca 2004 B	300 mg	w544	Schizophrenia	Parallel	Unspecified method	Double	No	14	14
Cloud 1992 A	300 mg	w545	Gastro-oesophageal reflux	Parallel	Closed list	Double	Yes	168	178
Dobrilla 1992	300 mg	w546	Gastro-oesophageal reflux	Parallel	Unspecified method	Double	No	58	59
Koskenpato 2008	300 mg	w547	Gastro-oesophageal reflux	Crossover	Unspecified method	Double	No	8	8
Quik 1990	300 mg	w548	Osteoarthritis	Parallel	Unspecified method	Double	No	109	107
Cloud 1989 B X4	300 mg	w549	Recently healed ulcers	Extension	Unspecified method	Double	Yes	198	95
Cavazzoni 2003	600 mg	w539	Schizophrenia	Parallel	Unspecified method	Double	No	58	60
Dammann 1990	600 mg	w540	Healthy volunteers	Crossover	Unspecified method	Single	Yes	4	4
Assuncao 2005	600 mg	w550	Schizophrenia	Parallel	Unspecified method	Double	Yes	27	27
Cloud 1992 A	600 mg	w545	Gastro-oesophageal reflux	Parallel	Closed list	Double	Yes	169	178
Quik 1990	600 mg	w548	Osteoarthritis	Parallel	Unspecified method	Double	No	109	107
Other H2RA vs Control									
Guerreiro 1991 X2	Unknown	w551	Recently healed ulcers	Extension	Unspecified method	Unknown	Yes	61	76
Murakami 2006	Unknown	w552	Recently healed ulcers	Parallel	Sealed envelopes	Open	Yes	156	153
Murakami 2006	Unknown	w552	Recently healed ulcers	Parallel	Sealed envelopes	Open	Yes	158	153
Ranitidine vs Control									
Vakil 2006	75 mg	w553	Gastro-oesophageal reflux	Parallel	Sealed envelopes	Double	No	17	16
Mignon 1990	150 mg	w554	Recently healed ulcers	Parallel	Closed list	Double	Yes	197	202
Andrade 1997	150 mg	w555	Recently healed ulcers	Parallel	Unspecified method	Double	Yes	25	25
Kang 1988	150 mg	w556	Recently healed ulcers	Parallel	Unspecified method	Double	Yes	35	35
Piai 1990	150 mg	w557	Recently healed ulcers	Parallel	Sealed envelopes	Open	Yes	29	31
Van Deventer 1989	150 mg	w558	Recently healed ulcers	Parallel	Unspecified method	Double	Yes	70	70
Kozarek 1985	150 mg	w559	Recently healed ulcers	Parallel	Unspecified method	Double	Yes	138	139
Alstead 1983 (healed gastric ulcer)	150 mg	w560	Recently healed ulcers	Parallel	Unspecified method	Double	Yes	15	16
Koelz 1986	150 mg	w561	Gastro-oesophageal reflux	Parallel	Unspecified method	Double	Yes	32	37
Boyd 1984	150 mg	w562	Recently healed ulcers	Parallel	Unspecified method	Double	Yes	21	26
Di Mario 1982	150 mg	w563	Recently healed ulcers	Parallel	Unspecified method	Open	Yes	31	30
Dawson 1984 X2	150 mg	w564	Recently healed ulcers	Extension	Unspecified method	Double	Yes	24	24
Goldin 1991	150 mg	w278	Recently healed ulcers	Parallel	Closed list	Double	Yes	24	12
GISU 1994	150 mg	w565	Recently healed ulcers	Parallel	Unspecified method	Open	Yes	105	95
Jensen 1994	150 mg	w566	Recently healed ulcers	Parallel	Unspecified method	Double	Yes	32	33
Blum 2000	150 mg	w104	Gastro-oesophageal reflux	Parallel	Unspecified method	Double	Yes	194	203
Hui 1992	150 mg	w373	Recently healed ulcers	Parallel	Unspecified method	Single	Yes	100	103
Monés 2001	150 mg	w567	Recently healed ulcers	Parallel	Unspecified method	Double	Yes	36	37
Murray 1987	150 mg	w568	Recently healed ulcers	Parallel	Unspecified method	Double	Yes	20	20
Mekel 1985	150 mg	w569	Recently healed ulcers	Parallel	Unspecified method	Double	Yes	22	21
Sue 1996	150 mg	w570	Recently healed ulcers	Parallel	Unspecified method	Double	Yes	117	109
Jorde 1987	150 mg	w571	Recently healed ulcers	Parallel	Unspecified method	Double	Yes	25	25
Ruszniewski 1993	150 mg	w572	Recently healed ulcers	Parallel	Unspecified method	Double	Yes	197	202
Manes 1998	150 mg	w573	Recently healed ulcers	Parallel	Unspecified method	Open	Yes	33	33
Zdrenghea 2004	150 mg	w574	Dyslipidaemia	Parallel	Unspecified method	Unknown	No	20	20
Hovdenak 1987	150 mg	w575	Recently healed ulcers	Parallel	Unspecified method	Double	Yes	29	30
Russo 1997 X2	150 mg	w66	Recently healed ulcers	Extension	Closed list	Double	Yes	24	26
Villalobos 1991	150 mg	w576	Recently healed ulcers	Parallel	Unspecified method	Double	Yes	24	27
Alstead 1983 (healed duodenal ulcer)	150 mg	w560	Recently healed ulcers	Parallel	Unspecified method	Double	Yes	20	17
Mehta 2014	150 mg	w577	Schizophrenia	Parallel	Other method	Unknown	No	25	25
Tamura 1991	200 mg	w578	Varices	Parallel	Sealed envelopes	Unknown	Yes	18	17
Penston 1993	225 mg	w579	Recently healed ulcers	Parallel	Unspecified method	Double	Yes	46	46
Ehsanullah 1988	300 mg	w580	Arthritis, rheumatic or musculoskeletal disorders	Parallel	Other method	Double	Yes	151	146

Webtable 3 (cont.): Details of trials involving a randomised comparison of a histamine-2 receptor antagonists (X) versus control (Y)

Trial name	Regimen	Ref.	Indication for treatment	Design	Allocation Concealment	Blinding of investigators and patients	Availability of data	No. of patients NX	NY
Robinson 1989	300 mg	w581	Arthritis, rheumatic or musculoskeletal disorders	Parallel	Unspecified method	Double	Yes	72	72
Van Groenendael 1996	300 mg	w582	Arthritis, rheumatic or musculoskeletal disorders	Parallel	Closed list	Double	Yes	47	47
Robinson 1991 A	300 mg	w583	Arthritis, rheumatic or musculoskeletal disorders	Parallel	Unspecified method	Double	Yes	112	115
Swift 1989	300 mg	w584	Arthritis, rheumatic or musculoskeletal disorders	Crossover	Pharmacy	Double	Yes	8	8
Berkowitz 1987	300 mg	w585	Healthy volunteers	Parallel	Unspecified method	Double	Yes	25	25
Nielsen 1998	300 mg	w586	Colorectal cancer	Parallel	Unspecified method	Double	No	-	-
Müller 1997	300 mg	w58	Healthy volunteers	Parallel	Unspecified method	Double	No	10	10
Müller 1992 B	300 mg	w587	Healthy volunteers	Parallel	Unspecified method	Double	No	11	11
Müller 1991	300 mg	w588	Healthy volunteers	Crossover	Unspecified method	Double	No	5	5
Nervi 1989	300 mg	w589	Gastro-oesophageal reflux	Parallel	Unspecified method	Unknown	No	20	20
Rush 1995	300 mg	w590	Gastro-oesophageal reflux	Parallel	Onsite computer	Double	Yes	408	404
Kar 1990	300 mg	w591	Gastro-oesophageal reflux	Parallel	Unspecified method	Double	No	15	15
Yanagawa 1991	300 mg	w592	Arthritis, rheumatic or musculoskeletal disorders	Parallel	Sealed envelopes	Unknown	Yes	43	37
Alberts 1991	300 mg	w593	Cancer or palliative care (non gastrointestinal)	Crossover	Unspecified method	Double	No	4	3
Yanagawa 1990	300 mg	w594	Arthritis, rheumatic or musculoskeletal disorders	Parallel	Sealed envelopes	Double	Yes	31	29
Müller 1992 A	300 mg	w595	Healthy volunteers	Parallel	Unspecified method	Double	No	14	14
Simon 1988	300 mg	w596	Arthritis, rheumatic or musculoskeletal disorders	Parallel	Unspecified method	Double	Yes	23	23
Primrose 1998	300 mg	w597	Gastric cancer	Parallel	Unspecified method	Double	Yes	112	110
Berenson 1987	300 mg	w598	Gastro-oesophageal reflux	Parallel	Unspecified method	Double	Yes	134	150
Robinson 1987	300 mg	w599	Gastro-oesophageal reflux	Parallel	Unspecified method	Unknown	No	-	-
Saunders 1986	300 mg	w600	Gastro-oesophageal reflux	Parallel	Pharmacy	Double	No	243	253
Hendel 1986	300 mg	w601	Gastro-oesophageal reflux	Parallel	Unspecified method	Double	No	10	8
Johansson 1986	300 mg	w602	Gastro-oesophageal reflux	Crossover	Unspecified method	Double	No	20	22
Müller 1994 C	300 mg	w603	Gastro-oesophageal reflux	Parallel	Unspecified method	Double	No	254	244
Hegarty 1997	300 mg	w604	Gastro-oesophageal reflux	Parallel	Unspecified method	Double	No	92	92
Gustavsson 1987	300 mg	w605	Gastro-oesophageal reflux	Parallel	Closed list	Double	No	15	15
Fullarton 1992	300 mg	w606	Hypergastrinaemia	Crossover	Unspecified method	Double	No	4	4
Hallerback 1998 B	300 mg	w607	Gastro-oesophageal reflux	Parallel	Unspecified method	Double	Yes	210	213
Orlowski 1986	300 mg	w608	Renal transplant	Parallel	Unspecified method	Double	Yes	21	21
Olubuyide 1986	300 mg	w609	Gastro-oesophageal reflux	Parallel	Unspecified method	Double	No	23	22
Sherbaniuk 1984	300 mg	w610	Gastro-oesophageal reflux	Parallel	Unspecified method	Double	No	37	36
Nielsen 1999	300 mg	w611	Colorectal cancer	Parallel	Unspecified method	Double	Yes	455	450
Wotherspoon 1997	300 mg	w612	Gastric cancer	Parallel	Unspecified method	Unknown	Yes	79	80
Tan 1996	300 mg	w613	End stage renal disease	Crossover	Unspecified method	Double	Yes	6	9
Sartori 2000	300 mg	w131	Cancer or palliative care (non gastrointestinal)	Parallel	Unspecified method	Unknown	Yes	77	75
Smith 1999	300 mg	w614	Healthy volunteers	Parallel	Unspecified method	Double	No	17	14
Singh 1999	300 mg	w615	Post-operative	Parallel	Sealed envelopes	Unknown	No	90	84
Goy 1983	300 mg	w616	Gastro-oesophageal reflux	Parallel	Closed list	Double	No	18	19
Wallace 2001	300 mg	w617	Familial adenomatous polyposis	Parallel	Closed list	Double	Yes	13	13
Czapla 1992	300 mg	w618	End stage renal disease	Crossover	Unspecified method	Double	Yes	7	8
Hadi 1989	300 mg	w619	Gastritis or duodenitis	Parallel	Unspecified method	Double	Yes	26	26
Farup 1997	300 mg	w620	Gastro-oesophageal reflux	Parallel	Unspecified method	Double	No	103	103
Wesdorp 1983	300 mg	w621	Gastro-oesophageal reflux	Parallel	Unspecified method	Double	No	19	17
Müller 1989	300 mg	w622	Healthy volunteers	Parallel	Unspecified method	Double	No	10	10
Müller 1998	300 mg	w190	Healthy volunteers	Parallel	Unspecified method	Double	Yes	16	8
Simon 1992	300 mg	w623	Healthy volunteers	Crossover	Unspecified method	Double	Yes	3	3
Elizalde 1983 (oesophagitis)	300 mg	w624	Gastro-oesophageal reflux	Parallel	Unspecified method	Double	No	-	-
Metzenroth 1991	300 mg	w625	Hip replacement	Parallel	Unspecified method	Unknown	No	20	20

Webtable 3 (cont.): Details of trials involving a randomised comparison of a histamine-2 receptor antagonists (X) versus control (Y)

Trial name	Regimen	Ref.	Indication for treatment	Design	Allocation Concealment	Blinding of investigators and patients	Availability of data	No. of patients NX	NY
Richter 2000 C	300 mg	w67	Gastro-oesophageal reflux	Parallel	Unspecified method	Double	No	283	70
Rovner 2003	300 mg	w626	Weight loss	Parallel	Unspecified method	Open	No	28	29
Zonneveld 1997	300 mg	w627	Psoriasis	Parallel	Unspecified method	Double	No	-	-
Grove 1985	300 mg	w628	Gastro-oesophageal reflux	Crossover	Unspecified method	Double	No	28	28
Corinaldesi 1991	300 mg	w629	Dental	Parallel	Unspecified method	Double	Yes	23	25
Hine 1984	300 mg	w630	Gastro-oesophageal reflux	Parallel	Unspecified method	Double	No	21	25
Ideo 1988	300 mg	w631	Varices	Parallel	Closed list	Single	Yes	22	27
Farup 1992	300 mg	w632	Oesophageal stricture	Parallel	Unspecified method	Double	Yes	37	34
Mehta 2014	300 mg	w577	Schizophrenia	Parallel	Other method	Unknown	No	25	25
Lehtola 1986	450 mg	w633	Gastro-oesophageal reflux	Parallel	Unspecified method	Double	No	21	20
Wilder-Smith 1990 B	525 mg	w634	Healthy volunteers	Crossover	Unspecified method	Double	No	6	6
ten Wolde 1996	600 mg	w635	Rheumatoid arthritis	Parallel	Closed list	Double	Yes	15	15
Swift 1989	600 mg	w584	Arthritis, rheumatic or musculoskeletal disorders	Crossover	Pharmacy	Double	Yes	8	8
Müller 1992 B	600 mg	w587	Healthy volunteers	Parallel	Unspecified method	Double	No	9	11
King 2002	600 mg	w636	Colorectal cancer	Parallel	Unspecified method	Double	Yes	60	57
Veien 1995	600 mg	w637	Eczema	Parallel	Unspecified method	Double	No	23	24
Euler 1993 B	600 mg	w638	Gastro-oesophageal reflux	Parallel	Unspecified method	Double	No	106	116
Hegarty 1997	600 mg	w604	Gastro-oesophageal reflux	Parallel	Unspecified method	Double	No	95	92
Cetin 1997	600 mg	w639	Psoriasis	Parallel	Unspecified method	Double	No	25	25
Jurlander 1995	600 mg	w640	Leukaemia	Parallel	Unspecified method	Open	No	15	16
Nielson 1991	600 mg	w641	HIV	Parallel	Unspecified method	Double	Yes	9	9
Nielsen 1990	600 mg	w642	Multiple myeloma	Parallel	Unspecified method	Double	No	10	10
Roufail 1992	600 mg	w643	Gastro-oesophageal reflux	Parallel	Unspecified method	Double	No	109	113
Silver 1996	600 mg	w644	Gastro-oesophageal reflux	Parallel	Unspecified method	Double	No	261	251
Silver 1996	600 mg	w644	Gastro-oesophageal reflux	Parallel	Unspecified method	Double	No	260	251
Bartlett 1998	600 mg	w645	HIV	Parallel	Unspecified method	Double	Yes	38	43
Vendelbo Johansen 1997	600 mg	w646	Infectious mononucleosis	Parallel	Unspecified method	Double	No	50	41
Tefera 1996 (healed duodenal ulcer+ metronizadole)	600 mg	w647	Gastritis or duodenitis	Parallel	Closed list	Double	No	9	15
Tefera 1996 (healed duodenal ulcer+ no metronizadole)	600 mg	w647	Gastritis or duodenitis	Parallel	Closed list	Double	No	11	6
Simon 1992	600 mg	w623	Healthy volunteers	Crossover	Unspecified method	Double	Yes	3	3
Rovner 2003	600 mg	w626	Weight loss	Parallel	Unspecified method	Open	No	28	29
Zonneveld 1997	600 mg	w627	Psoriasis	Parallel	Unspecified method	Double	No	-	-
Houben 1995	600 mg	w141	Healthy volunteers	Crossover	Unspecified method	Double	No	3	3
Ranjbar 2013	600 mg	w648	Schizophrenia	Parallel	Unspecified method	Double	No	25	27
Wilder-Smith 1990 C	900 mg	w634	Healthy volunteers	Crossover	Unspecified method	Double	No	7	7
Euler 1993 B	1200 mg	w638	Gastro-oesophageal reflux	Parallel	Unspecified method	Double	No	106	116
Roufail 1992	1200 mg	w643	Gastro-oesophageal reflux	Parallel	Unspecified method	Double	No	120	113
Chen ZQ 2010	Unknown	w649	Dual antiplatelet therapy	Parallel	Unspecified method	Unknown	Yes	100	100
Ramixotidine vs Control									
Colle 1988	750 mg	w446	Healthy volunteers	Parallel	Closed list	Double	No	8	8
Roxatidine vs Control									
Simon 1991	75 mg	w650	Healthy volunteers	Parallel	Unspecified method	Double	No	10	10
European Cooperative Roxatidine (duodenal ulcer)	75 mg	w651	Recently healed ulcers	Parallel	Unspecified method	Double	Yes	211	209
European Cooperative Roxatidine (gastric ulcer)	75 mg	w651	Recently healed ulcers	Parallel	Unspecified method	Double	Yes	153	152
Damman 1995	75 mg	w652	Healthy volunteers	Crossover	Unspecified method	Single	No	4	4
Müller 1994 A	150 mg	w653	Healthy volunteers	Crossover	Unspecified method	Double	Yes	10	10
Lassman 1988	150 mg	w654	Healthy volunteers	Parallel	Unspecified method	Double	No	10	4
Damman 1995	150 mg	w652	Healthy volunteers	Crossover	Unspecified method	Single	No	4	4

Webtable 3 (cont.): Details of trials involving a randomised comparison of a histamine-2 receptor antagonists (X) versus control (Y)

Trial name	Regimen	Ref.	Indication for treatment	Design	Allocation Concealment	Blinding of investigators and patients	Availability of data	No. of patients NX	NY
**Healing Trials**									
Cimetidine vs Control									
Braverman 1986	400 mg	w655	Peptic ulcers	Parallel	Unspecified method	Double	Yes	182	168
Young 1989 A	400 mg	w656	Peptic ulcers	Parallel	Unspecified method	Double	Yes	182	168
Gray 1978 (incompletely healed ulcers)	400 mg	w414	Peptic ulcers	Parallel	Unspecified method	Double	Yes	3	3
Domschke 1976	800 mg	w657	Peptic ulcers	Parallel	Unspecified method	Double	No	-	-
Frank 1989 B	800 mg	w658	Peptic ulcers	Parallel	Pharmacy	Double	Yes	83	80
Braverman 1986	800 mg	w655	Peptic ulcers	Parallel	Unspecified method	Double	Yes	165	168
Bodemar 1976	800 mg	w659	Peptic ulcers	Parallel	Unspecified method	Double	No	15	15
Frank 1989 A	800 mg	w660	Peptic ulcers	Parallel	Unspecified method	Double	Yes	92	94
Bodemar 1979 B	800 mg	w442	Peptic ulcers	Parallel	Unspecified method	Double	Yes	15	14
Mosci 1989	800 mg	w661	Peptic ulcers	Parallel	Unspecified method	Double	Yes	26	26
Tay 1988	800 mg	w662	Peptic ulcers	Parallel	Other method	Double	Yes	17	15
Young 1989 A	800 mg	w656	Peptic ulcers	Parallel	Unspecified method	Double	Yes	165	168
Moshal 1977	800 mg	w663	Peptic ulcers	Parallel	Unspecified method	Double	Yes	19	19
Hoffenberg 1985	800 mg	w664	Peptic ulcers	Parallel	Pharmacy	Double	Yes	24	22
Isenberg 1982	900 mg	w665	Peptic ulcers	Parallel	Unspecified method	Double	No	35	31
Hentschel 1979	1000 mg	w666	Peptic ulcers	Parallel	Unspecified method	Double	Yes	32	31
Garbøl 1979	1000 mg	w667	Peptic ulcers	Parallel	Closed list	Double	Yes	23	25
Tárnok 1980	1000 mg	w668	Peptic ulcers	Parallel	Unspecified method	Unknown	Yes	22	20
Sonnenberg 1979 X1	1000 mg	w379	Peptic ulcers	Extension	Unspecified method	Double	Yes	31	32
Peter 1978	1000 mg	w669	Peptic ulcers	Parallel	Unspecified method	Double	Yes	38	40
Fica 1987	1000 mg	w670	Peptic ulcers	Parallel	Unspecified method	Unknown	Yes	20	20
Bongiorno 1983	1000 mg	w671	Peptic ulcers	Parallel	Unspecified method	Double	Yes	30	30
Marini 1980	1000 mg	w672	Peptic ulcers	Parallel	Pharmacy	Double	Yes	18	18
Mazure 1980	1000 mg	w673	Peptic ulcers	Parallel	Unspecified method	Double	Yes	20	20
Lam 1983	1000 mg	w674	Peptic ulcers	Parallel	Unspecified method	Open	Yes	40	20
Eichenberger 1982 X1	1000 mg	w388	Peptic ulcers	Extension	Unspecified method	Double	Yes	23	23
Dobrilla 1980	1000 mg	w675	Peptic ulcers	Parallel	Unspecified method	Double	Yes	17	15
Danielsson 1980 X1	1000 mg	w676	Peptic ulcers	Parallel	Unspecified method	Double	Yes	25	25
Ström 1981	1000 mg	w677	Peptic ulcers	Parallel	Unspecified method	Double	Yes	24	24
Corinaldesi 1980	1000 mg	w678	Peptic ulcers	Parallel	Unspecified method	Unknown	No	12	12
Johannessen 1986	1000 mg	w679	Peptic ulcers	Parallel	Unspecified method	Double	Yes	24	24
Barakat 1984 A	1000 mg	w359	Peptic ulcers	Parallel	Unspecified method	Double	Yes	40	39
Dawson 1984 X1	1000 mg	w564	Peptic ulcers	Extension	Unspecified method	Single	Yes	20	20
Frost 1977 X1	1000 mg	w680	Peptic ulcers	Extension	Unspecified method	Double	Yes	27	24
Giger 1979	1000 mg	w681	Peptic ulcers	Parallel	Unspecified method	Double	Yes	16	18
Ciclitira 1977	1000 mg	w682	Peptic ulcers	Parallel	Unspecified method	Double	Yes	35	27
Bianchi Porro 1982	1000 mg	w683	Peptic ulcers	Parallel	Pharmacy	Double	Yes	30	30
Buligescu 1985	1000 mg	w684	Peptic ulcers	Parallel	Unspecified method	Double	Yes	20	20
Gotthard 1982 (prepyloric ulcer)	1000 mg	w685	Peptic ulcers	Parallel	Unspecified method	Double	Yes	12	12
Gray 1977	1000 mg	w686	Peptic ulcers	Parallel	Unspecified method	Double	Yes	20	20
Gotthard 1982 (duodenal ulcer)	1000 mg	w685	Peptic ulcers	Parallel	Unspecified method	Double	Yes	18	18
Grewal 1979	1000 mg	w687	Peptic ulcers	Parallel	Unspecified method	Double	Yes	25	25
Marini 1981	1000 mg	w688	Peptic ulcers	Parallel	Unspecified method	Double	Yes	10	10
Gotlieb-Jensen 1983	1000 mg	w689	Peptic ulcers	Parallel	Unspecified method	Double	Yes	21	21
Kennedy 1978	1000 mg	w690	Peptic ulcers	Parallel	Unspecified method	Double	Yes	12	12
Giacosa 1982	1000 mg	w691	Peptic ulcers	Parallel	Unspecified method	Open	Yes	30	29
Monnis 1988	1000 mg	w692	Peptic ulcers	Parallel	Unspecified method	Double	Yes	12	11
Mukhtar 1982	1000 mg	w693	Peptic ulcers	Parallel	Unspecified method	Double	Yes	19	19
Guha Mazumdar 1981	1000 mg	w694	Peptic ulcers	Parallel	Unspecified method	Double	No	24	26
Popoola 1978 (duodenal ulcer)	1000 mg	w474	Peptic ulcers	Parallel	Unspecified method	Double	Yes	12	9

Webtable 3 (cont.): Details of trials involving a randomised comparison of a histamine-2 receptor antagonists (X) versus control (Y)

Trial name	Regimen	Ref.	Indication for treatment	Design	Allocation Concealment	Blinding of investigators and patients	Availability of data	No. of patients NX	NY
Sonnenberg 1981 X1	1000 mg	w403	Peptic ulcers	Extension	Closed list	Double	Yes	59	62
Manousos 1978 (gastric ulcer)	1000 mg	w695	Peptic ulcers	Parallel	Unspecified method	Double	Yes	9	9
Falaiye 1979	1000 mg	w696	Peptic ulcers	Parallel	Unspecified method	Double	Yes	16	11
Vantini 1978	1000 mg	w697	Peptic ulcers	Parallel	Unspecified method	Double	Yes	21	18
Tytgat 1982	1000 mg	w698	Peptic ulcers	Parallel	Other method	Double	Yes	30	24
Chen 1979	1000 mg	w699	Peptic ulcers	Parallel	Unspecified method	Double	Yes	46	40
Zespolowa 1980 (gastric ulcer)	1000 mg	w700	Peptic ulcers	Parallel	Pharmacy	Double	Yes	36	44
Villalobos 1978	1000 mg	w701	Peptic ulcers	Parallel	Unspecified method	Double	Yes	24	21
Bardhan 1979 (duodenal ulcer)	1000 mg	w702	Peptic ulcers	Parallel	Unspecified method	Double	Yes	78	50
Patty 1988	1000 mg	w703	Peptic ulcers	Parallel	Unspecified method	Double	Yes	18	19
Chen 1980 A	1000 mg	w704	Peptic ulcers	Parallel	Unspecified method	Double	Yes	25	25
Jensen 1979	1000 mg	w705	Peptic ulcers	Parallel	Unspecified method	Double	Yes	24	26
Manousos 1978 (duodenal ulcer)	1000 mg	w695	Peptic ulcers	Parallel	Unspecified method	Double	Yes	13	9
Gudmand-Höyer 1977	1000 mg	w706	Peptic ulcers	Parallel	Unspecified method	Unknown	Yes	30	30
Villalobos 1979	1000 mg	w707	Peptic ulcers	Parallel	Unspecified method	Double	Yes	10	10
Alcala-Santaella 1980 (gastric ulcer)	1000 mg	w708	Peptic ulcers	Parallel	Unspecified method	Double	Yes	8	6
Alcala-Santaella 1980 (duodenal ulcer)	1000 mg	w708	Peptic ulcers	Parallel	Unspecified method	Double	Yes	12	14
Guelrud 1978	1000 mg	w709	Peptic ulcers	Parallel	Unspecified method	Double	Yes	30	30
Matos-Villalobos 1979	1000 mg	w710	Peptic ulcers	Parallel	Unspecified method	Double	Yes	15	15
Gugler 1979	1000 mg	w711	Anastomotic ulcers	Parallel	Unspecified method	Double	Yes	7	8
Festen 1979	1000 mg	w712	Anastomotic ulcers	Parallel	Unspecified method	Double	Yes	12	9
Zespolowa 1980 (duodenal ulcer)	1000 mg	w700	Peptic ulcers	Parallel	Pharmacy	Double	Yes	66	63
O'Laughlin 1982	1200 mg	w713	Peptic ulcers	Parallel	Unspecified method	Double	Yes	9	9
Graham 1985	1200 mg	w714	Peptic ulcers	Parallel	Unspecified method	Unknown	Yes	87	85
Akdamar 1981	1200 mg	w715	Peptic ulcers	Parallel	Unspecified method	Unknown	Yes	65	67
Bodemar 1976	1200 mg	w659	Peptic ulcers	Parallel	Unspecified method	Double	No	15	15
Binder 1978 A	1200 mg	w716	Peptic ulcers	Parallel	Unspecified method	Double	Yes	45	53
Binder 1978 B	1200 mg	w716	Peptic ulcers	Parallel	Unspecified method	Double	Yes	30	30
Binder 1978 C	1200 mg	w716	Peptic ulcers	Parallel	Unspecified method	Double	Yes	41	37
Binder 1978 D	1200 mg	w716	Peptic ulcers	Parallel	Unspecified method	Double	Yes	36	36
Barbezat 1978	1200 mg	w717	Peptic ulcers	Parallel	Unspecified method	Double	No	6	7
Bank 1976	1200 mg	w718	Peptic ulcers	Parallel	Unspecified method	Double	Yes	8	19
Dölle1978	1200 mg	w719	Peptic ulcers	Parallel	Unspecified method	Double	Yes	33	34
Collen 1980 B	1200 mg	w720	Peptic ulcers	Parallel	Sealed envelopes	Double	Yes	11	12
Clarke 1980	1200 mg	w721	Peptic ulcers	Parallel	Unspecified method	Double	Yes	15	15
Van Deventer 1985	1200 mg	w722	Peptic ulcers	Parallel	Pharmacy	Double	No	21	20
Bodemar 1979 B	1200 mg	w442	Peptic ulcers	Parallel	Unspecified method	Double	Yes	15	14
Hetzel 1978 A	1200 mg	w443	Peptic ulcers	Parallel	Unspecified method	Double	Yes	43	42
Khanna 1989	1200 mg	w723	Peptic ulcers	Parallel	Unspecified method	Double	Yes	20	20
Collen 1980 A	1200 mg	w724	Peptic ulcers	Parallel	Sealed envelopes	Double	Yes	12	15
Englert 1978	1200 mg	w725	Peptic ulcers	Parallel	Other method	Double	Yes	60	62
Dyck 1978	1200 mg	w726	Peptic ulcers	Parallel	Unspecified method	Double	Yes	31	32
LoIudice 1981	1200 mg	w727	Peptic ulcers	Parallel	Unspecified method	Unknown	Yes	38	32
Moshal 1977	1200 mg	w663	Peptic ulcers	Parallel	Unspecified method	Double	Yes	17	19
Gaon 1981	1200 mg	w728	Peptic ulcers	Parallel	Unspecified method	Unknown	No	10	10
Isenberg 1983	1200 mg	w729	Peptic ulcers	Parallel	Other method	Double	Yes	45	46
Landecker 1979	1200 mg	w730	Peptic ulcers	Parallel	Unspecified method	Double	Yes	25	23
Sewing 1978	1200 mg	w731	Peptic ulcers	Parallel	Unspecified method	Double	Yes	20	20
Braverman 1986	1600 mg	w655	Peptic ulcers	Parallel	Unspecified method	Double	Yes	188	168
Barbezat 1978	1600 mg	w717	Peptic ulcers	Parallel	Unspecified method	Double	No	10	7
Bank 1976	1600 mg	w718	Peptic ulcers	Parallel	Unspecified method	Double	Yes	11	19

Webtable 3 (cont.): Details of trials involving a randomised comparison of a histamine-2 receptor antagonists (X) versus control (Y)

Trial name	Regimen	Ref.	Indication for treatment	Design	Allocation Concealment	Blinding of investigators and patients	Availability of data	No. of patients NX	NY
Davies 1986 (gastric ulcer)	1600 mg	w732	Peptic ulcers	Parallel	Unspecified method	Double	Yes	11	10
Blackwood 1976	1600 mg	w733	Peptic ulcers	Parallel	Unspecified method	Double	No	-	-
Young 1989 A	1600 mg	w656	Peptic ulcers	Parallel	Unspecified method	Double	Yes	188	168
Davies 1986 (duodenal ulcer)	1600 mg	w732	Peptic ulcers	Parallel	Unspecified method	Double	Yes	3	3
Bardhan 1979 (duodenal ulcer)	2000 mg	w702	Peptic ulcers	Parallel	Unspecified method	Double	Yes	72	50
Machado 1978	Unknown	w734	Peptic ulcers	Parallel	Unspecified method	Double	Yes	18	17
Famotidine vs Control									
Simon 1987 B	40 mg	w735	Peptic ulcers	Parallel	Unspecified method	Double	Yes	149	145
Dammann 1985 A	40 mg	w736	Peptic ulcers	Parallel	Unspecified method	Double	Yes	34	39
McCullough 1989	40 mg	w737	Peptic ulcers	Parallel	Unspecified method	Double	Yes	79	78
Dammann 1987 B	40 mg	w738	Peptic ulcers	Parallel	Other method	Double	Yes	167	169
Gitlin 1987	40 mg	w739	Peptic ulcers	Parallel	Telephone	Double	Yes	96	100
Gitlin 1987	40 mg	w739	Peptic ulcers	Parallel	Telephone	Double	Yes	89	100
Dammann 1985 B	40 mg	w740	Peptic ulcers	Parallel	Unspecified method	Double	Yes	71	73
Gitlin 1987	80 mg	w739	Peptic ulcers	Parallel	Telephone	Double	Yes	99	100
Nizatidine vs Control									
Dyck 1987 X1	50 mg	w741	Peptic ulcers	Extension	Unspecified method	Double	Yes	103	101
Cloud 1989 A	100 mg	w742	Peptic ulcers	Parallel	Unspecified method	Double	Yes	94	98
Cloud 1992 B	300 mg	w743	Peptic ulcers	Parallel	Other method	Double	Yes	153	152
Cloud 1992 B	300 mg	w743	Peptic ulcers	Parallel	Other method	Double	Yes	151	152
Eliakim 1994	300 mg	w744	Peptic ulcers	Parallel	Unspecified method	Double	Yes	27	28
Cloud 1989 A	300 mg	w742	Peptic ulcers	Parallel	Unspecified method	Double	Yes	92	98
Levendoglu 1986	300 mg	w745	Peptic ulcers	Parallel	Unspecified method	Double	Yes	23	19
Dyck 1987 X2	300 mg	w741	Peptic ulcers	Extension	Unspecified method	Double	Yes	84	82
Dyck 1987 X1	300 mg	w741	Peptic ulcers	Extension	Unspecified method	Double	Yes	106	101
Dyck 1987 X1	300 mg	w741	Peptic ulcers	Extension	Unspecified method	Double	Yes	108	101
Cloud 1989 B X1	300 mg	w549	Peptic ulcers	Extension	Unspecified method	Double	Yes	276	279
Cloud 1989 B X2	300 mg	w549	Peptic ulcers	Extension	Unspecified method	Double	Yes	28	26
Cloud 1989 B X3	300 mg	w549	Peptic ulcers	Extension	Unspecified method	Double	Yes	66	72
Oxmetidine vs Control									
Lam 1985 B	600 mg	w746	Peptic ulcers	Parallel	Unspecified method	Double	Yes	40	38
Ranitidine vs Control									
Forssell 1983 (gastric ulcer)	200 mg	w747	Peptic ulcers	Parallel	Unspecified method	Double	No	15	14
Dobrilla 1981 A	200 mg	w748	Peptic ulcers	Parallel	Other method	Double	Yes	20	20
Forssell 1983 (duodenal ulcer)	200 mg	w747	Peptic ulcers	Parallel	Unspecified method	Double	Yes	9	10
Berstad 1980	200 mg	w749	Peptic ulcers	Parallel	Unspecified method	Double	Yes	25	25
Darle 1984 B (gastric ulcer + prepyloric ulcer)	200 mg	w750	Peptic ulcers	Parallel	Other method	Double	Yes	9	13
Darle 1984 B (duodenal ulcer)	200 mg	w751	Peptic ulcers	Parallel	Other method	Double	Yes	16	12
Pan 1987	300 mg	w752	Peptic ulcers	Parallel	Unspecified method	Double	Yes	27	28
Martinez 1988	300 mg	w753	Peptic ulcers	Parallel	Unspecified method	Double	No	10	10
Fica 1987	300 mg	w670	Peptic ulcers	Parallel	Unspecified method	Unknown	Yes	20	20
Schulz 1984	300 mg	w754	Peptic ulcers	Parallel	Unspecified method	Double	Yes	25	20
Hirschowitz 1986 A	300 mg	w755	Peptic ulcers	Parallel	Unspecified method	Double	Yes	103	103
Marks 1982	300 mg	w756	Peptic ulcers	Parallel	Closed list	Double	Yes	40	40
Lee 1982	300 mg	w757	Peptic ulcers	Parallel	Closed list	Double	Yes	62	67
Hirschowitz 1986 B X1	300 mg	w758	Peptic ulcers	Extension	Unspecified method	Double	Yes	195	187
Hirschowitz 1986 B X2	300 mg	w758	Peptic ulcers	Extension	Unspecified method	Double	Yes	57	67
Guo-zong 1988	300 mg	w759	Peptic ulcers	Parallel	Unspecified method	Double	Yes	10	13
Hirschowitz 1983 X1	300 mg	w760	Peptic ulcers	Extension	Unspecified method	Unknown	Yes	195	187
Hirschowitz 1983 X2	300 mg	w760	Peptic ulcers	Extension	Unspecified method	Unknown	Yes	58	66
Chatterji 1980	300 mg	w761	Peptic ulcers	Parallel	Unspecified method	Double	Yes	15	15
Ashton 1982	300 mg	w762	Peptic ulcers	Parallel	Unspecified method	Double	Yes	22	20

Webtable 3 (cont.): Details of trials involving a randomised comparison of a histamine-2 receptor antagonists (X) versus control (Y)

Trial name	Regimen	Ref.	Indication for treatment	Design	Allocation Concealment	Blinding of investigators and patients	Availability of data	No. of patients NX	NY
Dobrilla 1981 B	300 mg	w763	Peptic ulcers	Parallel	Unspecified method	Double	Yes	63	59
Dawson 1984 X1	300 mg	w564	Peptic ulcers	Extension	Unspecified method	Single	Yes	18	20
Gibinski 1982 (gastric ulcer)	300 mg	w764	Peptic ulcers	Parallel	Unspecified method	Double	Yes	40	41
Gibinski 1982 (duodenal ulcer)	300 mg	w764	Peptic ulcers	Parallel	Unspecified method	Double	Yes	90	87
Jhala 1995	300 mg	w233	Peptic ulcers	Parallel	Unspecified method	Double	Yes	33	19
Nair 1990	300 mg	w765	Peptic ulcers	Parallel	Unspecified method	Double	Yes	28	32
Nelis 1982	300 mg	w766	Peptic ulcers	Parallel	Other method	Double	Yes	14	13
Giacosa 1982	300 mg	w691	Peptic ulcers	Parallel	Unspecified method	Open	Yes	31	29
Moshal 1981	300 mg	w767	Peptic ulcers	Parallel	Unspecified method	Double	Yes	25	25
Murray 1986	300 mg	w768	Peptic ulcers	Parallel	Unspecified method	Double	Yes	20	20
Pasquali 1981	300 mg	w769	Peptic ulcers	Parallel	Unspecified method	Double	Yes	15	15
Johnson 1993	300 mg	w770	Peptic ulcers	Parallel	Unspecified method	Double	Yes	152	116
Tildesley 1993 (gastric ulcer)	300 mg	w771	Peptic ulcers	Parallel	Other method	Double	Yes	28	15
Tildesley 1993 (duodenal ulcer)	300 mg	w771	Peptic ulcers	Parallel	Other method	Double	Yes	36	12
Korman 1982	300 mg	w772	Peptic ulcers	Parallel	Unspecified method	Double	Yes	25	25
Makalinao 1984 (gastric ulcer)	300 mg	w773	Peptic ulcers	Parallel	Unspecified method	Double	Yes	21	23
Leroux 1983 (gastric ulcer)	300 mg	w774	Peptic ulcers	Parallel	Unspecified method	Double	Yes	17	13
Mach 1995 B	300 mg	w775	Peptic ulcers	Parallel	Unspecified method	Open	Yes	21	18
Mach 1995 A	300 mg	w776	Peptic ulcers	Parallel	Unspecified method	Double	Yes	46	38
Yap 1985	300 mg	w777	Peptic ulcers	Parallel	Other method	Double	Yes	23	24
Roberts 1982	300 mg	w778	Peptic ulcers	Parallel	Unspecified method	Double	Yes	39	41
Mach 1992	300 mg	w779	Peptic ulcers	Parallel	Unspecified method	Double	Yes	30	26
Dupas 2000	300 mg	w780	Peptic ulcers	Parallel	Unspecified method	Double	Yes	180	163
Mach 1991	300 mg	w781	Peptic ulcers	Unknown	Unspecified method	Unknown	No	24	20
Lanza 1994	300 mg	w234	Peptic ulcers	Parallel	Unspecified method	Double	Yes	86	44
Makalinao 1984 (duodenal ulcer)	300 mg	w773	Peptic ulcers	Parallel	Unspecified method	Double	Yes	24	27
Leroux 1983 (duodenal ulcer)	300 mg	w774	Peptic ulcers	Parallel	Unspecified method	Double	Yes	20	21
Elizalde 1983 (duodenal ulcer)	300 mg	w624	Peptic ulcers	Parallel	Unspecified method	Double	Yes	20	15
Elizalde 1983 (gastric and duodenal ulcer)	300 mg	w624	Peptic ulcers	Parallel	Unspecified method	Double	Yes	3	5
Elizalde 1983 (gastric ulcer)	300 mg	w624	Peptic ulcers	Parallel	Unspecified method	Double	No	-	-
Robins 1984	300 mg	w782	Peptic ulcers	Parallel	Unspecified method	Double	Yes	25	28
Sheers 1982	400 mg	w783	Peptic ulcers	Parallel	Unspecified method	Double	Yes	25	23
Massarrat 1998	600 mg	w784	Peptic ulcers	Parallel	Unspecified method	Double	Yes	78	78
Tefera 1996 (no metronizadole)	600 mg	w647	Peptic ulcers	Parallel	Closed list	Double	Yes	34	39
Tefera 1996 (metronizadole)	600 mg	w647	Peptic ulcers	Parallel	Closed list	Double	Yes	36	30
Lanza 1998	800 mg	w785	Peptic ulcers	Parallel	Unspecified method	Double	No	115	89
Trautman 1981	Unknown	w786	Peptic ulcers	Parallel	Unspecified method	Double	No	-	-
Witzel 1981	Unknown	w787	Peptic ulcers	Parallel	Unspecified method	Double	No	25	25
Roxatidine vs Control									
Gilinsky 1992	150 mg	w788	Peptic ulcers	Parallel	Unspecified method	Double	Yes	177	179

Acute Bleeding Trials

Trial name	Regimen	Ref.	Indication for treatment	Design	Allocation Concealment	Blinding of investigators and patients	Availability of data	NX	NY
Cimetidine vs Control									
Darle 1984 A	800 mg	w789	Bleeding Peptic Ulcer	Parallel	Unspecified method	Double	Yes	33	39
Siddiqi 1979	800 mg	w790	UGI bleeding of any cause	Parallel	Unspecified method	Open	Yes	58	55
Stiel 1984	800 mg	w791	Bleeding Peptic Ulcer	Parallel	Unspecified method	Double	Yes	29	26
Zuckerman 1984	900 mg	w792	UGI bleeding of any cause	Extension	Onsite computer	Double	Yes	153	132
Terés 1980 X2	1000 mg	w793	UGI bleeding of any cause	Extension	Unspecified method	Double	Yes	20	20
Carstensen 1980	1200 mg	w794	Bleeding Peptic Ulcer	Parallel	Closed list	Double	Yes	40	48
Hoare 1979	1200 mg	w795	Bleeding Peptic Ulcer	Parallel	Closed list	Double	Yes	38	33
Terés 1980 X1	1200 mg	w796	UGI bleeding of any cause	Extension	Pharmacy	Double	Yes	20	20
Olivero 1981	1200 mg	w797	Bleeding Peptic Ulcer	Parallel	Unspecified method	Double	No	20	20

Webtable 3 (cont.): Details of trials involving a randomised comparison of a histamine-2 receptor antagonists (X) versus control (Y)

Trial name	Regimen	Ref.	Indication for treatment	Design	Allocation Concealment	Blinding of investigators and patients	Availability of data	No. of patients NX	NY
Baltas 1984	1200 mg	w798	UGI bleeding of any cause	Parallel	Other method	Double	Yes	36	36
Kang 1977	1200 mg	w799	UGI bleeding of any cause	Parallel	Unspecified method	Double	Yes	32	33
Stadler 1985 (pirenzipine)	1200 mg	w800	Bleeding Peptic Ulcer	Parallel	Unspecified method	Unknown	Yes	18	20
Barer 1983	1600 mg	w801	UGI bleeding of any cause	Parallel	Pharmacy	Double	Yes	259	260
Galmiche 1978	1600 mg	w802	Bleeding Peptic Ulcer	Parallel	Unspecified method	Double	Yes	24	24
Birnie 1984	1600 mg	w803	UGI bleeding of any cause	Parallel	Unspecified method	Double	Yes	106	107
La Brooy 1979	1600 mg	w804	UGI bleeding of any cause	Parallel	Unspecified method	Double	Yes	51	50
Famotidine vs Control									
Fullarton 1991 B	70.4 mg	w805	Bleeding Peptic Ulcer	Parallel	Unspecified method	Double	Yes	10	10
Walt 1992	76.8 mg	w806	Bleeding Peptic Ulcer	Parallel	Other method	Double	Yes	497	508
Ranitidine vs Control									
Gray 1986	150 mg	w807	Bleeding Peptic Ulcer	Parallel	Unspecified method	Double	Yes	50	52
Ermolov 1995	150 mg	w808	Bleeding Peptic Ulcer	Parallel	Unspecified method	Unknown	Yes	31	33
Stadler 1985 (pirenzipine)	150 mg	w800	Bleeding Peptic Ulcer	Parallel	Unspecified method	Unknown	Yes	20	20
Coraggio 1984	300 mg	w809	UGI bleeding of any cause	Parallel	Unspecified method	Unknown	Yes	20	20
Basso 1986	300 mg	w810	UGI bleeding of any cause	Parallel	Unspecified method	Double	Yes	33	33
Stave 1986	300 mg	w811	Bleeding Peptic Ulcer	Parallel	Unspecified method	Double	No	32	34
Dawson 1982	450 mg	w812	UGI bleeding of any cause	Parallel	Unspecified method	Double	Yes	76	75

Design: Parallel=parallel trial. Factorial=Factorial trial. ‘Extension’ studies are studies in which patients are randomised more than once during the trial (only the parts of the trial that involve a valid randomised comparison for this meta-analysis, labelled X1, X2 etc., are shown in this table). Data shown for crossover trials reflect only events during the first phase of the trial. The suffixes A, B, C, D are used to distinguish different trials published by the same author in the same year.

Allocation concealment: IVRS (Interactive Voice Response System); Telephone (telephone central office); Pharmacy (pharmacy coded containers administered sequentially); Onsite computer (onsite computer accessible only after entering patient details); Sealed envelopes (sequentially numbered, opaque or without further specification); Closed list (closed list of random numbers); Other (other specified method); Unspecified (stated as being ‘randomised’ but with no other details).

Blinding of investigators and patients: Single=single-blind; Double=double-blind; Open=open label.

Availability of data: Data available for at least one endpoint of interest in the meta-analyses.

In cases where the number randomised was not reported, the number evaluated was used instead. In addition, for crossover trials, where neither the number randomised nor the number evaluated was reported, the total number of participants divided by number of arms was used.

Webtable 4: Details of trials involving a randomised comparison of a proton pump inhibitor (X) versus a prostaglandin analogue (Y)

Trial name	Regimen	Ref.	Indication for treatment	Design	Allocation Concealment	Blinding of investigators and patients	Availability of data	No. of patients	
								NX	NY
**Prevention Trials**									
Lansoprazole vs Misoprostol									
Graham 2002	15 mg vs 800 ug	w51	Recently healed ulcers	Parallel	Other method	Double	Yes	136	134
Graham 2002	30 mg vs 800 ug	w51	Recently healed ulcers	Parallel	Other method	Double	Yes	133	134
Lansoprazole vs Rebamipide									
Murakami 2003	30 mg vs 300 mg	w813	Gastritis or duodenitis	Crossover	Unspecified method	Unknown	No	8	8
Omeprazole vs Misoprostol									
OMNIUM 1998 X2	20 mg vs 400 ug	w113	Recently healed ulcers	Extension	Unspecified method	Double	Yes	274	296
OMNIUM 1998 X1 (erosions)	20 mg vs 800 ug	w113	Antiplatelet therapy	Extension	Unspecified method	Double	No	118	97
OMNIUM 1998 X1 (erosions)	40 mg vs 800 ug	w113	Antiplatelet therapy	Extension	Unspecified method	Double	No	109	97
Omeprazole vs Rebamipide									
Thong-Ngam 2010	20 mg vs 300 mg	w814	Gastro-oesophageal reflux	Parallel	Unspecified method	Unknown	Yes	25	18
Kojima 2012	Unknown vs Unknown	w815	Healthy volunteers	Parallel	Unspecified method	Unknown	No	-	-
Kojima 2012	Unknown vs Unknown	w815	Healthy volunteers	Parallel	Unspecified method	Unknown	No	-	-
Pantoprazole vs Misoprostol									
Stupnicki 2003	20 mg vs 400 ug	w816	Arthritis, rheumatic or musculoskeletal disorders	Parallel	Closed list	Double	Yes	257	258
Rabeprazole vs Rebamipide									
Thong-Ngam 2010	20 mg vs 300 mg	w814	Gastro-oesophageal reflux	Parallel	Unspecified method	Unknown	Yes	13	18
**Healing Trials**									
Lansoprazole vs Rebamipide						Unspecified method			
Takayama 2012	60 mg vs 600 mg	w817	Endoscopic submucosal dissection	Parallel	Unspecified method	Unknown	Yes	31	29
Matsui 2012	60 mg vs 600 mg	w818	Endoscopic submucosal dissection	Parallel	Unspecified method	Unknown	Yes	27	27
Nakamura 1995	120 mg vs 900 mg	w819	Peptic ulcers	Parallel	Unspecified method	Unknown	Yes	5	5
Omeprazole vs Misoprostol									
OMNIUM 1998 X1 GU+DU	20 mg vs 800 ug	w113	Peptic ulcers	Extension	Unspecified method	Double	Yes	190	201
OMNIUM 1998 X1 GU+DU	40 mg vs 800 ug	w113	Peptic ulcers	Extension	Unspecified method	Double	Yes	205	201
Omeprazole vs Rebamipide									
Song 2011	20 mg vs 300 mg	w820	Peptic ulcers	Parallel	Unspecified method	Double	Yes	63	65

DU=duodenal ulcer; GU=gastric ulcer.

Design: Parallel=parallel trial. Factorial=Factorial trial. ‘Extension’ studies are studies in which patients are randomised more than once during the trial (only the parts of the trial that involve a valid randomised comparison for this meta-analysis, labelled X1, X2 etc., are shown in this table). Data shown for crossover trials reflect only events during the first phase of the trial. The suffixes A, B, C, D are used to distinguish different trials published by the same author in the same year.

Allocation concealment: IVRS (Interactive Voice Response System); Telephone (telephone central office); Pharmacy (pharmacy coded containers administered sequentially); Onsite computer (onsite computer accessible only after entering patient details); Sealed envelopes (sequentially numbered, opaque or without further specification); Closed list (closed list of random numbers); Other (other specified method); Unspecified (stated as being ‘randomised’ but with no other details).

Blinding of investigators and patients: Single=single-blind; Double=double-blind; Open=open label.

Availability of data: Data available for at least one endpoint of interest in the meta-analyses.

In cases where the number randomised was not reported, the number evaluated was used instead. In addition, for crossover trials, where neither the number randomised nor the number evaluated was reported, the total number of participants divided by number of arms was used.

Webtable 5: Details of trials involving a randomised comparison of a proton pump inhibitor (X) versus a histamine-2 receptor antagonist (Y)

Trial name	Regimen	Ref.	Indication for treatment	Design	Allocation Concealment	Blinding of investigators and patients	Availability of data	No. of patients NX	NY
**Prevention Trials**									
Esomeprazole vs Famotidine									
Tunggal 2011	20 mg vs 40 mg	w821	Acute coronary syndrome	Parallel	Sealed envelopes	Double	Yes	55	52
Ng 2012	20 mg vs 40 mg	w822	Acute coronary syndrome	Parallel	Sealed envelopes	Double	Yes	164	149
Esomeprazole vs Ranitidine									
Norman Hansen 2005	20 mg vs 300 mg	w823	Gastro-oesophageal reflux	Parallel	Onsite computer	Open	No	658	610
Hansen 2006	20 mg vs 300 mg	w824	Gastro-oesophageal reflux	Parallel	Unspecified method	Open	No	658	610
Kilic 2008 (amoxycillin+moxifloxacine)	80 mg vs 800 mg	w825	Gastritis or duodenitis	Parallel	Unspecified method	Open	Yes	30	30
Kilic 2008 (amoxycillin+clarithromycin)	80 mg vs 800 mg	w825	Gastritis or duodenitis	Parallel	Unspecified method	Open	Yes	30	30
Nadeau 2012	Unknown vs Unknown	w826	Percutaneous coronary intervention	Parallel	Unspecified method	Unknown	No	-	-
Lansoprazole vs Famotidine									
Miyake 2005	15 mg vs 40 mg	w827	Rheumatoid arthritis	Parallel	Sealed envelopes	Unknown	Yes	13	13
Endo 2000	15 mg vs 40 mg	w828	Gastro-oesophageal reflux	Parallel	Unspecified method	Double	No	23	25
Endo 2000	30 mg vs 40 mg	w828	Gastro-oesophageal reflux	Parallel	Unspecified method	Double	No	22	25
Mine 2000	30 mg vs 40 mg	w829	Gastro-oesophageal reflux	Crossover	Unspecified method	Double	No	9	8
Lansoprazole vs Lafutidine									
Kim 2008	60 mg vs 40 mg	w830	Gastritis or duodenitis	Parallel	Unspecified method	Double	Yes	112	119
Lansoprazole vs Nizatidine									
Murakami 2003	30 mg vs 300 mg	w813	Gastritis or duodenitis	Crossover	Unspecified method	Unknown	No	8	8
Lansoprazole vs Ranitidine									
Petite 1991 B	15 mg vs 300 mg	w831	Gastro-oesophageal reflux	Parallel	Unspecified method	Double	Yes	63	55
Bardhan 1999 A X2	15 mg vs 150 mg	w832	Recently healed ulcers	Extension	Unspecified method	Double	Yes	121	116
Müller 1997	15 mg vs 300 mg	w58	Healthy volunteers	Parallel	Unspecified method	Double	No	10	10
Meining 1998	15 mg vs 150 mg	w833	Gastritis or duodenitis	Parallel	Unspecified method	Unknown	No	72	71
Gough 1996	15 mg vs 600 mg	w834	Gastro-oesophageal reflux	Parallel	Unspecified method	Double	Yes	96	81
Peura 2009 B	15 mg vs 300 mg	w835	Gastro-oesophageal reflux	Parallel	Unspecified method	Double	No	100	106
Richter 2000 C	15 mg vs 300 mg	w67	Gastro-oesophageal reflux	Parallel	Unspecified method	Double	No	284	283
Spadaccini 1998 (healed duodenal ulcer)	15 mg vs 800 ug	w836	Recently healed ulcers	Parallel	Unspecified method	Open	No	18	13
Spadaccini 1998 (healed gastric ulcer)	15 mg vs 800 mg	w836	Recently healed ulcers	Parallel	Unspecified method	Open	Yes	2	2
Spadaccini 1998	15 mg vs 800 mg	w836	Gastritis and/or duodenitis	Parallel	Unspecified method	Open	No	18	25
Petite 1991 B	30 mg vs 300 mg	w831	Gastro-oesophageal reflux	Parallel	Unspecified method	Double	Yes	59	55
Plein 1997	30 mg vs 300 mg	w837	Gastro-oesophageal reflux	Parallel	Onsite computer	Double	No	107	58
Bardhan 1999 A X2	30 mg vs 150 mg	w832	Recently healed ulcers	Extension	Unspecified method	Double	Yes	122	116
Robinson 1995 A	30 mg vs 300 mg	w838	Gastro-oesophageal reflux	Parallel	Unspecified method	Double	Yes	119	128
Bardhan 1995 A	30 mg vs 300 mg	w839	Gastro-oesophageal reflux	Parallel	Closed list	Double	Yes	77	77
Feldman 1993	30 mg vs 300 mg	w840	Gastro-oesophageal reflux	Parallel	Unspecified method	Double	Yes	62	33
Jansen 1999	30 mg vs 600 mg	w841	Gastro-oesophageal reflux	Parallel	Unspecified method	Double	No	68	65
Jones 1997	30 mg vs 300 mg	w842	Gastro-oesophageal reflux	Parallel	Other method	Double	No	213	219
Howden 2001	30 mg vs 300 mg	w843	Gastro-oesophageal reflux	Parallel	Sealed envelopes	Double	No	297	296
Gough 1996	30 mg vs 600 mg	w834	Gastro-oesophageal reflux	Parallel	Unspecified method	Double	Yes	89	81
Sontag 1997 B X1	30 mg vs 300 mg	w844	Gastro-oesophageal reflux	Parallel	Unspecified method	Double	No	69	36
Richter 2000 C	30 mg vs 300 mg	w67	Gastro-oesophageal reflux	Parallel	Unspecified method	Double	No	288	283
Sontag 1997	30 mg vs 300 mg	w845	Gastro-oesophageal reflux	Parallel	Unspecified method	Double	No	105	54
Swarbrick 1996	30 mg vs 600 mg	w846	Oesophageal stricture	Parallel	Pharmacy	Double	Yes	78	80
Bardhan 1995 A	60 mg vs 300 mg	w839	Gastro-oesophageal reflux	Parallel	Closed list	Double	Yes	75	77
Treiber 1997	60 mg vs 600 mg	w847	Gastritis or duodenitis	Parallel	Unspecified method	Single	No	20	20
Lansoprazole vs Roxatidine									
Shimizu 2006	30 mg vs 150 mg	w848	Asthma	Parallel	Unspecified method	Double	Yes	15	15
Omeprazole vs Cimetidine									
Bate 1998	10 mg vs 800 mg	w849	Gastro-oesophageal reflux	Parallel	Unspecified method	Double	Yes	77	79

Webtable 5 (cont.): Details of trials involving a randomised comparison of a proton pump inhibitor (X) versus histamine-2 receptor antagonist (Y)

Trial name	Regimen	Ref.	Indication for treatment	Design	Allocation Concealment	Blinding of investigators and patients	Availability of data	No. of patients NX	NY
Bate 1990	20 mg vs 1600 mg	w850	Gastro-oesophageal reflux	Parallel	Unspecified method	Double	Yes	138	134
Meineche-Schmidt 1997 (dyspepsia)	20 mg vs 800 mg	w140	Gastro-oesophageal reflux	Parallel	Unspecified method	Double	No	225	244
Thorens 1996	20 mg vs 800 mg	w851	Gastro-oesophageal reflux	Parallel	Unspecified method	Double	No	19	18
Dehn 1990	40 mg vs 1600 mg	w852	Gastro-oesophageal reflux	Parallel	Unspecified method	Double	No	31	36
Bate 1997	1600 mg vs 400 mg	w853	Gastro-oesophageal reflux	Parallel	Unspecified method	Double	Yes	112	109
Omeprazole vs Famotidine									
Shimatani 2007	10 mg vs 40 mg	w854	Healthy volunteers	Crossover	Unspecified method	Unknown	No	4	4
Yano 2012	10 mg vs 20 mg	w855	Acute coronary syndrome	Parallel	Other method	Open	Yes	90	90
J-FOCUS	10 mg vs 20 mg	w856	Gastro-oesophageal reflux	Parallel	Closed list	Open	No	142	132
Fujiwara 2005	20 mg vs 40 mg	w857	Gastro-oesophageal reflux	Parallel	Unspecified method	Unknown	Yes	53	53
Wada 2005	20 mg vs 40 mg	w858	Gastro-oesophageal reflux	Parallel	Unspecified method	Open	No	27	27
Hu 2003	20 mg vs 20 mg	w859	Gastritis or duodenitis	Parallel	Unspecified method	Unknown	Yes	39	38
Kawano 2002	20 mg vs 40 mg	w860	Gastro-oesophageal reflux	Parallel	Sealed envelopes	Unknown	Yes	29	27
Miner 2006 A	20 mg vs 40 mg	w861	Healthy volunteers	Crossover	Unspecified method	Double	Yes	11	11
Miner 2006 A	20 mg vs 20 mg	w861	Healthy volunteers	Crossover	Unspecified method	Double	Yes	11	11
Soga 1999	20 mg vs 40 mg	w862	Gastro-oesophageal reflux	Parallel	Sealed envelopes	Unknown	No	36	35
Guslandi 1994	20 mg vs 40 mg	w863	Gastro-oesophageal reflux	Parallel	Unspecified method	Unknown	Yes	6	6
Kim 2015	20 mg vs 40 mg	w864	Gastro-oesophageal reflux	Parallel	Unspecified method	Double	Yes	167	162
Arbel 2013	40 mg vs 80 mg	w865	Antiplatelet therapy	Crossover	Sealed envelopes	Single	Yes	21	21
Omeprazole vs other H2RA									
Marks 1994	20 mg vs Unknown	w866	Gastro-oesophageal reflux	Parallel	Unspecified method	Open	Yes	19	18
Omeprazole vs Ranitidine									
Kim 2004	10 mg vs 300 mg	w867	Gastro-oesophageal reflux	Parallel	Unspecified method	Unknown	No	50	50
Hallerback 1994	10 mg vs 300 mg	w868	Gastro-oesophageal reflux	Parallel	Unspecified method	Double	Yes	133	128
Blum 2000	10 mg vs 150 mg	w104	Gastro-oesophageal reflux	Parallel	Unspecified method	Double	Yes	202	194
Karnes 1990	10 mg vs 300 mg	w869	Healthy volunteers	Parallel	Closed list	Double	No	12	12
Bardhan 1999 B	10 mg vs 300 mg	w870	Gastro-oesophageal reflux	Parallel	Onsite computer	Double	No	227	229
Jaspersen 1999	10 mg vs 300 mg	w871	Gastro-oesophageal reflux	Parallel	Unspecified method	Unknown	No	10	10
Lauritsen 1999	10 mg vs 150 mg	w872	Recently healed ulcers	Parallel	Closed list	Double	Yes	308	312
Venables 1997 A	10 mg vs 300 mg	w873	Gastro-oesophageal reflux	Parallel	Unspecified method	Double	Yes	338	326
ASTRONAUT X2	20 mg vs 300 mg	w874	Recently healed ulcers	Extension	Unspecified method	Double	Yes	210	215
Zeitoun 1989	20 mg vs 300 mg	w875	Gastro-oesophageal reflux	Parallel	Unspecified method	Double	Yes	76	80
Kaplan-Machlis 2000	20 mg vs 300 mg	w876	Gastro-oesophageal reflux	Parallel	Sealed envelopes	Open	No	138	130
Díaz de Rojas 1998	20 mg vs 150 mg	w877	Recently healed ulcers	Parallel	Unspecified method	Double	Yes	215	219
Annibale 1998	20 mg vs 300 mg	w878	Gastro-oesophageal reflux	Parallel	Unspecified method	Double	No	106	111
Maton 1999	20 mg vs 300 mg	w879	Gastro-oesophageal reflux	Parallel	Unspecified method	Double	Yes	156	161
Angelini 1993	20 mg vs 300 mg	w880	Gastro-oesophageal reflux	Parallel	Unspecified method	Double	No	51	51
Hungin 1993	20 mg vs 300 mg	w881	Gastro-oesophageal reflux	Parallel	Unspecified method	Open	Yes	215	209
Di Mario 1994	20 mg vs 150 mg	w882	Recently healed ulcers	Parallel	Unspecified method	Open	Yes	30	12
Armstrong 2005	20 mg vs 300 mg	w883	Gastro-oesophageal reflux	Parallel	Onsite computer	Double	Yes	196	194
Bianchi Porro 1992 B	20 mg vs 300 mg	w884	Gastro-oesophageal reflux	Parallel	Unspecified method	Double	No	30	30
Frame 1991	20 mg vs 300 mg	w885	Gastro-oesophageal reflux	Parallel	Unspecified method	Double	Yes	86	86
Festen1999	20 mg vs 600 mg	w886	Gastro-oesophageal reflux	Parallel	Unspecified method	Double	No	222	224
Hallerback 1994	20 mg vs 300 mg	w868	Gastro-oesophageal reflux	Parallel	Unspecified method	Double	Yes	131	128
Blum 2000	20 mg vs 150 mg	w104	Gastro-oesophageal reflux	Parallel	Unspecified method	Double	Yes	193	194
Karnes 1990	20 mg vs 300 mg	w869	Healthy volunteers	Parallel	Closed list	Double	No	12	12
Savarino 2000	20 mg vs 300 mg	w887	Gastro-oesophageal reflux	Parallel	Closed list	Open	Yes	30	30
Bardhan 1999 B	20 mg vs 300 mg	w870	Gastro-oesophageal reflux	Parallel	Onsite computer	Double	No	221	229
Sartori 2000	20 mg vs 300 mg	w131	Cancer or palliative care (non gastrointestinal)	Parallel	Unspecified method	Unknown	Yes	76	77
Smith 1994	20 mg vs 150 mg	w888	Oesophageal stricture	Parallel	Unspecified method	Double	No	180	185

Webtable 5 (cont.): Details of trials involving a randomised comparison of a proton pump inhibitor (X) versus histamine-2 receptor antagonist (Y)

Trial name	Regimen	Ref.	Indication for treatment	Design	Allocation Concealment	Blinding of investigators and patients	Availability of data	No. of patients NX	NY
Zeitoun 1987	20 mg vs 300 mg	w889	Gastro-oesophageal reflux	Parallel	Unspecified method	Double	No	76	80
Tham 1996	20 mg vs 1200 mg	w890	Gastro-oesophageal reflux	Parallel	Unspecified method	Single	No	20	20
Richter 1996	20 mg vs 300 mg	w891	Gastro-oesophageal reflux	Parallel	Unspecified method	Open	Yes	100	97
Revicki 1998	20 mg vs 300 mg	w892	Gastro-oesophageal reflux	Parallel	Unspecified method	Double	Yes	156	161
Dent 1994	20 mg vs 300 mg	w893	Gastro-oesophageal reflux	Parallel	Closed list	Double	Yes	53	51
Vigneri 1995	20 mg vs 450 mg	w894	Gastro-oesophageal reflux	Parallel	Closed list	Single	No	70	70
Sandmark 1988	20 mg vs 300 mg	w895	Gastro-oesophageal reflux	Parallel	Onsite computer	Double	Yes	75	77
Lauritsen 1999	20 mg vs 150 mg	w872	Recently healed ulcers	Parallel	Closed list	Double	Yes	308	312
Venables 1997 A	20 mg vs 300 mg	w873	Gastro-oesophageal reflux	Parallel	Unspecified method	Double	Yes	330	326
Figura 1995	20 mg vs 150 mg	w896	Recently healed ulcers	Parallel	Unspecified method	Unknown	Yes	14	13
Cavaljuga 2003	20 mg vs 150 mg	w897	Gastritis or duodenitis	Parallel	Unspecified method	Unknown	No	-	-
Bianchi Porro 1991 B	20 mg vs 300 mg	w898	Gastro-oesophageal reflux	Parallel	Unspecified method	Double	Yes	27	17
Ruth 1988	20 mg vs 300 mg	w899	Gastro-oesophageal reflux	Parallel	Unspecified method	Double	Yes	12	10
Bate 1991	20 mg vs 300 mg	w900	Gastro-oesophageal reflux	Parallel	Unspecified method	Double	Yes	143	140
Archambault 1996	20 mg vs 450 mg	w901	Gastro-oesophageal reflux	Parallel	Closed list	Single	No	1001	480
Silvis 1996	20 mg vs 300 mg	w902	Oesophageal stricture	Parallel	Unspecified method	Unknown	No	15	18
Houben 1995	20 mg vs 600 mg	w141	Healthy volunteers	Crossover	Unspecified method	Double	No	3	3
Green 1995	35 mg vs 525 mg	w903	Gastro-oesophageal reflux	Parallel	Unspecified method	Open	Yes	101	97
Bergmann 1995 B	40 mg vs 300 mg	w904	Gastro-oesophageal reflux	Parallel	Unspecified method	Unknown	No	294	136
Prewett 1991	40 mg vs 300 mg	w905	Healthy volunteers	Parallel	Unspecified method	Double	No	12	12
Blum 1986	40 mg vs 300 mg	w906	Gastro-oesophageal reflux	Parallel	Unspecified method	Double	No	82	82
Havelund 1988	40 mg vs 300 mg	w907	Gastro-oesophageal reflux	Parallel	Closed list	Double	No	80	82
Jaspersen 1996	40 mg vs 300 mg	w908	Gastro-oesophageal reflux	Parallel	Unspecified method	Unknown	No	20	18
Karnes 1990	40 mg vs 300 mg	w869	Healthy volunteers	Parallel	Closed list	Double	No	12	12
Pellegrini 2005 G	40 mg vs 600 mg	w909	Gastro-oesophageal reflux	Parallel	Unspecified method	Open	No	48	47
Vantrappen 1988	40 mg vs 300 mg	w910	Gastro-oesophageal reflux	Parallel	Unspecified method	Double	Yes	31	30
Treiber 1997	40 mg vs 600 mg	w847	Gastritis or duodenitis	Parallel	Unspecified method	Single	No	20	20
Hurlimann 1994	40 mg vs 1200 mg	w911	Healthy volunteers	Parallel	Unspecified method	Double	No	-	-
Dammann 1986 B	40 mg vs 300 mg	w912	Gastro-oesophageal reflux	Parallel	Sealed envelopes	Double	No	89	89
Lundell 1991	40 mg vs 600 mg	w913	Gastro-oesophageal reflux	Parallel	Closed list	Double	Yes	51	47
Houben 1995	40 mg vs 600 mg	w141	Healthy volunteers	Crossover	Unspecified method	Double	No	3	3
Klinkenberg-Knol 1988	60 mg vs 300 mg	w914	Gastro-oesophageal reflux	Crossover	Unspecified method	Double	Yes	3	4
Klinkenberg-Knol 1987	60 mg vs 300 mg	w915	Gastro-oesophageal reflux	Parallel	Unspecified method	Double	Yes	25	26
Ofman 2000	Unknown vs Unknown	w916	Barrett's oesophagus	Parallel	Unspecified method	Double	No	-	-
Peters 1999 A	80 mg vs 300 mg	w917	Barrett's oesophagus	Parallel	Closed list	Double	No	33	35
Peters 1999 B	80 mg vs 300 mg	w918	Barrett's oesophagus	Parallel	Closed list	Double	No	33	35
Nadeau 2012	Unknown vs Unknown	w826	Percutaneous coronary intervention	Parallel	Unspecified method	Unknown	No	-	-
Ahmad 2012	Unknown vs Unknown	w919	Gastritis or duodenitis	Parallel	Unspecified method	Unknown	No	26	20
Omeprazole vs Roxatidine									
Guslandi 1994	20 mg vs 150 mg	w863	Gastro-oesophageal reflux	Parallel	Unspecified method	Unknown	Yes	6	6
Nakamura 2010	20 mg vs 150 mg	w920	Gastro-oesophageal reflux	Parallel	Unspecified method	Unknown	Yes	17	16
Goh 1994	40 mg vs 300 mg	w921	Gastritis or duodenitis	Parallel	Unspecified method	Unknown	No	11	14
Pantoprazole vs Famotidine									
Ng 2010	20 mg vs 80 mg	w922	Recently healed ulcers	Parallel	Pharmacy	Double	Yes	65	65
Arbel 2013	40 mg vs 80 mg	w865	Antiplatelet therapy	Crossover	Sealed envelopes	Single	Yes	21	21
Pantoprazole vs Lafutidine									
Maity 2014	40 mg vs 10 mg	w923	Gastro-oesophageal reflux	Parallel	Unspecified method	Open	Yes	61	61
Pantoprazole vs Nizatidine									
Kovacs 2002	20 mg vs 300 mg	w924	Gastro-oesophageal reflux	Parallel	Unspecified method	Double	Yes	80	82
Armstrong 2001	40 mg vs 300 mg	w925	Gastro-oesophageal reflux	Parallel	Closed list	Double	Yes	111	109

Webtable 5 (cont.): Details of trials involving a randomised comparison of a proton pump inhibitor (X) versus histamine-2 receptor antagonist (Y)

Trial name	Regimen	Ref.	Indication for treatment	Design	Allocation Concealment	Blinding of investigators and patients	Availability of data	No. of patients NX	No. of patients NY
Kovacs 2002	40 mg vs 300 mg	w924	Gastro-oesophageal reflux	Parallel	Unspecified method	Double	Yes	81	82
Pare 2003	40 mg vs 300 mg	w926	Gastro-oesophageal reflux	Parallel	Unspecified method	Double	No	111	109
Pantoprazole vs Ranitidine									
Metz 2003	10 mg vs 300 mg	w927	Gastro-oesophageal reflux	Parallel	Unspecified method	Double	Yes	89	95
Richter 2004	10 mg vs 300 mg	w928	Gastro-oesophageal reflux	Parallel	Other method	Double	No	88	88
Talley 2002	20 mg vs 300 mg	w929	Gastro-oesophageal reflux	Parallel	Closed list	Double	No	154	153
Van Zyl 2004	20 mg vs 300 mg	w930	Gastro-oesophageal reflux	Parallel	Closed list	Double	No	167	171
Ramirez-Barba 1999	20 mg vs 300 mg	w931	Gastro-oesophageal reflux	Parallel	Unspecified method	Double	No	184	89
Metz 2003	20 mg vs 300 mg	w927	Gastro-oesophageal reflux	Parallel	Unspecified method	Double	Yes	93	95
Adamek 2001	20 mg vs 150 mg	w932	Gastro-oesophageal reflux	Parallel	Unspecified method	Double	No	199	104
Dettmer 1998	20 mg vs 300 mg	w933	Gastro-oesophageal reflux	Parallel	Unspecified method	Double	No	105	104
Kaspari 2001	20 mg vs 300 mg	w934	Gastro-oesophageal reflux	Parallel	Unspecified method	Double	No	181	175
Van Zyl 2000	20 mg vs 300 mg	w935	Gastro-oesophageal reflux	Parallel	Closed list	Double	No	101	100
Richter 2004	20 mg vs 300 mg	w928	Gastro-oesophageal reflux	Parallel	Other method	Double	No	88	88
Dulbecco 2003	40 mg vs 30 mg	w936	Gastro-oesophageal reflux	Parallel	Unspecified method	Open	No	30	30
Duvnjak 2002	40 mg vs 300 mg	w937	Gastro-oesophageal reflux	Parallel	Unspecified method	Open	No	100	84
Metz 2003	40 mg vs 300 mg	w927	Gastro-oesophageal reflux	Parallel	Unspecified method	Double	Yes	94	95
Awad 2001	40 mg vs 300 mg	w938	Gastro-oesophageal reflux	Parallel	Onsite computer	Double	Yes	12	10
Teyssen 2001	40 mg vs 300 mg	w939	Healthy volunteers	Crossover	Closed list	Open	Yes	10	10
Koop 1995	40 mg vs 300 mg	w940	Gastro-oesophageal reflux	Parallel	Unspecified method	Double	No	166	83
Meneghelli 2002	40 mg vs 300 mg	w941	Gastro-oesophageal reflux	Parallel	Onsite computer	Double	No	128	128
Armbrecht 1997	40 mg vs 300 mg	w942	Gastro-oesophageal reflux	Parallel	Unspecified method	Double	Yes	11	13
Richter 2004	40 mg vs 300 mg	w928	Gastro-oesophageal reflux	Parallel	Other method	Double	No	85	88
Gallo 1998	40 mg vs 300 mg	w943	Gastro-oesophageal reflux	Parallel	Unspecified method	Double	No	211	104
Müller 1998	40 mg vs 300 mg	w190	Healthy volunteers	Parallel	Unspecified method	Double	Yes	16	16
Parri 2011	40 mg vs 150 mg	w944	Antiplatelet therapy	Parallel	Unspecified method	Open	No	54	51
Demir 2009 (diabetes mellitus)	80 mg vs 800 mg	w945	Diabetes	Parallel	Unspecified method	Unknown	Yes	49	49
Demir 2009 (no diabetes mellitus)	80 mg vs 800 mg	w945	Gastro-oesophageal reflux	Parallel	Unspecified method	Unknown	Yes	58	58
Nadeau 2012	Unknown vs Unknown	w826	Percutaneous coronary intervention	Parallel	Unspecified method	Unknown	No	-	-
Rabeprazole vs Famotidine									
Chan 2014 B	20 mg vs 40 mg	w946	Antiplatelet therapy	Parallel	Unspecified method	Double	Yes	84	79
ACCEL-PROTECT	20 mg vs 40 mg	w947	Percutaneous coronary intervention	Parallel	Unspecified method	Unknown	No	40	40
Rabeprazole vs Lafutidine									
Dewan 2011	20 mg vs 10 mg	w948	Gastro-oesophageal reflux	Parallel	Unspecified method	Open	Yes	101	101
Rabeprazole vs Ranitidine									
Jee 2005	10 mg vs 300 mg	w949	Gastro-oesophageal reflux	Parallel	Unspecified method	Unknown	No	42	42
Jeong 2006	20 mg vs 600 mg	w950	Gastro-oesophageal reflux	Parallel	Unspecified method	Open	Yes	53	57
Farley 2000	20 mg vs 600 mg	w951	Gastro-oesophageal reflux	Parallel	Unspecified method	Double	No	169	169
Dai 2015	20 mg vs 300 mg	w952	Gastro-oesophageal reflux	Parallel	Unspecified method	Unknown	Yes	65	65
**Healing Trials**									
Esomeprazole vs Ranitidine									
Astra Zeneca 285	20 mg vs 300 mg	w953	Peptic ulcers	Parallel	Sealed envelopes	Double	Yes	140	134
Astra Zeneca 286	20 mg vs 300 mg	w954	Peptic ulcers	Parallel	Sealed envelopes	Double	Yes	150	149
Astra Zeneca 285	40 mg vs 300 mg	w953	Peptic ulcers	Parallel	Sealed envelopes	Double	Yes	132	134
Astra Zeneca 286	40 mg vs 300 mg	w954	Peptic ulcers	Parallel	Sealed envelopes	Double	Yes	141	149
Lansoprazole vs Cimetidine									
Aoyama 1995	30 mg vs 800 mg	w955	Peptic ulcers	Parallel	Unspecified method	Unknown	Yes	70	23
Lansoprazole vs Famotidine									
Tsuji 1995	30 mg vs 40 mg	w956	Peptic ulcers	Parallel	Unspecified method	Unknown	Yes	8	8

Webtable 5 (cont.): Details of trials involving a randomised comparison of a proton pump inhibitor (X) versus histamine-2 receptor antagonist (Y)

Trial name	Regimen	Ref.	Indication for treatment	Design	Allocation Concealment	Blinding of investigators and patients	Availability of data	No. of patients NX	NY
Sakaguchi 1995	30 mg vs 40 mg	w957	Peptic ulcers	Parallel	Sealed envelopes	Unknown	Yes	10	10
Okai 1995	30 mg vs 40 mg	w958	Peptic ulcers	Parallel	Unspecified method	Unknown	Yes	12	12
Hotz 1992	30 mg vs 40 mg	w959	Peptic ulcers	Parallel	Other method	Double	Yes	174	90
Nema 2010	30 mg vs 40 mg	w960	Peptic ulcers	Parallel	Onsite computer	Unknown	Yes	30	30
Nakamura 1995	120 mg vs 40 mg	w819	Peptic ulcers	Parallel	Unspecified method	Unknown	Yes	5	5
Lansoprazole vs other H2RA									
Nema 2008	30 mg vs Unknown	w961	Peptic ulcers	Parallel	Onsite computer	Unknown	Yes	16	14
Lansoprazole vs Ranitidine									
Londong 1991	7.5 mg vs 300 mg	w962	Peptic ulcers	Parallel	Other method	Double	Yes	77	79
Licht 1990	7.5 mg vs 300 mg	w963	Peptic ulcers	Parallel	Unspecified method	Double	Yes	45	48
Londong 1991	15 mg vs 300 mg	w962	Peptic ulcers	Parallel	Other method	Double	Yes	80	79
Licht 1990	15 mg vs 300 mg	w963	Peptic ulcers	Parallel	Unspecified method	Double	Yes	45	48
Agrawal 2000	15 mg vs 300 mg	w964	Peptic ulcers	Parallel	Unspecified method	Double	Yes	119	117
Jhala 1995	15 mg vs 300 mg	w233	Peptic ulcers	Parallel	Unspecified method	Double	Yes	34	33
Spadaccini 1998 (duodenal ulcer)	15 mg vs 800 mg	w836	Peptic ulcers	Parallel	Unspecified method	Open	Yes	13	12
Lanza 1994	15 mg vs 300 mg	w234	Peptic ulcers	Parallel	Unspecified method	Double	Yes	87	86
Goldstein 2000	15 mg vs 300 mg	w965	Peptic ulcers	Parallel	Unspecified method	Double	Yes	114	116
Spadaccini 1998 (gastric ulcer)	15 mg vs 800 mg	w836	Peptic ulcers	Parallel	Unspecified method	Open	Yes	5	4
Michel 1994	30 mg vs 300 mg	w966	Peptic ulcers	Parallel	Unspecified method	Double	Yes	79	79
Londong 1991	30 mg vs 300 mg	w962	Peptic ulcers	Parallel	Other method	Double	Yes	78	79
Licht 1990	30 mg vs 300 mg	w963	Peptic ulcers	Parallel	Unspecified method	Double	Yes	45	48
Agrawal 2000	30 mg vs 300 mg	w964	Peptic ulcers	Parallel	Unspecified method	Double	Yes	117	117
Bardhan 1999 A X1	30 mg vs 300 mg	w832	Peptic ulcers	Extension	Unspecified method	Double	Yes	219	217
Capurso 1995 A	30 mg vs 300 mg	w967	Peptic ulcers	Parallel	Other method	Double	Yes	29	34
Jhala 1995	30 mg vs 300 mg	w233	Peptic ulcers	Parallel	Unspecified method	Double	Yes	33	33
Hawkey 1993	30 mg vs 300 mg	w968	Peptic ulcers	Parallel	Unspecified method	Double	Yes	95	98
Lazzaroni 1997	30 mg vs 300 mg	w969	Peptic ulcers	Parallel	Unspecified method	Single	Yes	40	40
Bardhan 1994	30 mg vs 300 mg	w970	Peptic ulcers	Parallel	Closed list	Double	Yes	82	83
Lanza 1994	30 mg vs 300 mg	w234	Peptic ulcers	Parallel	Unspecified method	Double	Yes	84	86
Russo 1997 X1	30 mg vs 300 mg	w66	Peptic ulcers	Extension	Other method	Double	Yes	68	64
Goldstein 2000	30 mg vs 300 mg	w965	Peptic ulcers	Parallel	Unspecified method	Double	Yes	112	116
Vreer 1999	30 mg vs 300 mg	w971	Peptic ulcers	Parallel	Unspecified method	Double	Yes	42	45
Hawkey 1993	60 mg vs 300 mg	w968	Peptic ulcers	Parallel	Unspecified method	Double	Yes	96	98
Bardhan 1994	60 mg vs 300 mg	w970	Peptic ulcers	Parallel	Closed list	Double	Yes	85	83
Lansoprazole vs Roxatidine									
Fujino 1995	30 mg vs 150 mg	w972	Peptic ulcers	Parallel	Sealed envelopes	Unknown	Yes	66	66
Imaeda 2011	30 mg vs 150 mg	w973	Endoscopic submucosal dissection	Parallel	Other method	Unknown	Yes	65	64
Omeprazole vs Cimetidine									
Bigard 1987	20 mg vs 800 mg	w974	Peptic ulcers	Parallel	Unspecified method	Double	Yes	144	154
Archambault 1988	20 mg vs 1200 mg	w975	Peptic ulcers	Parallel	Unspecified method	Double	Yes	85	84
Wilairatana 1989	20 mg vs 800 mg	w976	Peptic ulcers	Parallel	Unspecified method	Double	Yes	129	128
Crowe 1989	20 mg vs 800 mg	w977	Peptic ulcers	Parallel	Unspecified method	Double	Yes	98	91
Pan 1994	20 mg vs 800 mg	w978	Peptic ulcers	Parallel	Unspecified method	Single	Yes	44	64
Kim 1994	20 mg vs 900 mg	w979	Peptic ulcers	Parallel	Unspecified method	Unknown	Yes	13	12
Bate 1989	20 mg vs 800 mg	w980	Peptic ulcers	Parallel	Unspecified method	Double	Yes	105	92
Lauritsen 1985	30 mg vs 1000 mg	w981	Peptic ulcers	Parallel	Unspecified method	Double	Yes	67	65
Danish Omeprazole Study	30 mg vs 1000 mg	w982	Peptic ulcers	Parallel	Closed list	Double	Yes	79	82
Dahlgren 1988	30 mg vs 800 mg	w983	Peptic ulcers	Parallel	Unspecified method	Double	Yes	79	75
Lauritsen 1989 B	30 mg vs 1000 mg	w984	Peptic ulcers	Parallel	Unspecified method	Double	Yes	90	86

Webtable 5 (cont.): Details of trials involving a randomised comparison of a proton pump inhibitor (X) versus histamine-2 receptor antagonist (Y)

Trial name	Regimen	Ref.	Indication for treatment	Design	Allocation Concealment	Blinding of investigators and patients	Availability of data	No. of patients NX	NY
Omeprazole vs Famotidine									
Popovic 1990	20 mg vs 40 mg	w985	Peptic ulcers	Parallel	Unspecified method	Double	Yes	70	69
Lin 1994	20 mg vs 40 mg	w986	Peptic ulcers	Parallel	Closed list	Unknown	Yes	30	30
Esaki 2002	20 mg vs 40 mg	w987	Endoscopic mucosal resection	Parallel	Unspecified method	Single	Yes	8	8
Delle Fave 1992	20 mg vs 40 mg	w988	Peptic ulcers	Parallel	Unspecified method	Double	Yes	116	125
Ramesh Kumar 1992	20 mg vs 40 mg	w989	Peptic ulcers	Parallel	Other method	Double	Yes	73	67
Misra 1993	20 mg vs 40 mg	w990	Peptic ulcers	Parallel	Unspecified method	Double	Yes	30	30
Yamaguchi 2005	20 mg vs 40 mg	w991	Endoscopic mucosal resection	Parallel	Unspecified method	Unknown	No	29	28
Ye 2006	20 mg vs 40 mg	w992	Endoscopic mucosal resection	Parallel	Unspecified method	Double	Yes	50	50
Tomita 2012	20 mg vs 40 mg	w993	Endoscopic submucosal dissection	Parallel	Unspecified method	Single	Yes	79	79
Di Mario 1993 A	40 mg vs 40 mg	w994	Peptic ulcers	Parallel	Unspecified method	Unknown	Yes	12	7
Goh 1996	40 mg vs 80 mg	w995	Peptic ulcers	Parallel	Unspecified method	Single	Yes	33	33
Hsu 2001	40 mg vs 80 mg	w996	Peptic ulcers	Parallel	Unspecified method	Unknown	Yes	60	60
Omeprazole vs H2RA									
Wang 1996 (no amoxycillin)	20 mg vs Unknown	w997	Peptic ulcers	Extension	Unspecified method	Unknown	Yes	22	21
Omeprazole vs Ranitidine									
Hui 1989 A	10 mg vs 300 mg	w998	Peptic ulcers	Parallel	Unspecified method	Double	Yes	90	90
Hui 1989 B	10 mg vs 300 mg	w999	Peptic ulcers	Parallel	Unspecified method	Double	Yes	3	3
ASTRONAUT X1	20 mg vs 300 mg	w874	Peptic ulcers	Extension	Unspecified method	Double	Yes	113	117
Marks 1991	20 mg vs 300 mg	w1000	Peptic ulcers	Parallel	Unspecified method	Double	Yes	104	106
Valenzuela 1991	20 mg vs 300 mg	w1001	Peptic ulcers	Parallel	Unspecified method	Double	Yes	151	158
Walan 1989	20 mg vs 300 mg	w1002	Peptic ulcers	Parallel	Unspecified method	Double	Yes	203	205
Bergmann 1995 A	20 mg vs 300 mg	w1003	Peptic ulcers	Parallel	Unspecified method	Unknown	Yes	373	172
Miszputen 1997	20 mg vs 300 mg	w1004	Peptic ulcers	Parallel	Unspecified method	Double	Yes	92	88
Lee 1991 A (gastric ulcer)	20 mg vs 300 mg	w1005	Peptic ulcers	Parallel	Unspecified method	Double	Yes	10	6
Classen 1985 A	20 mg vs 300 mg	w1006	Peptic ulcers	Parallel	Unspecified method	Double	Yes	168	166
Hotz 1995 B (gastric ulcer)	20 mg vs 300 mg	w1007	Peptic ulcers	Parallel	Onsite computer	Unknown	Yes	15	13
Hallerbäck 1998 A	20 mg vs 300 mg	w1008	Peptic ulcers	Parallel	Unspecified method	Double	Yes	195	198
Arber 1994	20 mg vs 300 mg	w1009	Peptic ulcers	Parallel	Unspecified method	Double	Yes	103	100
Al Karawi 1996	20 mg vs 300 mg	w1010	Peptic ulcers	Parallel	Unspecified method	Double	Yes	67	67
Barbara 1987	20 mg vs 300 mg	w1011	Peptic ulcers	Parallel	Unspecified method	Double	Yes	64	64
Ahmed 1995	20 mg vs 300 mg	w1012	Peptic ulcers	Parallel	Unspecified method	Open	Yes	21	20
Bardhan 1986	20 mg vs 300 mg	w1013	Peptic ulcers	Parallel	Unspecified method	Double	Yes	34	35
Lysy 1992	20 mg vs 300 mg	w1014	Peptic ulcers	Parallel	Unspecified method	Double	Yes	30	30
Chelvam 1989	20 mg vs 300 mg	w1015	Peptic ulcers	Parallel	Unspecified method	Double	Yes	109	111
Archimandritis 1995	20 mg vs 300 mg	w1016	Peptic ulcers	Parallel	Unspecified method	Unknown	No	126	119
Italian Cooperative Group on Omeprazole	20 mg vs 300 mg	w1017	Peptic ulcers	Parallel	Unspecified method	Double	Yes	84	83
Zaterka 1993	20 mg vs 300 mg	w1018	Peptic ulcers	Parallel	Unspecified method	Double	Yes	120	121
Crowe 1994	20 mg vs 300 mg	w1019	Peptic ulcers	Parallel	Unspecified method	Double	Yes	117	121
Delchier 1989	20 mg vs 300 mg	w1020	Peptic ulcers	Parallel	Unspecified method	Double	Yes	75	76
Choi 1994	20 mg vs 300 mg	w1021	Peptic ulcers	Parallel	Other method	Double	Yes	126	124
Catalano 1991	20 mg vs 300 mg	w1022	Peptic ulcers	Parallel	Unspecified method	Double	Yes	22	21
Pare 1990	20 mg vs 300 mg	w1023	Peptic ulcers	Parallel	Unspecified method	Double	Yes	39	41
Hui 1989 A	20 mg vs 300 mg	w998	Peptic ulcers	Parallel	Unspecified method	Double	Yes	90	90
Mulder 1989	20 mg vs 300 mg	w1024	Peptic ulcers	Parallel	Onsite computer	Double	Yes	91	90
Hui 1989 B	20 mg vs 300 mg	w999	Peptic ulcers	Parallel	Unspecified method	Double	Yes	2	3
Rossini 1989	20 mg vs 300 mg	w1025	Peptic ulcers	Parallel	Unspecified method	Double	Yes	9	9
Wang 1992	20 mg vs 300 mg	w1026	Peptic ulcers	Parallel	Unspecified method	Double	Yes	118	108
McFarland 1990	20 mg vs 300 mg	w1027	Peptic ulcers	Parallel	Unspecified method	Double	Yes	126	122
Classen 1985 B	20 mg vs 300 mg	w1028	Peptic ulcers	Parallel	Unspecified method	Double	Yes	84	74
Glise 1991	20 mg vs 300 mg	w1029	Peptic ulcers	Parallel	Unspecified method	Double	No	234	240

Webtable 5 (cont.): Details of trials involving a randomised comparison of a proton pump inhibitor (X) versus histamine-2 receptor antagonist (Y)

Trial name	Regimen	Ref.	Indication for treatment	Design	Allocation Concealment	Blinding of investigators and patients	Availability of data	No. of patients NX	NY
Sonnenberg 1999	20 mg vs 300 mg	w1030	Peptic ulcers	Parallel	Unspecified method	Double	No	279	263
Hotz 1995 B (duodenal ulcer)	20 mg vs 300 mg	w1007	Peptic ulcers	Parallel	Closed list	Unknown	Yes	13	12
Diaz Rubio 1991	20 mg vs 300 mg	w1031	Peptic ulcers	Parallel	Unspecified method	Double	Yes	79	79
Parodi 1992	20 mg vs 300 mg	w1032	Peptic ulcers	Parallel	Unspecified method	Unknown	Yes	10	10
Lee 1991 A (duodenal ulcer)	20 mg vs 300 mg	w1005	Peptic ulcers	Parallel	Unspecified method	Double	Yes	21	16
Cataldo 1996	27 mg vs 400 mg	w1033	Peptic ulcers	Parallel	Unspecified method	Unknown	Yes	20	20
Kager 1991	30 mg vs 300 mg	w1034	Peptic ulcers	Parallel	Unspecified method	Double	Yes	74	73
ASTRONAUT X1	40 mg vs 300 mg	w874	Peptic ulcers	Extension	Unspecified method	Double	Yes	114	117
Walan 1989	40 mg vs 300 mg	w1002	Peptic ulcers	Parallel	Unspecified method	Double	Yes	194	205
Sonnenberg 1998	40 mg vs 300 mg	w1035	Peptic ulcers	Parallel	Onsite computer	Double	No	279	263
Bardhan 1986	40 mg vs 300 mg	w1013	Peptic ulcers	Parallel	Unspecified method	Double	Yes	36	35
Di Mario 1993 A	40 mg vs 300 mg	w994	Peptic ulcers	Parallel	Unspecified method	Unknown	Yes	12	23
Pare 1990	40 mg vs 300 mg	w1023	Peptic ulcers	Parallel	Unspecified method	Double	Yes	38	41
Tulassay 1992	40 mg vs 300 mg	w1036	Peptic ulcers	Parallel	Unspecified method	Unknown	Yes	22	23
Michopoulos 2000	40 mg vs 600 mg	w1037	Peptic ulcers	Parallel	Unspecified method	Open	No	78	78
Pellegrini 2005 U	40 mg vs 600 mg	w909	Peptic ulcers	Parallel	Other method	Open	Yes	27	28
Cooperative Study 1990 (gastric ulcer)	40 mg vs 300 mg	w1038	Peptic ulcers	Parallel	Unspecified method	Double	Yes	21	25
Cooperative Study 1990 (duodenal ulcer)	40 mg vs 300 mg	w1038	Peptic ulcers	Parallel	Unspecified method	Double	Yes	96	98
Omeprazole vs Nizatidine									
Russo 1994	20 mg vs 600 mg	w1039	Peptic ulcers	Parallel	Unspecified method	Open	Yes	15	16
Brummer 1996	20 mg vs 300 mg	w1040	Peptic ulcers	Parallel	Unspecified method	Open	Yes	11	12
Di Mario 1993 A	40 mg vs 300 mg	w994	Peptic ulcers	Parallel	Unspecified method	Unknown	Yes	12	12
Other PPI vs Famotidine									
Nakashima 2009	Unknown vs 40 mg	w1041	Peptic ulcers	Parallel	Unspecified method	Unknown	Yes	20	20
Other PPI vs other H2RA									
Sasakii 1999	Unknown vs Unknown	w1042	Peptic ulcers	Parallel	Unspecified method	Open	Yes	116	196
Noh 2013	Unknown vs Unknown	w1043	Endoscopic submucosal dissection	Parallel	Unspecified method	Unknown	No	92	98
Kang 2011	Unknown vs Unknown	w1044	Endoscopic submucosal dissection	Parallel	Unspecified method	Unknown	No	-	-
Pantoprazole vs Famotidine									
Jeon 2010	40 mg vs 40 mg	w1045	Endoscopic submucosal dissection	Parallel	Unspecified method	Unknown	No	11	13
Oh 2010	40 mg vs 40 mg	w1046	Endoscopic submucosal dissection	Parallel	Unspecified method	Unknown	No	31	31
Seo 2011	40 mg vs 40 mg	w1047	Endoscopic submucosal dissection	Parallel	Unspecified method	Unknown	No	74	74
Pantoprazole vs Ranitidine									
Papp 1997	40 mg vs 300 mg	w1048	Peptic ulcers	Parallel	Unspecified method	Double	Yes	160	166
Schepp 1995	40 mg vs 300 mg	w1049	Peptic ulcers	Parallel	Unspecified method	Double	Yes	177	89
Cremer 1995	40 mg vs 300 mg	w1050	Peptic ulcers	Parallel	Unspecified method	Double	Yes	137	139
Chen 1999	40 mg vs 300 mg	w1051	Peptic ulcers	Parallel	Unspecified method	Double	Yes	80	80
Meneghelli 2000	40 mg vs 300 mg	w1052	Peptic ulcers	Parallel	Unspecified method	Double	Yes	111	111
Judmaier 1994	40 mg vs 300 mg	w1053	Peptic ulcers	Parallel	Unspecified method	Double	Yes	98	104
Fric 1996	40 mg vs 300 mg	w1054	Peptic ulcers	Parallel	Unspecified method	Double	Yes	47	48
Hotz 1995 A	40 mg vs 300 mg	w1055	Peptic ulcers	Parallel	Unspecified method	Double	Yes	166	82
van Rensburg 1994	40 mg vs 300 mg	w1056	Peptic ulcers	Parallel	Closed list	Double	Yes	102	97
Dibildox 1996	40 mg vs 300 mg	w1057	Peptic ulcers	Parallel	Unspecified method	Double	Yes	89	89
Rabeprazole vs other H2RA									
Saigenji 2002	10 mg vs Unknown	w1058	Peptic ulcers	Parallel	Unspecified method	Open	Yes	77	76
Rabeprazole vs Ranitidine									
Breiter 2000	20 mg vs 300 mg	w1059	Peptic ulcers	Parallel	Unspecified method	Double	Yes	188	188

Webtable 5 (cont.): Details of trials involving a randomised comparison of a proton pump inhibitor (X) versus histamine-2 receptor antagonist (Y)

Trial name	Regimen	Ref.	Indication for treatment	Design	Allocation Concealment	Blinding of investigators and patients	Availability of data	No. of patients NX	NY
Rabeprazole vs Roxatidine									
Takeuchi 2011	10 mg vs 150 mg	w1060	Endoscopic submucosal dissection	Parallel	Sealed envelopes	Unknown	No	30	30
**Acute Bleeding Trials**									
Esomeprazole vs Cimetidine									
Bai 2015	192 mg vs 1440 mg	w1061	Bleeding Peptic Ulcer	Parallel	Closed list	Double	Yes	111	111
Lansoprazole vs Roxatidine									
Ashida 2007	60 mg vs 150 mg	w1062	UGI bleeding of any cause	Parallel	Unspecified method	Double	Yes	135	135
Omeprazole vs Cimetidine									
Chua 1996	40 mg vs 1200 mg	w1063	Bleeding Peptic Ulcer	Parallel	Sealed envelopes	Unknown	Yes	10	10
Chua 1996	80 mg vs 1200 mg	w1063	Bleeding Peptic Ulcer	Parallel	Sealed envelopes	Unknown	Yes	10	10
Lin 2006	80 mg vs 800 mg	w1064	Bleeding Peptic Ulcer	Parallel	Sealed envelopes	Open	Yes	66	67
Lin 2006	160 mg vs 800 mg	w1064	Bleeding Peptic Ulcer	Parallel	Sealed envelopes	Open	Yes	67	67
Lin 1998	160 mg vs 1200 mg	w1065	Bleeding Peptic Ulcer	Parallel	Sealed envelopes	Unknown	Yes	50	50
Omeprazole vs Ranitidine									
Fasseas 2001	40 mg vs 200 mg	w1066	Bleeding Peptic Ulcer	Parallel	Unspecified method	Single	Yes	45	47
Artal 1996	79.2 mg vs 300 mg	w1067	Bleeding Peptic Ulcer	Parallel	Unspecified method	Unknown	No	-	-
Artal 1996	79.2 mg vs 300 mg	w1067	Bleeding Peptic Ulcer	Parallel	Unspecified method	Unknown	No	-	-
Lanas 1995	80 mg vs 300 mg	w1068	Bleeding Peptic Ulcer	Parallel	Unspecified method	Open	Yes	28	23
Sheu 2002	80 mg vs 150 mg	w1069	Bleeding Peptic Ulcer	Parallel	Unspecified method	Unknown	Yes	86	89
Cardi 1997	80 mg vs 400 mg	w1070	Bleeding Peptic Ulcer	Parallel	Sealed envelopes	Double	Yes	21	24
Coraggio 1998	80 mg vs 200 mg	w1071	Bleeding Peptic Ulcer	Parallel	Unspecified method	Unknown	Yes	24	24
Artal 1996	80 mg vs 300 mg	w1067	Bleeding Peptic Ulcer	Parallel	Unspecified method	Unknown	No	-	-
Artal 1996	80 mg vs 300 mg	w1067	Bleeding Peptic Ulcer	Parallel	Unspecified method	Unknown	No	-	-
Orti 1995	96 mg vs 200 mg	w1072	Bleeding Peptic Ulcer	Parallel	Unspecified method	Open	Yes	267	252
Villanueva 1995	120 mg vs 200 mg	w1073	Bleeding Peptic Ulcer	Parallel	Sealed envelopes	Unknown	Yes	48	48
Artal 1996	120 mg vs 300 mg	w1067	Bleeding Peptic Ulcer	Parallel	Unspecified method	Unknown	No	-	-
Artal 1996	120 mg vs 300 mg	w1067	Bleeding Peptic Ulcer	Parallel	Unspecified method	Unknown	No	-	-
Labenz 1997 A	272 mg vs 530 mg	w1074	Bleeding Peptic Ulcer	Parallel	Sealed envelopes	Unknown	Yes	20	20
Pantoprazole vs Ranitidine									
Hsu 2004	80 mg vs 150 mg	w1075	Bleeding Peptic Ulcer	Parallel	Sealed envelopes	Unknown	Yes	52	50
van Rensburg 2009	192 mg vs 312 mg	w1076	Bleeding Peptic Ulcer	Parallel	Other method	Double	Yes	625	631
Jensen 2006	192 mg vs 150 mg	w1077	Bleeding Peptic Ulcer	Parallel	Onsite computer	Double	Yes	72	77
Gashi 2012	Unknown vs Unknown	w1078	Bleeding Peptic Ulcer	Parallel	Unspecified method	Double	No	60	62

Design: Parallel=parallel trial. Factorial=Factorial trial. 'Extension' studies are studies in which patients are randomised more than once during the trial (only the parts of the trial that involve a valid randomised comparison for this meta-analysis, labelled X1, X2 etc., are shown in this table). Data shown for crossover trials reflect only events during the first phase of the trial. The suffixes A, B, C, D are used to distinguish different trials published by the same author in the same year.

Allocation concealment: IVRS (Interactive Voice Response System); Telephone (telephone central office); Pharmacy (pharmacy coded containers administered sequentially); Onsite computer (onsite computer accessible only after entering patient details); Sealed envelopes (sequentially numbered, opaque or without further specification); Closed list (closed list of random numbers); Other (other specified method); Unspecified (stated as being 'randomised' but with no other details).

Blinding of investigators and patients: Single=single-blind; Double=double-blind; Open=open label.

Availability of data: Data available for at least one endpoint of interest in the meta-analyses.

In cases where the number randomised was not reported, the number evaluated was used instead. In addition, for crossover trials, where neither the number randomised nor the number evaluated was reported, the total number of participants divided by number of arms was used.

Webtable 6: Details of trials involving a randomised comparison of a histamine-2 receptor antagonist (X) versus a prostaglandin analogue (Y)

Trial name	Regimen	Ref.	Indication for treatment	Design	Allocation Concealment	Blinding of investigators and patients	Availability of data	No. of patients NX	NY
**Prevention Trials**									
Famotidine vs Misoprostol									
Yildiz 1996	40 mg vs 400 ug	w1079	Antiplatelet therapy	Parallel	Unspecified method	Unknown	No	16	16
Famotidine vs Rebamipide									
FORCE	20 mg vs 300 mg	w1080	Antiplatelet therapy	Parallel	Unspecified method	Single	Yes	66	63
Ranitidine vs Enprostil									
Lauritsen 1987	150 mg vs 35 ug	w1081	Recently healed ulcers	Parallel	Closed list	Double	Yes	71	71
Bardhan 1989	150 mg vs 35 ug	w1082	Recently healed ulcers	Parallel	Unspecified method	Single	Yes	64	64
Heijerman 1990	300 mg vs 70 ug	w1083	Cystic fibrosis	Crossover	Unspecified method	Double	No	4	4
Ranitidine vs Misoprostol									
Goldin 1991	150 mg vs 400 ug	w278	Recently healed ulcers	Parallel	Closed list	Double	Yes	24	25
Raskin 1996	300 mg vs 800 ug	w1084	Arthritis, rheumatic or musculoskeletal disorders	Parallel	Unspecified method	Double	Yes	269	269
Valentini 1995	300 mg vs 400 ug	w1085	Cancer pain	Parallel	Unspecified method	Unknown	Yes	31	30
Bianchi Porro 1997	300 mg vs 600 ug	w1086	Arthritis, rheumatic or musculoskeletal disorders	Parallel	Closed list	Double	Yes	24	23
Bianchi Porro 1997	300 mg vs 400 ug	w1086	Arthritis, rheumatic or musculoskeletal disorders	Parallel	Closed list	Double	Yes	24	23
Ranitidine vs Rioprostil									
Rampal 1989	150 mg vs 300 ug	w1087	Recently healed ulcers	Parallel	Unspecified method	Double	No	95	95
Businger 1989 B	150 mg vs 600 ug	w1088	Recently healed ulcers	Parallel	Unspecified method	Double	Yes	110	97
Roxatidine vs Misoprostol									
Müller 1994 B	150 mg vs 400 ug	w653	Healthy volunteers	Parallel	Unspecified method	Single	Yes	12	12
**Healing Trials**									
Cimetidine vs Enprostil									
Frexinos 1989	800 mg vs 70 ug	w1089	Peptic ulcers	Parallel	Closed list	Double	Yes	39	41
Winters 1986	800 mg vs 70 ug	w1090	Peptic ulcers	Parallel	Other method	Double	Yes	181	188
Jazrawi 1988	800 mg vs 70 ug	w1091	Peptic ulcers	Parallel	Unspecified method	Double	Yes	59	61
Carling 1987	800 mg vs 70 mg	w1092	Peptic ulcers	Parallel	Unspecified method	Double	Yes	52	54
Unge 1989 A	1000 mg vs 70 ug	w1093	Peptic ulcers	Parallel	Unspecified method	Double	Yes	24	28
Villalobos 1987	1200 mg vs 70 ug	w1094	Peptic ulcers	Parallel	Closed list	Single	Yes	19	21
Cimetidine vs Misoprostol									
Wang 1991	800 mg vs 800 ug	w1095	Peptic ulcers	Parallel	Unspecified method	Double	Yes	106	94
Dajani 1992	800 mg vs 800 ug	w1096	Peptic ulcers	Parallel	Unspecified method	Double	Yes	106	94
Aenishänslin 1985	1200 mg vs 200 ug	w1097	Peptic ulcers	Parallel	Unspecified method	Double	Yes	39	38
Aenishänslin 1985	1200 mg vs 800 ug	w1097	Peptic ulcers	Parallel	Unspecified method	Double	Yes	39	38
Rachmilewitz 1986	1200 mg vs 200 ug	w1098	Peptic ulcers	Parallel	Closed list	Double	Yes	208	166
Rachmilewitz 1986	1200 mg vs 800 ug	w1098	Peptic ulcers	Parallel	Closed list	Double	Yes	208	208
Nicholson 1985	1200 mg vs 200 ug	w1099	Peptic ulcers	Parallel	Closed list	Double	Yes	240	228
Nicholson 1985	1200 mg vs 800 ug	w1099	Peptic ulcers	Parallel	Closed list	Double	Yes	240	235
Baccaro 1987	1200 mg vs 800 ug	w1100	Peptic ulcers	Parallel	Other method	Double	Yes	45	54
Fich 1985 B	1200 mg vs 800 ug	w1101	Peptic ulcers	Parallel	Closed list	Double	Yes	11	11
Fich 1985 B	1200 mg vs 200 ug	w1101	Peptic ulcers	Parallel	Closed list	Double	Yes	11	10
Fich 1985 A	1200 mg vs 800 ug	w1102	Peptic ulcers	Parallel	Closed list	Double	Yes	24	24
Fich 1985 A	1200 mg vs 200 ug	w1102	Peptic ulcers	Parallel	Closed list	Double	Yes	24	23
Petersen 1987	1200 mg vs 800 ug	w1103	Peptic ulcers	Parallel	Unspecified method	Double	Yes	21	22
O'Keefe 1985	1200 mg vs 200 ug	w1104	Peptic ulcers	Parallel	Unspecified method	Double	Yes	22	22
O'Keefe 1985	1200 mg vs 800 ug	w1104	Peptic ulcers	Parallel	Unspecified method	Double	Yes	22	22
Cimetidine vs Rioprostil									
Bianchi Porro 1989	800 mg vs 600 ug	w1105	Peptic ulcers	Parallel	Unspecified method	Double	Yes	127	130
Famotidine vs Rebamipide									
Nakamura 1995	40 mg vs 900 mg	w819	Peptic ulcers	Parallel	Unspecified method	Unknown	Yes	5	5

Webtable 6 (cont.): Details of trials involving a randomised comparison of a histamine-2 receptor antagonist (X) versus prostaglandin analogue (Y)

Trial name	Regimen	Ref.	Indication for treatment	Design	Allocation Concealment	Blinding of investigators and patients	Availability of data	No. of patients NX	No. of patients NY
Kim 2010	40 mg vs 300 mg	w1106	Endoscopic mucosal resection	Parallel	Unspecified method	Unknown	Yes	31	32
Ranitidine vs Enprostil									
Lartigue 1994	300 mg vs 70 ug	w1107	Peptic ulcers	Parallel	Unspecified method	Double	Yes	318	324
Bianchi Porro 1992 A	300 mg vs 70 ug	w1108	Peptic ulcers	Parallel	Unspecified method	Double	Yes	22	21
Bunce 1986	300 mg vs 70 ug	w1109	Peptic ulcers	Parallel	Unspecified method	Double	No	10	7
Bardhan 1988 B	300 mg vs 70 ug	w1110	Peptic ulcers	Parallel	Unspecified method	Double	Yes	155	158
Bardhan 1991	300 mg vs 70 ug	w1111	Peptic ulcers	Parallel	Unspecified method	Double	Yes	85	71
Dammann 1986 A	300 mg vs 70 ug	w1112	Peptic ulcers	Parallel	Other method	Double	Yes	46	47
Morgan 1990	300 mg vs 140 ug	w1113	Peptic ulcers	Parallel	Closed list	Double	Yes	49	47
Mackinnon 1987	300 mg vs 70 ug	w1114	Peptic ulcers	Parallel	Unspecified method	Double	Yes	75	67
Lauritsen 1986	300 mg vs 70 ug	w1115	Peptic ulcers	Parallel	Unspecified method	Double	Yes	90	90
Ranitidine vs Misoprostol									
Goldin 1988	300 mg vs 800 ug	w1116	Peptic ulcers	Parallel	Other method	Double	Yes	34	37
Simjee 1987	300 mg vs 800 ug	w1117	Peptic ulcers	Parallel	Unspecified method	Double	Yes	30	30
Gonvers 1987	300 mg vs 800 ug	w1118	Peptic ulcers	Parallel	Unspecified method	Double	Yes	42	45
Ranitidine vs Rioprostil									
Lavignolle 1989	300 mg vs 600 ug	w1119	Peptic ulcers	Parallel	Unspecified method	Double	Yes	80	76
Lauritsen 1989 A	300 mg vs 200 ug	w1120	Peptic ulcers	Parallel	Closed list	Double	Yes	30	30
Dammann 1989	300 mg vs 600 ug	w1121	Peptic ulcers	Parallel	Unspecified method	Double	Yes	107	105
Quinton 1989	300 mg vs 600 ug	w1122	Peptic ulcers	Parallel	Other method	Double	Yes	48	43
Dammann 1987 A	300 mg vs 600 ug	w1123	Peptic ulcers	Parallel	Unspecified method	Double	Yes	104	104
Coremans 1989	300 mg vs 600 ug	w1124	Peptic ulcers	Parallel	Unspecified method	Double	Yes	153	157
Businger 1989 A	300 mg vs 600 ug	w1125	Peptic ulcers	Parallel	Unspecified method	Double	Yes	119	118
Rutgeerts 1989	300 mg vs 600 ug	w1126	Peptic ulcers	Parallel	Closed list	Double	Yes	100	94

Data shown for crossover trials reflect only events during the first phase of the trial. The suffixes A, B are used to distinguish different trials published by the same author in the same year.

Allocation concealment: IVRS (Interactive Voice Response System); Telephone (telephone central office); Pharmacy (pharmacy coded containers administered sequentially); Onsite computer (onsite computer accessible only after entering patient details); Sealed envelopes (sequentially numbered, opaque or without further specification); Closed list (closed list of random numbers); Other (other specified method); Unspecified (stated as being 'randomised' but with no other details).

Blinding of investigators and patients: Single=single-blind; Double=double-blind; Open=open label.

Availability of data: Data available for at least one endpoint of interest in the meta-analyses.

In cases where the number randomised was not reported, the number evaluated was used instead. In addition, for crossover trials, where neither the number randomised nor the number evaluated was reported, the total number of participants divided by number of arms was used.

Webtable 7: Details of trials involving a randomised comparison of combinations of gastroprotectants

Trial name	Regimen	Ref.	Indication for treatment	Design	Allocation Concealment	Blinding of investigators and patients	Availability of data	No. of patients NX	NY
**Prevention Trials**									
Misoprostol + Ranitidine vs Ranitidine									
Yang 1996	800 ug vs Unknown	w1127	Renal transplant	Parallel	Unspecified method	Open	Yes	25	28
Rebamipide + Lansoprazole vs Lansoprazole									
Yoshida 2010	900 mg vs 0	w1128	Gastro-oesophageal reflux	Parallel	Unspecified method	Open	Yes	20	21
Rebamipide + Omeprazole vs Omeprazole									
Mizukami 2011	300 mg vs Unknown	w1129	Healthy volunteers	Crossover	Pharmacy	Double	Yes	6	6
**Healing Trials**									
Enprostil + Cimetidine vs Cimetidine									
Murata 2005	50 ug vs Unknown	w1130	Peptic ulcers	Parallel	Sealed envelopes	Unknown	Yes	85	86
Rebamipide + Lansoprazole vs Lansoprazole									
Saita 1996	300 mg vs Unknown	w1131	Peptic ulcers	Parallel	Unspecified method	Unknown	No	33	27
Rebamipide + Other PPI vs Rebamipide + Other H2RA									
Jang 2012	Unknown vs Unknown	w1132	Endoscopic submucosal dissection	Parallel	Unspecified method	Unknown	No	40	37
Rebamipide + Other PPI vs Other PPI									
Araki 2012	300 mg vs Unknown	w1133	Endoscopic submucosal dissection	Parallel	Unspecified method	Unknown	Yes	51	49
Kobayashi 2012	300 mg vs Unknown	w1134	Endoscopic submucosal dissection	Parallel	Closed list	Unknown	Yes	85	85
Jang 2012	Unknown vs Unknown	w1132	Endoscopic submucosal dissection	Parallel	Unspecified method	Unknown	No	40	44
**Acute Bleeding Trials**									
Misoprostol + Other PPI vs Other PPI									
Yilmaz 2007	600 ug vs Unknown	w1135	UGI bleeding of any cause	Parallel	Unspecified method	Unknown	Yes	56	67

Design: Data shown for crossover trials reflect only events during the first phase of the trial.

Allocation concealment: IVRS (Interactive Voice Response System); Telephone (telephone central office); Pharmacy (pharmacy coded containers administered sequentially); Onsite computer (onsite computer accessible only after entering patient details); Sealed envelopes (sequentially numbered, opaque or without further specification); Closed list (closed list of random numbers); Other (other specified method); Unspecified (stated as being ‘randomised’ but with no other details).

Blinding of investigators and patients: Double=double-blind; Open=open label.

Availability of data: Data available for at least one endpoint of interest in the meta-analyses.

In cases where the number randomised was not reported, the number evaluated was used instead. In addition, for crossover trials, where neither the number randomised nor the number evaluated was reported, the total number of participants divided by number of arms was used.

References for trials listed in Webtables 1-7

w1 Metz DC et al. Clinical trial: dexlansoprazole MR, a proton pump inhibitor with dual delayed-release technology, effectively controls symptoms and prevents relapse in patients with healed erosive oesophagitis. Aliment Pharm Therap 2009;29(7):742-54.

w2 Fass R et al. The effect of dexlansoprazole MR on nocturnal heartburn and GERD-related sleep disturbances in patients with symptomatic GERD. Am J Gastroenterology 2011;106(3):421-31.

w3 Howden CW et al. Clinical trial: efficacy and safety of dexlansoprazole MR 60 and 90mg in healed erosive oesophagitis - maintenance of healing and symptom relief. Aliment Pharm Therap 2009;30(9):895-907.

w4 Johnson DA et al. Esomeprazole once daily for 6m is effective therapy for maintaining healed erosive esophagitis and for controlling GERD symptoms: randomized, double-blind, placebo-controlled study of efficacy and safety. Am J Gast 2001;96(1):27-34.

w5 Vakil N et al. The new proton pump inhibitor esomeprazole is effective as a maintenance therapy in GERD patients with healed erosive oesophagitis: a 6-month, randomized, double-blind, placebo-controlled study of efficacy and safety. Aliment Pharm Therap 2001;15(7):927-35.

w6 Scheiman JM et al. Prevention of ulcers by esomeprazole in at-risk patients using non-selective NSAIDs and COX-2 inhibitors. Am J Gastroenterol 2006;101(4):701-10.

w7 Yeomans N et al. Efficacy of esomeprazole (20 mg once daily) for reducing the risk of gastroduodenal ulcers associated with continuous use of low-dose aspirin. Am J Gastroenterol 2008;103(10):2465-73.

w8 Hawkey C et al. Improvements with esomeprazole in patients with upper gastrointestinal symptoms taking non-steroidal anti-inflammatory drugs, including selective COX-2 inhibitors. Am J Gastroenterol 2005;100(5):1028-36.

w9 Johnson DA et al. Effect of esomeprazole on nighttime heartburn and sleep quality in patients with GERD: A randomized, placebo-controlled trial. Am J Gastroenterol 2005;100(9):1914-22.

w10 Hawkey CJ et al. Maintenance treatment with esomeprazole following initial relief of non-steroidal anti-inflammatory drug-associated upper gastrointestinal symptoms: the NASA2 and SPACE2 studies. Arthritis Res Ther 2007;9(1):17.

w11 Katz PO et al. Esomeprazole resolves chronic heartburn in patients without erosive oesophagitis. Aliment Pharm Therap 2003;18(9):875-82.

w12 Cheung CK et al. The effect of proton pump inhibitor (PPI) on plasma 5-hydroxytryptamine (5-HT) levels in patients with functional dyspepsia (FD): A double-blind randomized control trial. Gastroenterology. 2013;1:S679.

w13 Hsu PI et al. Esomeprazole prevents recurrent peptic ulcers in clopidogrel users with ulcer history. Gastroenterology 2010;1:S20.

w14 Yu L et al. Prevention effect of PPIs on induced gastrointestinal injury in CHD patients. J Gastroen Hepatol. 2013;28:13.

w15 Tan VP et al. Treatment of non-erosive reflux disease with a proton pump inhibitor in Chinese patients: a randomized controlled trial. Gastroenterology 2011;46(7):906-12.

w16 Sugano K et al. Randomised clinical trial: esomeprazole for the prevention of nonsteroidal anti-inflammatory drug-related peptic ulcers in Japanese patients. Aliment Pharm Therap 2012;36(2):115-25.

w17 Hsu PI. Proton pump inhibitor prevents clinically significant upper gastrointestinal events in clopidogrel users with ulcer history. Gastroenterology 2012;142:S27 (114).

w18 Sugano K et al. Multinational, double-blind, randomised, placebo-controlled, prospective study of esomeprazole in the prevention of recurrent peptic ulcer in low-dose acetylsalicylic acid users: the LAVENDER study. Gut 2014;63(7):1061-8.

w19 Peura DA et al. Esomeprazole treatment of frequent heartburn: two randomized, double-blind, placebo-controlled trials. Postgraduate Medicine 2014;126(4):33-41.

w20 Pilichiewicz AN et al. Symptoms and disease burden in patients with functional dyspepsia: An observational 12 month follow-up. J Gastroen Hepatol 2012;27:133-4.

w21 Hsu PI et al. Esomeprazole with clopidogrel reduces peptic ulcer recurrence, compared with clopidogrel alone, in patients with atherosclerosis. Gastroenterology 2011;140(3):791-8.

w22 Faruqi S et al. Chronic cough and esomeprazole: a double-blind placebo-controlled parallel study. Respirology. 2011;16(7):1150-6.

w23 Scheiman JM et al. Prevention of peptic ulcers with esomeprazole in patients at risk of ulcer development treated with low-dose acetylsalicylic acid: a randomised, controlled trial (OBERON). Heart. 2011;97(10):797-802.

w24 Johnson D et al. Clinical trial: esomeprazole for moderate-to-severe nighttime heartburn and gastro-oesophageal reflux disease-related sleep disturbances. Aliment Pharm Therap 2010;32(2):182-90.

w25 Reimer C et al. Proton-pump inhibitor therapy induces acid-related symptoms in healthy volunteers after withdrawal of therapy. Gastroenterology 2009;137:80-7.

w26 Wilder-Smith CH et al. Quantification of dental erosions in patients with GERD using optical coherence tomography before and after double-blind, randomized treatment with esomeprazole or placebo. Am J Gastroenterol 2009;104:2788-95.

w27 Chan FK et al. Combination of a cyclo-oxygenase-2 inhibitor and a proton-pump inhibitor for prevention of recurrent ulcer bleeding in patients at very high risk: a double-blind, randomised trial. Lancet 2007;369(9573):1621-6.

w28 Fass R et al. The effect of esomeprazole 20 mg twice daily on acoustic and perception parameters of the voice in laryngopharyngeal reflux. Neurogastroent Motil 2010;22(2):134-45.

w29 Johnsson F et al. Symptom-relieving effect of esomeprazole 40mg daily in patients with heartburn. Scand J Gastroentero 2003;38(4):347-53.

w30 Van Zanten SV et al. Esomeprazole 40mg once a day in patients with functional dyspepsia: the randomized, placebo-controlled "ENTER" trial.[Erratum appears in Am J Gastroenterol. 2006 Sep;101(9):2171]. Am J Gastroentero 2006;101(9):2096-106.

w31 Reichel O et al. Double-blind, placebo-controlled trial with esomeprazole for symptoms and signs associated with laryngopharyngeal reflux. Otolaryng Head Neck 2008;139(3):414-20.

w32 Talley NJ et al. Randomized-controlled trial of esomeprazole in functional dyspepsia patients with epigastric pain or burning: does a 1-week trial of acid suppression predict symptom response? Aliment Pharm Therap 2007;26(5):673-82.

w33 Yi CH et al. The effect of acid suppression on sleep and cardiac autonomic regulation in GERD. Hepato-Gastroenterol 2008;55(86-87):1649-52.

w34 Kiljander TO et al. Effect of esomeprazole 40mg once or twice daily on asthma: a randomized, placebo-controlled study. Am J Resp Crit Care 2010;181(10):1042-8.

w35 Hove KD et al. Effects of 12 weeks' treatment with a proton pump inhibitor on insulin secretion, glucose metabolism and markers of cardiovascular risk in patients with type 2 diabetes: a randomised double-blind prospective placebo-controlled study. Diabetologia 2013;56(1):22-30.

w36 Fernando H et al. Randomized double-blind placebo-controlled crossover study to determine the effects of esomeprazole on inhibition of platelet function by clopidogrel. J Thromb Haemost 2011;9(8):1582-9.

w37 Liuzzo J et al. Prospective, randomized, placebo-controlled evaluation of esomeprazole in coronary artery disease patients. EPAC: esomeprazole prevention of atypical chest pains. J Invasive Cardiol 2011;23(6):222-6.

w38 Meineche-Schmidt V et al. Randomised clinical trial: identification of responders to short-term treatment with esomeprazole for dyspepsia in primary care - a randomised, placebo-controlled study. Aliment Pharm Therap 2011;33(1):41-9.

w39 Goldstein JL et al. Clinical trial: the incidence of NSAID-associated endoscopic gastric ulcers in patients treated with PN 400 (naproxen plus esomeprazole magnesium) vs. enteric-coated naproxen alone. Aliment Pharm Therap 2010;32(3):401-13.

w40 Goldstein JL et al. Clinical trial: the incidence of NSAID-associated endoscopic gastric ulcers in patients treated with PN 400 (naproxen plus esomeprazole magnesium) vs. enteric-coated naproxen alone. Aliment Pharm Therap. 2010;32(3):401-13.

w41 Andersson T et al. Effect of esomeprazole with/without acetylsalicylic acid, omeprazole and lansoprazole on pharmacokinetics and pharmacodynamics of clopidogrel in healthy volunteers. Am J Cardiovasc Drugs 2014;14(3):217-27.

w42 Jiang QH et al. Efficacy of combined aluminum magnesium carbonate, esomeprazole and deanxit in treatment of gastroesophageal reflux disease in young or middle-aged patients with anxiety: An analysis of 60 cases. World Chinese Journal of Digestology 2013;21(23):2331-4. [Chinese]

w43 Mastronarde JG et al. Efficacy of esomeprazole for treatment of poorly controlled asthma. New Engl J Med 2009;360(15):1487-99.

w44 Vaezi MF et al. Treatment of chronic posterior laryngitis with esomeprazole. Laryngoscope 2006;116(2):254-60.

w45 Kiljander TO et al. Effects of esomeprazole 40mg twice daily on asthma: a randomized placebo-controlled trial. Am J Resp Crit Care 2006;173(10):1091-7.

w46 Dimango E et al. Effect of esomeprazole versus placebo on pulmonary exacerbations in cystic fibrosis. BMC Pulm Med. 2014;14:21.

w47 Shaheen NJ et al. Randomised clinical trial: high-dose acid suppression for chronic cough - a double-blind, placebo-controlled study. Aliment Pharm Therap 2011;33(2):225-34.

w48 Van Zanten SV et al. One-week acid suppression trial in uninvestigated dyspepsia patients with epigastric pain or burning to predict response to 8 weeks' treatment with esomeprazole: A randomized, placebo-controlled study. Aliment Pharm Therap 2007; 26:665-72.

w49 Byun M et al. Efficacy of proton pump inhibitor for chronic cough caused by gastroesophageal reflux: Preliminary results of a double-blind, placebo-controlled, randomized clinical trial. American Journal of Respiratory and Critical Care Medicine Conference: American Thoracic Society International Conference, ATS 2015;191.

w50 Peura DA et al. Clinical trial: Lansoprazole 15 or 30 mg once daily vs. Placebo for treatment of frequent nighttime heartburn in self-treating subjects. Aliment Pharm Therap 2009;30(5):459-68.

w51 Graham DY et al. Ulcer prevention in long-term users of nonsteroidal anti-inflammatory drugs: results of a double-blind, randomized, multicenter, active- and placebo-controlled study of misoprostol vs lansoprazole. Arch Intern Med 2002;162(2):169-75.

w52 Wong WM et al. Double blind, randomised, placebo controlled study of four weeks of lansoprazole for the treatment of functional dyspepsia in Chinese patients. Gut 2002;51(4):502-6.

w53 Hongo M et al. Efficacy and safety of lansoprazole (AG-1749) 15 mg and 30 mg in Japanese patients with non-erosive reflux disease (NERD): A phase III multicenter, double-blind, placebo-controlled trial. Jpn Pharmacol & Ther 2 2008;36:655-71. [Japanese]

w54 Kushner PR et al. Lansoprazole 15 mg once daily for 14 days is effective for treatment of frequent heartburn: results of 2 randomized, placebo-controlled, double-blind studies. Postgrad Med 2009;121(4):67-75.

w55 Peura DA et al. Lansoprazole in the treatment of functional dyspepsia: Two double-blind, randomized, placebo-controlled trials. Am J Med 2004;116(11):740-8.

w56 Sasaki T et al. A randomized, single-blind study of lansoprazole for the prevention of exacerbations of chronic obstructive pulmonary disease in older patients. J Am Geriatr Soc 2009;57(8):1453-7.

References for trials listed in Webtables 1-7

w57 Kovacs TOG et al. Double-blind comparison of lansoprazole 15 mg, lansoprazole 30 mg and placebo as maintenance therapy in patients with healed duodenal ulcers resistant to H2- receptor agonists. Aliment Pharm Therap 1999;13(7): 959-967.

w58 Muller P et al. Studies on the protective effect of lansoprazole on human gastric mucosa against low-dose acetylsalicylic acid. An endoscopic controlled double-blind study. Arzneimittel-Forsch 1997;47(6):758-60. [German]

w59 Earnest DL et al. A placebo-controlled dose-ranging study of lansoprazole in the management of reflux esophagitis. Am J Gastroenterol 1998;93(2):238-43.

w60 Castell DO et al. Efficacy and safety of lansoprazole in the treatment of erosive reflux esophagitis. The Lansoprazole Group. Am J Gastroenterol 1996;91(9):1749-57.

w61 Lanza F et al. Prevention of duodenal ulcer recurrence with 15mg lansoprazole: a double-blind placebo-controlled study. The Lansoprazole Study Group. Digest Dis Sci 1997;42(12):2529-36.

w62 Kovacs TO et al. Double-blind comparison of lansoprazole 15mg, lansoprazole 30mg, and placebo in the maintenance of healed gastric ulcer. Digest Dis Sci 1998;43(4):779-85.

w63 Sontag SJ et al. Lansoprazole prevents recurrence of erosive reflux esophagitis previously resistant to H2-RA therapy. The Lansoprazole Maintenance Study Group. Am J Gastroenterol 1996;91(9):1758-65.

w64 Robinson M et al. Effective maintenance treatment of reflux esophagitis with low-dose lansoprazole. A randomized, double-blind, placebo-controlled trial. Ann Intern Med 1996;124(10):859-67.

w65 Richter JE et al. Lansoprazole in the treatment of heartburn in patients without erosive oesophagitis. Aliment Pharm Therap 1999;13(6):795-804.

w66 Russo A et al. Bedtime administration of lansoprazole does not modify its greater efficacy vs ranitidine in the acute and long-term treatment of duodenal ulcer. Results from a multicentre, randomised, double blind clinical trial. Ital J Gastroenterol Hepatol 1997;29(4):312-9.

w67 Richter JE et al. Lansoprazole compared with ranitidine for the treatment of nonerosive gastroesophageal reflux disease. Arch Intern Med 2000;160(12):1803-9.

w68 Suzuki H et al. Effect of lansoprazole on the Epigastric symptoms of functional dyspepsia (ELF study): A multicentre, prospective, randomized, double-blind, placebo-controlled clinical trial. United European Gastroenterology Journal 2013;1(6):445-52.

w69 Petite JP et al. The effects of lansoprazole on the function of the lower esophagus function and esophageal motility in man. Med Chir Dig 1991;20(1):58-60. [French]

w70 Bell N et al. Duration of effect of lansoprazole on gastric pH and acid secretion in normal male volunteers. Aliment Pharm Therap 2001;15(1):105-13.

w71 Brummer RJ et al. Initial and chronic gastric acid inhibition by lansoprazole and omeprazole in relation to meal administration. Digest Dis Sci 1997;42(10):2132-7.

w72 Dumper J et al. Lansoprazole in globus pharyngeus: double-blind, randomized, placebo-controlled trial. J Ototlarngol-Head N 2008;37(5):657-63.

w73 Gaetani M et al. Chronic oral administration of lansoprazole does not affect the hypothalamic pituitary gonadal axis in healthy young men. Eur J Gastroen Hepat 1995;7(3):211-3.

w74 Jang JY et al. Randomized prospective trial of the effect of induced hypergastrinemia on the prevention of pancreatic atrophy after pancreatoduodenectomy in humans. Ann Surg 2003;237(4):522-9.

w75 Lai KC et al. Lansoprazole reduces ulcer relapse after eradication of Helicobacter pylori in nonsteroidal anti-inflammatory drug users-a randomized trial. Aliment Pharm Therap 2003;18(8):829-36.

w76 Pakozdi A et al. Does long term therapy with lansoprazole slow progression of oesophageal involvement in systemic sclerosis? Clin Exp Rheumatol 2009;27(3)(Suppl.):8.

w77 Xia HH et al. Symptomatic response to lansoprazole predicts abnormal acid reflux in endoscopy-negative patients with non-cardiac chest pain. Aliment Pharm Therap 2003;17(3):369-77.

w78 Leung WK et al. Initial treatment with lansoprazole in young dyspeptic patients with negative urea breath test result: a randomized controlled trial with 12-month follow-up. Am J Gastroenterol 2007;102(7):1483-8.

w79 Lai KC et al. Lansoprazole for the prevention of recurrences of ulcer complications from long-term low-dose aspirin use. N Engl J Med 2002;346(26):2033-8.

w80 Collet JP et al. Prasugrel but not high dose clopidogrel overcomes the lansoprazole neutralizing effect of P2Y12 inhibition: Results of the randomized DOSAPI study. Eur J Clin Pharmacol 2014:70(9):1049-1057.

w81 Talwar V et al. Clinical trial: chest pain caused by presumed gastro-oesophageal reflux in coronary artery disease - controlled study of lansoprazole vs. placebo. Alimentary Pharmacology & Therapeutics 2010;32(2):191-9.

w82 Zhang J R et al. Efficacy of clopidogrel and clinical outcome when clopidogrel is coadministered with atorvastatin and lansoprazole: A prospective, randomized, controlled trial. Medicine (United States) 2015 ;94(50).

w83 Feng Y et al. Efficacy of lansoprazole combined with conventional treatment in relieving gastrointestinal symptoms in patients with anxiety. World Chinese J Digestology 2014;22(26):4012-6. [Chinese]

w84 Fletcher J et al. BMI is superior to symptoms in predicting response to proton pump inhibitor: randomised trial in patients with upper gastrointestinal symptoms and normal endoscopy. Gut 2011;60(4):442-8.

w85 Jones GR et al. BMI is superior to symptom characteristics or esophageal pH metry in predicting response to PPI therapy in patients with normal endoscopy. Gastroenterology 2010;(1):S562.

w86 Adamopoulos AB et al. Do proton pump inhibitors attenuate the effect of aspirin on platelet aggregation? A randomized crossover study. J Cardiovasc Pharm 2009;54(2):163-8.

w87 Littner MR et al. Effects of 24 weeks of lansoprazole therapy on asthma symptoms, exacerbations, quality of life, and pulmonary function in adult asthmatic patients with acid reflux symptoms. Chest 2005;128(3):1128-35.

w88 Havas T. Posterior pharyngolaryngitis. Double-blind randomised placebo-controlled trial of proton pump inhibitor therapy. Aust J Otoloryngology 1999;3(3):243-6.

w89 El-Serag HB et al. Lansoprazole treatment of patients with chronic idiopathic laryngitis: A placebo-controlled trial. Am J Gastroenterol 2001;96(4):979-83.

w90 Borjesson M et al. Nutcracker oesophagus: a double-blind, placebo-controlled, cross-over study of the effects of lansoprazole. Aliment Pharm Therap 2003;18(11-12):1129-35.

w91 Fuchs W et al. Lansoprazole does not affect the bioavailability of oral contraceptives. Brit J Clin Pharmaco 1994;38(4):376-80.

w92 Meikle AW et al. Effect of lansoprazole on male hormone function. Drug Invest 1994;8(4):191-202.

w93 Vaezi MF et al. Proton pump inhibitor therapy improves symptoms in postnasal drainage. Gastroenterology 2010;139(6):1887-93.e1; e11.

w94 Takahiko S et al. The proton pump inhibitor, lansoprazole, reduces frequency of exacerbations in patients with chronic obstructive pulmonary disease. Chest Conference: Chest. 2011;140.

w95 Takebayashi K et al. Effect of combination therapy with alogliptin and lansoprazole on glycemic control in patients with type 2 diabetes. Endocr J 2014;61(10):1031-9.

w96 Inukai T et al. Effect of combination therapy with alogliptin and lansoprazole on glycemic control in patients with type 2 diabetes. Diabetes 2015;64:A657.

w97 Farup PG et al. Patients with functional dyspepsia responding to omeprazole have a characteristic gastro-oesophageal reflux pattern. Scand J Gastroentero 1999;34(6):575-9.

w98 K D Bardhan Erosive Oesophagitis: outcome of repeated long term maintenance treatment with low dose omeprazole 10 mg or placebo. Gut 1998;43:458-64.

w99 Talley NJ et al. Efficacy of omeprazole in functional dyspepsia: double-blind, randomized, placebo-controlled trials (the Bond and Opera studies). Aliment Pharm Therap 1998;12(11):1055-65.

w100 Lauritsen K et al. Omeprazole 20 mg three days a week and 10 mg daily in prevention of duodenal ulcer relapse: Double-blind comparative trial. Gastroenterology 1991;100(3):663-9.

w101 Carlsson R et al. Gastro-oesophageal reflux disease in primary care: an international study of different treatment strategies with omeprazole. International GORD Study Group. Eur J Gastroen Hepat 1998;10(2):119-24.

w102 Bate CM et al. Omeprazole 10mg or 20mg once daily in the prevention of recurrence of reflux oesophagitis. Solo Investigator Group. Gut 1995;36(4):492-8.

w103 Allgood LD et al. Comparison of Prilosec OTC (omeprazole magnesium 20.6 mg) to placebo for 14 days in the treatment of frequent heartburn. J Clin Pharm Ther 2005;30(2):105-12.

w104 Blum AL et al. Short course acid suppressive treatment for patients with functional dyspepsia: results depend on Helicobacter pylori status. The Frosch Study Group. Gut 2000;47(4):473-80.

w105 Richter JE et al. Efficacy of omeprazole for the treatment of symptomatic acid reflux disease without esophagitis. Arch Intern Med 2000;160(12):1810-6.

w106 Venables TL et al. Maintenance treatment for gastro-oesophageal reflux disease: A placebo-controlled evaluation of 10 milligrams omeprazole once daily in general practice. Scand J Gastroentero 1997;32(7):627-32.

w107 Laursen LS et al. Omeprazole in the long-term treatment of gastro-oesophageal reflux disease. A double-blind randomized dose-finding study. Scand J Gastroentero 1995;30(9):839-46.

w108 Lind T et al. Heartburn without oesophagitis: efficacy of omeprazole therapy and features determining therapeutic response. Scand J Gastroentero 1997;32(10):974-9.

w109 Uemura N et al. Efficacy and safety of omeprazole in Japanese patients with nonerosive reflux disease. J Gastroenterol 2008;43(9):670-8.

w110 Cullen D et al. Primary gastroduodenal prophylaxis with omeprazole for non-steroidal anti-inflammatory drug users. Aliment Pharm Therap 1998;12:135-40.

w111 Ekström P et al. Prevention of peptic ulcer and dyspeptic symptoms with omeprazole in patients receiving continuous non-steroidal anti-inflammatory drug therapy. A nordic multicentre study. Scand J Gastroentero 1996;31(8):753-8.

w112 Bianchi Porro G et al. Prevention of gastroduodenal damage with omeprazole in patients receiving continuous NSAIDs treatment. A double blind placebo controlled study. Ital J Gastroenterol 1998;30(1):43-7.

w113 Hawkey CJ et al. Omeprazole compared with misoprostol for ulcers associated with nonsteroidal anti-inflammatory drugs. Omeprazole versus Misoprostol for NSAID-induced Ulcer Management (OMNIUM) Study Group. New Engl J Med 1998;338(11):727-34.

w114 Brun J et al. High dose proton pump inhibitor response as an initial strategy for a clinical diagnosis of gastro-oesophageal reflux disease (GERD). Fam Pract 2000;17(5):401-4.

w115 Sgouros SN et al. Long-term acid suppressive therapy may prevent the relapse of lower esophageal (Schatzki's) rings: A prospective, randomized, placebo-controlled study. Am J Gastroenterol 2005;100(9):1929-34.

References for trials listed in Webtables 1-7

w116 Meineche-Schmidt V. Empiric treatment with high and standard dose of omeprazole in general practice: Two-week randomized placebo-controlled trial and 12-month follow-up of health-care consumption. Am J Gastroenterol 2004;99(6):1050-8.

w117 Veldhuyzen van ZS et al. Adding once-daily omeprazole 20 mg to metronidazole/amoxicillin treatment for Helicobacter pylori gastritis: A randomized, double-blind trial showing the importance of metronidazole resistance. Am J Gastroenterol 1998;93(1):5-10.

w118 Bhatt DL et al. Clopidogrel with or without omeprazole in coronary artery disease. N Engl J Med 2010;363:1909-17.

w119 Ishizaki T et al. Comparison of the interaction potential of a new proton pump inhibitor, E3810, versus omeprazole with diazepam in extensive and poor metabolizers of S-mephenytoin 4'-hydroxylation. Clin Pharmacol Ther 1995;58(2):155-64.

w120 Marzio L et al. Short- and long-term omeprazole for the treatment and prevention of duodenal ulcer, and effect on Helicobacter pylori. Am J Gastroenterol 1995;90(12):2172-6.

w121 Hetzel DJ et al. Healing and relapse of severe peptic esophagitis after treatment with omeprazole. Gastroenterology 1988;95(4):903-12.

w122 Goh KL et al. Prevention of duodenal ulcer relapse with omeprazole 20 mg daily: a randomized double-blind, placebo-controlled study. J Gastroen Hepatol 1995;10(1):92-7.

w123 Ford GA et al. Omeprazole in the treatment of asthmatics with nocturnal symptoms and gastro-oesophageal reflux: a placebo-controlled cross-over study. Postgrad Med J 1994;70(823):350-4.

w124 Blohme I et al. A study of the interaction between omeprazole and cyclosporine in renal transplant patients. Brit J Clin Pharmaco 1993;35(2):156-60.

w125 Guslandi M et al. Omeprazole-induced changes in gastric mucus secretion. Method Find Exp Clin 1992;14(3):219-23.

w126 Garg PK et al. Role of omeprazole in prevention and treatment of postendoscopic variceal sclerotherapy esophageal complications. Double-blind randomized study. Digest Dis Sci 1995;40(7):1569-74.

w127 Gerson LB et al. A prospective study of oesophageal 24-h ambulatory pH monitoring in patients with functional dyspepsia. Digest Liver Dis 2005;37(2):87-91.

w128 Hatlebakk JG et al. Heartburn treatment in primary care: randomised, double blind study for 8 weeks. Brit Med J 1999;319(7209):550-3.

w129 Sutfin T et al. Stereoselective interaction of omeprazole with warfarin in healthy men. Ther Drug Monit 1989;11(2):176-84.

w130 Unge P et al. A study of the interaction of omeprazole and warfarin in anticoagulated patients. Brit J Clin Pharmaco 1992;34(6):509-12.

w131 Sartori S et al. Randomized trial of omeprazole or ranitidine versus placebo in the prevention of chemotherapy-induced gastroduodenal injury. J Clin Oncol 2000;18(3):463-7.

w132 Sontag SJ et al. Two doses of omeprazole versus placebo in symptomatic erosive esophagitis: the U.S. Multicenter Study. Gastroenterology 1992;102(1):109-18.

w133 Simon B et al. Protective effects of omeprazole against low-dose acetylsalicylic acid. An endoscopic controlled double blind study in healthy volunteers. Arzneimittel-Forschung/Drug Research 1995;45(6):701-3. [German].

w134 Sontag SJ et al. Daily omeprazole surpasses intermittent dosing in preventing relapse of oesophagitis: a US multi-centre double-blind study. Aliment Pharm Therap 1997;11(2):373-80.

w135 Howard JM et al. Macroscopic healing of esophagitis does not improve esophageal motility. Digest Dis Sci 1994;39(3):648-54.

w136 Rokkas T et al. Effectiveness of acid suppression in preventing gastroesophageal reflux disease (GERD) after successful treatment of Helicobacter pylori infection. Digest Dis Sci 2001;46(7):1567-72.

w137 Bate CM et al. Reflux symptom relief with omeprazole in patients without unequivocal esophagitis. Aliment Pharm Therap 1996;10(4):547-55.

w138 Heijerman HG et al. Improvement of fecal fat excretion after addition of omeprazole to pancrease in cystic fibrosis is related to residual exocrine function of the pancreas. Digest Dis Sci 1993;38(1):1-6.

w139 Levin TR et al. Omeprazole improves peak expiratory flow rate and quality of life in asthmatics with gastroesophageal reflux. Am J Gastroenterol 1998;93(7):1060-3.

w140 Meineche-Schmidt V et al. Anti-secretory therapy in 1017 patients with ulcerlike or refluxlike dyspepsia in general practice. Eur J of General Practice 1997;3:125-30.

w141 Houben GMP et al. Twenty-four hour intragastric acidity: 300mg ranitidine b.d., 20mg omeprazole o.m., 40mg omeprazole o.m. vs. placebo. Aliment Pharmacol Ther 1995;9:649-54.

w142 Ostovaneh MR et al. Comparing omeprazole with fluoxetine for treatment of patients with heartburn and normal endoscopy who failed once daily proton pump inhibitors: double-blind placebo-controlled trial. Neurogastroenterology & Motility. 2014;26(5):670-8.

w143 Aimi M et al. Effects of omeprazole on sleep disturbance: Randomized multicenter double-blind placebo-controlled trial. Clinical and Translational Gastroenterology 2014;5 (6):(e57).

w144 Stephenson KA and Fagan JJ. Effect of perioperative proton pump inhibitors on the incidence of pharyngocutaneous fistula after total laryngectomy: a prospective randomized controlled trial. Head Neck. 2015;37(2):255-9.

w145 Ren Y H et al. 2011 Omeprazole affects clopidogrel efficacy but not ischemic events in patients with acute coronary syndrome undergoing elective percutaneous coronary intervention. Chinese Medical Journal 124(6):856-861.

w146 Dammann HG et al. Intragastric acidity under 28-day omeprazole treatment. Hepato-Gastroenterol 1985;32(4):191-4.

w147 Maev IV et al. Clinico-functional evaluation of the efficacy of treating gastroesophageal reflux disease combined with bronchial asthma with omeprazole. Terapevt Arkh 2002;74(8):55-7. [Russian]

w148 Bolling-Sternevald E et al. Effect of profound acid suppression in functional dyspepsia: A double-blind, randomized, placebo-controlled trial. Scand J Gastroentero 2002;37(12):1395-402.

w149 Laine L et al. US double-blind, controlled trials of omeprazole and amoxycillin for treatment of Helicobacter pylori. Aliment Pharm Therap 1998;12(4):377-82.

w150 Rabeneck L et al. A double blind, randomized, placebo-controlled trial of proton pump inhibitor therapy in patients with uninvestigated dyspepsia. Am J Gastroenterol 2002;97(12):3045-51.

w151 Kiljander TO et al. Gastroesophageal reflux in asthmatics: A double-blind, placebo- controlled crossover study with omeprazole. Chest 1999;116(5):1257-64.

w152 Scheiman JM et al. Omeprazole ameliorates aspirin-induced gastroduodenal injury. Digest Dis Sci 1994;39(1):97-103.

w153 Achem SR et al. Effects of omeprazole versus placebo in treatment of noncardiac chest pain and gastroesophageal reflux. Digest Dis Sci 1997;42(10):2138-45.

w154 Caraco Y et al. Interethnic difference in omeprazole's inhibition of diazepam metabolism. Clin Pharmacol Ther 1995;58(1):62-72.

w155 Bortolotti M et al. Obstructive sleep apnoea is improved by a prolonged treatment of gastrooesophageal reflux with omeprazole. Digest Liver Dis 2006;38(2):78-81.

w156 Dorta G et al. The effects of omeprazole on healing and appearance of small gastric and duodenal lesions during dosing with diclofenac in healthy subjects. Aliment Pharm Therap 2000;14(5):535-41.

w157 Kiljander TO et al. Chronic cough and gastro-oesophageal reflux: a double-blind placebo-controlled study with omeprazole. Eur Respir J 2000;16(4):633-8.

w158 Fallahi GH et al. The effect of omeprazole on asthmatic adolescents with gastroesophageal reflux disease.[Erratum appears in Allergy Asthma Proc. 2008 Nov-Dec;29(6):675]. Allergy Asthma Proc 2008;29(5):517-20.

w159 Meier JH et al. Does omeprazole (Prilosec) improve respiratory function in asthmatics with gastroesophageal reflux? A double-blind, placebo-controlled crossover study. Digest Dis Sci 1994;39(10):2127-33.

w160 Sharma B et al. Acid secretory capacity and plasma gastrin concentration after administration of omeprazole to normal subjects. Aliment Pharm Therap 1987;1(1):67-76.

w161 Teichtahl H et al. Adult asthma and gastro-oesophageal reflux: the effects of omeprazole therapy on asthma. Aust NZ J Med 1996;26(5):671-6.

w162 Rabeneck L et al. SODA (severity of dyspepsia assessment): a new effective outcome measure for dyspepsia-related health. J Clin Epidemiol 2001;54(8):755-65.

w163 Unge P et al. Does omeprazole improve antimicrobial therapy directed towards gastric Campylobacter pylori in patients with antral gastritis? A pilot study. Scand J Gastroentero 1989;167(Suppl.):49-54.

w164 Watson RG et al. Double blind cross-over placebo controlled study of omeprazole in the treatment of patients with reflux symptoms and physiological levels of acid reflux-the "sensitive oesophagus". Gut 1997;40(5):587-90.

w165 Serfaty-Lacrosniere C et al. Hypochlorhydria from short-term Omeprazole treatment does not inhibit intestinal absorption of calcium, phosphorous, magnesium or zinc from food in humans. J Am Coll Nutr 1995;14(4):364-8.

w166 Budzynski J et al. The effect of double dose of omeprazole on the course of angina pectoris and treadmill stress test in patients with coronary artery disease - A randomised, double-blind, placebo controlled, crossover trial. Int J Cardiol 2008;127(2):233-9.

w167 Chen YQ et al. Efficacy of Jiangni Decoction in treatment of recurrent reflux esophagitis. World Chinese J Digestol 2013;21(21):2076-80. [Chinese]

w168 Emami M et al. Efficacy of omeprazole on cough, Pulmonary function and quality of life of patients with sulfur mustard lung injury: A placebo-control, Cross-over clinical trial study. Journal of Research in Medical Sciences 2014;19(11):1027-33.

w169 Poh CH et al. The effect of antireflux treatment on patients with gastroesophageal reflux disease undergoing a mental arithmetic stressor. Neurogastroent Motil 2011;23(11):e489-96.

w170 Whellan DJ et al. PA32540 (a coordinated-delivery tablet of enteric-coated aspirin 325 mg and immediate-release omeprazole 40 mg) versus enteric-coated aspirin 325 mg alone in subjects at risk for aspirin-associated gastric ulcers: results of two 6-month, phase 3 studies. Am Heart J 2014;168(4):495-502.e4.

w171 Goldstein JL et al. Prevalence of gastric ulcers in aspirin users and does the presence of erosions foreshadow ulcer development. Gastroenterology 2013;(1):S565.

w172 Cai J et al. 2010 Impact of different proton pump inhibitors on the antiplatelet activity of clopidogrel in combination with aspirin for patients undergoing coronary stent implantation. Zhongguo Ying Yong Sheng Li Xue Za Zhi= Chinese J Applied Physiol 26(3):266-9. [Chinese]

w173 Boeree MJ et al. No effects of high-dose omeprazole in patients with severe airway hyperresponsiveness and (a)symptomatic gastro-oesophageal reflux. Eur Respir J 1998;11(5):1070-4.

w174 Noordzij JP et al. Evaluation of omeprazole in the treatment of reflux laryngitis: a prospective, placebo-controlled, randomized, double-blind study. Laryngoscope 2001;111(12):2147-51.

w175 Pandak WM et al. Short course of omeprazole: a better first diagnostic approach to noncardiac chest pain than endoscopy, manometry, or 24-hour esophageal pH monitoring. J Clin Gastroenterol 2002;35(4):307-14.

References for trials listed in Webtables 1-7

w176 Ours TM et al. A prospective evaluation of esophageal testing and a double-blind, randomized study of omeprazole in a diagnostic and therapeutic algorithm for chronic cough. Am J Gastroenterol 1999;94(11):3131-8.

w177 Freedberg DE et al. Proton pump inhibitors alter specific taxa in the human fecal microbiome: Results of a crossover trial. Gastroenterology 2015;(1):S619.

w178 Dabirmoghadam P et al. The efficacy of n-acetyl cysteine in reflux laryngitis. Otolaryngology - Head and Neck Surgery (United States). 2012;147:77.

w179 Richter JE et al. Oral pantoprazole for erosive esophagitis: A placebo-controlled, randomized clinical trial. Am J Gastroenterol 2000;95(11):3071-80.

w180 Pilotto A et al. Short and long-term therapy for reflux oesophagitis in the elderly: A multi-centre, placebo-controlled study with pantoprazole. Aliment Pharm Therap 2003;17(11):1399-406.

w181 Van Rensberg et al. Efficacy and safety of pantoprazole 20mg once daily treatment in patients with ulcer-like functional dyspepsia. Curr Med Res Opin 2008;24(7):2009-18.

w182 Holtmann G et al. Improvement of non-steroidal anti-inflammatory drug-induced gastrointestinal symptoms during proton pump inhibitor treatment: are G-protein ß3 subunit genotype, Helicobacter pylori status, and environmental factors response modifiers? Digestion 2011;84(4):289-98.

w183 van der Velden AW et al. Pharmacological dependency in chronic treatment of gastroesophageal reflux disease: a randomized controlled clinical trial. Digestion 2010;81(1):43-52.

w184 Bianchi Porro G et al. Efficacy of pantoprazole in the prevention of peptic ulcers, induced by non-steroidal anti-inflammatory drugs: A prospective, placebo-controlled, double-blind, parallel-group study. Digest Liver Dis 2000;32(3):201-8.

w185 Dammann HG et al. Effects of pantoprazole on endocrine function in healthy male volunteers. Aliment Pharm Therap 1994;8(5):549-54.

w186 Dos Santos LH et al. Evaluation of pantoprazol treatment response of patients with asthma and gastroesophageal reflux: a randomized prospective double-blind placebo-controlled study. Jornal Brasileiro De Pneumologia E Tisilogia 2006;33(2):119-27. [Brazilian].

w187 Dabos KJ et al. The effect of mastic gum on Helicobacter pylori: a randomized pilot study. Phytomedicine 2010;17(3-4):296-9.

w188 Wo JM et al. Double-blind, placebo-controlled trial with single-dose pantoprazole for laryngopharyngeal reflux. Am J Gastroentero 2006;101(9):1972-8.

w189 Schwartz MP et al. Effect of inhibition of gastric acid secretion on antropyloroduodenal motor activity and duodenal acid hypersensitivity in functional dyspepsia. Aliment Pharm Therap 2001;15(12):1921-8.

w190 Muller P et al. The action of the proton pump inhibitor pantoprazol against acetylsalicylic acid-induced gastroduodenopathy in comparison to ranitidine. An endoscopic controlled, double blind comparison. Arzneimittel-Forschung 1998;48(5):482-5. [German]

w191 Suurna MV et al. Randomized placebo-controlled trial of pantoprazole for daytime sleepiness in GERD and obstructive sleep disordered breathing. Otolaryngol Head Neck Surg 2008;139(2):286-90.

w192 Cervelli MJ, Shaman A, Meade A, Carroll R, McDonald SP. Effect of gastric acid suppression with pantoprazole on the efficacy of calcium carbonate as a phosphate binder in haemodialysis patients. Nephrology 2012;17(5):458-65.

w193 Niklasson A, Lindstrom L, Simren M, Lindberg G, Bjornsson E. Dyspeptic symptom development after discontinuation of a proton pump inhibitor: a double-blind placebo-controlled trial. American Journal of Gastroenterology 2010;105(7):1531-7.

w194 Baysal B et al. Effect of pantoprazole and Helicobacter pylori therapy on uninvestigated dyspeptic patients. Turkish Journal of Gastroenterology 2015;26(1):6-14.

w195 Gonzalez-Ortiz M et al. Effect of pantoprazole on insulin secretion in drug-naïve patients with type 2 diabetes. Diabetes Res Clin Pract. 2015;108(1):e11-3.

w196 Lai B et al. Effect of gastric acid suppression with pantoprazole on the efficacy of sevelamer hydrochloride as a phosphate binder in haemodialysis patients: a pilot study. Nephrology 2012;17(4):402-6.

w197 Yu L W et al. Wang 2013 Mechanism and preventive effect of teprenone on dual anti-platelet drugs in CVD patients. Journal of Gastroenterology and Hepatology 28:317-318.

w198 Yang ZY. Efficacy and safety of pantoprazole sodium entericcoated tablets combined with mosapride in treatment of functional dyspepsia. World Chinese Journal of Digestology 2014;22(29):4514-8. [Chinese]

w199 Zhao J H et al. Lu 2015 Pantoprazole for prevention and treatment of gastrointestinal bleeding in elderly patients after percutaneous coronary intervention. [Chinese]. World Chinese Journal of Digestology 23(13):2111-2115.

w200 Eherer AJ et al. Effect of pantoprazole on the course of reflux-associated laryngitis: A placebo-controlled double-blind crossover study. Scand J Gastroentero 2003;38(5):462-7.

w201 Singh PK et al. Pantoprazole improves glycemic control in type 2 diabetes: a randomized, double-blind, placebo-controlled trial. J Clin Endocrinol Metab 2012;97(11):E2105-8.

w202 Kinoshita Y et al. Japan Rabeprazole Study Group for NERD. Randomised clinical trial: a multicentre, double-blind, placebo-controlled study on the efficacy and safety of rabeprazole 5 mg or 10 mg once daily in patients with non-erosive reflux disease. Aliment Pharm & Therap 2011;33(2):213-24.

w203 Caos A et al. Long-term prevention of erosive or ulcerative gastro-oesophageal reflux disease relapse with rabeprazole 10 or 20mg vs. Placebo: results of a 5-year study in the United States. Aliment Pharm Therap 2005;22(3):193-202.

w204 Caos A et al. Rabeprazole for the prevention of pathologic and symptomatic relapse of erosive or ulcerative gastroesophageal reflux disease. Rebeprazole Study Group. Am J Gastroenterol 2000;95(11):3081-8.

w205 Cloud ML et al. Rabeprazole in treatment of acid peptic diseases: results of three placebo-controlled dose-response clinical trials in duodenal ulcer, gastric ulcer, and gastroesophageal reflux disease (GERD). The Rabeprazole Study Group. Digest Dis Sci 1998;43(5):993-1000.

w206 Birbara C et al. Rabeprazole for the prevention of recurrent erosive or ulcerative gastro-oesophageal reflux disease. Rabeprazole Study Group. Eur J Gastroen Hepat 2000;12(8):889-97.

w207 Miner P, Jr. et al. Rabeprazole in nonerosive gastroesophageal reflux disease: a randomized placebo-controlled trial. Am J Gastroenterol 2006;97(6):1332-9.

w208 Tominaga K, et al. Differences of influencing factors of PPI and placebo responder in functional dyspepsia: Clinical advantage of effect on suppression of acid with rabeprazole in functional dyspepsia (the CAESAR study). Gastroenterology 2011;1):S884.

w209 Tanaka M et al. The therapeutic effectiveness of the coadministration of weekly risedronate and proton pump inhibitor in osteoporosis treatment. J Osteoporosis 2014;2014;607145.

w210 Munesue M et al. Rabeprazole improves symptoms of patients with functional dyspepsia: Multicenter, double-blind, randomized, placebo-controlled clinical trial; samurai study. Gastroenterology 2012;1):S171.

w211 Hidaka H et al. Long-term administration of PPI reduces treatment failures after esophageal variceal band ligation. Gastroenterological Endoscopy 2012;54:834.

w212 Itoh S et al. The effects of risedronate administered in combination with a proton pump inhibitor for the treatment of osteoporosis. J Bone Miner Metab 2013;31(2):206-11.

w213 Iwakiri R et al. Randomised clinical trial: rabeprazole improves symptoms in patients with functional dyspepsia in Japan. Aliment Pharm Therap 2013;38(7):729-40.

w214 Chen H et al. Rabeprazole combined with hydrotalcite is effective for patients with bile reflux gastritis after cholecystectomy. Can J Gastroenterol 2010;24(3):197-201.

w215 Dammann HG et al. The effects of oral rabeprazole on endocrine and gastric secretory function in healthy volunteers. Aliment Pharm Therap 1999;13(9):1195-203.

w216 Laheij RJ et al. Proton-pump inhibitor therapy for acetylsalicylic acid associated upper gastrointestinal symptoms: a randomized placebo-controlled trial. Aliment Pharm Therap 2003;18(1):109-15.

w217 van Rossum LG et al. Health-related quality of life in patients with cardiovascular disease-the effect of upper gastrointestinal symptom treatment. Aliment Pharm Therap 2004;19(10):1099-104.

w218 Santarelli L et al. Post-cholecystectomy alkaline reactive gastritis: a randomized trial comparing sucralfate versus rabeprazole or no treatment. Eur J Gastroen Hepat 2003;15(9):975-9.

w219 Johanson J et al. Rabeprazole significantly improves heartburn vs placebo in endoscopically negative GERD patients with moderate to severe symptoms. Am J Gastroenterol 2001;96(9)(Suppl.):18.

w220 Chan FKL et al. Rabeprazole for the prevention of gastroduodenal injury associated with nonsteroidal anti-inflammatory drugs (NSAIDS): A double-blind randomized trial. Gastroenterology 2014;(1):S-208.

w221 Katare SS et al. A prospective, comparative, randomized, controlled, open label, 3-arm study for evaluation of efficacy, safety and quality of life with troxipide and rabeprazole given alone and in combination in patients of gastritis. Indian J Pharmacol 2013;45:S12.

w222 Sun Q and Yao HX. Clinical observation of rabeprazole combined with flupentixol and melitracen treatment to non-erosive gastroesophageal reflux disease. Journal of Shanghai Jiaotong University (Medical Science). 2014;34(3):352-6+60. [Chinese]

w223 Peterson KA et al. The role of gastroesophageal reflux in exercise-triggered asthma: a randomized controlled trial. Digest Dis Sci 2008;54(3):564-71.

w224 Pawar S et al. Treatment of postnasal drip with proton pump inhibitors: A prospective, randomized, placebo-controlled study. Am J Rhinol 2007;21(6):695-701.

w225 Steward DL et al. Proton pump inhibitor therapy for chronic laryngo-pharyngitis: a randomized placebo-control trial. Otolaryng Head Neck 2004;131(4):342-50.

w226 Lam PKY et al. Rabeprazole is effective in treating laryngopharyngeal reflux in a randomized placebo-controlled trial. Clinical Gastroenterology and Hepatology 2010;8(9):770-6.

w227 Leelayuthachai T et al. A randomized, placebo-controlled trial of proton-pump inhibitor (PPI) on sleep parameters in patients with coexisting abnormal esophageal acid exposure and obstructive sleep apnea (OSA). Chest Conference: CHEST 2012;142.

w228 Johannson J et al. Impact of proton pump inhibitors on benign anastomotic stricture formations after esophagectomy and gastric tube reconstruction: results from a randomized clinical trial. Ann Surg 2009;250:667-73.

w229 Holtmann G et al. A placebo controlled randomised treatment trial for functional dyspepsia including post-treatment drug withdraw and placebo withdraw effects. Gastroenterology 2013;S733.

w230 Tulassay Z et al. Effect of esomeprazole triple therapy on eradication rates of Helicobacter pylori, gastric ulcer healing and prevention of relapse in gastric ulcer patients. Eur J Gastroen Hepat 2008;20(6):526-36.

w231 Avner DL et al. A comparison of three doses of lansoprazole (15, 30 and 60 mg) and placebo in the treatment of duodenal ulcer. The Lansoprazole Study Group. Aliment Pharm Therap 1995;9(5):521-8.

w232 Avner DL et al. Comparison of once daily doses of lansoprazole (15, 30, and 60 mg) and placebo in patients with gastric ulcer. Am J Gastroenterol 1995;90(8):1289-94.

w233 Jhala NC et al. The effects of short-term lansoprazole therapy on Helicobacter pylori infection and antral gastritis in duodenal ulcer patients. Am J Gastroenterol 1995;90(10):1824-8.

w234 Lanza F et al. Double-blind comparison of lansoprazole, ranitidine, and placebo in the treatment of acute duodenal ulcer. Am J Gastroenterol 1994;89(8):1191-200.

w235 Fu L and M. Luo 2012 Therapeutic effect of continuing anti-secretory treatment after. Chinese J Gastroenterology 17(5):297-299. [Chinese]

w236 Harford W et al. Double-blind, multicenter evaluation of lansoprazole and amoxicillin dual therapy for the cure of Helicobacter pylori infection. Helicobacter 1996;1(4):243-50.

w237 Hsu CC et al. One-week low-dose triple therapy without anti-acid treatment has sufficient efficacy on Helicobacter pylori eradication and ulcer healing. Hepato-Gastroenterol 2003;50(53):1731-4.

w238 D'Imperio N et al. Randomized study on the effectiveness of omeprazole in the treatment of operative endoscopy-induced oesophagogastric lesions. Argo Gastroenterol Clin 1994;7(2):71-6. [Italian]

w239 Jung JM et al. Role of anti-secretory treatment in addition to Helicobacter pylori eradication triple therapy in the treatment of peptic ulcer. Korean J Gastroenterol/Taehan Sohwagi Hakhoe Chi 2008;51(1):11-8. [Korean]

w240 Dotto P et al. Comparison of three different therapeutic regimens for eradicating Helicobacter pylori. Curr Ther Res Clin E 1993;53(5):557-64.

w241 Valenzuela JE et al. Comparison of once-daily doses of omeprazole (40 and 20 mg) and placebo in the treatment of benign gastric ulcer: A multicenter, randomized, double- blind study. Am J Gastroenterol 1996;91(12):2516-22.

w242 Sezer R et al. Effect of omeprazole plus ciprofloxacin on healing of duodenal ulcer and clearance of Helicobacter pylori. Med Bul Istanbul Med Fac 1995;28(2):10-14.

w243 Graham DY et al. Omeprazole versus placebo in duodenal ulcer healing. The United States experience. Digest Dis Sci 1990;35(1):66-72.

w244 Colin R et al. Duodenal ulcer healing with 1-week eradication triple therapy followed, or not, by anti-secretory treatment: a multicentre double-blind placebo-controlled trial. Aliment Pharm Therap 2002;16(6):1157-62.

w245 Marzio L et al. Triple therapy for 7 days vs. triple therapy for 7 days plus omeprazole for 21 days in treatment of active duodenal ulcer with Helicobacter pylori infection. A double blind placebo controlled trial. Digest Liver Dis 2003;35(1):20-3.

w246 Tepes B et al. Is a one-week course of triple anti-Helicobacter pylori therapy sufficient to control active duodenal ulcer? Aliment Pharm Therap 2001;15(7):1037-45.

w247 Labenz J et al. One-week low-dose triple therapy for Helicobacter pylori is sufficient for relief from symptoms and healing of duodenal ulcers. Aliment Pharmacol Ther 1997;11(1):89-93.

w248 Uygurer et al. Duodenal ulcer healing with one week therapy. Turkish Journal of Gastroenterology 1998;9(1):46-9. [Turkish]

w249 Zafar M et al. Gastroprotective evaluation of phyllanthus emblica in the patients with gastric ulcers. Value in Health 2012;15 (7):A635.

w250 Neil GA et al. Helicobacter pylori eradication as a surrogate marker for the reduction of duodenal ulcer recurrence. Aliment Pharm Therap 1998;12(7):619-33.

w251 Bago J et al. Effect of acid suppression therapy and eradication of Helicobacter pylori infection on duodenal ulcer healing and recurrence. Croat J Gastro Hep 1998;7(3-4):77-81.

w252 Takeuchi, T et al. Strategies for peptic ulcer healing after 1 week proton pump inhibitor-based triple Helicobacter pylori eradication therapy in Japanese patients: Differences of gastric ulcers and duodenal ulcers. J Clin Biochem Nutr 2012;51(3):189-195.

w253 Cloud ML et al. Rabeprazole in treatment of acid peptic diseases: results of three placebo-controlled dose-response clinical trials in duodenal ulcer, gastric ulcer, and gastroesophageal reflux disease (GERD). The Rabeprazole Study Group. Digest Dis Sci 1998;43(5):993-1000.

w254 Sung JJY et al. Intravenous esomeprazole for prevention of recurrent peptic ulcer bleeding: a randomized trial. Ann Intern Med 2009;150(7):455-64.

w255 Hawkey GM et al. Drug treatments in upper gastrointestinal bleeding: value of endoscopic findings as surrogate end points. Gut 2001;49(3):372-9.

w256 Khuroo MS et al. A comparison of omeprazole and placebo for bleeding peptic ulcer. New Engl J Med 1997;336(15):1054-8.

w257 Javid G et al. Omeprazole as adjuvant therapy to endoscopic combination injection sclerotherapy for treating bleeding peptic ulcer. Am J Med 2001;111(4):280-4.

w258 Sofia C et al. Endoscopic injection therapy vs. multipolar electrocoagulation vs. laser vs. injection + octreotide vs. injection + omeprazole in the treatment of bleeding peptic ulcers. A prospective randomized study. Hepato-Gastroenterol 2000;47(35):1332-6.

w259 Lau JYW et al. Effect of intravenous omeprazole on recurrent bleeding after endoscopic treatment of bleeding peptic ulcers. New Engl J Med 2000;343(5):310-6.

w260 Hasselgren G et al. Continuous intravenous infusion of omeprazole in elderly patients with peptic ulcer bleeding. Results of a placebo-controlled multicenter study. Scand J Gastroentero 1997;32(4):328-33.

w261 Lau JY et al. Omeprazole before endoscopy in patients with gastrointestinal bleeding. New Engl J Med 2007;356(16):1631-40.

w262 Schaffalitzky de Muckadell OBS et al. Effect of omeprazole on the outcome of endoscopically treated bleeding peptic ulcers. Randomized double-blind placebo-controlled multicentre study. Scand J Gastroentero 1997;32(4):320-7.

w263 Daneshmend TK et al. Omeprazole versus placebo for acute upper gastrointestinal bleeding: randomised double blind controlled trial. Brit Med J 1992;304(6820):143-7.

w264 Zargar SA et al. Pantoprazole infusion as adjuvant therapy to endoscopic treatment in patients with peptic ulcer bleeding: prospective randomized controlled trial. J Gastroen Hepatol 2006;21(4):716-21.

w265 Hung WK et al. Randomized trial comparing pantoprazole infusion, bolus and no treatment on gastric pH and recurrent bleeding in peptic ulcers. Aust NZ J Surg 2007;77(8):677-81.

w266 Reele SB et al. Gastrointestinal absorption of D-xylose, glucose, and vitamin B12 during chronic therapy with arbaprostil. Curr Ther Res Clin El 1982;32(2):336-46.

w267 Lanza FL et al. Effect of enprostil on the gastroduodenal mucosa of healthy volunteers. Aliment Pharmacol Ther 1990;4(6):601-13.

w268 Davis TM et al. Effect of enprostil on glucose and lipid metabolism in type 2 diabetes. Diabetic Med 1989;6(5):400-5.

w269 Santillan RM et al. Prevention of gastroduodenal injury induced by NSAIDs with low-dose misoprostol. P W Pharmacol Soc 1999;42:33-4.

w270 Donnelly MT et al. Low-dose misoprostol for the prevention of low-dose aspirin-induced gastroduodenal injury. Aliment Pharm Therap 2000;14(5):529-34.

w271 Delmas PD et al. Misoprostol in the prevention of gastric erosions caused by nonsteroidal anti-inflammatory agents. Rev Rhum 1994;61(2):115-20.

w272 Graham DY et al. Prevention of NSAID-induced gastric ulcer with misoprostol: multicentre, double-blind, placebo-controlled trial. Lancet 1988;2(8623):1277-80.

w273 Spaggiari L et al. Evaluation of the clinical efficacy of gastric cytoprotection with misoprostol in patients treated with NSAIDs after proctologic surgery. Clin Terap 1993;142(3):235-41. [Italian]

w274 Bocanegra TS et al. Diclofenac/misoprostol compared with diclofenac in the treatment of osteoarthritis of the knee or hip: A randomized, placebo controlled trial. J Rheumatol 1998;25(8):1602-11.

w275 Van Beusekom HJ et al. ECSTRA study. A double-blind, placebo-controlled study on the effects of adding misoprostol to a 'standard treatment' of rheumatoid arthritis. TGO-Tijdschr Ther Ge 1995;20(3):7-11. [Dutch]

w276 Munger M et al. Misoprostol effects on diclofenac-induced cardiorenal changes in salt-sensitive patients with hypertension: The MEDIC study. Pharmacotherpy 2008;28(7):834-42.

w277 Ahmed WU et al. Misoprostol in the treatment of NSAID-induced gastroduodenal lesions. Indian J Gastroenterol 1991;10(4):135-6.

w278 Goldin E et al. Efficacy of misoprostol and ranitidine in the prevention of duodenal ulcer relapse and its correlation with endogenous gastric prostanoid synthesis. Aliment Pharm Therap 1991;5(2):173-80.

w279 Bach GL et al. Therapy of non-steroidal antirheumatic drug-induced gastrointestinal lesions with misoprostol. Zeitschrift fur Rheumatologie 1991;50(3):175-80. [German]

w280 Curtis LD et al. Misoprostol in renal transplantation. Transplant P 1993;25(1:Pt 1):602.

w281 Raskin JB et al. Misoprostol dosage in the prevention of nonsteroidal anti-inflammatory drug-induced gastric and duodenal ulcers: a comparison of three regimens. Ann Intern Med 1995;123(5):344-50.

w282 Pazzi P et al. Misoprostol for the treatment of chronic erosive gastritis: A double-blind placebo-controlled trial. Am J Gastroenterol 1994;89(7):1007-13.

w283 De Melo Gomes JA et al. The safety of Arthrotec in patients with rheumatoid arthritis or osteoarthritis: an assessment of the upper gastrointestinal tract by endoscopy. Scand J Rheumatol 1992;96(Suppl.):23-31.

w284 Doherty M et al. The efficacy of Arthrotec in the treatment of osteoarthritis. Scand J Rheumatol 1992;21(96)(Suppl.):15-21.

w285 Agrawal NM et al. Misoprostol co-administered with diclofenac for prevention of gastroduodenal ulcers. A one-year study. Digest Dis Sci 1995;40(5):1125-31.

w286 Hannequin JR et al. Efficacy of Arthrotec in the treatment of rheumatoid arthritis. Scand J Rheumatol 1992;96(Suppl.):7-14.

w287 Akkuzu B et al. Efficacy of misoprostol in the treatment of tinnitus in patients with diabetes and/or hypertension. Auris Nasus Larynx 2004;31(3):226-32.

w288 Zuinen C et al. Diclofenac/misoprostol vs diclofenac/placebo in treating acute episodes of tendinitis/bursitis of the shoulder. Drugs 1993;45(Suppl.1):17-23.

w289 Chandrasekaran AN et al. Double blind, placebo controlled trial on the cytoprotective effect of misoprostol in subjects with rheumatoid arthritis, osteoarthritis and seronegative spondarthropathy on NSAIDs. J Assoc Phys of Ind 1991;39:919-21.

w290 Henriksson K et al. Helicobacter pylori infection, ABO blood group, and effect of misoprostol on gastroduodenal mucosa in NSAID-treated patients with rheumatoid arthritis. Digest Dis Sci 1993;38(9):1688-96.

w291 Spaziani E et al. Chronic gastritis and prostaglandins. Results of endoscopic screening. Minerva Gastroenterol Diet 1994;40(4):167-75. [Italian]

w292 Bardhan KD et al. The prevention and healing of acute non-steroidal anti-inflammatory drug-associated gastroduodenal mucosal damage by misoprostol. Brit J Rheumatol 1993;32(11):990-5.

w293 Boers M et al. No effect of misoprostol on renal function of rheumatoid patients treated with diclofenac. Brit J Rheumatol 1991;30(1):56-9.

w294 Briner W et al. Synthetic prostaglandin E1 misoprostol as a treatment for tinnitus. Arch Otolaryngol 1993;119(6):652-4.

References for trials listed in Webtables 1-7

w295 Fujimori S et al. Prevention of nonsteroidal anti-inflammatory drug-induced small-intestinal injury by prostaglandin: a pilot randomized controlled trial evaluated by capsule endoscopy. Gastrointest Endosc 2009;69(7):1339-46.

w296 Kelly SM et al. Increased gastric juice epidermal growth factor after non-steroidal anti-inflammatory drug ingestion. Gut 1994;35(5):611-4.

w297 Evers S et al. Prostaglandin analog mechanisms are not effective in refractory chronic cluster headache. Headache 1998;38(8):618-20.

w298 Levi S et al. Effects of nonsteroidal anti-inflammatory drugs and misoprostol on gastroduodenal epithelial proliferation in arthritis. Gastroenterology 1992;102(5):1605-11.

w299 Elliott SL et al. Efficacy of 12 months misoprostol as prophylaxis against NSAID-induced gastric ulcers. Scand J Rheumatol 1994;23(4):171-6.

w300 Duenas-Gonzalez A et al. Misoprostol prophylaxis for high-dose chemotherapy-induced mucositis: a randomized double-blind study. Bone Marrow Transpl 1995;17(5):809-12.

w301 Roth SH et al. A controlled study comparing the effects of nabumetone, ibuprofen, and ibuprofen plus misoprostol on the upper gastrointestinal tract mucosa. Arch Intern Med 1993;153(22):2565-71.

w302 Graham DY et al. Duodenal and gastric ulcer prevention with misoprostol in arthritis patients taking NSAIDs. Ann Intern Med 1993;119(4):257-62.

w303 Silverstein FE et al. Misoprostol reduces serious gastrointestinal complications in patients with rheumatoid arthritis receiving nonsteroidal anti-inflammatory drugs. A randomized, double-blind, placebo-controlled trial. Ann Intern Med 1995;123(4):241-9.

w304 Shiokawa Y et al. Evaluation of misoprostol's clinical utility for gastric/duodenal ulcers seen under long-term use of non-steroidal anti-inflammatory drugs (NSAID)-I. Evaluation of mucosal prophylactic effects by a placebo-controlled double blind comparative study. Ryumachi 1991;31(5):554-71. [Japanese]

w305 Saggioro A et al. Misoprostol prevents NSAID-induced gastroduodenal lesions in patients with osteoarthritis and rheumatoid arthritis. Ital J Gastroenterol 1991;23:119-23.

w306 Fullerton T et al. Evaluation of the renal protective effect of misoprostol in elderly, osteoarthritic patients at risk for nonsteroidal anti-inflammatory drug-induced renal dysfunction. J Clin Pharmacol 1993;33(12):1225-32.

w307 Bolten W et al. Treatment of NSAID-induced gastrointestinal complaints by comedication with the prostagladin analogue misoprostol in rheumatoid arthritis patients. A multicentred double-blind placebo controlled study. Aktuel Rheumatol 1989;14(5):214-20. [German]

w308 Bennett PN et al. Misoprostol does not alter the pharmacokinetics of propranolol. Postgrad Med J 1991;67(787):455-7.

w309 Hilbrands LB et al. Randomized trial of misoprostol in patients with chronic renal transplant rejection. Transplant Int 1996;9(Suppl.1):41-4.

w310 Goodwin JS et al. Effect of misoprostol on immune function in elderly subjects. Prostaglandins 1987;33(Suppl.):7.

w311 Moran M et al. Prevention of acute graft rejection by the prostaglandin E1 analogue misoprostol in renal-transplant recipients treated with cyclosporine and prednisone. New Engl J Med 1990;322(17):1183-8.

w312 Weinblatt ME et al. Lack of a renal-protective effect of misoprostol in rheumatoid arthritis patients receiving cyclosporin A. Results of a randomized, placebo-controlled trial. Arthrit Rheum 1994;37(9):1321-5.

w313 Mavligit GM et al. Gastroduodenal mucosal injury during hepatic arterial infusion of chemotherapeutic agents. Lack of cytoprotection by prostaglandin E1 analogue. Gastroenterology 1987;92(3):566-9.

w314 Kim HJ et al. Effects of misoprostol on renal function and plasma endothelin-1 levels during early postoperative stage of living donor renal transplant recipients. Transplant P 1996;28(3):1207-9.

w315 Hausken T et al. Misoprostol treatment exacerbates abdominal discomfort in patients with non-ulcer dyspepsia and erosive prepyloric changes. A double-blind, placebo-controlled, multicentre study. Scand J Gastroenterol 1990;25(10):1028-33.

w316 Yilmaz I et al. Misoprostol in the treatment of tinnitus: a double-blind study. Otolaryngology - Head & Neck Surgery 2004;130(5):604-10.

w317 Wasiak W et al. A six week double blind, placebo controlled, crossover study of the effect of misoprostol in the treatment of aspirin sensitive asthma. Thorax 1999;54(10):900-4.

w318 Nishida U et al. Evaluation of small bowel blood flow in healthy subjects receiving low-dose aspirin. World J Gastroenterology 2011;17(2):226-30.

w319 Han X et al. Effect of Rebamipide on the Premalignant Progression of Chronic Gastritis: A Randomized Controlled Study. Clin Drug Invest. 2015;35(10):665-73.

w320 Mizukami K et al. Efficacy of rebamipide for low-dose aspirin-related gastrointestinal symptoms. Journal of Clin Biochem and Nutri 2012;51(3):216-20.

w321 Tozawa K et al. A randomized, double-blind, placebo-controlled study of rebamipide for gastric mucosal injury taking aspirin with or without clopidogrel. Digest Dis Sci 2014;59(8):1885-90.

w322 Fujimori S et al. Rebamipide has the potential to reduce the intensity of NSAID-induced small intestinal injury: a double-blind, randomized, controlled trial evaluated by capsule endoscopy. J Gastroenterology 2011;46(1):57-64.

w323 Kato M et al. Rebamipide prevents low-dose aspirin-induced small intestinal injuries: A randomized placebo-controlled double-blinded cross-over study using video capsule endoscopy and contrast-enhanced ultrasonography. Gastroenterology 2011;(1):S60 (271).

w324 Kamada T et al. Rebamipide improves chronic inflammation in the lesser curvature of the corpus after Helicobacter pylori eradication: A multicenter study. Biomed Res Int 2015: 865146.

w325 Kurokawa S et al. 2014 A randomized, double-blinded, placebo-controlled, multicenter trial, healing effect of rebamipide in patients with low-dose aspirin and/or non-steroidal anti-inflammatory drug induced small bowel injury. J Gastroenterology 49(2):239-44.

w326 Horiki N et al. Prevention of celecoxib-induced gastrointestinal mucosal injury by concomitant therapy with gastro-protective drug rebamipide. Digest Dis Sci 2012;57(11):2732.

w327 Adachi K et al. A study on the efficacy of rebamipide for patients with proton pump inhibitor-refractory non-erosive reflux disease. Digest Dis Sci 2012;57(6):1609-17.

w328 Abou-Raya A et al. Efficacy of rebamipide in prevention of COX-2 inhibition induced gastrointestinal injuries (gastropathy) in older adults with knee osteoarthritis: A randomized placebo-controlled study. Ann Rheum Dis Conf: Ann Europe Cong Rheumatology Europe League Against Rheumatism, EULAR. 2012;71.

w329 Watanabe T et al. A multicenter, randomized, double-blind, placebo-controlled trial of high-dose rebamipide treatment for low-dose aspirin-induced moderate-to-severe small intestinal damage. PLoS ONE 2015;10(4):(e0122330).

w330 Paladini G et al. Prophylactic and therapeutic role of rioprostil in NSAIDs induced gastroduodenal lesions. Scand J Gastroentero 1989;24(164)(Suppl.):242-6.

w331 Purushotham AD et al. Prostaglandins in the treatment of reflux oesophagitis: double-blind placebo controlled clinical trial. J Roy Coll Surg Edin 1991;36(4):216-8.

w332 Karim SM et al. Effects of some naturally occurring prostaglandins and synthetic analogues on gastric secretion and ulcer healing in man. Adv Prostag Thromb Res 1976;2:529-39.

w333 Gibinski K et al. Double-blind clinical trial on gastroduodenal ulcer healing with prostaglandin E2 analogues. Gut 1977;18(8):636-9.

w334 Jaszewski R et al. Failure of a cytoprotective dose of Arbacet to heal acute gastric ulcers. Am J Gastroenterol 1988;83(7):734-6.

w335 Euler AR et al. A multiclinic trial evaluating arbaprostil [15(R)-15-methyl prostaglandin E2] as a therapeutic agent for gastric ulcer. Gastroenterology 1989;96(4):967-71.

w336 Euler AR et al. Failure of a cytoprotective dose of arbaprostil to heal acute duodenal ulcers. Results of a multiclinic trial. Gastroenterology 1987;92(3):604-7.

w337 Euler AR et al. A report of three multiclinic trials evaluating arbaprostil in arthritic patients with ASA/NSAID gastric mucosal damage. Gastroenterology 1990;98(6):1549-57.

w338 Jaszewski R et al. The effect of 15(R)-15-methyl prostaglandin E2 (Arbacet) on the healing of aspirin or nonsteroidal anti-inflammatory drug-induced gastric mucosal lesions: an endoscopic study. Am J Gastroenterol 1987;82(12):1271-4.

w339 Euler AR et al. Arbaprostil [15(R)-15-methyl prostaglandin E2] in a single night-time dose of either 50 or 100 micrograms in acute duodenal ulcer. Gastroenterology 1989;97(1):98-103.

w340 Euler AR et al. An evaluation of arbaprostil at multiple doses for the treatment of acute duodenal ulcer: a randomized double-blind placebo-controlled international trial. Am J Gastroenterol 1990;85(2):145-9.

w341 Chen FW et al. Effect of prostaglandin E2 and 15(R)15 methyl PGE2 on duodenal ulcer. Singap Med J 1980;21(3):596-8.

w342 Vantrappen G et al. Effect of 15(R)-15-methyl prostaglandin E2 (arbaprostil) on the healing of duodenal ulcer. A double-blind multicenter study. Gastroenterology 1982;83(2):357-63.

w343 Dal Monte PR et al. Evaluation of the efficacy and tolerability of enprostil, a synthetic prostaglandin E2 analog, in a clinical double-blind, placebo cotrolled trial. Clin Terapeut 1988;125(5):337-43. [Italian]

w344 Schubert TT et al. A US multicenter study of enprostil 35 micrograms twice daily for treatment of prepyloric, pyloric channel, and duodenal bulb ulcers. Enprostil Study Group. Digest Dis Sci 1989;34(9):1355-60.

w345 Bright-Asare P et al. Treatment of duodenal ulcer with enprostil, a prostaglandin E2 analogue. Am J Med 1986;81(2A):64-8.

w346 Navert H et al. Treatment of gastric ulcer with enprostil. Am J Med 1986;81(2A):75-9.

w347 Thomson AB et al. Treatment of duodenal ulcer with enprostil, a synthetic prostaglandin E2 analogue. Am J Med 1986;81(2A):59-63.

w348 Sontag SJ et al. Healing of NSAID-induced gastric ulcers with a synthetic prostaglandin analog (enprostil). Am J Gastroenterol 1994;89(7):1014-20.

w349 Agrawal NM et al. Healing of benign gastric ulcer: A placebo-controlled comparison of two dosage regimens of misoprostol, a synthetic analog of prostaglandin E2 analogues. Digest Dis Sci 1985;30(Suppl.11):164-70.

w350 Lam S-K et al. Prostaglandin E2 (misoprostol) overcomes the adverse effect of chronic cigarette smoking on duodenal ulcer healing. Digest Dis Sci 1986;31(Suppl.2):68-74.

w351 Brand DL et al. Misoprostol, a synthetic PGE1 analog, in the treatment of duodenal ulcers: A multicenter double-blind study. Digest Dis Sci 1985;30(Suppl.11):147-58.

w352 Bright-Asare P et al. Efficacy of misoprostol (twice daily dosage) in acute healing of duodenal ulcer. A multicenter double-blind controlled trial. Digest Dis Sci 1986;31(Suppl.2):63-7.

w353 Sontag SJ et al. Misoprostol in the treatment of duodenal ulcer: A multicenter double-blind placebo-controlled study. Digest Dis Sci 1985;30(Suppl.):159-63.

w354 Mazure PA et al. Misoprostol in the treatment of active duodenal ulcer: A double-blind trial. Acta Gastroenterologica Latinoamericana 1985;15(1):13-24. [Spanish]

w355 Ahmed WU et al. A double-blind study of misoprostol (SC-29333) in the healing of duodenal ulcer. J Gastroen Hepatol 1991;6(2):179-80.

w356 Jaszewski R et al. Treatment of nonsteroidal anti-inflammatory drug-induced gastric ulcers with misoprostol. A double-blind multicenter study. Digest Dis Sci 1992;37(12):1820-4.

References for trials listed in Webtables 1-7

w357 Newman RD et al. Misoprostol in the treatment of duodenal ulcer refractory to H2-blocker therapy. A placebo-controlled, multicenter, double-blind, randomized trial. Am J Med 1987;83(1A):27-31.

w358 Roth S et al. Misoprostol heals gastroduodenal injury in patients with rheumatoid arthritis receiving aspirin. Arch Intern Med 1989;149(4):775-9.

w359 Barakat MH et al. Cigarette smoking and duodenal ulcer healing. An endoscopic study of 197 patients. Digestion 1984;29(2):85-90.

w360 Terano A et al. Rebamipide, a gastro-protective and anti-inflammatory drug, promotes gastric ulcer healing following eradication therapy for H pylori in a Japanese population: randomized, double-blind, placebo-controlled trial. J Gastroenterol 2007;42:690-93.

w361 Hahm KB et al. Augmented eradication rates of Helicobacter pylori by new combination therapy with lansoprazole, amoxicillin, and rebamipide. Digest Dis Sci 1998;43(2):235-40.

w362 Fujioka T et al. Effects of rebamipide, a gastro-protective drug on the Helicobacter pylori status and inflammation in the gastric mucosa of patients with gastric ulcer: a randomized double-blind placebo-controlled multicentre trial. Aliment Pharmacol Ther 2003;18(Suppl.1):146-52.

w363 Sawaguchi M et al. Role of mucosal-protective agents, rebamipide and polaprezinc, in post-gastric endoscopic submucosal dissection ulcer healing A randomized controlled study. Gastrointestinal Endoscopy 2011;(1):AB424.

w364 Kato T et al. Clinical trial: rebamipide promotes gastric ulcer healing by proton pump inhibitor after endoscopic submucosal dissection--a randomized controlled study. J Gastroenterology 2010;45(3):285-90.

w365 Fujiwara S et al. (2011). A randomized controlled trial of rebamipide plus rabeprazole for the healing of artificial ulcers after endoscopic submucosal dissection. J Gastroenterology 46(5):595-602.

w366 Nebiki H et al. Effect of rebamipide on Helicobacter pylori infection in patients with peptic ulcer. Digest Dis Sci 1998;43(Suppl.9):203-6.

w367 Higuchi K et al. Rebamipide prevents recurrence of gastric ulcers without affecting Helicobacter pylori status. Digest Dis Sci 1998;43(Suppl.9):99-106.

w368 Caldwell JR et al. Endoscopic evaluation of Rioprostil in the management of non-steroidal anti-inflammatory drug-induced gastritis: an interim analysis. Scand J Gastroentero 1989;24(Suppl.164):239-41.

w369 Boucekkine T et al. Treatment of duodenal ulcer with rioprostil: a randomized multicentre double-blind study. Scand J Gastroentero 1989;24(Suppl.164):191-3.

w370 Benvestito V et al. Therapeutic use of rioprostil, 600 micrograms o.d., in duodenal peptic ulcer during its active uncomplicated phase. Minerva Diet Gastroentero 1988;34(3):191-5. [Italian]

w371 Yilmaz E et al. Cimetidine therapy for warts: a placebo-controlled, double-blind study. J Am Acad Dermatol 1996;34(6):1005-7.

w372 Bergstrand H et al. Effects of long-term treatment with low dose cimetidine on allergen-induced airway responses and selected immunological parameters in atopic asthmatics. Allergy 1985;40(3):187-97.

w373 Hui WM et al. Maintenance therapy for duodenal ulcer: a randomized controlled comparison of seven forms of treatment. Am J Med 1992;92(3):265-74.

w374 Furman D et al. A double-blind trial comparing high dose liquid antacid to placebo and cimetidine in improving symptoms and objective parameters in gastroesophageal reflux. Gastroenterology 1982;82(5 II):1062.

w375 Kibblewhite DJ et al. A double-blind controlled study of the efficacy of cimetidine in the treatment of the cervical symptoms of gastroesophageal reflux. J Otolarngol 1990;19(2):103-9.

w376 Welch RW et al. Reduction of aspirin-induced gastrointestinal bleeding with cimetidine. Gastroenterology 1978;74(2):459-63.

w377 Roth SH et al. Cimetidine therapy in nonsteroidal anti-inflammatory drug gastropathy. Double-blind long-term evaluation. Arch Intern Med 1987;147(10):1798-801.

w378 Wu S-X et al. Different dosages of azelastine and in combination of cimetidine in the treatment of chronic urticaria. J Clin Derm 2008;37(3):194-6. [Chinese]

w379 Sonnenberg A et al. Prevention of duodenal ulcer recurrence with cimetidine (author's transl). Deut Med Wochenschr 1979;104(20):725-30. [German]

w380 Kokron O et al. Randomised study in metastatic renal cell cancer: Coumarin versus coumarin + cimetidine. J Irish Coll Phys S 1993;22(Suppl.1):10-1.

w381 Lööf L et al. Psychological group counseling for the prevention of ulcer relapses. A controlled randomized trial in duodenal and prepyloric ulcer disease. J Clin Gastroenterol 1987;9(4):400-7.

w382 Becker U et al. Relapse prevention of duodenal ulcers with trimipramine, cimetidine, or placebo. A double-blind comparison. Scand J Gastroentero 1984;19(3):405-10.

w383 Sontag S et al. Cimetidine, cigarette smoking, and recurrence of duodenal ulcer. New Engl J Med 1984;311(11):689-93.

w384 Kinloch JD et al. The effect of cimetidine on the maintenance of healing of gastric ulceration. Postgrad Med J 1984;60(708):665-7.

w385 Visconti GP et al. Long-term trial of pirenzepine, cimetidine and placebo in the management of duodenal ulcer. Curr Ther Res Clin E 1983;34(5):853-6.

w386 Moshal MG et al. Pirenzepine, cimetidine and placebo in the long-term treatment of duodenal ulceration. A comparative study. S Afr Med J 1982;62(1):12-4.

w387 Bardhan KD et al. Antacid maintenance therapy in the prevention of duodenal ulcer relapse. Gut 1988;29(12):1748-54.

w388 Eichenberger PM et al. Treatment and relapse prophylaxis of duodenal ulcer with pirenzepine and cimetidine. Scand J Gastroenterol 1982;17(72)(Suppl.):197-206.

w389 Ubilluz R. Prevention of duodenal ulcer recurrence. A double-blind study of cimetidine (Tagamet). Clin Trials J 1982;19(3):150-61.

w390 Sontag S et al. Three cimetidine dose schedules versus placebo in preventing duodenal ulcer recurrences. A multicenter double blind study. Gastroenterology 1981;80(5 II):1290.

w391 Da Silva EP et al. Long-term treatment of gastric ulcer with cimetidine. Clin Ther 1981;4(1):24-31.

w392 Salera M et al. Long-term effects and after-effects of cimetidine in duodenal ulcer patients. Ital J Gastroenterol 1979;11(2):49-52.

w393 Cocco AE et al. A multi-center study of the recurrence of duodenal ulcer on long-term treatment with cimetidine. Gastroenterology 1981;80(5 II):1125.

w394 Bianchi Porro G et al. Long-term prophylactic treatment with cimetidine in duodenal ulceration. Ital J Gastroenterol 1979;11(4):181-3.

w395 Massarrat S et al. Long term prophylaxis of duodenal ulcer with cimetidine. Deut Med Wochenschr 1982;107(28):1085-8. [German]

w396 Dew MJ et al. Maintenance therapy for duodenal ulcer-a trial comparing cimetidine with a prostaglandin synthetase promoter. J Clin Hosp Pharm 1984;9(4):357-9.

w397 Fitzpatrick WJ et al. Bedtime cimetidine maintenance treatment: optimum dose and effect on subsequent natural history of duodenal ulcer. Gut 1982;23(3):239-42.

w398 Berstad A et al. Maintenance treatment of duodenal ulcer patients with a single bedtime dose of cimetidine. Scand J Gastroentero 1979;14(7):827-31.

w399 Broor SL et al. Duodenal ulcer: maintenance treatment with cimetidine. Indian J Gastroen 1983;2(2):81-2.

w400 Hentschel E et al. Effect of cimetidine treatment in the prevention of gastric ulcer relapse: a one year double blind multicentre study. Gut 1983;24(9):853-6.

w401 Piper DW et al. The effect of maintenance cimetidine therapy on the medical, social and economic aspects of patients with chronic gastric ulcers. A placebo-controlled prospective study. Med J Australia 1986;145(8):400-3.

w402 Pym B et al. Cost-effectiveness of cimetidine maintenance therapy in chronic gastric and duodenal ulcer. Gastroenterology 1990;99(1):27-35.

w403 Sonnenberg A et al. Predictors of duodenal ulcer healing and relapse. Gastroenterology 1981;81(6):1061-7.

w404 Marks IN et al. A maintenance regimen of sucralfate 2g at night for reduced relapse rate in duodenal ulcer disease. A one-year follow-up study. Am J Med 1989;86(6A):136-40.

w405 Murray FE et al. Management of reflux oesophagitis: role of weight loss and cimetidine. Irish J Med Sci 1991;160(1):2-4.

w406 Kaul B et al. Gastroesophageal reflux disease. Acute and maintenance treatments with cimetidine. Scand J Gastroentero 1986;21(2):139-45.

w407 Kumar N et al. Long term behaviour of healed duodenal ulcer with and without maintenance therapy. Journal of the Association of Physicians of India 1990;38(Suppl.1):707-11.

w408 Salim AS et al. Sulphydryl-containing agents and the prevention of duodenal ulcer relapse. Pharmacology 1993;46(5):281-8.

w409 Salim AS et al. The relationship between Helicobacter pylori and oxygen-derived free radicals in the mechanism of duodenal ulceration. Internal Med 1993;32(5):359-64.

w410 Young MD et al. Efficacy of once-daily cimetidine in preventing recurrence of duodenal ulcer. Clin Ther 1989;11(4):529-38.

w411 Takemoto T et al. Efficacy of sucralfate in the prevention of recurrence of peptic ulcer-double blind multicenter study with cimetidine. Scand J Gastroentero 1987;140(Suppl.):49-60.

w412 Rohner HG et al. Long term cimetidine therapy for the prevention of recurring peptic ulcer: a multicenter study. Zeitschrift fur Gastroenterologie 1985;23(8):403-11. [German]

w413 Burland WL et al. Cimetidine treatment for the prevention of recurrence of duodenal ulcer: an international collaborative study. Postgrad Med J 1980;56(653):173-6.

w414 Gray GR et al. Long-term cimetidine in the management of severe duodenal ulcer dyspepsia. Gastroenterology 1978;74(2):397-401.

w415 Salim AS. Oxygen-derived free radicals and the prevention of duodenal ulcer relapse: A new approach. Am J Med Sciences 1990;300(1):1-6.

w416 Jean F et al. Maintenance therapy of ulcer disease. Comparative multicenter study of sucralfate, cimetidine and a placebo. Rev Med Interne 1985;6(3):321-6. [French]

w417 Leao GC et al. Cimetidine in the treatment of gastric ulcer. Arq Gastroenterol 1980;17(2):63-8. [Portuguese]

w418 Aubia J et al. Dialysis pruritus: Effect of cimetidine. J Dialysis 1980;4(4):141-5.

w419 Lai KN et al. Effect of long-term cimetidine treatment on left ventricular function in haemodialysis patients with active hyperparathyroidism. Brit J Clin Pharmaco 1982;13(5):693-7.

w420 Paoluzi P et al. Incompletely and completely healed duodenal ulcers' outcome in maintenance treatment: a double blind controlled study. Gut 1985;26(10):1080-5.

w421 Libeskind M et al. Maintenance treatment of patients with healed peptic ulcer with sucralfate, placebo and cimetidine. Scand J Gastroentero 1983;83(Suppl.):69-70.

w422 Stoa-Birketvedt G et al. Effect of cimetidine suspension on appetite and weight in overweight subjects. Brit Med J 1993;306(6885):1091-3.

w423 Rasmussen MH et al. Cimetidine suspension as adjuvant to energy restricted diet in treating obesity. Brit Med J 1993;306(6885):1093-6.

References for trials listed in Webtables 1-7

w424 Langman MJ et al. Prospective, double-blind, placebo-controlled randomized trial of cimetidine in gastric cancer. British Stomach Cancer Group. Brit J Cancer 1999;81(8):1356-62.

w425 Huang W-H et al. Cimetidine as adjuvant treatment in rectal cancer. Chinese J Cancer Prevent & Treat 2007;14(8):623-4. [Chinese]

w426 Macphee GJ et al. Effects of cimetidine on carbamazepine auto- and hetero-induction in man. Brit J Clin Pharmaco 1984;18(3):411-9.

w427 Thilagarajah R et al. Oral cimetidine gives effective symptom relief in painful bladder disease: A prospective, randomized, double-blind placebo-controlled trial. BJU Int 2001;87(3);207-212.

w428 La Brooy SJ et al. Cimetidine in the maintenance treatment of gastric ulceration (GU). Hepato-Gastroenterol 1980;27(Suppl.):E26.

w429 Farup PG et al. Low-dose antacids versus 400 mg cimetidine twice daily for reflux oesophagitis. A comparative, placebo-controlled, multicentre study. Scand J Gastroentero 1990;25(3):315-20.

w430 Danielsson A et al. Effect of fifteen months of double-blind treatment with cimetidine and placebo on peptic ulcer recurrence. Eur J Clin Pharmacol 1981;19(5):335-41.

w431 Frank WO et al. Reduction of indomethacin induced gastroduodenal mucosal injury and gastrointestinal symptoms with cimetidine in normal subjects. J Rheumatol 1989;16(9):1249-52.

w432 Barr GD et al. A two-year prospective controlled study of maintenance cimetidine and gastric ulcer. Gastroenterology 1983;85(1):100-4.

w433 Korman MG et al. Relapse of duodenal ulcer after cessation of long-term cimetidine treatment. A double-blind controlled study. Digest Dis Sci 1980;25(2):88-91.

w434 Jensen KG et al. Long-term treatment of peptic ulcer with cimetidine (Tagamet). A controlled trial. Uges Laeger 1980;142(51):3367-9. [Danish]

w435 Kang JY et al. The use of long term cimetidine in the prevention of gastric ulcer relapse: Double blind trial. Aust NZ J Med 1980;10(1):111.

w436 Nyren O et al. Absence of therapeutic benefit from antacids or cimetidine in non-ulcer dyspepsia. New Engl J Med 1986;314(6):339-43.

w437 Bardhan KD et al. Double-blind comparison of cimetidine and placebo in the maintenance of healing of chronic duodenal ulceration. Gut 1979;20(2):158-62.

w438 Dronfield MW et al. Controlled trial of maintenance cimetidine treatment in healed duodenal ulcer: short and long-term effects. Gut 1979;20(6):526-30.

w439 Bijlsma JW et al. Treatment of endoscopy-negative NSAID-induced upper gastrointestinal symptoms with cimetidine: an international multicentre collaborative study. Aliment Pharm Therap 1988;2(Suppl.):83.

w440 Anderson J et al. Effect of H1- and H2-receptor antagonists on sputum volume and lung function in patients with generalized bronchiectasis. Brit J Dis Chest 1985;79(3):272-4.

w441 Blackwood WS et al. Prevention by bedtime cimetidine of duodenal-ulcer relapse. Lancet 1978;311(8065):626-7.

w442 Bodemar G et al. Short- and long-term treatment with cimetidine in peptic ulcer disease and the pharmacokinetics of cimetidine. Scand J Gastroentero 1979;55(Suppl.):96-106.

w443 Hetzel DJ et al. Cimetidine treatment of duodenal ulceration: short term clinical trial and maintenance study. Gastroenterology 1978;74:389-92.

w444 Gudmand-Hoyer E et al. Prophylactic effect of cimetidine in duodenal ulcer disease. Brit Med J 1978;1(6120):1095-7.

w445 Birger Jensen K et al. Prophylactic effect of cimetidine in gastric ulcer patients. Scand J Gastroentero 1979;14(2):175-6

w446 Colle M et al. Plasma prolactin, sex steroids and gastrin in human volunteers treated for 2 weeks with therapeutic doses of cimetidine or the new histamine H2-receptor antagonist ramixotidine (CM 57755A). Eur J Clin Pharmacol 1988;35(5):529-34.

w447 Matsumoto S. The effect of cimetidine as BRM - Adjuvant immuno-chemotherapy for colorectal carcinoma. [Japanese]. Biotherapy 1996;10(3):511-5.

w448 Johnsson F et al. Cimetidine improves GERD symptoms in patients selected by a validated GERD questionnaire. Aliment Pharm Therap 1993;7(1):81-6.

w449 Hetzel DJ et al. Prevention of duodenal ulcer relapse by cimetidine: a one-year double-blind trial. Med J Australia 1979;1(11):529-31.

w450 Hansky J et al. Long-term cimetidine in duodenal ulcer disease. Digest Dis Sci 1979;24(6):465-7.

w451 Mekel RC et al. Long-term treatment with cimetidine. S Afr Med J 1978;26:1089-91.

w452 Nesland AA et al. Effect of cimetidine in patients with non-ulcer dyspepsia and erosive prepyloric changes. Scand J Gastroentero 1985;20(5):629-35.

w453 Svendsen LB et al. Cimetidine as an adjuvant treatment in colorectal cancer. A double-blind, randomized pilot study. Dis Colon Rectum 1995;38(5):514-8.

w454 Talley NJ et al. Randomized, double-blind, placebo-controlled crossover trial of cimetidine and pirenzepine in nonulcer dyspepsia. Gastroenterology 1986;91(1):149-56.

w455 Singal AK et al. Cimetidine in the treatment of non-ulcer dyspepsia: results of a randomized double-blind, placebo-controlled study. Curr Med Res Opin 1989;11(6):390-7.

w456 Riemann JF et al. Cimetidine suspension in patients with stage 0 gastro-oesophageal reflux disease. Aliment Pharm Therap 1991;5(2):191-7.

w457 Rugstad HE et al. Effect of cimetidine on gastrointestinal symptoms in patients taking nonsteroidal anti-inflammatory drugs. Scand J Rheumatol 1994;23(4):177-82.

w458 Gotthard R et al. Treatment with cimetidine, antacid, or placebo in patients with dyspepsia of unknown origin. Scand J Gastroentero 1988;23(1):7-18.

w459 Lee SH et al. Plantar warts of defined aetiology in adults and unresponsiveness to low dose cimetidine. Australasian Journal of Dermatology. 2001;42(3):220-1.

w460 Tonnesen H et al. Effect of cimetidine on survival after gastric cancer. Lancet 1988;2(8618):990-2.

w461 Carpenter GB et al. Evaluation of combined H1- and H2-receptor blocking agents in the treatment of seasonal allergic rhinitis. J Allergy Clin Immun 1983;71(4):412-7.

w462 Mackinnon M et al. Cimetidine in the management of symptomatic patients and duodenitis. A double-blind controlled trial. Digest Dis Sci 1982;27(3):217-9.

w463 Niv Y et al. Treatment of duodenitis with cimetidine: A clinical, endoscopic, and histologic study. J Clin Gastroenterol 1990;12(6):628-32.

w464 Rune SJ et al. Recurrence of duodenal ulcer pain after treatment with cimetidine for four and eight weeks. Gut 1980;21(2):151-3.

w465 Bennett JR et al. Cimetidine in reflux oesophagitis. Hepato-Gastroenterol 1980;27(Suppl.):E5.

w466 Willing RL et al. Cimetidine and duodenitis - a placebo controlled double blinded study of the effect of cimetidine on the endoscopic and histological appearances of duodenitic mucosa. Gastroenterology 1980;78(5 II):1291.

w467 Camarri E et al. Double-blind placebo controlled cross-over study on cimetidine-prophylactic effect in patients under steroid treatment. Preliminary data. Int J Clin Pharm Th Tox 1980;18(6):258-60.

w468 Breen KJ et al. Treatment of reflux oesophagitis. A randomized, controlled evaluation of cimetidine. Med J Australia 1983;2(11):555-8.

w469 Hamid J et al. The effect of cimetidine on immunological parameters in Crohn's disease: a double blind trial. Immunol Lett 1983;6(6):293-8.

w470 Kelbaek H et al. Controlled clinical trial of treatment with cimetidine for non-ulcer dyspepsia. Acta Medica Scandinavica 1985;217(3):281-7.

w471 Machell RJ et al. Cimetidine in the prevention of gastric ulcer relapse. Postgrad Med J 1979;55(644):393-5.

w472 Brown P et al. Cimetidine in the treatment of reflux oesophagitis. Med J Australia 1979;2(2):96-7.

w473 Bardhan KD et al. Does treatment with cimetidine extended beyond initial healing of duodenal ulcer reduce the subsequent relapse rate? Brit Med J 1982;284(6316):621-3.

w474 Popoola AO et al. Clinical response and gastroduodenal mucosa morphology in cimetidine treated Nigerian patients duodenal ulcer and antroduodenitis. Nigerian Med J 1978;8(3):175-81.

w475 Sillero C et al. Controlled trial of cimetidine in acute pancreatitis. Eur J Clin Pharmacol 1981;21(1):17-21.

w476 Perez De OC et al. Controlled treatment of acute pancreatitis. Double-blind trial with cimetidine. Revista Clin Esp 1980;158(6):263-6. [Spanish]

w477 Stoa-Birketvedt G et al. Cimetidine reduces weight and improves metabolic control in overweight patients with Type 2 diabetes. Int J Obesity 1998;22(11):1041-5.

w478 Bendayan R et al. Lack of effect of cimetidine on cigarette smoking. Eur J Clin Pharmacol 1993;44(1):51-5.

w479 Bright-Asare P et al. Cimetidine, metoclopramide, or placebo in the treatment of symptomatic gastroesophageal reflux. J Clin Gastroenterol 1980;2(2):149-56.

w480 Layne E et al. Does cimetidine protect against GI bleeding in neurological/neurosurgical patients with steroids? Gastroenterology 1981;80(5 II):1205.

w481 Cohen CJ et al. Lack of effect of cimetidine on lymphocyte subsets in patients infected with human immunodeficiency virus type 1. Clin Infect Dis 1996;23(5):1049-54.

w482 Ferguson R et al. Cimetidine in treatment of reflux oesophagitis with peptic stricture. Brit Med J 1979;2(6188):472-4.

w483 Monroe EW et al. Combined H1 and H2 antihistamine therapy in chronic urticaria. Arch Dermatol 1981;117(7):404-7.

w484 Behar J et al. Cimetidine in the treatment of symptomatic gastroesophageal reflux: a double blind controlled trial. Gastroenterology 1978;74(2:Pt 2):441-8.

w485 Greenblatt DJ et al. Clinical importance of the interaction of diazepam and cimetidine. New Engl J Med 1984;310(25):1639-43.

w486 Hoogwerf BJ et al. Effect of cimetidine on plasma lipids and lipoproteins. Clin Pharmacol Ther 1984;36(2):217-20.

w487 LI Y et al. Effects of perioperative cimetidine administration on natural killer cells in patients with gastrointestinal cancer. Chinese J Cancer Res 1999;11(1):70-3.

w488 Larrain A et al. Medical and surgical treatment of nonallergic asthma associated with gastroesophageal reflux. Chest 1991;99(6):1330-5.

w489 Diller G and Orfanos CE. Management of idiopathic urticaria with H1 + H2 antagonists. A crossover double blind long-term study. Zeitschrift fur Hautkrankheiten 1983;58(11):785-93. [German]

w490 Shanehsaz S. M et al. 2015 A comparative study between the efficacy of oral cimetidine and low-dose systemic meglumine antimoniate (MA) with a standard dose of systemic MA in the treatment of cutaneous leishmaniasis. International Journal of Dermatology 54(7):834-838.

w491 Lissak A et al. Treatment of hirsutism with cimetidine: a prospective randomized controlled trial. Fertil Steril 1989;51(2):247-50.

w492 Lepsien G et al. Treatment of reflux oesophagitis with cimetidine (author's transl). Deut Med Wochenschr 1979;104(25):901-6. [German]

w493 Wallach D et al. Cimetidine versus placebo in the treatment of psoriasis. Dermatologica 1982;165(3):197-203.

w494 Fiasse R et al. Controlled trial of cimetidine in reflux esophagitis. Digest Dis Sci 1980;25(10):750-5.

References for trials listed in Webtables 1-7

w495 Festen HP et al. Cimetidine in the treatment of severe ulcerative reflux oesophagitis; results of an 8-week double-blind study and of subsequent long-term maintenance treatment. Neth J Med 1980;23(6):237-40.

w496 Bleehen SS et al. Cimetidine and chlorpheniramine in the treatment of chronic idiopathic urticaria: a multi-centre randomized double-blind study. Brit J Dermatol 1987;117(1):81-8.

w497 Commens CA et al. Cimetidine in chronic idiopathic urticaria: a randomized double-blind study. Brit J Dermatol 1978;99(6):675-9.

w498 Cook J et al. Lack of effect of H2 blockade in chronic urticaria. Brit J Dermatol 1979;101(Suppl.):2.

w499 Palmer RH et al. Cimetidine QID and BID in rapid heartburn relief and healing of lesions in gastroesophageal reflux disease. Clin Ther 1993;15(6):994-1001.

w500 Sitsen JM et al. Concomitant use of mirtazapine and cimetidine: a drug-drug interaction study in healthy male subjects. Eur J Clin Pharmacol 2000;56(5):389-94.

w501 Powell-Jackson P et al. Effect of cimetidine in symptomatic gastro-oesophageal reflux. Lancet 1978;2(8099):1068-9.

w502 Wesdorp E et al. Oral cimetidine in reflux esophagitis: a double blind controlled trial. Gastroenterology 1978;74(5:Pt 1):821-4.

w503 Palmer RH et al. Cimetidine 800mg twice daily for healing erosions and ulcers in gastroesophageal reflux disease. J Clin Gastroenterol 1990;12(Suppl.):29-34.

w504 Fitzpatrick WJ et al. Ileal resection: effect of cimetidine and taurine on intrajejunal bile acid precipitation and lipid solubilisation. Gut 1986;27(1):66-72.

w505 Matthews CAN et al. The effect of H1 and H2 histamine antagonists on symptomatic dermographism. Brit J Dermatol 1979;101:57-61.

w506 Jameson MB et al. A randomized, placebo-controlled, double-blind phase II trial of peri-operative cimetidine in early colorectal cancer. Journal of Clinical Oncology Conference. 2014;32(3 SUPPL. 1).

w507 Baglioni A et al. Famotidine versus placebo in prevention of the recurrence of duodenal ulcer disease. A multicenter study in Germany, Austria and Italy. Z Gastroenterol 1985;23(12):665-9. [German]

w508 Berlin RG et al. Nocturnal therapy with famotidine for 1 year is effective in preventing relapse of gastric ulcer. Aliment Pharm Therap 1991;5(2):161-71.

w509 Texter EC et al. Maintenance therapy of duodenal ulcer with famotidine. A multicenter United States study. Am J Med 1986;81(4B):25-32.

w510 Zaterka S et al. Famotidine in the short and long-term treatment of duodenal ulcer. Acta Gastroenterologica Latinoamericana 1988;18(2):115-21. [Spanish].

w511 Hongo M et al. A randomized, double-blind, placebo-controlled clinical study of the histamine H2-receptor antagonist famotidine in Japanese patients with nonerosive reflux disease. J Gastroenterol 2008;43(6):448-56.

w512 Taha AS et al. Famotidine for the prevention of peptic ulcers and oesophagitis in patients taking low-dose aspirin (FAMOUS): a phase III, randomised, double-blind, placebo-controlled trial. Lancet 2009;374:119-25.

w513 Taha AS et al. Famotidine for the prevention of gastric and duodenal ulcers caused by non-steroidal anti-inflammatory drugs. New Engl J Med 1996;334(22):1435-9.

w514 Kato M et al. Randomized, double-blind, placebo-controlled crossover trial of famotidine in patients with functional dyspepsia. Aliment Pharm Therap 2005;21(Suppl.2):27-31.

w515 Poyurovsky M et al. The effect of famotidine addition on olanzapine-induced weight gain in first-episode schizophrenia patients: A double-blind placebo-controlled pilot study. Eur Neuropsychopharm 2003;14(4):332-6.

w516 Schwartz JI et al. Novel oral medication delivery system for famotidine. J Clin Pharmacol 1995;35(4):362-7.

w517 Robinson M et al. Famotidine (20mg) b.d. relieves gastrooesophageal reflux symptoms in patients without erosive oesophagitis. Famotidine/GERD Investigation Group. Aliment Pharm Therap 1991;5(6):631-43.

w518 Simon TJ et al. Randomized, placebo-controlled comparison of famotidine 20mg b.d. or 40mg b.d. in patients with erosive oesophagitis. Aliment Pharm Therap 1994;8(1):71-9.

w519 Simon TJ et al. Acid suppression by famotidine 20mg twice daily or 40mg twice daily in preventing relapse of endoscopic recurrence of erosive esophagitis. Clin Ther 1995;17(6):1147-56.

w520 Simon TJ et al. Effect of normalization of esophageal acid reflux time on recurrence of erosive esophagitis: Randomized, placebo-controlled trial of two doses of famotidine. Curr Ther Res Clin E 1996;57(1):16-25.

w521 Liu CC et al. Maintenance treatment is not necessary after Helicobacter pylori eradication and healing of bleeding peptic ulcer: a 5-year prospective, randomized, controlled study. Arch Intern Med 2003;163(17):2020-4.

w522 Sabesin SM et al. Famotidine relieves symptoms of gastroesophageal reflux disease and heals erosions and ulcerations: Results of a multicenter, placebo-controlled, dose-ranging study. Arch Intern Med 1991;151(12):2394-400.

w523 Ohara S et al. Efficacy evaluation of lafutidine for mild reflux esophagitis in Japanese patients. J Japn Soc Gastroenterol 2010;107(4):588-97. [Japanese]

w524 Ohara S et al. A double-blind, controlled study comparing lafutidine with placebo and famotidine in Japanese patients with mild reflux esophagitis. J Gastroenterol 2010;45(12):1219-27.

w525 Hsu P.I et al. (2015). Can famotidine prevent recurrent peptic ulcer in platelet ADP-receptor antagonist users?-a randomized-controlled, double-blind, and double-dummy trial. J Gastroenterol Hepatol 30;153.

w526 Hsu PI. Can histamine-2 receptor antagonist prevent recurrent peptic ulcer in thienopyridine users? J Gastroenterol Hepatol. 2013;28:64.

w527 Laine L, Kivitz AJ, Bello AE, Grahn AY, Schiff MH, Taha AS. Double-blind randomized trials of single-tablet ibuprofen/high-dose famotidine vs. ibuprofen alone for reduction of gastric and duodenal ulcers. American Journal of Gastroenterology. 2012;107(3):379-86.

w528 Hudson N et al. Famotidine for healing and maintenance in nonsteroidal anti-inflammatory drug-associated gastroduodenal ulceration. Gastroenterology 1997;112(6):1817-22.

w529 Marrero JM et al. Effect of famotidine on oesophageal sensitivity in gastro-eoesophageal reflux disease. Gut 1994;35(4):447-50.

w530 Mestre TA et al. A pilot study evaluating the histamine H2 antagonist, famotidine, for levodopa-induced dyskinesia in Parkinson's disease. Mov Disord 2014;29:S250-S1.

w531 Meskanen K et al. A randomized clinical trial of histamine 2 receptor antagonism in treatment-resistant schizophrenia. J Clin Psychopharmacol 2013;33(4):472-8.

w532 Namikawa T et al. Feasibility study of supportive care using lafutidine, a histamine H2 receptor antagonist, to prevent gastrointestinal toxicity during chemotherapy for gastric cancer. Anticancer Research 2014;34(12):7297-301.

w533 Kato M et al. Lafutidine prevents low-dose aspirin and loxoprofen induced gastric injury: a randomized, double-blinded, placebo controlled study. J Gastroenterol Hepatol. 2010;25(10):1631-5.

w534 Carlson MC et al. H2 histamine receptor blockade in the treatment of Alzheimer disease: a randomized, double-blind, placebo-controlled trial of nizatidine. Alz Dis Assoc Dis 2002;16(1):24-30.

w535 Cerulli MA et al. Nizatidine as maintenance therapy of duodenal ulcer disease in remission. Scand J Gastroentero 1987;136(Suppl.):79-83.

w536 Rampal P et al. A quality of life study in five hundred and eighty-one duodenal ulcer patients. Maintenance versus intermittent treatment with nizatidine. Scand J Gastroentero 1994;206(Suppl.):44-51.

w537 Di Mario F et al. Nizatidine 150mg at night in the prophylaxis of gastric ulcer relapse: a 12-month prospective, randomized, double-blind multicentre study versus placebo. Eur J Gastroen Hepat 1993;5(2):97-102.

w538 Levine LR et al. Nizatidine prevents peptic ulceration in high-risk patients taking nonsteroidal anti-inflammatory drugs. Arch Intern Med 1993;153(21):2449-54.

w539 Cavazzoni P et al. Nizatidine for prevention of weight gain with olanzapine: A double-blind placebo-controlled trial. Eur Neuropsychopharm 2003;13(2):81-5.

w540 Dammann HG et al. Effective prevention of piroxicam-induced lesions of the gastric mucosa with nizatidine. Z Gastroenterol 1990;28(2):94-6. [German]

w541 Hansen JM et al. Placebo-controlled trial of cisapride and nizatidine in unselected patients with functional dyspepsia. Am J Gastroenterol 1998;93(3):368-74.

w542 Cloud ML et al. Nizatidine versus placebo in gastroesophageal reflux disease: a 12-week, multicenter, randomized, double-blind study. Am J Gastroenterol 1991;86(12):1735-42.

w543 Atmaca M et al. Nizatidine treatment and its relationship with leptin levels in patients with olanzapine-induced weight gain. Hum Psychopharm 2003;18(6):457-61.

w544 Atmaca M et al. Nizatidine for the treatment of patients with quetiapine-induced weight gain. Hum Psychopharm 2004;19(1):37-40.

w545 Cloud ML et al. Nizatidine versus placebo in gastroesophageal reflux disease. A six-week, multicenter, randomized, double-blind comparison. Digest Dis Sci 1992;37(6):865-74.

w546 Dobrilla G et al. Treatment of erosive reflux oesophagitis: a double-blind multicentre trial with nizatidine 300mg b.i.d. versus placebo. Ital J Gastroenterol 1992;24(6):338-41.

w547 Koskenpato J et al. Nizatidine and gastric emptying in functional dyspepsia. Digest Dis Sci 2008;53(2):352-7.

w548 Quik RF et al. A comparison of two doses of nizatidine versus placebo in the treatment of reflux oesophagitis. Aliment Pharm Therap 1990;4(2):201-11.

w549 Cloud ML et al. Healing and recurrence of active duodenal ulcer with nizatidine. Clin Pharmacol Ther 1989;46(3):310-6.

w550 Assuncao SS et al. Weight gain management in patients with schizophrenia during treatment with olanzapine in association with nizatidine.[Erratum appears in Rev Bras Psiquiatr. 2007;29(1):v]. Revista Brasileira de Psiquiatria 2006;28(4):270-6.

w551 Guerreiro S et al. [Prospective study of duodenal ulcer. Analysis of prognostic factors]. Acta Med Port 1991;4(4):191-7. [Portuguese]

w552 Murakami K et al. Maintenance therapy with H2-receptor antagonist until assessment of Helicobacter pylori eradication can reduce recurrence of peptic ulcer after successful eradication of the organism: prospective randomized controlled trial. J Gastroen Hepatol 2006;21(6):1048-53.

w553 Vakil N et al. The effect of over-the-counter ranitidine 75mg on night-time heartburn in patients with erosive oesophagitis on daily proton pump inhibitor maintenance therapy. Aliment Pharm Therap 2006;23(5):649-53.

w554 Mignon M et al. Intermittent treatment or prevention of recurrence in duodenal ulcer disease? A controlled, double-blind study with one-a-day ranitidine 150 mg for one year. Gastroen Clin Biol 1990;14(10):732-8. [French]

w555 Andrade EO et al. Controlled study of ranitidine for prevention of recurrent duodenal ulcer. Rev Gastro-ent Mex 1997;62(2):94-7. [Spanish]

w556 Kang JY et al. Dietary supplementation with pectin in the maintenance treatment of duodenal ulcer. A controlled study. Scand J Gastroentero 1988;23(1):95-9.

References for trials listed in Webtables 1-7

w557 Piai G et al. The influence of severe bulb deformity on duodenal ulcer relapse. Hepato-Gastroenterol 1990;37(1):131-4.

w558 Van Deventer GM et al. A randomized study of maintenance therapy with ranitidine to prevent the recurrence of duodenal ulcer. New Engl J Med 1989;320(17):1113-9.

w559 Kozarek R et al. Maintenance therapy with ranitidine following healing of acute duodenal ulcer. Curr Ther Res Clin E 1985;38(2):341-51.

w560 Alstead EM et al. Raniditine in the prevention of gastric and duodenal ulcer relapse. Gut 1983;24(5):418-20.

w561 Koelz HR et al. Healing and relapse of reflux esophagitis during treatment with ranitidine. Gastroenterology 1986;91(5):1198-205.

w562 Boyd EJS et al. Safety of ranitidine maintenance treatment of duodenal ulcer. Scand J Gastroentero 1984;19(3):394-400.

w563 Di Mario F et al. Ranitidine in long-term duodenal ulcer treatment: a multicentre trial. Digestion 1982;24(4):260-3.

w564 Dawson J et al. Effect of ranitidine and cimetidine on gastric ulcer healing and recurrence. Scand J Gastroentero 1984;19(5):665-8.

w565 Di Mario F et al. Long-term treatment of patients with gastric ulcer: Sucralfate versus rantidine versus no treatment: An interim report at the end of year 1 of a 3-year multicenter. Curr Ther Res Clin E 1994;55(9):1117-25.

w566 Jensen DM et al. A controlled study of ranitidine for the prevention of recurrent haemorrhage from duodenal ulcer. New Engl J Med 1994;330(6):382-6.

w567 Mones J et al. Helicobacter pylori eradication versus one-year maintenance therapy: effect on relapse and gastritis outcome. Revista Espanola de Enfermedades Digestivas 2001;93(6):372-89. [Spanish]

w568 Murray WR et al. Maintenance Ranitidine Treatment after Haemorrhage from a Duodenal Ulcer. A 3-year Study. Scand J Gastroentero 1987;23(2):183-7.

w569 Mekel RC et al. Prevention of duodenal ulcer relapse with ranitidine. S Afr Med J 1985;6:385-6.

w570 Sue SO et al. Effectiveness of ranitidine 150 mg at bedtime as maintenance therapy for healed gastric ulcers. Clin Ther 1996;18(6):1175-83.

w571 Jorde R et al. Ranitidine 150mg at night in the prevention of gastric ulcer relapse. Gut 1987;28(4):460-3.

w572 Ruszniewski P et al. Two year maintenance treatment of duodenal ulcer disease with ranitidine 150mg: a prospective multicentre randomised study. GEMUD (Groupe d'Etude de la Maladie Ulcereuse Duodenale). Gut 1993;34(12):1662-5.

w573 Manes G et al. Increased risk for Helicobacter pylori recurrence by continuous acid suppression: a randomized controlled study. Ital J Gastroenterol 1998;30(1):28-33.

w574 Zdrenghea D et al. The effect of simvastatin associated with ranitidine and alcohol upon serum lipids. Romanian Journal of Internal Medicine 2004;42(1):143-8.

w575 Hovdenak N et al. Drug prophylaxis in duodenal ulcer disease. A placebo-controlled trial with ranitidine. Tidsskrift for den Norske Laegeforening 1987;107(1):29-30. [Norwegian]

w576 Villalobos JJ et al. The recurrence of duodenal ulcer in patients undergoing treatment with ranitidine and placebo. A multicenter study. Revista de Gastroenterologia de Mexico 1991;56(2):65-9. [Spanish]

w577 Mehta VS et al. Role of ranitidine in negative symptoms of schizophrenia--an open label study. Asian Journal of Psychiatry. 2014;12:150-4.

w578 Tamura S et al. Prospective randomized study on the effect of ranitidine against injection ulcer after endoscopic injection sclerotherapy for esophageal varices. Am J Gastroenterol 1991;86(4):477-80.

w579 Penston JG et al. A placebo-controlled investigation of duodenal ulcer recurrence after withdrawal of long-term treatment with ranitdine. Aliment Pharm Therap 1993;7(3):259-65.

w580 Ehsanullah RSB et al. Prevention of gastroduodenal damage induced by non-steroidal anti-inflammatory drugs: controlled trial of ranitidine. Brit Med J 1988;297(6655):1017-21.

w581 Robinson MG et al. Effect of ranitidine on gastroduodenal mucosal damage induced by nonsteroidal anti-inflammatory drugs. Digest Dis Sci 1989;34(3):424-8.

w582 Van Groenendael JH et al. The effect of ranitidine on NSAID related dyspeptic symptoms with and without peptic ulcer disease of patients with rheumatoid arthritis and osteoarthritis. Clin Rheumatol 1996;15(5):450-6.

w583 Robinson M et al. Ranitidine prevents duodenal ulcers associated with non-steroidal anti-inflammatory drug therapy. Aliment Pharm Therap 1991;5(2):143-50.

w584 Swift GL et al. Effect of ranitidine on gastroduodenal mucosal damage in patients on long-term non-steroidal anti-inflammatory drugs. Digestion 1989;44(2):86-94.

w585 Berkowitz JM et al. Ranitidine protects against gastroduodenal mucosal damage associated with chronic aspirin therapy. Arch Intern Med 1987;147(12):2137-9.

w586 Nielsen HJ et al. The effect of ranitidine on long-term survival in primary colorectal cancer. A 40 months interim analysis. GI Can 1998;2(3):227-33.

w587 Muller P et al. High-dose ranitidine protects completely stomach and duodenum against piroxicam. An endoscopic double-blind pilot-study. Z Gastroenterol 1992;30(12):851-3. [German]

w588 Muller P et al. Protection from gastroduodenal adverse effects of acetylsalicylic acid with ranitidine. An endoscopic controlled double-blind study of healthy subjects. Arzneimittel-Forsch 1991;41(6):638-9. [German]

w589 Nervi M et al. Usefulness of ranitidine in the treatment of esophagitis caused by gastro-esophageal acid reflux. Min Diet Gastroent 1989;35(3):195-9. [Italian]

w590 Rush DR et al. Clinical effectiveness and quality of life with ranitidine vs placebo in gastroesophageal reflux disease patients: A clinical experience network (CEN) study. J Fam Practice 1995;41(2):126-36.

w591 Kar P et al. Ranitidine versus placebo: A double-blind trial in the management of reflux esophagitis. Curr Ther Res Clin E 1990;47(4):731-4.

w592 Yanagawa A et al. Prophylactic efficacy of the H2-blocker ranitidine against gastroduodenal lesions caused by non-steroidal anti-inflammatory drugs (NSAIDs): Controlled trial study. Jpn J Rheum 1991;3(4):275-87.

w593 Alberts DS et al. Lack of ranitidine effects on cyclophosphamide bone marrow toxicity or metabolism: A placebo-controlled clinical trial. J Natl Cancer I 1991;83(23):1739-43.

w594 Yanagawa A et al. Prophylactic efficacy of ranitidine against gastroduodenal mucosal damage from non-steroidal anti-inflammatory drugs: A randomized placebo-controlled study. Round Table Series - Roy Soc Med 1990;(21):97-103.

w595 Muller P et al. Protective effects of ranitidine in stomach and duodenum against piroxicam. An endoscopically controlled double-blind study. Arzneimittel-Forsch 1992;42(12):1492-4. [German]

w596 Simon B et al. Ranitidine in the therapy of NSAID-induced gastroduodenal lesions. Results of a randomized, placebo-controlled, double-blind study in patients with rheumatic diseases. Scand J Gastroentero 1988;23(154)(Suppl.):18-21.

w597 Primrose JN et al. A prospective randomised controlled study of the use of ranitidine in patients with gastric cancer. Gut 1998;42(1):17-9.

w598 Berenson M et al. Effect of Smoking in a controlled study of ramititdine treatment in Gastroesophageal reflux Disease. J Clin Gastroenterol 1987;(5):499-503.

w599 Robinson MG et al. Do endoscopic findings influence response to H2 antagonist therapy for gastroesophageal reflux disease? Am J Gastroenterol 1987;82(6):519-22.

w600 Saunders JHet al. Dyspepsia: Incidence of non-ulcer disease in a controlled trial of ranitidine in general practice. Brit Med J 1986;292(6521):665-8.

w601 Hendel L et al. Long-term ranitidine in progressive systemic sclerosis (scleroderma) with gastroesophageal reflux. Scand J Gastroentero 1986;21(7):799-805.

w602 Johansson K-E et al. Double-blind crossover study of ranitidine and placebo in gastro-oesophageal reflux disease. Scand J Gastroentero 1986;21(7):769-78.

w603 Muller P et al. Ranitidine in the treatment of non-ulcer dyspepsia. A placebo-controlled study in the Federal Republic of Germany. Arzneimittel-Forsch 1994;44(10):1130-2. [German]

w604 Hegarty JH et al. Prevention de la recurrence de l'oesophagite de reflux: Etude controlee par placebo sur la ranitidine 150mg b.i.d. et 300mg b.i.d. Can J Gastroenterol 1997;11(1):83-8. [French]

w605 Gustavsson S et al. Reflux esophagitis: assessment of therapy effects and observer variation by video documentation of endoscopy findings. Scand J Gastroentero 1987;22(5):585-91.

w606 Fullarton GM et al. The effect of H2-blockade on plasma gastrin concentration in patients with an achlorhydric stomach. Aliment Pharm Therap 1992;6(5):557-63.

w607 Hallerback B et al. Gastro-oesophageal reflux symptoms-clinical findings and effect of ranitidine treatment. Eur J Surg 1998;583(Suppl.):6-13.

w608 Orlowski T et al. The influence of the H-2 receptor antagonist ranitidine on cadaver kidney allograft function. Int Urol Nephrol 1986;18(2):205-10.

w609 Olubuyide IO et al. Non-ulcer dyspepsia in Nigerians clinical and therapeutic results. Scand J Gastroentero 1986;124(Suppl.):83-7.

w610 Sherbaniuk R et al. Ranitidine in the treatment of symptomatic gastroesophageal reflux. Clin Res 1984;30(2):289A.

w611 Nielsen HJ et al. The effect of ranitidine on post-operative infectious complications after elective colorectal surgery. GI Cancer 1999;3(2):87-95.

w612 Wotherspoon HA et al. Randomized controlled trial of an H2-receptor antagonist in gastric cancer. Brit J Surg 1997;84(8):1168-9.

w613 Tan CC et al. Ranitidine reduces phosphate binding in dialysis patients receiving calcium carbonate. Nephrol Dial Transpl 1996;11(5):851-3.

w614 Smith AD et al. Dyspepsia on withdrawal of ranitidine in previously asymptomatic volunteers. Am J Gastroenterol 1999;94(5):1209-13.

w615 Singh K et al. Impact of ranitidine on prognosis after simple closure of perforated duodenal ulcer. Trop Gastroentero 1999;20(2):90-2.

w616 Goy JA et al. Ranitidine and placebo in the treatment of reflux oesophagitis. A double-blind randomized trial. Med J Australia 1983;2(11):558-61.

w617 Wallace MH et al. Randomized, placebo-controlled trial of gastric acid-lowering therapy on duodenal polyposis and relative adduct labeling in familial adenomatous polyposis. Dis Colon Rectum 2001;44(11):1585-9.

w618 Czapla K et al. Ranitidine reduces aluminium toxicity in patients with renal failure. Nephrol Dial Transpl 1992; 7:1246-8.

w619 Hadi S et al. Clinical investigation of ranitidine in patients with gastritis. Clin Ther 1989;11(5):590-4.

w620 Farup PG et al. Ranitidine effectively relieves symptoms in a subset of patients with functional dyspepsia. Scand J Gastroentero 1997;32(8):755-9.

w621 Wesdorp IC et al. Treatment of reflux oesophagitis with ranitidine. Gut 1983;24(10):921-4.

w622 Muller P et al. Ranitidine protects the human stomach and duodenal mucosa against low-dose acetylsalicylic acid. Zeitschrift fur Gastroenterologie 1989;27(12):722-4. [German]

w623 Simon B et al. Clinical studies on acid inhibition by ranitidine under simultaneously administered pentagastin. Arzneimittel-Forsch1992;42(2):133-5. [German]

References for trials listed in Webtables 1-7

w624 Elizalde RO et al. Reflux esophagitis peptic ulcer duodenal and gastric treatment with ranitidine. Preliminary report of double-blind multi-institutional study. Invest Med Int 1983;10(1):3-6. [Spanish]

w625 Metzenroth H et al. Prevention of ossification after total hip endoprosthesis with indomethacin and its effect on gastric mucosa. Zeitschrift fur Orthopadie und Ihre Grenzgebiete 1991;129(2):178-82. [German]

w626 Lopez-Mato A et al. Randomized, open label study on the use of ranitidine at different doses for the management of weight gain associated with olanzapine administration. Vertex: Revista Argentina de Psiquiatria 2003;14(52):85-96. [Spanish]

w627 Zonneveld IM et al. Ranitidine does not affect psoriasis: A multicenter, double-blind, placebo-controlled study. J Am Acad Dermatol 1997;36(6 I):932-4.

w628 Grove O et al. Ranitidine and high-dose antacid in reflux oesophagitis. A randomized, placebo-controlled trial. Scand J Gastroentero 1985;20(4):457-61.

w629 Corinaldesi R et al. Patients with duodenitis have gastric secretory and motor functions like those of duodenal ulcer patients: Results of a short-term treatment with ranitidine. J Clin Gastroenterol 1991;13(3):296-302.

w630 Hine KR et al. Ranitidine in reflux oesophagitis. A double-blind placebo-controlled study. Digestion 1984;29(2):119-23.

w631 Ideo G et al. Nadolol can prevent the first gastrointestinal bleeding in cirrhotics: a prospective, randomized study. Hepatology 1988;8(1):6-9.

w632 Farup PG et al. Long-term treatment with 300 mg ranitidine once daily after dilatation of peptic oesophageal strictures. Scand J Gastroenterol 1992;27(7):594-8.

w633 Lehtola J et al. Ranitidine, 150mg three times a day, in the treatment of reflux oesophagitis. A placebo-controlled, double-blind study. Scand J Gastroentero 1986;21(2):175-80.

w634 Wilder-Smith CH et al. Tolerance to oral H2-receptor antagonists. Digest Dis Sci 1990;35(8):976-83.

w635 Ten Wolde S et al. High-dose ranitidine for the prevention of recurrent peptic ulcer disease in rheumatoid arthritis patients taking NSAIDs. Aliment Pharm Therap 1996;10(3):347-51.

w636 King J et al. A randomised placebo control double-blind trial of ranitidine in patients with colorectal hepatic metastases: Survival advantage in patients with poor cell mediated immunity. Gastrointest Oncol 2002;4(1):39-44.

w637 Veien NK et al. Ranitidine treatment of hand eczema in patients with atopic dermatitis: A double-blind, placebo-controlled trial. J Am Acad Dermatol 1995;32(6):1056-7.

w638 Euler AR et al. Ranitidine is effective therapy for erosive esophagitis. Am J Gastroenterol 1993;88(4):520-4.

w639 Cetin L et al. High-dose ranitidine is ineffective in the treatment of psoriasis. Brit J Dermatol 1997;137(6):1021-2.

w640 Jurlander J et al. Improved vaccination response during ranitidine treatment, and increased plasma histamine concentrations, in patients with B cell chronic lymphocytic leukemia. Leukemia 1995;9(11):1902-9.

w641 Nielson HJ et al. Ranitidine improves certain cellular immune responses in asymptomatic HIV-infected individuals. J Acq Immun Def Synd 1991;4(6):577-84.

w642 Nielsen HJ et al. The effect of ranitidine on cellular immunity in patients with multiple myeloma. Cancer Immunol Immun 1990;32(3):201-5.

w643 Roufail W et al. Ranitidine for erosive oesophagitis: a double-blind, placebo-controlled study. Glaxo Erosive Esophagitis Study Group. Aliment Pharm Therap 1992;6(5):597-607.

w644 Silver MT et al. Ranitidine 300mg twice daily and 150mg four-times daily are effective in healing erosive oesophagitis. Aliment Pharm Therap 1996;10(3):373-80.

w645 Bartlett JA et al. A placebo-controlled trial of ranitidine in patients with early human immunodeficiency virus infection. J Infect Dis 1998;177(1):231-4.

w646 Vendelbo Johansen L et al. Infectious mononucleosis treated by an antihistamine: a comparison of the efficacy of ranitidine (Zantac) vs placebo in the treatment of infectious mononucleosis. Clin Orthop Relat R 1997;22(2):123-5.

w647 Tefera S et al. Bismuth-based combination therapy for Helicobacter pylori-associated peptic ulcer disease (metronidazole for eradication, ranitidine for pain). Am J Gastroenterol 1996;91(5):935-41.

w648 Ranjbar F et al. The effect of ranitidine on olanzapine-induced weight gain. Biomed Res Int. 2013;2013:639391.

w649 Chen ZQ. Influence of Ranitidine on gastrointestinal hemorrhage and thrombosis caused by dual antiplatelet therapy after PCI in patients with coronary heart disease. Cardiology. 2010;117:48-9.

w650 Simon B et al. 75 mg roxatidine nocte protects human gastric mucosa against 300 mg acetylsalicylic acid nocte. Z Gastroenterol 1991;29(11):599-601. [German]

w651 The European Cooperative Roxatidine Study Group. Roxatidine acetate as maintenance treatment for patients with peptic ulcer disease. Verdauungskrankheiten 1991;9(6):231-8. [German]

w652 Dammann HG et al. The comparison of extemporaneous preparations of omeprazole, pantoprazole oral suspension and intravenous pantoprazole on the gastric pH of critically ill-patients. Arzneimittel Forsch 1995; 45(8):880-882.[German]

w653 Muller P et al. Comparison of the protective effects of roxatidine and misoprostol on diclofenac gastroduodenal pathology. An endoscopic, controlled study of volunteers. Arzneimittel Forsch 1994;44(10):1127-9. [German]

w654 Lassman H.B et al. 1988 The pharmacodynamics and pharmacokinetics of multiple doses of the new H2-receptor antagonist, roxatidine acetate, in healthy men. Drugs 35(Suppl. 3):53-64.

w655 Braverman AJ et al. Dose validation and study design criteria in current cimetidine studies. Clin Ther 1986;8(Suppl):49-56.

w656 Young MD et al. Determining the optimal dosage regimen for H2-receptor antagonist therapy-a dose validation approach. Aliment Pharm Therap 1989;3(1):47-57.

w657 Domschke W et al. Kinetics of duodenal ulcer healing: effect of treatment with cimetidine. Acta Hep-Gastroenterol 1976;23(6):441-3.

w658 Frank WO et al. Once-daily bedtime dosing regimen of cimetidine in the treatment of gastric ulcer. Clin Ther 1989;11(5):595-603.

w659 Bodemar G et al. Cimetidine in the treatment of active duodenal and prepyloric ulcers. Lancet 1976;2(7978):161-4.

w660 Frank WO et al. Acute treatment of benign gastric ulcer with once-daily bedtime dosing of cimetidine compared with placebo. Aliment Pharm Therap 1989;3(6):573-84.

w661 Mosci AS et al. Relationship between Sakita endoscopic stage and duration of treatment for duodenal ulcer healing. Braz J Med Biol Res 1989;22(10):1219-23.

w662 Tay HH et al. Twice daily cimetidine in the initial treatment of chronic gastric ulcer-a double-blind placebo-controlled trial. Med J Malaysia 1988;43(2):181-5.

w663 Moshal MG et al. Treatment of duodenal ulcers with cimetidine. S Afr Med J 1977;19:760-3.

w664 Hoffenberg P et al. Comparative treatment of gastric ulcer with pirenzepin, cimetidine and placebo. Revista Medica de Chile 1985;113(6):537-40. [Spanish]

w665 Isenberg J et al. Double-blind comparisons of cimetidine and low-dose antacid versus placebo in the healing of benign gastric ulcer. Gastroenterology 1982;82(5 II):1090.

w666 Hentschel E et al. Therapy of duodenal and prepyloric ulcers with cimetidine (author's transl). Wien Klin Wochenschr 1979;91(2):53-7. [German]

w667 Garbol J et al. A controlled trial of cimetidine (Tagamet) in the treatment of stomach ulcer. Ugeskrift for Laeger 1979;141(48):3301-3. [Danish]

w668 Tarnok F et al. Comparative study of the effects of placebo, atropine, cimetidine and carbenoxolone on patients with duodenal ulcer. Orvosi Hetilap 1980;121(26):1567-70. [Hungarian]

w669 Peter P et al. Cimetidine in the treatment of duodenal ulcer (author's transl). Deut Med Wschr 1978;103(29):1163-6. [German]

w670 Fica V et al. Comparative study of the efficacy of ranitidine, cimetidine (Asiloc), De-Nol and spirulina in the treatment of gastric and duodenal ulcer. Rev Med Int, Neuro, Psi, Neuro, Derm-Ven 1987;Medicina(1):21-35. [Romanian]

w671 Bongiorno A et al. Cimetidine, pirenzepine, trithiozine in duodenal ulcer: Blind study versus placebo. Curr Ther Res Clin E 1983;34(4 II):718-27.

w672 Marini U et al. A double blind trial of cimetidine and fentonium bromide, alone or combined, in the healing of duodenal ulceration. Curr Ther Res Clin E 1980;27(2):239-46.

w673 Mazure PA et al. Cimetidine in active duodenal ulcer. Curr Ther Res Clin E 1980;27(4):601-7.

w674 Lam SK et al. Accurate prediction of duodenal-ulcer healing rate by discriminant analysis. Gastroenterology 1983;85(2):403-12.

w675 Dobrilla G et al. Pirenzepine and cimetidine in the short-term treatment of duodenal ulcer: A vs-placebo endoscopic controlled trial. Curr Ther Res Clin E 1980;28(3 I):371-6.

w676 Danielsson A et al. The relationship between active peptic ulcer, endoscopic duodenitis and symptomatic state after treatment with cimetidine. Ann Clin Res 1980;12(1):5-12.

w677 Strom M et al. Antacid/anticholinergic, cimetidine, and placebo in treatment of active peptic ulcers. Scand J Gastroentero 1981;16(5):593-602.

w678 Corinaldesi R et al. Evaluation of basal and pentagastrin stimulated gastric secretion after treatment withdrawal opposite to a placebo therapy. Policlinico - Sezione Medica 1980;87(3):246-50. [Italian]

w679 Johannessen T et al. 1- and 4-week cimetidine treatment for duodenal ulcer. Scand J Gastroentero 1986;21(6):701-4.

w680 Frost F et al. Cimetidine in patients with gastric ulcer: a multicentre controlled trial. Brit Med J 1977;2(6090):795-7.

w681 Giger M et al. Therapy of duodenal ulcer with cimetidine, pirenzipine and placebo: report on a double-blind randomized study. Schweiz Med Wschr 1979;16:617-8. [German]

w682 Ciclitira PJ et al. Double-blind controlled trial of cimetidine in the healing of gastric ulcer. Gut 1977;20(8):730-4.

w683 Bianchi Porro G et al. Pirenzepine versus cimetidine in duodenal ulcer. A double-blind, placebo-controlled, short-term clinical trial. Digestion 1982;23(2):110-5.

w684 Buligescu L et al. The value of cimetidine in the treatment of gastroduodenal ulcer. Medecine Interne 1985;23(1):51-5.

w685 Gotthard R et al. Treatment of active prepyloric and duodenal ulcers with antacid/anticholinergic, cimetidine and placebo. Scand J Gastroentero 1982;75(Suppl.):86-96.

w686 Gray GR et al. Oral cimetidine in severe duodenal ulceration. A double-blind controlled trial. Lancet 1977;1(8001):4-7.

w687 Grewal GS et al. Cimetidine in treatment of duodenal ulcer: results of a short term double blind study. J Assoc Physicians India 1979;27(9):785-9.

w688 Marini G et al. Endoscopic evaluation of sulglycotide on healing duodenal ulcer. A controlled double-blind trial with two dosage levels. Clin Trials J 1981;18(2):105-13.

w689 Gotlieb-Jensen K et al. Occurrence of Candida in gastric ulcers. Significance for the healing process. Gastroenterology 1983;85(3):535-7.

w690 Kennedy T et al. Cimetidine for recurrent ulcer after vagotomy or gastrectomy: a randomised controlled trial. Brit Med J 1978;1(6122):1242-3.

References for trials listed in Webtables 1-7

w691 Giacosa A et al. Comparison between ranitidine, cimetidine, pirenzepine and placebo in the short term treatment of duodenal ulcer. Scand J Gastroentero 1982;72(Suppl.):215-9.

w692 Monnis M et al. Effects of sucralfate on gastric acid secretion and duodenal ulcer healing. J Assoc Phys India 1988;36(4):251-3.

w693 Mukhtar ED et al. Double-blind comparative study of cimetidine 1gm daily and placebo in the treatment of patients with severe duodenal ulceration in the Sudan. E Afr Med J 1982;59(6):413-5.

w694 Guha Mazumdar DN et al. H-2 receptor antagonist (cimetidine) in the treatment of chronic duodenal ulcer and its effect on gastric secretions. J Assoc Phys India 1981;29(12):999-1006.

w695 Manousos ON et al. A double-blind study of cimetidine in patients with duodenal or gastric ulcer in Greece. J Int Med Res 1978;6(5):381-3.

w696 Falaiye JM et al. A double-blind comparative study of cimetidine and placebo in adult Nigerian duodenal ulcer patients with special reference to gastroduodenal mucosal morphology and intestinal bacterial activity. Afr J Med Medi Sci 1979;8(1-2):61-70.

w697 Vantini I et al. Serum fasting gastrin levels after short-term treatment with cimetidine in patients with duodenal ulcer. Acta Hepato-Gastro 1978;25(5):376-9.

w698 Tytgat GN et al. Controlled trial comparing colloidal bismuth subcitrate tablets, cimetidine and placebo in the treatment of gastric ulceration. Scand J Gastroentero 1982;80(Suppl.):31-8.

w699 Chen PC et al. Double-blind therapeutic trial of active duodenal ulcer disease with 'cimetidine'. J Formos Med Assoc 1979;78(9):777-83. [Chinese].

w700 Zespolowa P et al. Multicentre controlled clinical trial of gastric and duodenal ulcer healing with cimetidine. Polski Tygodnik Lekarski 1980;35(2):61-4. [Polish]

w701 Villalobos JJ et al. Cimetidine in the treatment of duodenal ulcer: double-blind study. J Int Med Res 1978;6(4):351-4.

w702 Bardhan KD et al. Comparison of two doses of cimetidine and placebo in the treatment of duodenal ulcer: a multicentre trial. Gut 1979;20(1):68-74.

w703 Patty I et al. Cimetidine and placebo-cimetidine in the treatment of patients with chronic gastric ulcer (a multiclinical randomized double-blind comparative study). Acta Medica Hungarica 1988;45(1):83-95. [Hungarian]

w704 Chen PH et al. A double blind study on short-term cimetidine in the treatment of duodenal ulcer. Taiwan i Hsueh Hui Tsa Chih - Journal of the Formosan Medical Association 1980;79(12):1149-54.

w705 Jensen KG et al. Cimetidine (Tagamet) in the treatment of duodenal ulcer. A controlled trial. Ugeskrift for Laeger 1979;141(48):3298-300. [Danish]

w706 Gudmand-Hoyer E et al. A pragmatic trial of cimetidine in duodenal ulcer patients. Scand J Gastroentero 1977;12(5):611-3.

w707 Villalobos MM et al. Ulcera gastrica:estudio doble ciego comparativo con cimetidina-placebo. Gen 1979;33(1):33-7. [Spanish]

w708 Alcala-Santaella R et al. Controlled study of the treatment of a gastroduodenal ulcer with cimetidine. Revista Espanola de las Enfermedades del Aparato Digestivo 1980;58(2):155-68. [Spanish]

w709 Guelrud M et al. Effectos de los bloqueadores H2 (Cimetidina) en el tratamiento de la ulcera duodenal: estudio doble ciego multicentrico. Gen 1978; 32(4):451-9. [Spanish]

w710 Matos-Villalobos M et al. Ulcera Duodenal: Tratamiento doble ciego con cimetidina-placebo. Gen 1979;33(1):39-42.

w711 Gugler R et al. Cimetidine for anastomotic ulcers after partial gastrectomy. A randomized controlled trial. N Engl J Med 1979;301(20):1077-80.

w712 Festen HP et al. Cimetidine in anastomotic ulceration after partial gastrectomy. Gastroenterology 1979;77(1):83-5.

w713 O'Laughlin JC et al. Resistance to medical therapy of gastric ulcers in rheumatic disease patients taking aspirin. A double-blind study with cimetidine and follow-up. Digest Dis Sci 1982;27(11):976-80.

w714 Graham DY et al. Healing of benign gastric ulcer: comparison of cimetidine and placebo in the United States. Ann Intern Med 1985;102(5):573-6.

w715 Akdamar K et al. Cimetidine versus placebo in the treatment of benign gastric ulcer. Gastroenterology 1981;80(5 II):1098.

w716 Binder HJ et al. Cimetidine in the treatment of duodenal ulcer: a multicenter double blind study. Gastroenterology 1978;74(2)(Pt 2):t-8.

w717 Barbezat GO et al. Effect of prolonged cimetidine therapy on gastric acid secretion in man. Gut 1978;19(2):151-4.

w718 Bank S et al. Histamine H2-receptor antagonists in the treatment of duodenal ulcers. S Afr Med J 1976;50:1781-5.

w719 Dolle W et al. The effect of cimetidine on in-patients with duodenal ulcer - a double blind trial. Acta Gastro-ent Belg 1978;41(7-8):424-6.

w720 Collen MJ et al. Cimetidine vs placebo in duodenal ulcer therapy. Six-week controlled double-blind investigation without any antacid therapy. Digest Dis Sci 1980;25(10):744-9.

w721 Clarke AC et al. Effects of cimetidine on the healing of benign gastric ulcers. Aust NZ J Med 1980;10(4):430-2.

w722 Van Deventer GM et al. Sucralfate and cimetidine as single agents and in combination for treatment of active duodenal ulcers. A double-blind, placebo-controlled trial. Am J Med 1985;79(2 C):39-44.

w723 Khanna SS et al. Sucralfate in the short-term treatment of duodenal ulcer. A double-blind comparison with cimetidine and placebo. J Assoc Phys India 1989;37(8):503-5.

w724 Collen M et al. Cimetidine vs. placebo in prepyloric gastric ulcer therapy. Six week controlled double blind investigation without any antacid therapy. Acta Gastroent Latinoameri 1980;10(3):199-202.

w725 Englert E Jr et al. Cimetidine, antacid, and hospitalization in the treatment of benign gastric ulcer: a multicenter double blind study. Gastroenterology 1978;74(2:Pt 2):416-25.

w726 Dyck WP et al. Cimetidine and placebo in the treatment of benign gastric ulcer: a multicenter double blind study. Gastroenterology 1978;74(2:Pt 2):410-5.

w727 Loiudice TA et al. Cimetidine in the treatment of gastric ulcer induced by steroidal and nonsteroidal anti-inflammatory agents. Am J Gastroenterol 1981;75(2):104-10.

w728 Gaon D et al. Effect of cimetidine on lactase activity in patients with duodenal ulcer. Acta Gastroenterologica Latinoamericana 1981;11(2):285-90. [Spanish]

w729 Isenberg JI et al. Healing of benign gastric ulcer with low-dose antacid or cimetidine. A double-blind, randomized, placebo-controlled trial. N Engl J Med 1983;308:1319-24.

w730 Landecker KD et al. Cimetidine and gastric ulcer healing: a double-blind controlled trial. Med J Australia 1979;2(1):43-5.

w731 Sewing KF et al. In-patient treatment of peptic ulcer with cimetidine. II. Controlled double-blind trial on gastric ulcer patients (author's transl). Deutsche Medizinische Wochenschrift 1978;103(4):152-4. [German]

w732 Davies J et al. The influence of cimetidine on peptic ulcer in patients with arthritis taking anti-inflammatory drugs. Brit J Rheumatol 1986;25(1):54-8.

w733 Blackwood WS et al. Cimetidine in duodenal ulcer. Controlled trial. Lancet 1976;2(7978):174-6.

w734 Machado L et al. Randomized double-blind evaluation of the treatment of gastric ulcer with cimetidine vs placebo. GEN 1978;32(4):461-6. [Spanish]

w735 Simon B et al. Famotidine once-a-day in the therapy of acute, benign gastric ulcer: A worldwide experience. J Clin Gastroenterol 1987;9(Suppl.2):19-22.

w736 Dammann HG et al. Famotidine: Nocturnal administration for gastric ulcer healing. Results of multicenter trials in Austria and Germany. Digestion 1985;32(Suppl.1):45-50.

w737 McCullough AJ et al. Suppression of nocturnal acid secretion with famotidine accelerates gastric ulcer healing. Gastroenterology 1989;97(4):860-6.

w738 Dammann HG et al. Famotidine: proven once-a-day treatment for gastric ulcer. Scand J Gastroentero 1987;134(Suppl.):29-33.

w739 Gitlin N et al. A multicenter, double-blind, randomized, placebo-controlled comparison of nocturnal and twice-a-day famotidine in the treatment of active duodenal ulcer disease. Gastroenterology 1987;92(1):48-53.

w740 Dammann HG et al. Accelerated healing of benign gastric ulcers with a single evening dose of famotidine. Schweizerische Medizinische Wochenschrift 1985;115(14):484-8. [German]

w741 Dyck WP et al. Treatment of duodenal ulceration in the United States. Scand J Gastroentero 1987;136(Suppl.):47-55.

w742 Cloud ML et al. Healing and subsequent recurrence of duodenal ulcer in a clinical trial comparing nizatidine 300-mg and 100-mg evening doses and placebo in the treatment of active duodenal ulcer. Curr Ther Res Clin E 1989;45(3):359-67.

w743 Cloud ML et al. Nizatidine versus placebo in active benign gastric ulcer disease: an eight-week, multicenter, randomized, double-blind comparison. The Nizatidine Benign Gastric Ulcer Disease Study Group. Clin Pharmacol Ther 1992;52(3):307-13.

w744 Eliakim R et al. The effect of nizatidine on duodenal ulcer healing and on mucosal inflammatory mediators. Israel J Med Sci 1994;30(10):751-6.

w745 Levendoglu H et al. Nizatidine: a new histamine receptor blocker in the treatment of active duodenal ulcers. Am J Gastroenterol 1986;81(12):1167-70.

w746 Lam SK et al. Factors influencing healing of duodenal ulcer. Control of nocturnal secretion by H2 blockade and characteristics of patients who failed to heal. Digest Dis Sci 1985;30(1):45-51.

w747 Forssell H et al. Gastroduodenal ulcer healing evaluated by repetitive endoscopic determination of ulcer size in patients on ranitidine or placebo. Curr Ther Res Clin E 1983;33(3 I):459-65.

w748 Dobrilla G et al. Endoscopic double-blind controlled trial of ranitidine vs placebo in the short-term treatment of duodenal ulcer. Hepato-gastroenterol 1981;28(1):49-52.

w749 Berstad A et al. Treatment of duodenal ulcer with ranitidine, a new histamine H2-receptor antagonist. Scand J Gastroentero 1980;15(5):637-9.

w750 Darle N et al. Ranitidine in the treatment of gastric, prepyloric and duodenal ulcer: A controlled prospective double-blind trial. Ann Clin Res 1984;16(1):5-9.

w751 Darle N et al. Ranitidine in the treatment of gastric, prepyloric and duodenal ulcer: A controlled prospective double-blind trial. Annals of Clinical Research 1984;16(1):5-9.

w752 Pan GZ et al. Ranitidine in the treatment of duodenal ulcer. Chinese J Int Med 1987;26(1):30-1. [Chinese]

w753 Martinez RO et al. Gastroduodenal lesions in rheumatoid arthritis. Evaluation and treatment. Acta Gastro-ent Latin 1988;18(2):87-96. [Spanish]

w754 Schulz TB et al. Treatment of gastric ulcer with ranitidine. Scand J Gastroentero 1984;19(1):119-21.

w755 Hirschowitz BI et al. Treatment of benign chronic gastric ulcer with ranitidine. A randomized, double-blind, and placebo-controlled six week trial. J Clin Gastroenterol 1986;8(3 II):371-6.

w756 Marks IN et al. Ranitidine heals duodenal ulcers. S Afr Med J 1982;61(5):152-4.

w757 Lee FI et al. Comparison of twice-daily ranitidine and placebo in the treatment of duodenal ulcer - A multicentre study in the United Kingdom. Hepato-Gastroenterol 1982;29(3):127-9.

w758 Hirschowitz BI et al. A multicenter study of ranitidine treatment of duodenal ulcers in the United States. J Clin Gastroenterol 1986;8(3 II):359-66.

References for trials listed in Webtables 1-7

w759 Pan G et al. Ranitidine in acute duodenal ulcer. Double blind controlled trial. Chinese Med J-Peking 1988;101(4):277-9.

w760 Hirschowitz BI et al. Lessons from the U.S. Multicenter trial of ranitidine treatment for duodenal ulcer. J Clin Gastroenterol 1983;5(Suppl.1):115-22.

w761 Chatterji AN et al. A double-blind and randomised placebo-controlled study of ranitidine in duodenal ulcer patients. Hepato-gastroenterol 1980;27(Suppl.E36);299.

w762 Ashton MG et al. Healing of gastric ulcers after one, two, and three months of ranitidine. Brit Med J Clin Res Ed 1982;284(6314):467-8.

w763 Dobrilla G et al. Placebo controlled studies with ranitidine in duodenal ulcer. Scand J Gastroentero 1981;69(Suppl.):101-7.

w764 Gibinski K et al. The treatment of duodenal and gastric ulcer with ranitidine-a controlled, multicentre clinical trial. Deut Z Verd Stoffwech 1982;42(2-3):64-9. [German]

w765 Nair KV et al. Randomised double blind placebo controlled clinical trial of sucralfate and ranitidine in chronic duodenal ulcer. J Assoc Phys India 1990;38(3):211-2.

w766 Nelis GF et al. Controlled trial with ranitidine in the treatment of peptic ulcer. Neth J Med 1982;24(6):224-8.

w767 Moshal MG et al. Ranitidine in uncomplicated duodenal ulceration. A double-blind endoscopically controlled trial. S Afr Med J 1981;10:393-4.

w768 Murray WR et al. Duodenal Ulcer Healing After Presentation with Haemorrhage. Gut 1986;27(11):1387-9.

w769 Pasquali R et al. Effect of prolonged administration of ranitidine on pituitary and thyroid hormones, and their response to specific hypothalamic-releasing factors. Clin Endocrinol 1981;15(5):457-62.

w770 Johnson JA et al. Ranitidine 300mg at bedtime is effective for gastric ulcers: a 12-wk, multicenter, randomized, double-blind, placebo-controlled comparison. The Ranitidine 300mg HS Gastric Ulcer Study Group. Am J Gastroenterol 1993;88(7):1071-5.

w771 Tildesley G et al. Ranitidine in the treatment of gastric and duodenal ulcers associated with non-steroidal anti-inflammatory drugs. Brit J Rheumatol 1993;32(6):474-8.

w772 Korman MG et al. Ranitidine in duodenal ulcer: incidence of healing and effect of smoking. Digest Dis Sci 1982;27(8):712-5.

w773 Makalinao AU et al. Ranitidine in the treatment of peptic ulcer. Clin Ther 1984;6(2):185-92.

w774 Leroux P et al. Effect of ranitidine on healing of peptic ulcer: a 2-month study. Am J Gastroenterol 1983;78(4):227-30.

w775 Mach T et al. Effectiveness and tolerance of selected Polish antacids in the therapy of duodenal ulcer disease. A placebo- and ranitidine-controlled study. Polski Tygodnik Lekarski 1995;50(1-35):797-800. [Polish]

w776 Mach T et al. A clinical trial on the effect of aluminum containing antacids on the course of duodenal ulcer healing and morphology of the gastric mucosa. Folia Medica Cracoviensia 1995;36(1-4):3-20. [Polish]

w777 Yap I et al. Ranitidine in the acute treatment of duodenal ulcer-a double-blind placebo-controlled trial. Singap Med J 1985;26(7):539-42.

w778 Roberts DM et al. Clinical trial of ranitidine in the treatment of peptic ulcer. Brit J Clin Pract 1982;36(1):9-12.

w779 Mach T et al. Low-dose antacids versus ranitidine in the short-term treatment of patients with duodenal ulcer. Endoscopic and histologic placebo-controlled study. Materia Medica Polona 1992;24(3):201-4. [Polish]

w780 Dupas JL et al. Acid suppression therapy is not required after one-week anti-Helicobacter pylori triple therapy for duodenal ulcer healing. Gastroen Clin Biol 2000;24(6-7):638-43. [French]

w781 Mach T et al. Effect of selected antacids on morphometric parameters of the gastric mucosa of patients with active duodenal ulcer. Przeglad Lekarski 1991;48(10):642-5. [Polish]

w782 Robins AH et al. Effect of the H2-receptor antagonist ranitidine on depression and anxiety in duodenal ulcer patients. Postgrad Med J 1984;60:353-5.

w783 Sheers R et al. Ranitidine and duodenal ulceration: a short-term and maintenance study. J Roy Soc Med 1982;75(5):323-6.

w784 Massarrat S et al. Efficacy of tetracycline and metronidazole alone or with ranitidine on the healing of duodenal ulcer and eradication of Helicobacter pylori. A randomized controlled multicenter study. Tetra-Metro-Ran Study Group. Arzneimittel-Forschung 1998;48(6):686-90. [German]

w785 Lanza FL et al. Ranitidine bismuth citrate plus clarithromycin: a dual therapy regimen for patients with duodenal ulcer. Helicobacter 1998;3(3):212-21.

w786 Trautman A et al. Ranitidine in the treatment of peptic ulcer disease. Ann Roy Coll Physic 1981;14(3):189.

w787 Witzel L et al. Effect of rantidine on ulcer healing. Gastroenterology 1981;80(5 II):1318.

w788 Gilinsky NH et al. A multicenter, double-blind, randomized, placebo-controlled comparison of nocturnal roxatidine in the treatment of active duodenal ulcer disease. Am J Gastroenterol 1992;87(7):847-53.

w789 Darle N et al. Treatment of acute massive gastroduodenal haemorrhage with cimetidine in elderly patients. Ann Chir Gynaecol 1984;73(2):64-8. [French]

w790 Siddiqi SM et al. Cimetidine in acute upper gastrointestinal bleeding. Brit Med J 1979;1(6168):954-5.

w791 Stiel D et al. Cimetidine reduces the risk of rebleeding from duodenal ulcers displaying signs of recent haemorrhage. Scand J Gastroentero 1984;19(6):798-801.

w792 Zuckerman G et al. Controlled trial of medical therapy for active upper gastrointestinal bleeding and prevention of rebleeding. Am J Med 1984;76(3):361-6.

w793 Teres J et al. Cimetidine in acute gastric mucosal bleeding: results of a double-blind randomized trial. Digest Dis Sci 1980;25(2):92-6

w794 Carstensen HE et al. Cimetidine for severe gastroduodenal hemorrhage: A randomized controlled trial. Scand J Gastroentero 1980;15(1):103-5.

w795 Hoare AM et al. Cimetidine in bleeding peptic ulcer. Lancet 1979;2(8144):671-3.

w796 Teres J et al. Cimetidine in acute gastric mucosal bleeding: results of a double-blind randomized trial. Digest Dis Sci 1980;25(2):92-6.

w797 Olivero S et al. Double blind evaluation of cimetidine efficacy in stopping bleeding from gastro duodenal peptic lesions. Minerva Chirurgica 1981;36(19):1243-52. [Italian]

w798 Baltas B et al. Randomized prospective trial of cimetidine in the treatment of acute upper gastrointestinal haemorrhage. Acta Chirurgica Hungarica 1984;25(3):147-57.

w799 Kang JY et al. Treatment of acute upper gastro-intestinal bleeding with cimetidine. Scot Med J 1977;22(4):297-8.

w800 Stadler H et al. Conservative therapy of upper gastrointestinal hemorrhage (prospective, randomized, controlled. Wiener Medizinische Wochenschrift 1985 536;135(21):533-4. [German]

w801 Barer D et al. Cimetidine and tranexamic acid in the treatment of acute upper-gastrointestinal-tract bleeding. New Engl J Med 1983;308(26):1571-5.

w802 Galmiche JP et al. Treatment of bleeding due to peptic ulcer with cimetidine. A double blind trial (author's transl). Gastroen Clin Biol 1978;2(10):771-6. [French]

w803 Birnie GG et al. A double-blind randomized trial of cimetidine in acute upper gastrointestinal bleeding. Scand J Gastroenterol 1984;19(7):885-8.

w804 La Brooy SJ et al. Controlled trial of cimetidine in upper gastrointestinal haemorrhage. Gut 1979;20(10):892-5.

w805 Fullarton GM et al. Controlled study of the effects of intravenous famotidine on intragastric pH in bleeding peptic ulcers. Aliment Pharmacol Ther 1991;5(1):77-84.

w806 Walt RP et al. Continuous intravenous famotidine for haemorrhage from peptic ulcer. Lancet 1992;340(8827):1058-62.

w807 Gray BN et al. Controlled clinical trial of ranitidine in bleeding peptic ulcer. Aust NZ J Surg 1986;56(9):723-7.

w808 Ermolov AS et al. Stress-limiting effect of zantac in duodenal ulcer bleeding. Vop Med Khim 1995;41(5):56-9. [Russian]

w809 Coraggio F et al. Somatostatin and ranitidine in the control of iatrogenic haemorrhage of the upper gastrointestinal tract. BMJ 1984;289(6439):224.

w810 Basso N et al. Ranitidine and somatostatin. Their effects on bleeding from the upper gastrointestinal tract. Arch Surg-Chicago 1986;121(7):833-5.

w811 Stave R et al. Prevention of recurrent ulcer bleeding, a multicentre study of the effect of ranitidine and trimipramine over one year. Scand J Gastroentero 1986;124(Suppl.):55-62.

w812 Dawson J et al. Ranitidine in acute upper gastrointestinal haemorrhage. Brit Med J Clin Res Ed 1982;285(6340):476-7.

w813 Murakami K et al. Influence of anti-ulcer drugs used in Japan on the result of (13)C-urea breath test for the diagnosis of Helicobacter pylori infection. J Gastroenterol 2003;38(10):937-41.

w814 Thong-Ngam D et al. Effect of omeprazole, rabeprazole, and rebamipide on the accuracy of urea breath test in patients with Helicobacter pylori infection Asian Biomedicine. 2010;4(2):337-42.

w815 Kojima Y, Umegaki E, Yoda Y, Nouda S, Kuramoto T, Takeuchi T, et al. Prophylactic effect of rebamipide on gastric/small-intestinal mucosal damage induced by low-dose aspirin: A prospective randomized clinical trial. Gastrointestinal Endosc 2012;1):AB259.

w816 Stupnicki T et al. Efficacy and tolerability of pantoprazole compared with misoprostol for the prevention of NSAID-related gastrointestinal lesions and symptoms in rheumatic patients. Digestion 2003;68(4):198-208.

w817 Takayama M et al. A prospective randomized controlled study of a rebamipid monotherapy in the treatment of endoscopic submucosal dissection (ESD)-induced ulcers. J Gastroenerol and Hepatol 2012;27:419.

w818 Matsui, S et al. Effect of rebamipide for endoscopic submucosal dissection (ESD)-induced ulcer in early gastric cancer: A randomized controlled trial. Gastrointestinal Endoscopy 2012 (1):AB237.

w819 Nakamura M et al. Increased immunoreactivity and concentration of basic fibroblast growth factor in lansoprazole-treated gastric mucosa. J Clin Gastroenterol 1995;21(Suppl.):9.

w820 Song KH et al. Healing effects of rebamipide and omeprazole in Helicobacter pylori-positive gastric ulcer patients after eradication therapy: a randomized double-blind, multinational, multi-institutional comparative study. Digestion. 2011;84(3):221-9.

w821 Tunggal P et al. Effect of esomeprazole versus famotidine on platelet inhibition by clopidogrel: a double-blind, randomized trial. Am Heart J 2011;162(5):870-4.

w822 Ng FH et al. Esomeprazole compared with famotidine in the prevention of upper gastrointestinal bleeding in patients with acute coronary syndrome or myocardial infarction. Am J Gastroenterol 2012;107(3):389-96.

w823 Norman Hansen AH et al. A randomised prospective study comparing the effectiveness of esomeprazole treatment strategies in clinical practice for 6 months in the management of patients with symptoms of gastroesophageal reflux disease. Int J 2005;59:665-71.

w824 Hansen AN et al. Long-term management of patients with symptoms of gastro-oesophageal reflux disease - a Norwegian randomised prospective study comparing the effects of esomeprazole and ranitidine treatment strategies on health-related quality of life in a general practitioners setting. Int J Clin Pract 2006;60(1):15-22.

References for trials listed in Webtables 1-7

w825 Kilic ZM et al. Moxifloxacine plus amoxicillin and ranitidine bismuth citrate or esomeprazole triple therapies for Helicobacter pylori infection. Digest Dis Sci 2008;53(12):3133-7.

w826 Nadeau P et al. Interaction between 2C19*2 gene polymorphism and proton pump inhibitors on platelet reactivity in clopidogrel-treated patients. Can J Cardiol 2012;1):S153.

w827 Miyake K et al. Preventive therapy for non-steroidal anti-inflammatory drug-induced ulcers in Japanese patients with rheumatoid arthritis: the current situation and a prospective controlled-study of the preventive effects of lansoprazole or famotidine. Aliment Pharm Therap 2005;21(Suppl.72):67-72.

w828 Endo M et al. Long-term maintenance treatment of reflux esophagitis resistant to H2-RA with PPI (lansoprazole). Nippon Rinsho - Jpn J Clin Med 2000;58(9):1865-70. [Japanese]

w829 Mine S et al. Ultrasonographic evaluation of lansoprazole-induced improvement of submucosal injury in patients with gastroesophageal reflux. Am J Gastroenterol 2000;95(2):381-6.

w830 Kim N et al. Lafutidine versus lansoprazole in combination with clarithromycin and amoxicillin for one versus two weeks for Helicobacter pylori eradication in Korea. Helicobacter. 2008;13(6):542-9.

w831 Petite JP et al. Lansoprazole versus ranitidine in the treatment of reflux esophagitis. Multicentric study. Med Chir Digest 1991;20(8):462-8. [French]

w832 Bardhan KD et al. Lansoprazole is superior to ranitidine as maintenance treatment for the prevention of duodenal ulcer relapse. Aliment Pharm Therap 1999;13(6):827-32.

w833 Meining A et al. Changes in Helicobacter pylori-induced gastritis in the antrum and corpus during and after 12 months of treatment with ranitidine and lansoprazole in patients with duodenal ulcer disease. Aliment Pharm Therap 1998;12(8):735-40.

w834 Gough AL et al. Lansoprazole versus ranitidine in the maintenance treatment of reflux oesophagitis. Aliment Pharm Therap 1996;10(4):529-39.

w835 Peura DA et al. Lansoprazole for long-term maintenance therapy of erosive esophagitis: double-blind comparison with ranitidine. Digest Dis Sci 2009;54(5):955-63.

w836 Spadaccini A et al. Triple regimens using lansoprazole or ranitidine bismuth citrate for Helicobacter pylori eradication. Aliment Pharm Therap 1998;12(10):997-1001.

w837 Plein K et al. Efficacy of therapy with lansoprazole in patients with acute reflux esophagitis compared with ranitidine. Leber Magen Darm 1997;27(3):145-53. [German]

w838 Robinson M et al. A comparison of lansoprazole and ranitidine in the treatment of erosive oesophagitis. Aliment Pharm Therap 1995;9(1):25-31.

w839 Bardhan KD et al. Lansoprazole versus ranitidine for the treatment of reflux oesophagitis. Aliment Pharm Therap 1995;9(2):145-51.

w840 Feldman M et al. Treatment of reflux esophagitis resistant to H2-receptor antagonists with lansoprazole, a new H+/K+_ATPase inhibitor: A controlled, double-blind study. Am J Gastroenterol 1993;88(8):1212-7.

w841 Jansen JB et al. Standard-dose lansoprazole is more effective than high-dose ranitidine in achieving endoscopic healing and symptom relief in patients with moderately severe reflux oesophagitis. The Dutch Lansoprazole Study Group. Aliment Pharm Therap 1999;13(12):1611-20.

w842 Jones RH et al. Lansoprazole 30mg daily versus ranitidine 150mg b.d. In the treatment of acid-related dyspepsia in general practice. Aliment Pharm Therap 1997;11(3):541-6.

w843 Howden CW et al. Management of heartburn in a large, randomized, community-based study: comparison of four therapeutic strategies.[Erratum appears in Am J Gastroenterol 2001;96(9):2809]. Am J Gastroentero 2001;96(6):1704-10.

w844 Sontag SJ et al. Lansoprazole heals erosive reflux oesophagitis in patients with Barrett's oesophagus. Aliment Pharm Therap 1997;11(1):147-56.

w845 Sontag SJ et al. Lansoprazole heals erosive reflux esophagitis resistant to histamine H2- receptor antagonist therapy. Am J Gastroenterol 1997;92(3):429-37.

w846 Swarbrick ET et al. Prevention of recurrence of oesophageal stricture, a comparison of lansoprazole and high-dose ranitidine. Eur J Gastroen Hepat 1996;8(5):431-8.

w847 Treiber G et al. Age-dependent eradication of Helicobacter pylori with dual therapy. Aliment Pharm Therap 1997;11(4):711-8.

w848 Shimizu Y et al. A proton pump inhibitor, lansoprazole, ameliorates asthma symptoms in asthmatic patients with gastro-esophageal reflux disease. Tohoku J Exp Med 2006;209(3):181-9.

w849 Bate CM et al. Omeprazole is more effective than cimetidine in the prevention of recurrence of GERD-associated heartburn and the occurrence of underlying oesophagitis. Aliment Pharm Therap 1998;12(1):41-7.

w850 Bate CM et al. Comparison of omeprazole and cimetidine in reflux oesophagitis: symptomatic, endoscopic, and histological evaluations. Gut 1990;31(9):968-72.

w851 Thorens J et al. Bacterial overgrowth during treatment with omeprazole compared with cimetidine: A prospective randomised double blind study. Gut 1996;39(1):54-9.

w852 Dehn TCB et al. Double blind comparison of omeprazole (40 mg od) versus cimetidine (400 mg qd) in the treatment of symptomatic erosive reflux oesophagitis, assessed endoscopically, histologically and by 24 h pH monitoring. Gut 1990;31(5):509-13.

w853 Bate CM et al. Omeprazole is more effective than cimetidine for the relief of all grades of gastro-oesophageal reflux disease-associated heartburn, irrespective of the presence or absence of endoscopic oesophagitis. Aliment Pharm Therap 1997;11(4):755-63.

w854 Shimatani T et al. Which has superior acid-suppressive effect, 10mg omeprazole once daily or 20mg famotidine twice daily? Effects of single or repeated administration in Japanese Helicobacter pylori-negative CYP2C19 extensive metabolizers. Digest Dis Sci 2007;52(2):390-5.

w855 Yano H et al. Influence of omeprazole and famotidine on the antiplatelet effects of clopidogrel in addition to aspirin in patients with acute coronary syndromes - A prospective, randomized, multicenter study. Circ J 2012;76(11):2673-80.

w856 Sakurai K et al. Efficacy of omeprazole, famotidine, mosapride and teprenone in patients with upper gastrointestinal symptoms: an omeprazole-controlled randomized study (J-FOCUS). BMC Gastroenterology. 2012;12:42.

w857 Fujiwara Y et al. Famotidine vs. omeprazole: A prospective randomized multicentre trial to determine efficacy in non-erosive gastro-oesophageal reflux disease. Aliment Pharm Therap 2005;21(Suppl.2):10-8.

w858 Wada T et al. Efficacy of famotidine and omeprazole in healing symptoms of non-erosive gastro-oesophageal reflux disease: randomized-controlled study of gastro-oesophageal reflux disease. Aliment Pharm Therap 2005;21(Suppl.9)(2):2-9.

w859 Hu FL et al. Comparison of H2-receptor antagonist- and proton-pump inhibitor-based triple regimens for the eradication of Helicobacter pylori in Chinese patients with gastritis or peptic ulcer. J Int Med Res 2003;31(6):469-74.

w860 Kawano S et al. Randomized comparative study of omeprazole and famotidine in reflux esophagitis. J Gastroen Hepatol 2002;17(9):955-9.

w861 Miner PB, Jr. et al. Comparison of gastric pH with omeprazole magnesium 20.6mg (Prilosec OTC) o.m. famotidine 10mg (Pepcid AC) b.d. and famotidine 20mg b.d. over 14 days of treatment. Aliment Pharm Therap 2007;25(1):103-9.

w862 Soga T et al. Is a proton pump inhibitor necessary for the treatment of lower-grade reflux esophagitis? J Gastroenterol 1999;34(4):435-40.

w863 Guslandi M et al. Acid inhibitors and gastric blood flow. Med Sci Res 1994;22(3):211.

w864 Kim EH et al. Efficacy of Lafutidine Versus Famotidine in Patients with Reflux Esophagitis: A Multi-Center, Randomized, Double-Blind, Non-inferiority Phase III Trial. Dig Dis Sci. 2015;60(6):1724-32.

w865 Arbel Y et al. Platelet inhibitory effect of clopidogrel in patients treated with omeprazole, pantoprazole, or famotidine a prospective randomized crossover study. J Am Coll Cardiol 2012;1):E1410.

w866 Marks RD et al. Omeprazole versus H2-receptor antagonists in treating patients with peptic stricture and esophagitis. Gastroenterology 1994;106(4):907-15.

w867 Kim JW et al. Therapeutic effect of low-dose omeprazole vs. standard-dose ranitidine in mild to moderate reflux esophagitis. Korean J Gastroenterol/Taehan Sohwagi Hakhoe Chi 2004;43(3):153-9. [Korean]

w868 Hallerback B et al. Omeprazole or ranitidine in long-term treatment of reflux esophagitis. The Scandinavian Clinics for United Research Group. Gastroenterology 1994;107(5):1305-11.

w869 Karnes WE et al. Prolonged inhibition of acid secretion causes hypergastrinaemia without altering pH inhibition of gastrin release in humans. Aliment Pharm Therap 1990;4(5):443-56.

w870 Bardhan KD et al. Symptomatic gastro-oesophageal reflux disease: double blind controlled study of intermittent treatment with omeprazole or ranitidine. The European Study Group. Brit Med J 1999;318(7182):502-7.

w871 Jaspersen D et al. Omeprazole MUPS for reflux related chronic persistent cough. Endoskopie Heute 1999;12(2):12-4. [German]

w872 Lauritsen K et al. Omeprazole and ranitidine in the prevention of relapse in patients with duodenal ulcer disease. Can J Gastroenterol 1999;13(10):806-13.

w873 Venables TL et al. Omeprazole 10 milligrams once daily, omeprazole 20 milligrams once daily, or ranitidine 150 milligrams twice daily, evaluated as initial therapy for the relief of symptoms of gastro-oesophageal reflux disease in general practice. Scand J Gastroentero 1997;32(10):965-73.

w874 Yeomans ND et al. A comparison of omeprazole with ranitidine for ulcers associated with nonsteroidal anti-inflammatory drugs. New Engl J Med 1998;338(11):719-26.

w875 Zeitoun P et al. Omeprazole (20 mg od) versus ranitidine (150 mg bd) in reflux esophagitis. Gastroen Clin Biol 1989:13;457-62. [French]

w876 Kaplan-Machlis B et al. Effectiveness and costs of omeprazole vs ranitidine for treatment of symptomatic gastroesophageal reflux disease in primary care clinics in West Virginia. Arch Fam Med 2000;9(7):624-30.

w877 De Rojas FD et al. Omeprazole and ranitidine in long-term treatment of duodenal ulcer: A double-blind comparison of length of time in remission. Digest Dis Sci 1998;43(9):1964-9.

w878 Annibale B et al. Omeprazole in patients with mild or moderate reflux esophagitis induces lower relapse rates than ranitidine during maintenance treatment. Hepato-Gastroenterol 1998;45(21):742-51.

w879 Maton PN et al. Efficacy of omeprazole versus ranitidine for symptomatic treatment of poorly responsive acid reflux disease - a prospective, controlled trial. Aliment Pharm Therap 1999;13(6):819-26.

w880 Angelini G et al. Comparison between omeprazole and ranitidine in medium-term treatment of reflux oesophagitis. Giorn Ital End Dig 1993;16(2):85-9. [Italian]

w881 Hungin APS et al. A comparison of the efficacy of omeprazole 20 mg once daily with ranitidine 150 mg bd in the relief of symptomatic gastro-oesophageal reflux disease in general practice. Brit J Clin Res 1993;4:73-88.

w882 Di Mario F et al. Different doses of omeprazole in the maintenance treatment of patients with peptic ulcers resistant to H2 blockers. Curr Ther Res Clin E 1994;55(11):1363-71.

References for trials listed in Webtables 1-7

w883 Armstrong D et al. Heartburn-dominant, uninvestigated dyspepsia: a comparison of 'PPI-start' and 'H2-RA-start' management strategies in primary care-the CADET-HR Study. Aliment Pharm Therap 2005;21(10):1189-202.

w884 Bianchi Porro G et al. Short-term treatment of refractory reflux esophagitis with different doses of omeprazole or ranitidine. J Clin Gastroenterol 1992;15(3):192-8.

w885 Frame MH et al. Omeprazole produces significantly greater healing of erosive or ulcerative reflux oesophagitis than ranitidine: The Italian Reflux Study Group. Eur J Gastroen Hepat 1991;3(7):511-7.

w886 Festen HP et al. Omeprazole versus high-dose ranitidine in mild gastroesophageal reflux disease: short- and long-term treatment. The Dutch Reflux Study Group. Am J Gastroenterol 1999;94(4):931-6.

w887 Savarino V et al. Effect of gastric acid suppression on 13C-urea breath test: comparison of ranitidine with omeprazole. Aliment Pharm Therap 2000;14(3):291-7.

w888 Smith PM et al. A comparison of omeprazole and ranitidine in the prevention of recurrence of benign esophageal stricture. Restore Investigator Group. Gastroenterology 1994;107(5):1312-8.

w889 Zeitoun P et al. Omeprazole versus ranitidine in erosive oesophagitis. Lancet 1987;2(8559):621-2.

w890 Tham TC et al. Randomised controlled trial of ranitidine versus omeprazole in combination with antibiotics for eradication of Helicobacter pylori. Ulster Med J 1996;65(2):131-6.

w891 Richter JE et al. Omeprazole versus ranitidine or ranitidine/metoclopramide in poorly responsive symptomatic gastroesophageal reflux disease. Am J Gastroenterol 1996;91(9):1766-72.

w892 Revicki DA et al. Health-related quality of life outcomes of omeprazole versus ranitidine in poorly responsive symptomatic gastroesophageal reflux disease. Digest Dis Sci 1998;16(5):284-91.

w893 Dent J et al. Omeprazole v ranitidine for prevention of relapse in reflux oesophagitis. A controlled double blind trial of their efficacy and safety. Gut 1994;35(5):590-8.

w894 Vigneri S et al. A comparison of five maintenance therapies for reflux esophagitis. New Engl J Med 1995;333(17):1106-10.

w895 Sandmark S et al. Omeprazole or ranitidine in the treatment of reflux esophagitis. Results of a double-blind, randomized, Scandinavian multicenter study. Scand J Gastroentero 1988;23(5):625-32.

w896 Figura N et al. Omeprazole versus ranitidine in the prevention of duodenal ulcer recurrence after eradication therapy. Curr Ther Res Clin E 1995;56(6):568-72.

w897 Cavaljuga S et al. Four different treatment protocols for Helicobacter pylori infection-a clinical pilot study. Medicinski Arhiv 2003;57(3):151-6. [Croatian].

w898 Bianchi Porro G et al. High-dose famotidine in the maintenance treatment of refractory esophagitis: results of a "medium-term" open study. Am J Gastroenterol 1991;86(11):1585-7.

w899 Ruth M et al. The effect of omeprazole or rantidine treatment on 24-hour esophageal acidity in patients with reflux esophagitis. Scand J Gastroentero 1988;23(9):1141-6.

w900 Bate CM et al. Reflux oesophagitis resolves more rapidly with omeprazole 20mg once daily than with ranitidine 150mg twice daily: omeprazole 40mg once daily provides further benefit in unresponsive patients. Brit J Clin Res 1991;2:133-48.

w901 Archambault AP et al. Comparison of omeprazole with ranitidine for treatment of symptoms associated with gastroesophageal reflux disease and uncomplicated duodenal ulcer. Can J Gastroenterol 1996;10(3):156-62.

w902 Silvis SE et al. A randomized blinded comparison of omeprazole and ranitidine in the treatment of chronic esophageal stricture secondary to acid peptic esophagitis. Gastrointest Endosc 1996;43(3):216-21.

w903 Green JRB et al. A comparison of the cost-effectiveness of omeprazole and ranitidine in reflux oesophagitis. Brit J Med Eco 1995;8(3):157-69.

w904 Bergmann JF et al. Comparison of the cost-efficacy ratio of omeprazole and ranitidine in the treatment of reflux esophagitis. Gastroen Clin Biol 1995;19(5):482-6. [French]

w905 Prewett EJ et al. Nocturnal intragastric acidity during and after a period of dosing with either ranitidine or omeprazole. Gastroenterology 1991;100(4):873-7.

w906 Blum AL et al. Comparison of omeprazole and ranitidine in the treatment of reflux esophagitis. New Engl J Med 1986;314(11):716.

w907 Havelund T et al. Omeprazole and ranitidine in treatment of reflux oesophagitis: double blind comparative trial. Brit Med J Clin Res Ed 1988;296(6615):89-92.

w908 Jaspersen D et al. Omeprazole in the treatment of patients with complicated gastro-oesophageal reflux disease. J Gastroen Hepatol 1996;11(10):900-2.

w909 Pellegrini M et al. Is a long-term ranitidine-based triple therapy against Helicobacter pylori only a heritage of the past? A prospective, randomized clinicopharmacological study. Aliment Pharm Therap 2005;22(4):343-8.

w910 Vantrappen G et al. Omeprazole (40mg) is superior to ranitidine in short-term treatment of ulcerative reflux esophagitis. Digest Dis Sci 1988;33(5):523-9.

w911 Hurlimann S et al. Comparison of acid inhibition by either oral high-dose ranitidine or omeprazole. Aliment Pharm Therap 1994;8(2):193-201.

w912 Dammann HG et al. Differences in healing tendency of reflux oesophagitis with omeprazol and ranitidine. Results of an Austrian-German-Swiss multicenter trial. Deut Med Wochenschr 1986;111(4):123-8. [German]

w913 Lundell L et al. Prevention of relapse of reflux oesophagitis after endoscopic healing: the efficacy and safety of omeprazole compared with ranitidine. Digestion 1991;47(Suppl.):5.

w914 Klinkenberg-Knol EC et al. The effects of omeprazole and ranitidine on 24-hour pH in the distal oesophagus of patients with reflux oesophagitis. Aliment Pharm Therap 1988;2(3):221-7.

w915 Klinkenberg-Knol EC et al. Double-blind multicentre comparison of omeprazole and ranitidine in the treatment of reflux oesophagitis. Lancet 1987;1(8529):349-51.

w916 Ofman JJ et al. The economic impact of the diagnosis of dysplasia in Barrett's esophagus. Am J Gastroenterol 2000;95(10):2946-52.

w917 Peters FTM et al. Endoscopic regression of Barrett's oesophagus during omeprazole treatment; a randomised double blind study. Gut 1999;45(4):489-94.

w918 Peters FT et al. The influence of Helicobacter pylori on oesophageal acid exposure in GERD during acid suppressive therapy. Aliment Pharm Therap 1999;13(7):921-6.

w919 Ahmad MM. Efficacy of Helicobacter pylori eradication therapies on the background of metronidazole resistance in Bangladesh. J Gastroenterology Hepatol 2012;27:22.

w920 Nakamura K et al. Randomized controlled trial: roxatidine vs omeprazole for non-erosive reflux disease. Hepato-gastroenterol 2010;57(99-100):497-500.

w921 Goh KL et al. Roxithromycin in the eradication of Helicobacter pylori. Am J Gastroenterol 1994;89(11):2099-100.

w922 Ng F et al. Famotidine is inferior to pantoprazole in preventing recurrence of aspirin-related peptic ulcers or erosions. Gastroenterology 2010;138(1):82-8.

w923 Maity S et al. Randomized controlled trial of effectiveness of lafutidine versus pantoprazole in uninvestigated dyspepsia. Indian J Pharmacol. 2014;46(5):498-502.

w924 Kovacs TO et al. Comparison of the efficacy of pantoprazole vs. nizatidine in the treatment of erosive oesophagitis: a randomized, active-controlled, double-blind study. Aliment Pharm Therap 2002;16(12):2043-52.

w925 Armstrong D et al. Symptom relief in gastroesophageal reflux disease: A randomized, controlled comparison of pantoprazole and nizatidine in a mixed patient population with erosive esophagitis or endoscopy-negative reflux disease. Am J Gastroenterol 2001;96(10):2849-57.

w926 Pare P et al. Pantoprazole rapidly improves health-related quality of life in patients with heartburn: a prospective, randomized, double blind comparative study with nizatidine. J Clin Gastroenterol 2003;37(2):132-8.

w927 Metz DC et al. Pantoprazole maintenance therapy prevents relapse of erosive oesophagitis. Aliment Pharm Therap 2003;17(1):155-64.

w928 Richter JE et al. Prevention of erosive oesophagitis relapse with pantoprazole. Aliment Pharm Therap 2004;20(5):567-75.

w929 Talley NJ et al. Randomised controlled trial of pantoprazole versus ranitidine for the treatment of uninvestigated heartburn in primary care. Med J Australia 2002;177(8):423-7.

w930 Van ZJ et al. Efficacy and safety of pantoprazole versus ranitidine in the treatment of patients with symptomatic gastroesophageal reflux disease. Digestion 2004;70(1):61-9.

w931 Ramirez-Barba EJ et al. Low-dose pantoprazole (20 mg) is effective for treatment of mild reflux oesophagitis. Clin Drug Invest 1999;18(6):445-51.

w932 Adamek RJ et al. Relapse prevention in reflux oesophagitis with regard to Helicobacter pylori status: a double-blind, randomized, multicentre trial to compare the efficacy of pantoprazole versus ranitidine. Eur J Gastroent Hepat 2001;13(7):811-7.

w933 Dettmer A et al. Pantoprazole 20mg is effective for relief of symptoms and healing of lesions in mild reflux oesophagitis. Aliment Pharm Therap 1998;12(9):865-72.

w934 Kaspari S et al. Comparison of pantoprazole 20mg to ranitidine 150mg b.i.d. In the treatment of mild gastroesophageal reflux disease. Digestion 2001;63(3):163-70.

w935 Van Zyl JH et al. Efficacy and tolerability of 20mg pantoprazole versus 300mg ranitidine in patients with mild reflux-oesophagitis: a randomized, double-blind, parallel, and multicentre study. Eur J Gastroen Hepat 2000;12(2):197-202.

w936 Dulbecco P et al. Impact of long-term ranitidine and pantoprazole on accuracy of [13C]urea breath test. Digest Dis Sci 2003;48(2):315-21.

w937 Duvnjak M et al. Efficacy of pantoprazole compared with ranitidine in the treatment of gastroesophageal reflux disease: An open-labeled randomized parallel and longitudinal comparative trial. Pharmaca 2002;40(3-4):199-206.

w938 Awad RA et al. Pantoprazole effectively controls intra-oesophageal pH and promotes oesophageal healing: further evidence for ranitidine-induced tolerance in patients with gastro-oesophageal reflux disease. Clin Drug Invest 2001;21(4):265-72.

w939 Teyssen S et al. Reliable acid suppression with pantoprazole contrasts with rapid development of tolerance to ranitidine in healthy individuals. Clin Drug Invest 2001;21(4):273-9.

w940 Koop H et al. Comparative trial of pantoprazole and ranitidine in the treatment of reflux esophagitis: Results of a German multicenter study. J Clin Gastroenterol 1995;20(3):192-5.

w941 Meneghelli UG et al. Efficacy and tolerability of pantoprazole versus ranitidine in the treatment of reflux esophagitis and the influence of Helicobacter pylori infection on healing rate. Dis Esophagus 2002;15(1):50-6.

w942 Armbrecht U et al. Treatment of reflux oesophagitis of moderate and severe grade with ranitidine or pantoprazole-comparison of 24-hour intragastric and oesophageal pH. Aliment Pharm Therap 1997;11(5):959-65.

w943 Gallo S et al. Clinical superiority of pantoprazole over ranitidine in the treatment of reflux esophagitis grade II and III. A prospective, double-blind, double-placebo study. Mexican clinical experience. Mexican Pantoprazole Study Group. Rev Gastroenterol Mex 1998;63(1):11. [Spanish].

w944 Parri MS et al. Pantoprazole significantly interferes with antiplatelet effect of clopidogrel: results of a pilot randomized trial. Int J Card 2013;167(5):2177-81.

References for trials listed in Webtables 1-7

w945 Demir M et al. Efficacy of two different Helicobacter pylori eradication regimens in patients with type 2 diabetes and the effect of Helicobacter pylori eradication on dyspeptic symptoms in patients with diabetes: a randomized controlled study. Am J Med Sci 2009;338(6):459-64.

w946 Chan FKL et al. Proton-pump inhibitor (PPI) versus histamine-2 receptor antagonist (H2RA) for the prevention of recurrent upper gastrointestinal bleeding (UGI) in high-risk users of low-dose aspirin (ASA): An interim analysis. Gastroenterology 2014;(1):S-208.

w947 Park Y et al. Effect of rabeprazole versus famotidine on the pharmarcodynamics of clopidogrel. EuroIntervention 2013;9:18.

w948 Dewan B et al. Lafutidine 10 mg versus rabeprazole 20 mg in the treatment of patients with heartburn-dominant uninvestigated dyspepsia: A randomized, multicentric trial. Gastroenterol Res Pract 2011;640685.

w949 Jee SR et al. A randomized, comparative study of rabeprazole vs. ranitidine maintenance therapies for reflux esophagitis-multicenter study. Korean J Gastroenterol/Taehan Sohwagi Hakhoe Chi 2005;45(5):321-7. [Korean]

w950 Jeong HY et al. A randomized, prospective, comparative, multicenter study of rabeprazole and ranitidine in the treatment of reflux esophagitis. Korean J Gastroenterol/Taehan Sohwagi Hakhoe Chi 2006;47(1):15-21. [Korean]

w951 Farley A et al. Rabeprazole versus ranitidine for the treatment of erosive gastroesophageal reflux disease: a double-blind, randomized clinical trial. Raberprazole Study Group. Am J Gastroenterol 2000;95(8):1894-9.

w952 Dai RZ et al. Clinical effects of mosapride plus ranitidine vs mosapride plus rabeprazole for treatment of esophageal reflux laryngitis. World Chinese Journal of Digestology 2015;23(6):954-8. [Chinese]

w953 Goldstein JL et al. Healing of gastric ulcers with esomeprazole versus ranitidine in patients who continued to receive NSAID therapy: a randomized trial. Am J Gastroenterol 2005;100:2650-7.

w954 Goldstein JL et al. Clinical trial: healing of NSAID-associated gastric ulcers in patients continuing NSAID therapy - a randomized study comparing ranitidine with esomeprazole. Aliment Pharm Therap 2007;26(8):1101-11.

w955 Aoyama N et al. Evaluation of gastric ulcer healing by lansoprazole by measurement of ulcer diameter. J Clin Gastroenterol 1995;20(Suppl. 2):86-9.

w956 Tsuji S et al. Gastric ulcer healing and basic fibroblast growth factor: effects of lansoprazole and famotidine. J Clin Gastroenterol 1995;20(Suppl.):1-4.

w957 Sakaguchi M et al. Suppressive action of lansoprazole on gastric acidity and its clinical effect in patients with gastric ulcers: Comparison with famotidine. J Clin Gastroenterol 1995;20(Suppl. 2):27-31.

w958 Okai T et al. Comparison of lansoprazole and famotidine for gastric ulcer by endoscopic ultrasonography: A preliminary trial. J Clin Gastroenterol 1995;20(Suppl. 2):32-5.

w959 Hotz J et al. Lansoprazole versus famotidine: efficacy and tolerance in the acute management of duodenal ulceration. Aliment Pharmacol Ther 1992;6(1):87-95.

w960 Nema H et al. Comparative study of therapeutic effects of PPI and H2RA on ulcers during continuous aspirin therapy. World J Gastroentero. 2010;16(42):5342-6.

w961 Nema H et al. Effects of antacids on gastroduodenal mucosal injuries caused by low-dose aspirin. Ther Res 2008;29(11):1941-5. [Japanese]

w962 Londong W et al. Dose-related healing of duodenal ulcer with the proton pump inhibitor lansoprazole. Aliment Pharm Therap 1991;5:245-54.

w963 Licht H et al. Lansoprazole versus ranitidine in the treatment of duodenal ulcers: Results of a multicentric randomized double-blind study in similar groups. Med Chir Digest 1990;19(4):251-5. [French]

w964 Agrawal NM et al. Superiority of lansoprazole vs ranitidine in healing nonsteroidal anti-inflammatory drug-associated gastric ulcers: Results of a double-blind, randomized, multicenter study. Arch Intern Med 2000;160(10):1455-61.

w965 Goldstein JL et al. PPI based healing of NSAID associated ulcers in patients continuing NSAIDs. Am J Gastroenterol 2000;95(9):142.

w966 Michel P et al. Short report: treatment of gastric ulcer with lansoprazole or ranitidine: a multicentre clinical trial. Aliment Pharm Therap 1994;8:119-22.

w967 Capurso L et al. Lansoprazole in the treatment of peptic ulcer disease: A multicentre double-blind study. Gastroenterol Int 1995;8(3):125-32.

w968 Hawkey CJ et al. Improved symptom relief and duodenal ulcer healing with lansoprazole, a new proton pump inhibitor, compared with ranitidine. Gut 1993;34(10):1458-62.

w969 Lazzaroni M et al. Triple therapy with ranitidine or lansoprazole in the treatment of Helicobacter pylori-associated duodenal ulcer. Am J Gastroenterol 1997;92(4):649-52.

w970 Bardhan KD et al. Rapid healing of gastric ulcers with lansoprazole. Aliment Pharm Therap 1994;8(2):215-20.

w971 Vreer M et al. Modeling the cost-effectiveness of duodenal ulcer treatment with lansoprazole and ranitidine. Journal of Research in Pharmaceutical Economics 1999 9(4);43-61.

w972 Fujino MA et al. Quality of peptic ulcer healing induced by lansoprazole and roxatidine. J Clin Gastroenterol 1995;20 Suppl 2:S44-6.

w973 Imaeda, H., et al. (2011). Effect of lansoprazole versus roxatidine on prevention of bleeding and promotion of ulcer healing after endoscopic submucosal dissection for superficial gastric neoplasia. J Gastroenterol 46(11):1267-1272.

w974 Bigard MA et al. Comparative efficacy of omeprazole and cimetidine in the treatment of duodenal ulcer in the acute stage. A French multicenter, controlled therapeutic trial. Gastroen Clin Biol 1987;11(11):753-7. [French]

w975 Archambault AP et al. Omeprazole (20mg daily) versus cimetidine (1200mg daily) in duodenal ulcer healing and pain relief. Gastroenterology 1988;94(5 I):1130-4.

w976 Wilairatana S et al. Omeprazole or cimetidine once daily for the treatment of duodenal ulcers? J Gastroen Hepatol 1989;4(Suppl.2):45-52.

w977 Crowe JP et al. Symptom relief and duodenal ulcer healing with omeprazole or cimetidine. Aliment Pharm Therap 1989;3(1):83-91.

w978 Pan S et al. Histological maturity of healed duodenal ulcer and ulcer recurrence after treatment with omeprazole or cimetidine. J Gastroen Hepatol 1994;9(Suppl.):584-7.

w979 Kim NY et al. The effect of eradication of Helicobacter pylori upon the duodenal ulcer recurrence-a 24 month follow-up study. Korean Journal of Internal Medicine 1994;9(2):72-9.

w980 Bate CM et al. Randomised, double blind comparison of omeprazole and cimetidine in the treatment of symptomatic gastric ulcer. Gut 1989;30:1323-8.

w981 Lauritsen K et al. Effect of omeprazole and cimetidine on duodenal ulcer: A double-blind comparative trial. New Engl J Med 1985;312(15):958-61.

w982 Wulff HR et al. Omeprazole and cimetidine in the treatment of ulcers of the body of the stomach: A double blind comparative trial. Brit Med J 1989;298(6674):645-7.

w983 Dahlgren S et al. The effects of omeprazole and cimetidine on duodenal ulcer healing and the relief of symptoms. Aliment Pharm Therap 1988;2(6):483-92.

w984 Lauritsen K et al. Omeprazole in the treatment of prepyloric ulcer: Review of the results of the danish omeprazole study group. Scand J Gastroentero 1989;24(166)(Suppl.):54-7.

w985 Popovic O et al. Omeprazole 20 mg o.m. vs. famotidine 40 mg h.s. in the treatment of duodenal ulcer. Multicenter double-blind randomized trial. Gastroentero Arh1990;9(1):26-34. [Serbian]

w986 Lin C-K et al. Efficacy of omeprazole versus famotidine in the short-term treatment of gastric ulcer. Chinese J Gastroenterol 1994;11:272-78.

w987 Esaki M et al. Effects of omeprazole and famotidine on fibroblast growth factor-2 during artificial gastric ulcer healing in humans. Eur J Gastroen Hepat 2002;14(4):365-9.

w988 Delle Fave G et al. Omeprazole versus famotidine in the short-term treatment of duodenal ulcer disease. Aliment Pharm Therap 1992;6(4):469-78.

w989 Kumar TR et al. Comparative study of omeprazole and famotidine in the treatment of duodenal ulcer. Indian Journal of Gastroenterology 1992;11(2):73-5

w990 Misra SC et al. Omeprazole versus famotidine in the healing and relapse of duodenal ulcer. Aliment Pharm Therap 1993;7(4):443-9.

w991 Yamaguchi Y et al. A prospective randomized trial of either famotidine or omeprazole for the prevention of bleeding after endoscopic mucosal resection and the healing of endoscopic mucosal resection-induced ulceration. Aliment Pharmacol Ther 2005;21(Suppl.2):111-5.

w992 Ye BD et al. Omeprazole may be superior to famotidine in the management of iatrogenic ulcer after endoscopic mucosal resection: a prospective randomized controlled trial. Alimentary Pharmacol Ther 2006;24(5):837-43.

w993 Tomita T et al. Prospective randomized controlled trial to compare the effects of omeprazole and famotidine in preventing delayed bleeding and promoting ulcer healing after endoscopic submucosal dissection. J Gastroenterol Hepatol 2012;27(9):1441-6.

w994 Di Mario F et al. [Effects of H2 blockers and omeprazole on peptic secretion: a prospective, randomized study in duodenal ulcer subjects]. Acta Gastro-ent Belg 1993;56(2):223-8.

w995 Goh KL et al. Helicobacter pylori eradication with short-term therapy leads to duodenal ulcer healing without the need for continued acid suppression therapy. Eur J Gastroen Hepat 1996;8(5):421-3.

w996 Hsu CC et al. Famotidine versus omeprazole, in combination with amoxycillin and tinidazole, for eradication of Helicobacter pylori infection. Eur J Gastroen Hepat 2001;13(8):921-6.

w997 Wang, K., et al. (1996). "Omeprazole plus amoxicillin versus triple therapy eradicates Helicobacter pylori in the Chinese with peptic ulcer disease." Chung Hua i Hsueh Tsa Chih - Chinese Medical Journal 57(3):184-190.

w998 Hui W-M et al. Omeprazole and ranitidine in duodenal ulcer healing and subsequent relapse: A randomized double-blind study with weekly endoscopic assessment. J Gastroen Hepatol 1989;4(Suppl.):35-43.

w999 Hui WM et al. Parietal cells in duodenal ulcer disease: a histochemical study of the effects of omeprazole and ranitidine on mitochondrial activities. J Gastroen Hepatol 1989;4(2):143-9.

w1000 Marks IN et al. A comparison of omeprazole and ranitidine for duodenal ulcer in South African patients. A multi-racial study. Digest Dis Sci 1991;36(10):1395-400.

w1001 Valenzuela JE et al. U.S. experience with omeprazole in duodenal ulcer. Multicenter double-blind comparative study with ranitidine. The Omeprazole DU Comparative Study Group. Digest Dis Sci 1991;36(6):761-8.

w1002 Walan A et al. Effect of omeprazole and ranitidine on ulcer healing and relapse rates in patients with benign gastric ulcer. New Engl J Med 1989;320(2):69-75.

w1003 Bergmann JF et al. Prognostic factors for duodenal ulcers healing. A ranitidine versus omeprazole controlled trial. Presse Med 1995;24(12):559-62. [French]

w1004 Miszputen SJ et al. Comparative multicentric study between omeprazole and ranitidine in duodenal ulcer. Gastrenterol Endo Digest 1997;16(4):127-34. [Portugese]

w1005 Lee J-S et al. Effect of omeprazole and ranitidine on ulcer healing and relief of symptom in patient with peptic ulcer. J Korean Med Assoc 1991;34(4):420-6. [Korean]

References for trials listed in Webtables 1-7

w1006 Classen M et al. Short-duration treatment of duodenal ulcer with omeprazole and ranitidine: Results of a multi-centre trial in Germany. Deut Med Wochenschr 1985;110(6):210-5. [German]

w1007 Hotz J et al. Treatment of acute peptic gastroduodenal ulcer: omeprazole is superior to ranitidine especially in the early phase of ulcer healing. A prospective controlled randomized serial endoscopy study. Leber Magen Darm 1995;25(4):165-70. [German]

w1008 Hallerback B et al. Quality of life in duodenal ulcer treatment: A comparison of omeprazole and ranitidine in acute and intermittent treatment. Psychol Health Med 1998;3(4):417-27.

w1009 Arber N et al. A multicenter, double-blind, randomized controlled study of omeprazole versus ranitidine in the treatment of duodenal ulcer in Israel. Israel J Med Sci 1994;30(10):757-61.

w1010 Al Karawi MA et al. Double-blind comparative study of omeprazole 20 mg daily and ranitidine 300 mg daily in the treatment of duodenal ulcer. Saudi Med J 1996;17(5):576-80.

w1011 Barbara L et al. Omeprazole vs. ranitidine in the short-term treatment of duodenal ulcer: An Italian multicenter study. Hepato-Gastroenterol 1987;34(5):229-32.

w1012 Ahmed W et al. Omeprazole vs ranitidine in the healing of duodenal ulcer. J Pakistan Med Ass 1993;43(6):111-2.

w1013 Bardhan KD et al. A comparison of two different doses of omeprazole versus ranitidine in treatment of duodenal ulcers. J Clin Gastroenterol 1986;8(4):408-13.

w1014 Lysy J et al. Effect of duodenal ulcer healing induced by omeprazole and ranitidine on the generation of gastroduodenal eicosanoids, platelet-activating factor, pepsinogen A, and gastrin in duodenal ulcer patients. Scand J Gastroentero 1992;27(1):13-9.

w1015 Chelvam P et al. Omeprazole compared with ranitidine once daily in the treatment of duodenal ulcer. J Gastroen Hepatol 1989;4(Suppl.2):53-61.

w1016 Archimandritis A et al. Triple therapy eradicated H. pylori equally in patients pretreated with omeprazole or ranitidine. A 12-month follow-up. J Clin Gastroenterol 1995;20(1):12-6.

w1017 Italian Cooperative Group on Omeprazole. Omeprazole 20mg uid and ranitidine 150mg bid in the treatment of benign gastric ulcer. Hepato-gastroenterol 1991;38(5):400-3.

w1018 Zaterka S et al. Treatment of duodenal ulcer with omeprazole or ranitidine in a Brazilian population: A multicenter double-blind, parallel group study. Am J Gastroenterol 1993;88(3):397-401.

w1019 Crowe JP et al. Speed of symptom relief and predictability of healing in duodenal ulcer disease - A comparison of omeprazole 20mg with ranitidine 300mg daily: Omeprazole 40mg daily provides additional benefit in patients with an incomplete response. Eur J Clin Res 1994;5:141-50.

w1020 Delchier JC et al. Double-blind multicentre comparison of omeprazole 20mg once daily versus ranitidine 150mg twice daily in the treatment of cimetidine or ranitidine resistant duodenal ulcers. Gut 1989;30(9):1173-8.

w1021 Choi KW et al. A double-blind, randomized, parallel group study of omeprazole and ranitidine in Korean patients with gastric ulcer. J Gastroen Hepatol 1994;9(2):118-23.

w1022 Catalano F et al. Omeprazole vs. ranitidine in short-term treatment of Helicobacter pylori positive duodenal ulcer patients. Ital J Gastroenterol 1991;23(1):9-11.

w1023 Pare P et al. Healing of benign gastric and prepyloric ulcers: A prospective, endoscopy-controlled, randomized, double-blind, Canadian multicentre study of omeprazole 20 and 40mg daily and ranitidine 150 mg twice a day. Can J Gastroenterol 1990;4(1):7-12.

w1024 Mulder CJ et al. Omeprazole (20mg o.m.) versus ranitidine (150mg b.d.) in duodenal ulcer healing and pain relief. Aliment Pharm Therap 1989;3(5):445-51.

w1025 Rossini FP et al. Histological aspects and healing rates of gastric ulcers treated with omeprazole 20mg once daily or ranitidine 150mg B.I.D. Panminerva Medica 1989;31(2):94-6. [Italian]

w1026 Wang CY et al. Double-blind comparison of omeprazole 20mg OM and ranitidine 300mg NOCTE in duodenal ulcer: a Taiwan multi-centre study. J Gastroen Hepatol 1992;7(6):572-6.

w1027 McFarland RJ et al. Omeprazole provides quicker symptom relief and duodenal ulcer healing than ranitidine. Gastroenterology 1990;98(2):278-83.

w1028 Classen M et al. Healing rates following omeprazole and ranitidine treatment of gastric ulcer. Results of a German multicenter study. Deut Med Wochenschr 1985;110(16):628-33. [German]

w1029 Glise H et al. Two and four weeks' treatment for duodenal ulcer. Symptom relief and clinical remission comparing omeprazole and ranitidine. Scand J Gastroentero 1991;26(2):137-45.

w1030 Sonnenberg A et al. The effect of antibiotic therapy on bleeding from duodenal ulcer. Am J Gastroenterol 1999;94(4):950-4.

w1031 Diaz Rubio M et al. Comparative multicentric study of omeprazole versus ranitidine in the treatment of duodenal ulcer. Revista Espanola de Enfermedades Digestivas 1991;80(1):12-6. [Spanish]

w1032 Parodi MC et al. Effects of omeprazole on gastric mucus secretion, tissue prostaglandin synthesis, and acid inhibition in patients with duodenal ulcer: Comparison with ranitidine treatment. Curr Ther Res - Clin Exp 1992;52(1):13-23.

w1033 Cataldo MG et al. Treatment of patients with duodenal ulcer positive for Helicobacter pylori infection: Ranitidine or omeprazole associated with colloidal bismuth subcitrate plus amoxicillin. Curr Ther Res Clin E 1996;57(3):168-74.

w1034 Kager L et al. The effect of omeprazole and ranitidine on ulcer healing, relief of symptoms, and incidence of adverse events in the treatment of duodenal ulcer patients. Hepato-Gastroenterol 1991;38(4):287-90.

w1035 Sonnenberg A et al. Cost savings in duodenal ulcer therapy through Helicobacter pylori eradication compared with conventional therapies: results of a randomized, double-blind, multicenter trial. Gastrointestinal Utilization Trial Study Group. Arch Intern Med 1998;158(8):852-60.

w1036 Tulassay Z et al. Omeprazole versus ranitidine in the treatment of resistant duodenal ulcer. Gut 1992;33(6):863.

w1037 Michopoulos S et al. Randomized study comparing omeprazole with ranitidine as anti-secretory agents combined in quadruple second-line Helicobacter pylori eradication regimens. Aliment Pharm Therap 2000;14(6):737-44.

w1038 Cooperative study group. Double blind comparative study of omeprazole and ranitidine in patients with duodenal or gastric ulcer: a multicentre trial. Gut 1990;31(6):653-6.

w1039 Russo A et al. Histologic patterns of omeprazole and nizatidine-healed duodenal ulcers:accuracy of endoscopic diagnosis. Scand J Gastroentero 1994;206(Suppl.):20-4.

w1040 Brummer R-J et al. Effect of nizatidine 300mg at night and omeprazole 20mg in the morning on 24-hour intragastric pH and bacterial overgrowth in patients with acute duodenal ulcer. Digest Dis Sci 1996;41(10):2048-54.

w1041 Nakashima S et al. Usefulness of anti-ulcer drugs for the prevention and treatment of peptic ulcers induced by low doses of aspirin. World J Gastroentero 2009;15(6);727-31.

w1042 Sasakii M et al. The therapeutic effect of proton pump inhibitors on Helicobacter pylori-positive gastric ulcers. J Pharm Pharmacol 1999;51(7):825-30.

w1043 Noh MH et al. Analysis of preventive effect of PPI alone, PPI plus cytoprotective agents and H2RA plus cytoprotective agents on bleeding after endoscopic submucosal dissection: A prospective comparative randomized trial. Gastrointestinal Endoscopy. 2013;1):AB252-AB3.

w1044 Kang, KJ et al. Comparison of a proton pump inhibitor and a H2 receptor antagonist for management of iatrogenic ulcers after endoscopic submucosal dissection of gastric neoplasms. J Gastroen Hepatol 2011; 26;278.

w1045 Jeon TJ. Pantoprazole may be superior to famotidine in the ulcer healing after endoscopic submucosal dissection: A prospective randomized controlled trial. Dig. 2010;22 (4):A45.

w1046 Oh TH et al. Pantoprazole may be superior to famotidine in the ulcer healing after endoscopic submucosal dissection: A prospective randomized controlled trial. Digestion 2010;81 (3):162.

w1047 Seo JY et al. Efficacy of famotidine or pantoprazole for the prevention of delayed bleeding and healing of endoscopic submucosal dissection-induced ulcer: A prospective randomized trial. Gastroenterology 2011;(1):S573.

w1048 Papp J et al. Evaluation of the efficacy of pantoprazole and ranitidine in the treatment of duodenal ulcer. Result of an international multicenter double blind study. Orvosi Hetilap 1997;138(45):2863-6. [Hungarian]

w1049 Schepp W et al. Pantoprazole and ranitidine in the treatment of acute duodenal ulcer. A multicentre study. Scand J Gastroentero 1995;30(6):511-4.

w1050 Cremer M et al. A double-blind study of pantoprazole and ranitidine in treatment of acute duodenal ulcer. A multicenter trial. European Pantoprazole Study Group. Digest Dis Sci 1995;40(6):1360-4.

w1051 Chen TS et al. The efficacy of the third pump inhibitor-pantoprazole-in the short-term treatment of Chinese patients with duodenal ulcer. Hepato-gastroenterol 1999;46(28):2372-8.

w1052 Meneghelli UG et al. Pantoprazole versus ranitidine in the treatment of duodenal ulcer: a multicenter study in Brazil. Am J Gastroenterol 2000;95(1):62-6.

w1053 Judmaier G et al. Comparison of pantoprazole and ranitidine in the treatment of acute duodenal ulcer. Aliment Pharm Therap 1994;8(1):81-6.

w1054 Fric P et al. Treatment of duodenal ulcer with pantoprazole. A multicenter study. Casopis Lekaru Ceskych 1996;135(23):758-61. [Czech]

w1055 Hotz J et al. Pantoprazole is superior to ranitidine in the treatment of acute gastric ulcer. Scand J Gastroentero 1995;30(2):111-5.

w1056 van Rensburg CJ et al. Improved duodenal ulcer healing with pantoprazole compared with ranitidine: A multicentre study. Eur J Gastroen Hepat 1994;6(8):739-43.

w1057 Dibildox M et al. Superiority of pantoprazole over ranitidine in the treatment of duodenal ulcer. Mexican clinical experience. Mexican Study Group of Pantoprazole in Duodenal Ulcer. Rev Gastroenterol Mex 1996;61(3):193-8. [Spanish]

w1058 Saigenji K et al. Clinical evaluation of Pariet 10 mg tablets in patients with gastric ulcer diseases. Jpn Pharmacol & Ther 2002;30(9):675-93. [Japanese]

w1059 Breiter JR et al. Rabeprazole is superior to ranitidine in the management of active duodenal ulcer disease: Results of a double-blind, randomized North American study. Am J Gastroenterol 2000;95(4):936-42.

w1060 Takeuchi N et al. Gastric ulcer healing after treatment of endoscopic submucosal dissection in Japanese: Comparison of H receptor antagonist and proton pump inhibitor administration. J Biochem Nutrition 2011;49(3):216-221.

w1061 Bai Y et al. Intravenous Esomeprazole for Prevention of Peptic Ulcer Rebleeding: A Randomized Trial in Chinese Patients. Adv Ther 2015;32(11):1160-76.

w1062 Ashida K et al. Hemostatic effect of lansoprazole (AG-1749) injection in patients with upper gastrointestinal hemorrhage - a multicenter, randomized double-blind, clinical trial Jpn Pharmacol & Ther 2007;35(5):503-517. [Japanese]

w1063 Chua RT et al. Intravenous omeprazole prevents rebleeding in peptic ulcer patients with a non-bleeding visible vessel: A preliminary report of a randomized controlled study. Chinese Med J-Peking 1996;57:139-45.

w1064 Lin HJ et al. Role of intravenous omeprazole in patients with high-risk peptic ulcer bleeding after successful endoscopic epinephrine injection: a prospective randomized comparative trial. Am J Gastroenterol 2006;101(3):500-5.

w1065 Lin HJ et al. A prospective randomized comparative trial showing that omeprazole prevents rebleeding in patients with bleeding peptic ulcer after successful endoscopic therapy. Arch Intern Med 1998;158(1):54-8.

References for trials listed in Webtables 1-7

w1066 Fasseas P et al. Omeprazole versus ranitidine in the medical treatment of acute upper gastrointestinal bleeding: assessment by early repeat endoscopy. Int J Clin Pract 2001;55(10):661-4.

w1067 Artal A et al. Evaluation of intravenous ranitidine and omeprazole effect on the 24-hour gastric ph-metry in duodenal ulcer hemorrhage. Rev Esp Enferm Dig 1996;88(3):191-6. [Spanish]

w1068 Lanas A et al. Effect of parenteral omeprazole and ranitidine on gastric pH and the outcome of bleeding peptic ulcer. J Clin Gastroenterol 1995;21(2):103-6.

w1069 Sheu BS et al. Impact of intravenous omeprazole on Helicobacter Pylori eradication by triple therapy in patients with peptic ulcer bleeding. Aliment Pharm Therap 2002;16(1):137-43.

w1070 Cardi M et al. Intravenous omeprazole versus intravenous ranitidine in the treatment of hemorrhagic duodenal ulcer: a prospective randomized study. Ann Chir 1997;51(2):136-9. [French]

w1071 Coraggio F et al. Somatostatin in the prevention of recurrent bleeding after endoscopic haemostasis of peptic ulcer haemorrhage: a preliminary report. Eur J Gastroen Hepat 1998;10(8):673-6.

w1072 Orti E et al. Is upper gastrointestinal bleeding evolution influenced by the used antisecretory? Rev Esp Enferm Dig 1995;87(6):427-30. [Spanish]

w1073 Villanueva C et al. Omeprazole versus ranitidine as adjunct therapy to endoscopic injection in actively bleeding ulcers: a prospective and randomized study. Endoscopy 1995;27(4):308-12.

w1074 Labenz J et al. Efficacy of primed infusions with high dose ranitidine and omeprazole to maintain high intragastric pH in patients with peptic ulcer bleeding: a prospective randomised controlled study. Gut 1997;40(1):36-41.

w1075 Hsu PI et al. Intravenous pantoprazole versus ranitidine for prevention of rebleeding after endoscopic hemostasis of bleeding peptic ulcers. World J Gastroentero 2004;10(24):3666-9.

w1076 van Rensburg C et al. Clinical trial: intravenous pantoprazole vs ranitidine for the prevention of peptic ulcer rebleeding: a multicentre, multinational, randomized trial. Aliment Pharm Therap 2009;29:497-507.

w1077 Jensen DM et al. Continuous infusion of pantoprazole versus ranitidine for prevention of ulcer rebleeding: A U.S. multicenter randomized, double-blind study. Am J Gastroenterol 2006;101(9):1991-9.

w1078 Gashi Z et al. The efficacy of PPI after endoscopic hemostasis in patients with bleeding peptic ulcer and role of Helicobacter pylori. Medicinski Arhiv 2012;66(4):236-9.

w1079 Yildiz C et al. The effects of misoprostol and famotidine on gastric injury caused by long term nonsteroid anti-inflammatory drug usage. Turkish Journal of Gastroenterology 1996;7(3):222-7. [Turkish]

w1080 Yamao, J.-I et al. Assessing the efficacy of famotidine and rebamipide in the treatment of gastric mucosal lesions in patients receiving long-term NSAID therapy (FORCE - Famotidine or rebamipide in comparison by endoscopy). J Gastroenterol 2006;41(12):1178-1185.

w1081 Lauritsen K et al. Enprostil and ranitidine in prevention of duodenal ulcer relapse: one year double blind comparative trial. Brit Med J Clin Res Ed 1987;294(6577):932-4.

w1082 Bardhan KD et al. A comparison of low-dose maintenance treatment with enprostil against ranitidine in the prevention of duodenal ulcer recurrence. Aliment Pharm Therap 1989;3(5):489-97.

w1083 Heijerman HGM et al. Ranitidine compared with the dimethylprostaglandin E2 analogue enprostil as adjunct to pancreatic enzyme replacement in adult cystic fibrosis. Scand J Gastroenterol Suppl 1990;25(178):26-31.

w1084 Raskin JB et al. Misoprostol and ranitidine in the prevention of NSAID-induced ulcers: a prospective, double-blind, multicenter study. Am J Gastroenterol 1996;91(2):223-7.

w1085 Valentini M et al. Nonsteroidal antiinflammatory drugs for cancer pain: comparison between misoprostol and ranitidine in prevention of upper gastrointestinal damage. J Clin Oncol 1995;13(10):2637-42.

w1086 Bianchi Porro G et al. Double-blind, double-dummy endoscopic comparison of the mucosal protective effects of misoprostol versus ranitidine on naproxen-induced mucosal injury to the stomach and duodenum in rheumatic patients. Am J Gastroenterol 1997;92(4):663-7.

w1087 Rampal P et al. Rioprostil and ranitidine in the prevention of duodenal ulcer relapse. Scand J Gastroentero 1989;24(suppl.164):146-51.

w1088 Businger JA et al. Rioprostil, a new prostaglandin E1 analogue, in the once-daily treatment for the prevention of duodenal ulcer recurrence: A comparison with ranitidine. Scand J Gastroentero 1989;24(164)(Suppl.):152-60.

w1089 Frexinos J et al. Efficacy and tolerability of enprostil in the treatment of duodenal ulcer. Comparison with cimetidine. Gastroen Clin Biol 1989;13(2):188-92. [French]

w1090 Winters L et al. Comparison of enprostil and cimetidine in active duodenal ulcer disease: Summary of pooled European studies. Am J Med 1986;81(2A):69-74.

w1091 Jazrawi RP et al. Enprostil, a prostaglandin E2 analogue, in the treatment of duodenal ulcer; a multicentre * comparison with cimetidine. Aliment Pharm Therap 1988;2(4):353-9.

w1092 Carling L et al. Enprostil and cimetidine: comparative efficacy and safety in patients with duodenal ulcer. Scand J Gastroentero 1987;22(3):325-31.

w1093 Unge P et al. The comparative efficacy and safety of enprostil and cimetidine in the treatment of gastric ulcer. Opus Med 1989;34(3):61-4.

w1094 Villalobos Perez JJ et al. Comparative study of enprostil versus cimetidine in patients with duodenal ulcer during 6 weeks of treatment. Revista de Gastroenterologia de Mexico 1987;52(2):95-8. [Spanish]

w1095 Wang BE et al. A multiclinic double-blind, comparative clinical trial on misoprostol in the treatment of duodenal ulcer. Chinese Journal of Internal Medicine 1991;254:30(4):226-9. [Chinese]

w1096 Dajani EZ et al. Misoprostol in the treatment of duodenal ulcers in the People's Republic of China: A comparative, double-blind, multicenter study. Drug Develop Res 1992;27(4):415-23.

w1097 Aenishanslin W et al. Misoprostol and cimetidine in treatment of duodenal ulcer. Schweiz Med Wschr 1985;115(36):1225-31. [German]

w1098 Rachmilewitz D et al. A multicenter international controlled comparison of two dosage regimens of misoprostol with cimetidine in treatment of gastric ulcer in outpatients. Digest Dis Sci 1986;31(Suppl.2)75S-80S.

w1099 Nicholson PA. A multicenter international controlled comparison of two dosage regimes of misoprostol and cimetidine in the treatment of duodenal ulcer in out-patients. Digest Dis Sci 1985;30(Suppl.);71-7.

w1100 Baccaro JC et al. Misoprostol and cimetidine in the treatment of active duodenal ulcer. Multicenter, double-blind, clinico-endoscopic study. The Argentine experience. Acta Gastroentero Latino 1987;17(3):235-40. [Spanish]

w1101 Fich A et al. Comparison of misoprostol and cimetidine in the treatment of gastric ulcer. Israel J Med Sci 1985;21(12):968-72.

w1102 Fich A et al. Comparison of misoprostol and cimetidine in the treatment of duodenal ulcer. Israel J Med Sci 1985;21(2):102-6.

w1103 Petersen HD et al. Prostaglandin E1 (misoprostol) versus cimetidine in the treatment of duodenal ulcer. A controlled clinical trial. Ugeskrift for Laeger 1987;149(15):969-71. [Danish]

w1104 O'Keefe SJ et al. Misoprostol, a synthetic prostaglandin E1 analogue, in the treatment of duodenal ulcers. A double-blind, cimetidine-controlled trial. S Afr Med J 1985;9:321-4.

w1105 Bianchi Porro G et al. Rioprostil in the short-term treatment of duodenal ulcer: a multicentre double-blind trial vs. cimetidine. Scand J Gastroentero 1989;164(Suppl.):219-23.

w1106 Kim YJ et al. Rebamipide may be comparable to H2 receptor antagonist in healing iatrogenic gastric ulcers created by endoscopic mucosal resection: a prospective randomized pilot study. J Korean Med Sci 2010;25(4):583-8.

w1107 Lartigue S et al. Effect of enprostil on duodenal ulcer healing and ulcer recurrence. Comparison with ranitidine. Gastroen Clin Biol 1994;18(6-7):617-22. [French]

w1108 Bianchi Porro G et al. Enprostil vs ranitidine in the short term treatment of benign gastric ulcer. A double-blind placebo-controlled trial. Drug Invest 1992;4(2):143-8.

w1109 Bunce I et al. Platelet function in patients receiving enprostil. Am J Med 1986;81(2A):85-8.

w1110 Bardhan KD et al. A comparison of enprostil and ranitidine in treatment of duodenal ulcer. J Clin Gastroenterol 1988;10(2):137-42.

w1111 Bardhan KD et al. Gastric ulcer healing: A comparison of enprostil versus ranitidine. J Clin Gastroenterol 1991;13(2):157-62.

w1112 Dammann HG et al. Comparative clinical trial of enprostil and ranitidine in the treatment of gastric ulcer. Am J Med 1986;81(2A):80-4.

w1113 Morgan AG et al. A comparison between enprostil and ranitidine in the management of gastric ulceration. Aliment Pharm Therap 1990;4(6):635-41.

w1114 Mackinnon M et al. Enprostil and ranitidine: comparative efficacy and safety in patients with duodenal ulcer. Aust NZ J Med 1987;17(3):316-20.

w1115 Lauritsen K et al. Enprostil and ranitidine in duodenal ulcer healing: double blind comparative trial. Brit Med J Clin Res Ed 1986;292(6524):864-6.

w1116 Goldin E et al. Comparison of misoprostol and ranitidine in the treatment of duodenal ulcer. Israel J Med Sci 1988;24(6):282-5.

w1117 Simjee AE et al. A comparative study of misoprostol and ranitidine in the healing of duodenal ulcers. A double-blind controlled trial. S Afr Med J 1987;72:15-7.

w1118 Gonvers J-J et al. Gastric ulcer:a double blind comparison of 800mcg misoprostol versus 300mg ranitidine. Hepato-gastroenterol 1987; 34(5): 233-5.

w1119 Lavignolle A et al. Rioprostil vs. ranitidine in duodenal ulcer healing: a double-blind multicentre trial. Scand J Gastroentero 1989;24(suppl.164):194-7.

w1120 Lauritsen K et al. Rioprostil and ranitidine in the treatment of prepyloric gastric ulcer. A double-blind comparative trial. Scand J Gastroentero 1989;24(3):368-72.

w1121 Dammann HG et al. A single evening dose of rioprostil, 600 micrograms, in the treatment of acute duodenal ulcers. Scand J Gastroentero 1989;24(164)(Suppl.):215-8.

w1122 Quinton A et al. Treatment of benign gastric ulcer: A comparative clinical trial of rioprostil and ranitidine. Scand J Gastroentero 1989;24(164)(Suppl.):178-83.

w1123 Dammann HG et al. Rioprostil 600 micrograms nocte: high duodenal ulcer healing rates and low incidence of side effects. Adv Prostag Thromb L 1987;17A:303-6.

w1124 Coremans G et al. Efficacy and safety of rioprostil, 300 micrograms b.d., in the treatment of duodenal ulcer: a double-blind, controlled multicentre clinical study vs. ranitidine. Scand J Gastroentero 1989;164(Suppl.):198-205.

w1125 Businger JA et al. Rioprostil, a new prostaglandin E1 analogue, in the once-daily treatment of acute duodenal ulcer: a comparison with ranitidine. Scand J Gastroentero 1989;164(Suppl.):161-7.

w1126 Rutgeerts P et al. Efficacy and safety of rioprostil, 300 micrograms b.d., in the treatment of gastric ulcer: a comparison vs. ranitidine, 150mg b.d, in a randomized multicentre study. Scand J Gastroentero 1989;164(Suppl.):184-90.

w1127 Yang CW et al. Prevention of post-transplant peptic ulcer by misoprostol. Nephron 1996;74(1):131-5.

w1128 Yoshida N et al. Management of recurrence of symptoms of gastroesophageal reflux disease: synergistic effect of rebamipide with 15 mg lansoprazole. Digest Dis Sci 2010;55(12):3393-8.

References for trials listed in Webtables 1-7

w1129 Mizukami K et al. Aspirin-induced small bowel injuries and the preventive effect of rebamipide. World J Gastroenterology 2011;17(46):5117-22.

w1130 Murata H et al. Combination of enprostil and cimetidine is more effective than cimetidine alone in treating gastric ulcer: prospective multicenter randomized controlled trial. Hepato-gastroenterol 2005;52(66):1925-9.

w1131 Saita H et al. Combination therapy with lansoprazole, amoxicillin, and rebamipide for cure of Helicobacter pylori infection and histological gastritis in gastric ulcer patients. Jpn Arch Int Med 1996;43(2):15-25. [Japanese]

w1132 Jang JS et al. Interim analysis of preventive effect of PPI alone, PPI plus rebamipide and H2RA plus rebamipide on bleeding from after endoscopic submucosal dissection : A prospective randomized controlled trial. Gastrointestinal Endoscopy 2012;1: AB236-AB7.

w1133 Araki H et al. Combination of proton pump inhibitor and rebamipide, a free radical scavenger, promotes artificial ulcer healing after endoscopic submucosal dissection with dissection size >40 mm. J Clin Biochem and Nutri 2012;51(3):185-188.

w1134 Kobayashi M et al. Contributing factors to gastric ulcer healing after endoscopic submucosal dissection including the promoting effect of rebamipide. Dig Dis Sci 2012;57(1):119-126.

w1135 Yilmaz S et al. Does adding misoprostol to standard intravenous proton pump inhibitor protocol improve the outcome of aspirin/NSAID-induced upper gastrointestinal bleeding?: a randomized prospective study. Digest Dis Sci 2007;52(1):110-8.
